# Genome Sequencing Variations in the *Octodon degus*, an Unconventional Natural Model of Aging and Alzheimer's Disease

**DOI:** 10.3389/fnagi.2022.894994

**Published:** 2022-06-30

**Authors:** Michael J. Hurley, Claudio Urra, B. Maximiliano Garduno, Agostino Bruno, Allison Kimbell, Brent Wilkinson, Cristina Marino-Buslje, Marcelo Ezquer, Fernando Ezquer, Pedro F. Aburto, Elie Poulin, Rodrigo A. Vasquez, Robert Deacon, Ariel Avila, Francisco Altimiras, Peter Whitney Vanderklish, Guido Zampieri, Claudio Angione, Gabriele Constantino, Todd C. Holmes, Marcelo P. Coba, Xiangmin Xu, Patricia Cogram

**Affiliations:** ^1^Department of Clinical and Movement Neurosciences, UCL Queen Square Institute of Neurology, London, United Kingdom; ^2^Department of Ecological Sciences, Faculty of Sciences, Institute of Ecology and Biodiversity, Universidad de Chile, Santiago, Chile; ^3^Department of Anatomy and Neurobiology, School of Medicine, University of California, Irvine, Irvine, CA, United States; ^4^Department of Food and Drug, University of Parma, Parma, Italy; ^5^Zilkha Neurogenetic Institute, University of Southern California, Los Angeles, CA, United States; ^6^Bioinformatics Unit, Leloir Institute Foundation (FIL), Buenos Aires, Argentina; ^7^Centro de Medicina Regenerativa, Facultad de Medicina, Clínica Alemana-Universidad del Desarrollo, Santiago, Chile; ^8^Biomedical Sciences Research Laboratory, Faculty of Medicine, Universidad Católica de la Santísima Concepción, Concepción, Chile; ^9^Faculty of Engineering and Business, Universidad de las Americas, Santiago, Chile; ^10^Department Molecular Medicine, Scripps Research, La Jolla, CA, United States; ^11^School of Computing, Engineering and Digital Technologies, Teesside University, Middlesbrough, United Kingdom; ^12^Department Physiology & Biophysics, School of Medicine, University of California, Irvine, Irvine, CA, United States; ^13^Department of Psychiatry and Behavioral Sciences, Keck School of Medicine, University of Southern California, Los Angeles, CA, United States

**Keywords:** Alzheimer's disease, aging, genome, *APOE*, amyloids, lipid droplets, *Octodon degus*, drug development

## Abstract

The degu (*Octodon degus*) is a diurnal long-lived rodent that can spontaneously develop molecular and behavioral changes that mirror those seen in human aging. With age some degu, but not all individuals, develop cognitive decline and brain pathology like that observed in Alzheimer's disease including neuroinflammation, hyperphosphorylated tau and amyloid plaques, together with other co-morbidities associated with aging such as macular degeneration, cataracts, alterations in circadian rhythm, diabetes and atherosclerosis. Here we report the whole-genome sequencing and analysis of the degu genome, which revealed unique features and molecular adaptations consistent with aging and Alzheimer's disease. We identified single nucleotide polymorphisms in genes associated with Alzheimer's disease including a novel apolipoprotein E (*Apoe*) gene variant that correlated with an increase in amyloid plaques in brain and modified the *in silico* predicted degu APOE protein structure and functionality. The reported genome of an unconventional long-lived animal model of aging and Alzheimer's disease offers the opportunity for understanding molecular pathways involved in aging and should help advance biomedical research into treatments for Alzheimer's disease.

## Introduction

To understand genetic factors that contribute to aging and with the goal of establishing an unconventional natural (i.e., spontaneous and not through genetic manipulation) animal model that represents the aging process and also the sporadic late-onset form of Alzheimer's disease (AD) we analyzed the whole genome of the long-lived degu (*Octodon degus*) rodent. Degu naturally develop AD-like pathological features like many other mammals such as the guinea pig (Sharman et al., 2013; Bates et al., 2014), marmosets (Ridley et al., 1986; Wenk, 1993), dolphins (Gunn-Moore et al., 2018) and dogs (Jones et al., 2018). Comparative research using non-human non-conventional models of AD helps improve our understanding of dementia.

Degu live in a well-structured social organization, in complex underground burrows. They have a rich vocal repertoire including alarm calls, a diurnal circadian cycle and well-developed prefrontal areas that are sensitive to social deprivation (Vasquez, 1997; Poeggel et al., 2003; Lee, 2004; Jesseau et al., 2008; Ebensperger et al., 2016). In captivity they live ~10 years and adults weigh up to 220 g with an average body length of 12 cm.

More notable is the fact that with age, some, but not all degu, show hallmarks of human age-related diseases such as AD, type 2 diabetes and atherosclerosis (Homan et al., 2010; Ardiles et al., 2012; Hurley et al., 2018; Chang et al., 2021). Wild-caught and wild-type laboratory-outbred (but not laboratory-inbred) degu can spontaneously develop AD-like neuropathology including increased β-amyloid aggregates, tau hyperphosphorylation and neuroinflammation in the brain as part of their natural history (van Groen et al., 2011; Ardiles et al., 2012; Tarragon et al., 2013; Deacon et al., 2015; Salazar et al., 2016; Paushter et al., 2018). Remarkably, the degu and human β-amyloid peptide sequence are 97.5% homologous, with only one amino acid substitution relative to human (Inestrosa et al., 2005).

Degu develop age-dependent cognitive impairments in activities of daily living and by the age of ~3 years, 30% of degu captured from a natural population show impairments in burrowing performance, which was correlated with their expression profiles of AD markers and cytokine and complement component gene expression (Deacon et al., 2015; Altimiras et al., 2017).

Phylogenetically degu and mice are both in the same Rodentia family meaning the degu genome studies we report here in comparison to murine (*Mus musculus*) studies will be particularly valuable for within family comparison of transgenic mouse AD pathways in terms of which AD gene regulatory pathways are common to spontaneous AD-like features in the long-lived degu vs. different transgenic models using short-lived mice (Zhou et al., 2020). It may be the case that different genetic mouse AD models recapitulate some but not all gene regulatory features of spontaneously occurring AD-like features in degu and patients with AD.

Emerging evidence from experimental and epidemiological studies show that both genetic and environmental factors contribute to aging and the onset and progression of late-onset AD. Whole-genome studies provided a much-needed insight into this unconventional model of AD. Here we show several genes involved in inflammation, DNA repair, lipid metabolism and innate immune regulation as potential critical factors in the AD-like pathology that occurs in the degu. Based on the finding that only a proportion of the degu population spontaneously develop AD-like brain pathology, we hypothesized that there are genetic risk factors that mediate AD susceptibility in degu subpopulations. Single nucleotide polymorphisms (SNP) were identified in 19 AD associated genes in the degu genome [see Supplementary Table 3 including *Apoe* (Apolipoprotein E)] (Barzilai et al., 2012). As *APOE* status is the dominant driver of late-onset AD risk in humans (Serrano-Pozo et al., 2021), we characterized in detail the functional impact of a novel missense SNP in exon 3 (Mt4) of the degu *Apoe* (*odApoe*) and its association with late-onset AD-like phenotypes in this model. In addition, signaling pathway analysis indicated common pathways in human and degu aging and AD processes. Collectively, our observations showed that the use of unconventional long-lived animals as a model of AD could shed light into human aging and late-onset AD mechanisms and therefore such a model could be of use in drug development for the treatment of AD.

## Methods

### Animals

Degu from our outbred wild captive degu colony were housed in standard metal cages (50 × 40 × 35 cm) with a layer of wood shavings as bedding, containing a small metal nesting box (25 × 15 × 10 cm with a single entrance) under a controlled photoperiod (7 a.m. to 7 p.m.) and temperature (23°C). Water and a commercial rodent diet (Prolab RMH 3000, USA) were provided *ad libitum*. All animals used were males aged 4.5 years.

Ethical approval for this project was provided by the Institute of Ecology and Biodiversity Ethics Committee, and the experiments were performed in accordance with the UK Scientific Procedures Act (1986) and the NIH Guide for the Care and Use of Laboratory Animals (1978). All animals were handled consistently in accordance with ARRIVE guidelines.

### Behavioral Test of Daily Living: Burrowing

Behavioral assessment of 149 degu was performed to distinguish AD-like from normal degu using the burrowing test as previously described (Deacon et al., 2015).

### Whole-Genome Sequencing

The genomes of 11 degu were sequenced on the Illumina NovaSeq 6000 S4 platform at a sequencing depth of 60% by Quick Biology Inc. (Pasadena, CA 91007, USA). A single library was constructed using the TruSeq Nano DNA Kit (Illumina, CA, USA). Library integrity was evaluated by capillary electrophoresis using the Fragment Analyzer™ Automated CE System (Analytical Advanced Technologies, Iowa, USA) with the DNF-474 High Sensitivity NGS Fragment Analysis Kit (Analytical Advanced Technologies). The NCBI accession number for the whole-genome sequence of the degu was BioProject ID PRJNA623609.

### Genome Mapping and *Apoe* Mutation Evaluation

Genome mapping against the degu genome assembly OctDeg1.0 (GCA_000260255.1) was performed using BWA-MEM software v0.7.17-r1188 (Liu et al., 2015; Wong et al., 2017). We evaluated the variants detected previously by Sanger sequencing of exon 3 of *APOE*. We visualized each mutation in the mapped genomes using IGV v2.5.3 software (https://software.broadinstitute.org/software/igv/) to identify the presence or absence of each mutation.

### Variant Calling and Filtering

The mapped genomes were prepared with Picard-tools v2.16.1 using the AddOrReplaceReadGroups, MarkDuplicates, and CleanSam tools (https://broadinstitute.github.io/picard/). The SNP and INDEL variants were identified using the Haplotype Caller tool in GATK v4.0.9.0. Variants were filtered by variant quality Q > 100 and by variant depth DP > 20.

The last step was the annotation of the unique variant list to the reference genome using the *Octodon degus* GCA_000260255.1 annotation (https://www.ebi.ac.uk/ena/data/view/GCA_000260255) with SnpEff v4.3t tool (Cingolani et al., 2012). The sequences of AD related genes with annotated variants were obtained using the genome annotation deposited in NCBI (https://www.ncbi.nlm.nih.gov/assembly/GCF_000260255.1/). The annotated variants were filtered to keep missense variants. Genes that contain missense variants were obtained using the filtered variant. The OMA browser (https://omabrowser.org/oma/genomePW/) was used to obtain mouse (*Mus musculus*) ortholog genes. Then a gene ontology (GO) enrichment analysis by biological process was carried out using the gene ontology web tool (http://geneontology.org/). The most significant GO terms were selected filtering by FDR <0.05. To reduce redundancy the resulting GO term list was evaluated with REVIGO tool (http://revigo.irb.hr/).

### AD-Related Gene Evaluation

The sequences of the genes XBP1 Msh6, IGF1 and APOE were extracted from the mapped genomes. The predicted variants found using the GATK pipeline were evaluated *in silico* in the mapped genomes using IGV v2.5.3. The protein sequences were aligned using ClustalOmega software (https://www.ebi.ac.uk/Tools/msa/clustalo/) to validate the effects predicted by SnpEff. The amino acid sequences from human and mouse were obtained using Blast (https://blast.ncbi.nlm.nih.gov/Blast.cgi) to align the degu amino acid sequences, the best hit of each species was selected.

### Degu *Apoe* SNP Validation by Amplicon Sequencing

To validate the SNP detected in *odApoe*, exon 3 was sequenced by nested amplicon sequencing in exon 3 of *odApoe* using an Illumina MiSeq Reagent Nano Kit v2 (500 cycles) at the Universidad Andrés Bello, Santiago, Chile.

The SNP frequency was confirmed *via* Sanger sequencing at 137°C by Quick Biology Inc., Pasadena, CA 91007, US.

### Molecular Modeling

The 3-D homology model of *odApoe* was generated using frame 14 of the NMR structure 2L7N. The E213Q and E213K variants were generated starting from the selected 3-D homology model of wild-type *odAPOE* using the wizard mutagenesis tool of PyMol 1.8.2.1 and then refined by applying the same protocol used for the wild-type structure, and the selected structures of wild-type E213E-odAPOE, E213Q-odAPOE, and E213K-odAPOE were prepared for MD simulations (Supplementary Figure 1).

### β-Amyloid Immunohistochemical Staining and Quantification

Right brain hemispheres of degu were fixed in 4% paraformaldehyde, washed in PBS, and incubated in 30% sucrose overnight before sectioning (30 μm) with a freezing microtome (Leica SM2010R). The sections were then transferred into two 24-well plates per hemisphere. One well of sections per hemisphere was selected for subsequent β-amyloid immunochemical staining using mouse anti-β-amyloid (BioLegend clone 6E10, 1:500). Five randomly selected regions of interest (1.93 mm × 1.93 mm each) were assigned to matching coronal sections of the twelve degu of different variant carriers (4 E213K, 3 E213Q, 4 E213E, 1 Q213Q). Plaques were manually counted and plaque counts per cm^2^ were subject to the linear mixed-effect model (LME) statistical analysis, which handles correlated data from repeated measurements from multiple single animal tissue samples (Yu et al., 2022). In the graph, data bars represent mean ± standard error of the mean (SEM), and a statistical significance is considered when *p* ≤ 0.05. ^*^ indicates *p* < 0.05, ^**^ indicates *p* < 0.01, and ^***^ indicates *p* < 0.001.

### *Apoe* E213K Cell Line Generation

H9 human embryonic stem cells were cultured on Geltrex-coated plates and fed daily with mTeSR-1 medium (Stemcell Technologies). When confluent, human embryonic stem cells were transfected with *APOE-targeting* guide RNA (CCGGCCAGCCGCTACAGGAG) and the HDR repair template (ACAGGGCCGCGTGCGGGCCGCCACTGTGGGCTCCCTGGCCGGCCAGCCGCTACAGAAGCGTGCCCAGGCCTGGGGCGAGCGGCTGCGCGCGCGGATGGAGGAGATGGGCAGCCGGA). Following nucleofection, the cells were plated in three wells of a 6-well plate in mTeSR-1 medium supplemented with 10 μM Y-27632 2HCl (Selleckchem). The next day, a 48-h selection period was started with 0.6 μg/ml puromycin. The cells were fed daily with the mTeSR-1 medium until individual colonies became apparent. The colonies were then selected with 0.6 μg/ml puromycin and genotyped using the following primers: 5′-GATGCCGATGACCTGCAGAA – 3′ and 5′ – GCTTCGGCGTTCAGTGATTG – 3′ and restriction enzyme-based genotyping.

### Neuronal Differentiation

The E213K cell line and the wild-type isogenic control line were then differentiated into neurons as previously described (Zhang et al., 2013; Forrest et al., 2017). After 4 weeks of culture, the neurons were fixed with 4% paraformaldehyde/4% sucrose.

### Immunofluorescence Staining and Analysis

Fixed neurons were permeabilized for 15 min with 0.1% PBS-D (Digitonin) and stained with MAP2 and LipidTOX™ Red Neutral Lipid Stain. Coverslips were mounted and imaged using a Zeiss LSM 800 confocal microscope with a 20X objective. At least 50 neurons were imaged per condition. The acquired z-stacks were merged and puncta indicating lipid accumulation were counted using the ImageJ software.

## Results

### Degu Whole-Genome Sequencing Provides Insights Into Aging and AD-Associated Genes

The whole genome of 11 4.5-year-old male degu was sequenced. A 58-fold sequencing depth resulted in 98.9% mapped reads. The genome was mapped to the degu reference genome (GCF_000260255.1) with 259,905 contigs and 7,135 scaffolds published by the Broad Institute in 2012 (OctDeg1.0 assembly). We identified an average of 11,272,248 variants in all the sequenced individuals; among them, there were 9,501,642 SNP and 1,770,605 insertion-deletions (INDEL) at average frequencies of 3.2 SNP/kip and 0.6 INDEL/kip, respectively (Supplementary Table 1).

**Figure 1:**
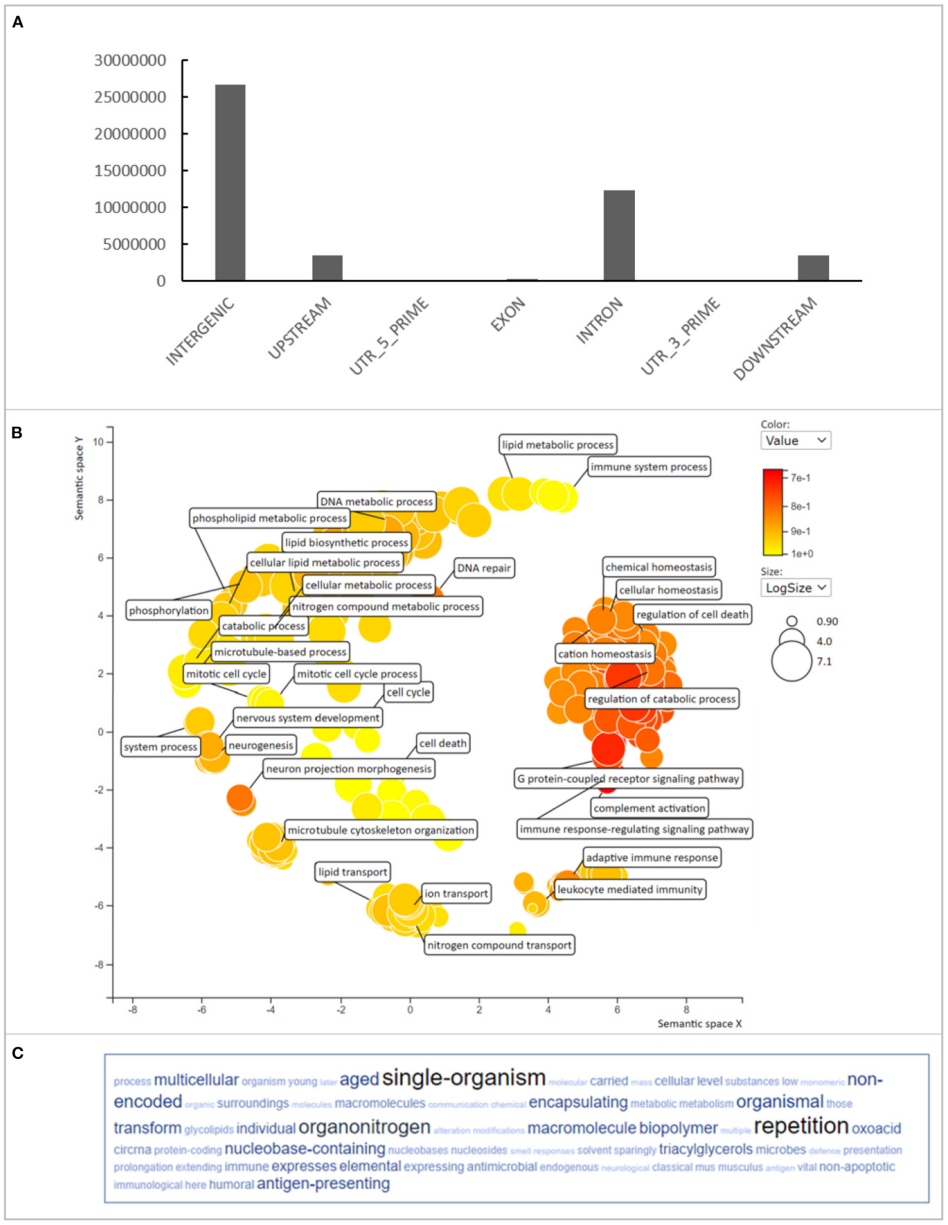
Variant annotation in Degu genomes. **(A)** Represents the variant distribution through the different genomic regions. **(B)** Represents the GO term enrichment of genes that had missense variants found in different Degu genomes. **(C)** Represents the most frequent keywords within the GO terms.

The SNP and INDEL variants identified were mainly (39.9%) located in intergenic regions of the degu genome. While the variants located in coding regions represented just 0.46% of the total, however there were still 307,936 variants located in exons (Figure 1A). Within the exonic variants, 103,362 were variants that caused an amino acid change and could therefore influence protein structure and function. The number of genes that contained missense variants and had orthologous genes with *Mus musculus* was 10,870 (Supplementary Table 2). Gene Ontology (GO) biological pathway enrichment analysis revealed that 355 enriched pathways that were associated with immune-regulation, lipid metabolism, DNA repair mechanisms, apoptosis, translation, glucose metabolism, oxidative stress and cell cycle (Figures 1B,C). These biological processes are highly relevant to aging and aging-related diseases (Barzilai et al., 2012; Tower, 2015; Anisimova et al., 2018).

### X-Box Binding Protein 1

During aging, there is a reduction in the capacity of the cells to control protein folding and aggregation, and activation of the unfolded protein response (UPR) is compromised. The transcription factors X-box binding protein (XBP1) is a basic leucine-zipper (bZIP) transcription factor, a family of proteins that can lead to disease states including neurodegeneration, diabetes and cancer (Cissé et al., 2017).

**Figure 2:**
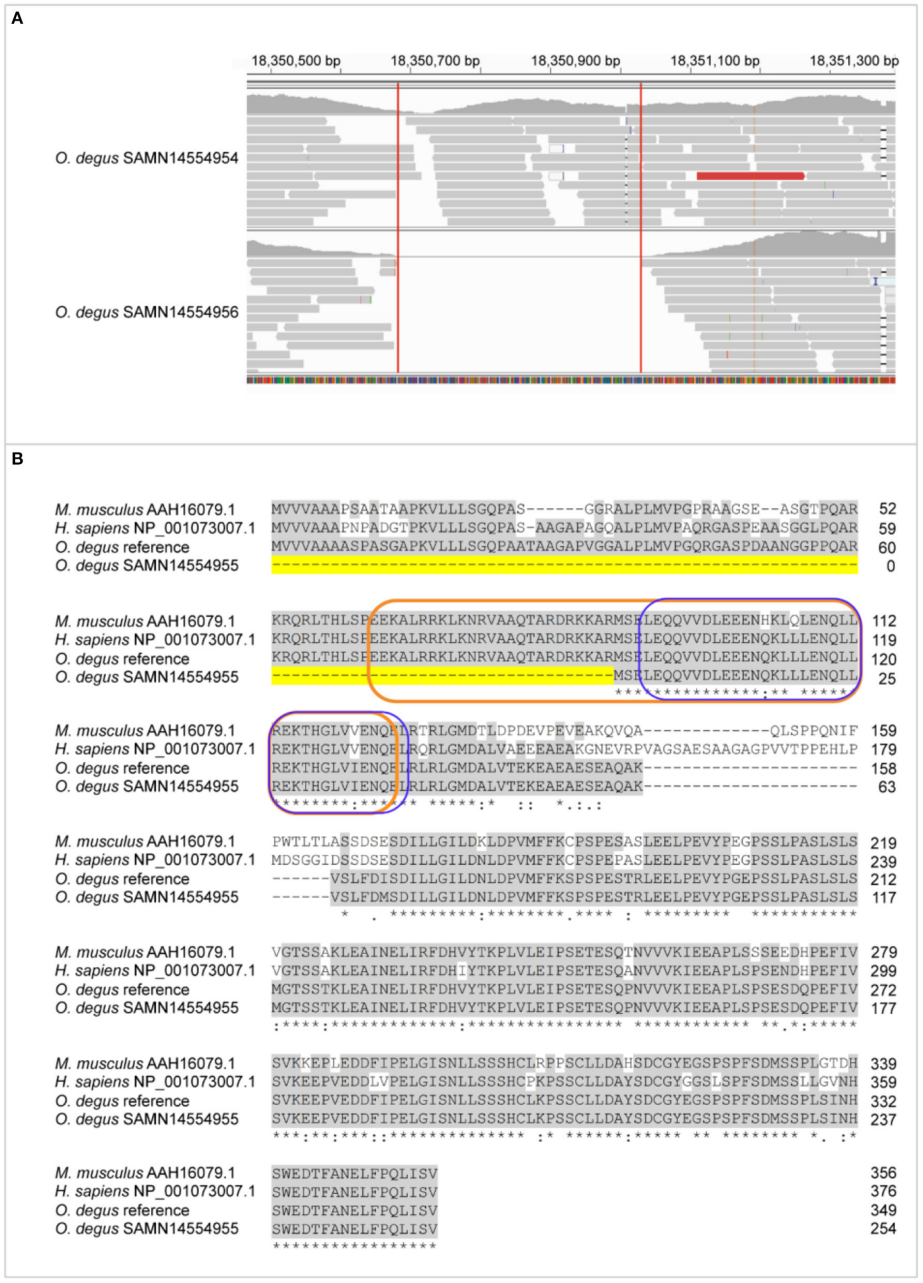
Exon 1 is lost in Xbp1 due to 27bp deletion of bZIP N-terminal. **(A)** Genome mapping of degu SAMN14554954, an animal that had the complete XBP1 sequence and degu SAMN14554956 that carried the exon 1 deletion compared to the degu reference genome GCF000260255 in the XBP1 coding sequence. **(B)** Alignment of amino acid sequence between M. musculus, H. sapiens XBP1s isoform and degu SAMN14554954 and SAMN14554956. Conserved amino acid sequences among all the aligned organism sequences are greyhighlighted. Dashed line highlighted in yellow represents the amino acid deletion of XBP1 in degu SAMN14554956. The bZIP domain is outlined orange and the leucine zipper outlined in blue.

The homology between degu and human and mouse XBP1 protein is 78.8 and 79.1% respectively with a similar homology (77.7%) between human and mouse XBP1. The human *XBP1* gene is spliced by the inositol-requiring enzyme 1 (IRE1) that removes a 26-nucleotide intron from XBP1 to shift the coding reading into a XBP1spliced form (XBP1s) that contains a bZIP domain. XBP1s has physiological roles in the brain and the immune system. In the degu we identified an INDEL of 343bp in *Xbp1*, this INDEL completely deletes exon 1 (Figure 2A) which contains 27 amino acids of the N-terminal bZIP domain (Figure 2B). The lack of exon 1 in the degu carrying this mutation could result in the absence of *Xbp1* unspliced form (*Xbp1u*) isoform due to the high number of amino acids that are removed. Dysregulation of bZIP can lead to neurodegeneration and diabetes. The INDEL we report deletes part of the bZIP domain possibly affecting DNA-binding and dimerization functions in the degu that carry this mutation. Such degu could be an excellent model with which to understand the effect of this mutation in aging, diabetes and AD.

### MutS Homolog 6

Among the GO terms found, DNA repair was one of the enriched biological functions that had genes with variants. Deficiency in repair of nuclear and mitochondrial DNA damage has been linked to aging and neurodegeneration. The MSH6 gene provides instructions for making a protein that plays an essential role in repairing DNA (Marsischky et al., 1996).

**Figure 3:**
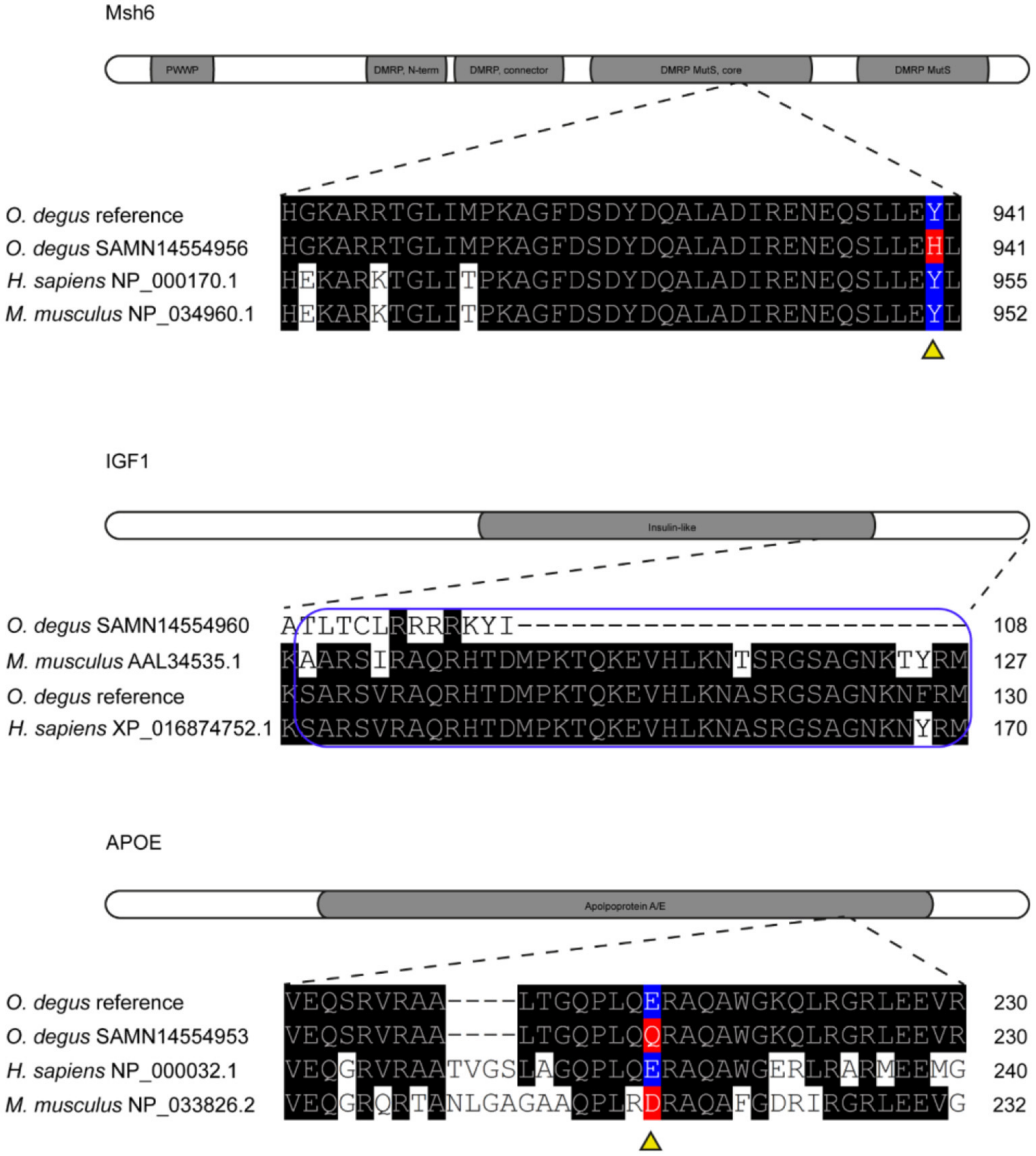
Missense variants in Msh6, IGF1 and APOE genes of degu. Representation of Msh6, IGF1 and APOE genes with missense mutations aligned to degu reference genome annotated gene and human amino acid sequence. Amino acid changes are highlighted in red. Reference amino acids are highlighted in blue. Gray shading shows functional domains of each gene. DMRP correspond to DNA mismatch protein MutS domain. The E-peptide of IGF1 is outlined in blue.

The homology between degu, human and mouse MSH6 amino acid sequences revealed an 85.5% of identity between degu and human (NP_000170.1) and 80.4% identity between degu and mouse (NP_034960.1) and an identity of 85.3% between human and mouse (Figure 3).

We found a SNP in the degu *Msh6* that caused an amino acid change from tyrosine to histidine in 55% of the degu, this residue is also highly conserved between human, degu and mouse (Figure 3). The SNP was in the DNA mismatch repair protein MutS core domain. Interestingly, mutations in MSH6 result in high rates of accumulation of base substitution mutations in humans (Marsischky et al., 1996). Further studies on the structural characterization of the mutation could help elucidate the effect of this mutation in the degu in comparison to humans and its effect in DNA repair and aging mechanisms.

### Insulin-Like Growth Factor 1

The homology between degu and human (XP_016874752.1) IGF1 is 97.9%, whereas, between degu and mouse (AAL34535.1) and human and mouse it is 92.1% and 93.7% respectively We identified an INDEL of 20 bp in exon 2 of the degu *Igf1*. This INDEL causes a frameshift at residue 76 that changed the amino acid sequence (Figure 3). This change in the amino acid sequence resulted in the complete deletion of E-peptide. This peptide is highly conserved (97.3%) between degu and human.

### Apolipoprotein E

The human *APOE* gene has 90.0 % homology with the degu *Apoe* gene. And the degu APOE amino acid sequence has 71.3% and 70.2% homology with the human and mouse APOE protein, respectively.

**Figure 4:**
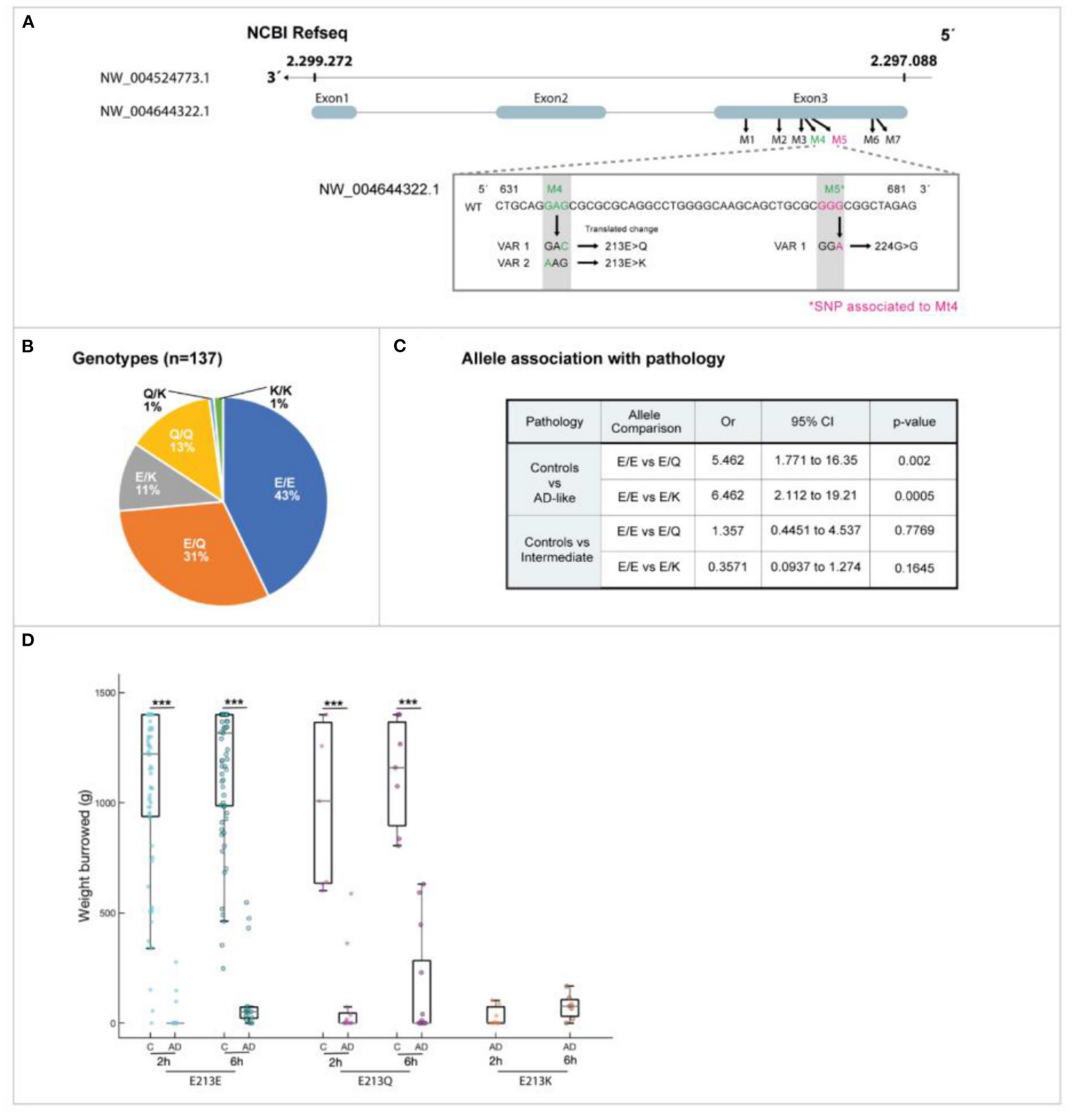
Novel degu *Apoe* (od*Apoe*) mutation: whole-genome sequencing. **(A)** At position 213, there are three possible residues: glutamic acid (Glu: E), glutamine (Gln: Q), or lysine (Lys: K). Variants 1 and 2 are 213 E>Q and 213 E>K, respectively. The neighboring silent SNP was located 109 base pairs upstream of the missense variant (shown in pink). **(B)** The genotype frequency in 137 wild degu. **(C)** Allele association with AD-like pathology in degu. **(D)** There was a significant difference in the association of the variants E213K and 213 E>Q with AD-like burrowing behavior (Mann–Whitney *U* test, *P* < 0.001)

We identified seven SNP in the degu *Apoe* gene compared to the reference sequence (NW_004524773.1), one of which was non-synonymous (denoted Mt4) and found in a 631 bp PCR product amplified from genomic DNA (Figure 4).

The degu *Apoe* Mt4 can yield one of three residues at position 213: glutamic acid (E), which is present in Refseq XP_004644379.1, glutamine (Q), or lysine (K) (Figure 4A). The allele E is conserved in human and degu but in mouse corresponds to aspartic acid. The effect of the degu *Apoe* [E213E], *Apoe* [E213Q] and *Apoe* [E213K] variants in protein structure and function were studied in detail.

### Degu *Apoe* Mt4 SNP Showed a Genetic Variation Across the Degu Population

The degu *Apoe* Mt4 frequency in 137 degu was confirmed by Sanger sequencing and the allele distribution was E213E = 43%, E213Q = 31%, E213K = 11.0%, Q/Q = 13%, Q213K = 1.0%, and K213K = 1.0% (Figure 4B).

### Degu *Apoe* Mt4 SNP Correlated With Deficits in Activities of Daily Living

We evaluated the effect of Mt4 on burrowing, a species-typical task that mirrors “activities of daily living” in patients that decline noticeably in the earliest stages of the AD. The allele E213Q (most frequent) showed a 6-fold correlation with burrowing deficits (OR = 6.46, *P* = 0.0005, Fisher's exact test) and allele E213K was also significantly associated with burrowing deficits by 5-fold (OR = 5.46, *P* = 0.002) (Figures 4C,D).

### Correlation Between the Degu *Apoe* Mt4 SNP and AD-Like Neuropathology

**Figure 5:**
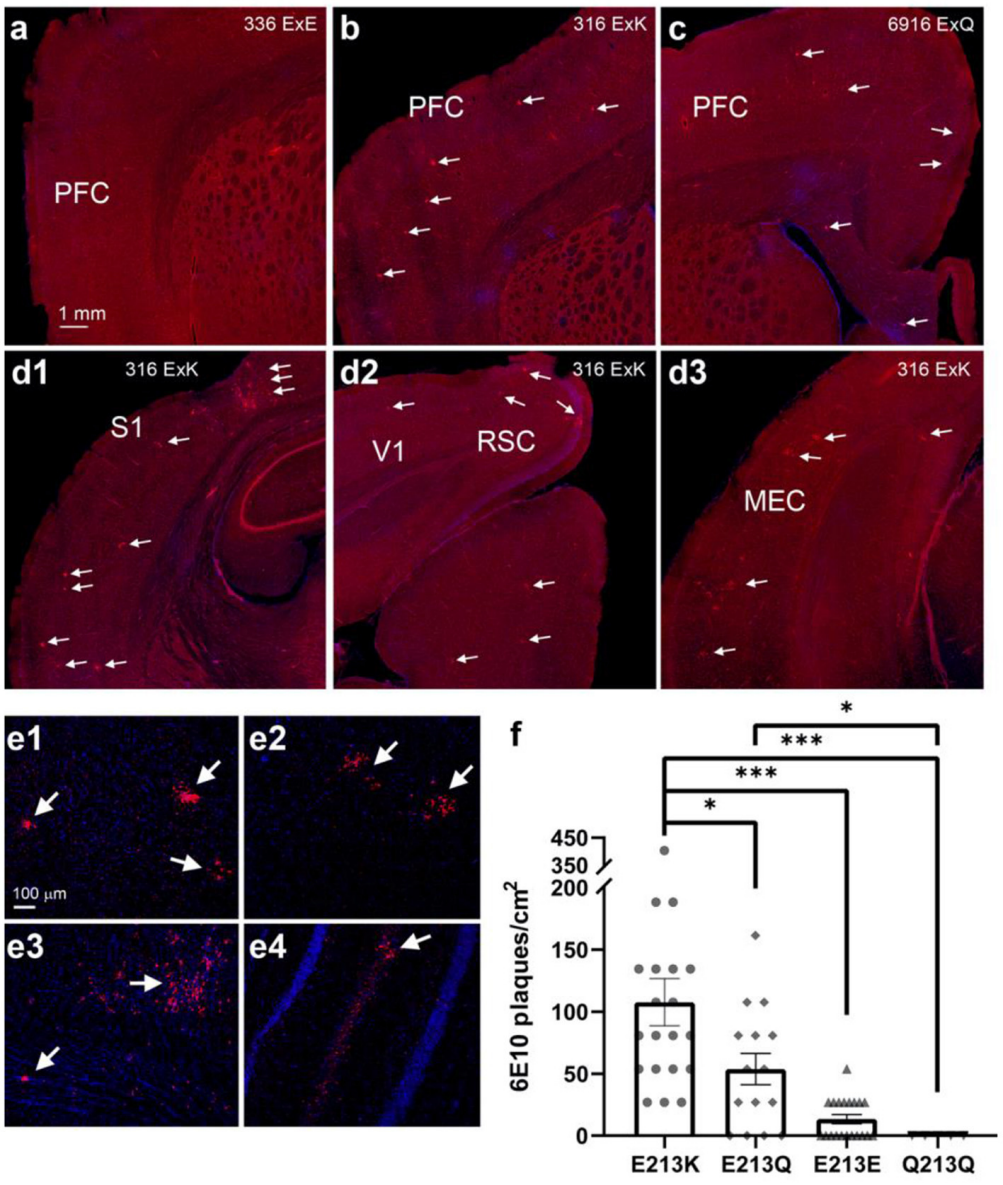
Degu carrying the odAPOE allele E213K presented the highest β-amyloid plaque accumulation in the brain. **(a-c)** PFC comparison of right brain hemispheres of degu presenting three different residue variants: E213E **(a)**, E213K **(b)**, and E213Q **(c)**. Residue variants E213K and E213Q show brain plaque depositions throughout the PFC, while wild-type E213E shows no perceivable plaques. White arrows denote β-amyloid plaques. **(d1-d3)** Widespread β-amyloid plaque depositions seen through various posterior brain regions in an E213K variant carrier. White arrows denote β-amyloid plaques. **(e1-e4)** Confocal micrographs of cortical **(e1-e3)** and hippocampal plaques **(e4)**. **(f)** There were among-variant differences in the number of plaques, with the E213K variant carriers showing significant differences compared with carriers of the other variants (4 E213K cases; 8 other cases; Linear mixed-effect model (LME) tests. (*P < 0.05, ***P < 0.001). PFC, prefrontal cortex; S1, primary somatosensory cortex; V1, primary visual cortex; RSC, retrosplenial cortex; MEC, medial entorhinal cortex.

Because *APOE4* is the strongest genetic risk factor for late-onset AD, with a correspondingly high accumulation of amyloid plaques (Jansen et al., 2015). We investigated whether degu Mt4 carrier subpopulations have more brain β-amyloid deposition. To examine this association, β-amyloid plaques were assessed in the brains of 12 degu (Figure 5). While no significant plaques were observed in the degu with the E213E and Q213Q *Apoe* variants, β-amyloid plaques were robustly observed throughout the cortical regions in the degu with the E213K and E213Q variants (Figures 5a–e). Our quantification of 6E10 immunopositive plaques in the degu brain sections showed that plaque abundance (plaques/cm^2^) differed significantly between the degu with *Apoe* E213K and other variant types (4 E213K cases; 8 other cases; Linear mixed-effect model (LME) tests, E213K vs. E213Q: *p* = 0.0101, E213K vs. E213E: *p* = 7.1919 × 10^−6^, E213K vs. Q213Q: *p* = 0.0003,) (Figure 5f).

### Degu *Apoe* Mt4 SNP Alters Protein Structure

In humans APOE binds lipoproteins by opening its four-helix bundle and exposing hydrophobic, amphipathic sequences to the lipid surface. We generated an *in silico* homologous model structure of the degu *APOE* based on the human APOE protein structure (Frieden et al., 2017) and identified 74% sequence identity between both proteins, denoting a similar structure, function, and evolutionary origin. The degu *Apoe* contains the equivalent of R112 and Glu255 but lacks the critical R61 equivalent (it contains T61). Despite this difference, Mt4 caused flexibility at the salt bridge dislodging helix C2 allowing the approach of E133 and R148 domains with a similar consequential effect to that observed in human APOE.

**Figure 6:**
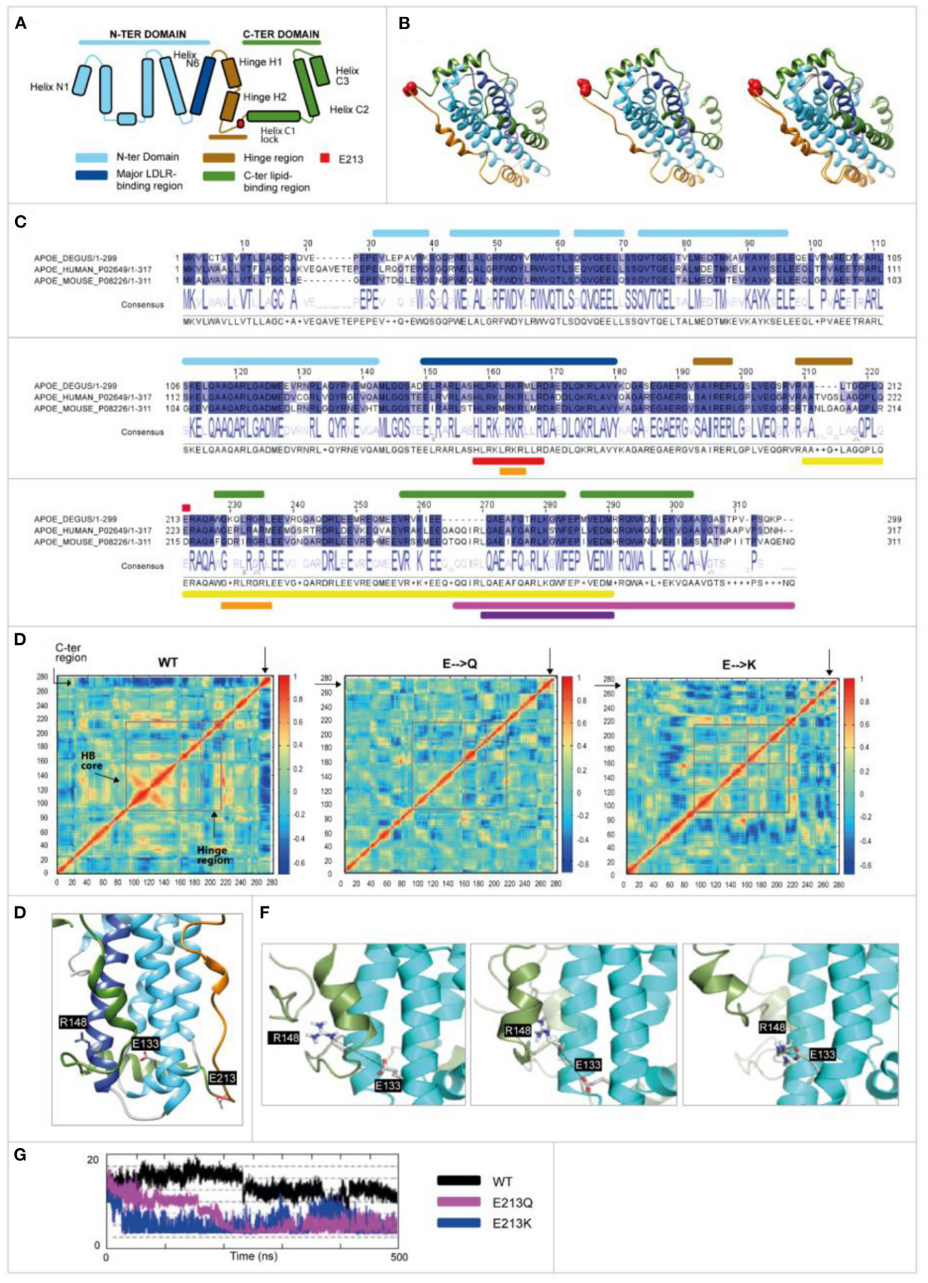
Human and odAPOE isoforms. **(A)** Secondary structural scheme of APOE based on the high-resolution structure of *APOE3*. **(B)** From left to right: ribbon representation of the APOE*3* structure (UniProt code: P02649; PDB code: 2L7B), od*Apoe* wild-type model, and APOE*3*-degu APOE superposition that are colored as shown in the scheme. Degu E213 and human E223 are shown as red spheres. **(C)** Degu, human, and mouse sequence alignment. Upper bars: secondary structure elements are colored as shown in the scheme. Red circle: Glutamic acid 213 (223 human). Lower bars: *APOE* (UniProt code: P02649) functional domains. Red: LDL and other lipoprotein receptor binding; orange: heparin binding; yellow: lipid-binding and lipoprotein association; pink: homo-oligomerization; violet: specificity for association with VLDL. **(D)** Displacement correlation matrix of the molecular dynamics simulation, with blue and red indicating uncorrelated and highly correlated motions, respectively (X- and Y-axis represent the amino acid number). Regions are indicated by gray boxes. The N-terminal helix bundle (HB) core region showed more correlated motions in the wild-type model while the C-terminal region showed more correlated motions with the rest of the protein in the E213Q and E213K models. Moreover, the hinge region showed differences. **(E)** A 3-D structure of wild-type degu *Apoe* showing the positions of the D133, R148, and E213 residues (represented as sticks). **(F)** Sequential representation of the salt bridge generation observed during the simulations. The N-terminal and C-terminal domains are depicted in cyan and green, respectively. The reference position of helix C2 during the wild-type degu APOE simulation is depicted as a cartoon representation in transparent green. **(G)** Distance (À) between D133 and R148 during the simulations (500 ns). The MD-derived distance between both residues is constant and larger in wild-type (black curve) than in E213K (blue) and E213Q (pink).

Schematic representations of the APOE structure, APOE3, and modeled the degu APOE structures and the alignment of degu, human, and mouse sequences of the relevant structural and functional correlates are shown in Figures 6A–C. Both Q and K variants reduced the local negative electrostatic potential (Supplementary Figure 1). The displacement correlation matrix was analyzed, and the three model proteins were found to have significantly different conformational properties (Figure 6D). The wild-type protein showed highly concerted motions at the helix bundle of the N-terminal domain, while the C-terminal end showed displacements that were completely uncorrelated with the rest of the protein. In contrast, for the E213Q and E213K proteins, the concerted motion at the N-terminal domain was no longer present, increasing the N-terminal domain flexibility. Their C-terminal domain showed motion correlated with the rest of the protein (not present in wild-type degu APOE; Figure 6D).

A unique salt bridge at Glu 133-Arg 148 (E133-R148) was generated in the E213Q and E213K protein simulations but not in the wild-type protein. The formation of this salt bridge in the wild-type protein was hindered by the presence of helix C2 (Figure 6E). In the E213Q and E213K simulations, the increased N-terminal and C-terminal flexibility dislodged helix C2, which in turn allowed the approach of E133 and R148 (Figures 6F,G).

### Degu *Apoe* Mt4 Isoforms Modulate Lipid Droplet Formation in Degu and Human Neurons

Because of previous reports linking lipid droplets with AD, aging, inflammation and diabetes in the present work we investigated the presence of lipid droplets in skin fibroblasts from AD patients, age-matched controls and old degu to determine whether alterations in lipid droplets occur in degu that may be relevant to AD-like pathology in the degu model of aging and AD.

**Figure 7:**
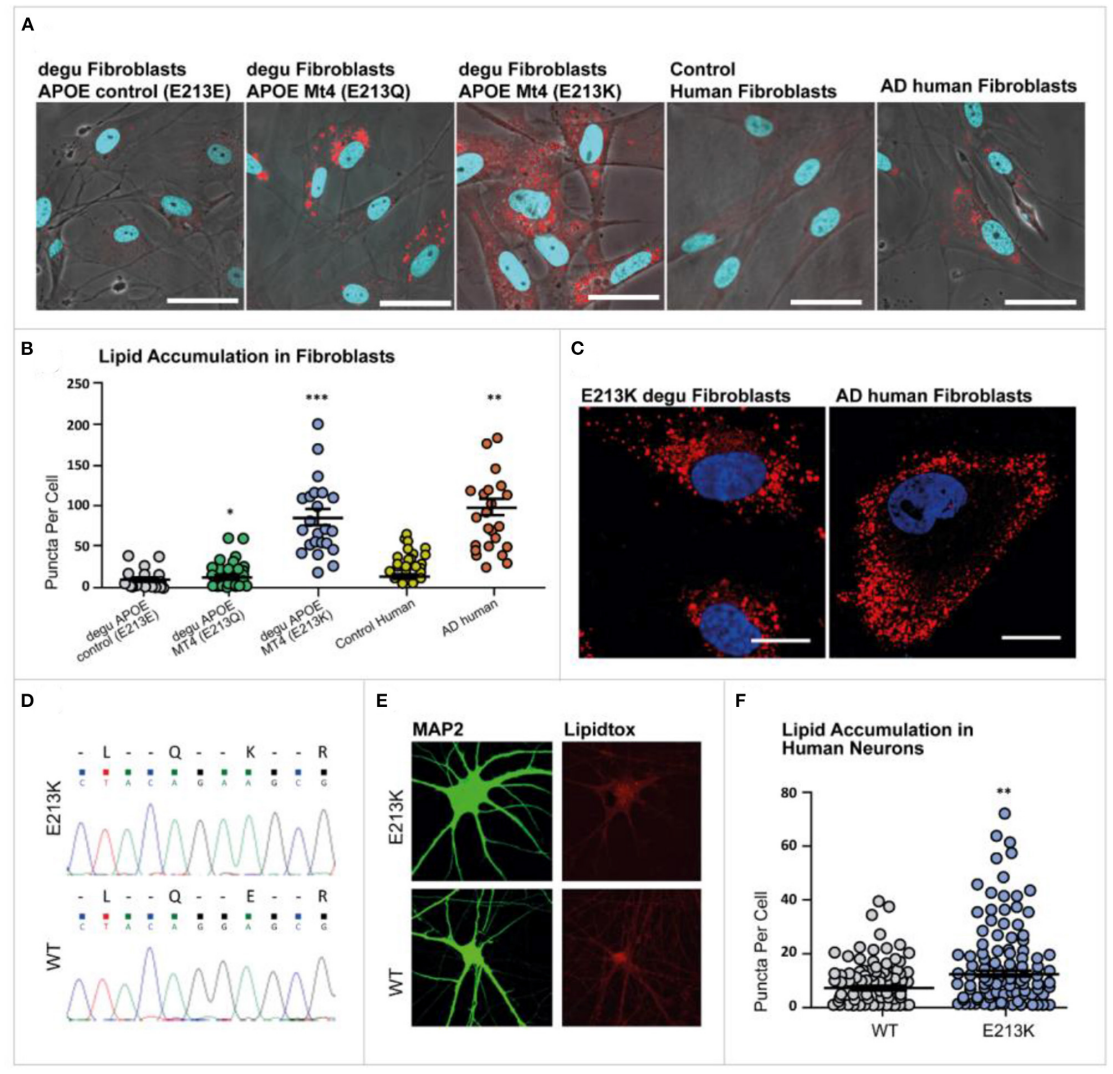
Lipid droplet accumulation in degu and human fibroblasts. Primary cultures of degu and human fibroblasts stained with LipidTOX™ Red Neutral Lipid stain (red), where nuclei were identified through DAPI counterstaining (blue). **(A)** Representative micrographs of E213E, E213Q, and E213K degu fibroblasts, as well as fibroblasts obtained from human controls and patients with AD. **(B)** Lipid droplet quantification in degu and human fibroblasts. **(C)** Representative images of E213K degu fibroblasts and fibroblasts from patients with AD showing prominent lipid droplet accumulation. **(D)** Heterozygous mutant cell line confirmed through Sanger sequencing. **(E)** Neurons were stained using MAP2 and lipid accumulation was visualized with LipidTOX™. **(F)** Compared with neurons derived from isogenic controls, neurons derived for degu and humans carrying od*Apoe* Mt4 E213Q and E223K, respectively, showed a significant increase in lipid puncta (*P* = 0.0095, Student's *t*-test, *n* = 124). od*Apoe* Mt4 and wild-type neurons derived from two independent cultures. The scale bar represents 20 μm.

The skin fibroblasts from AD patients and degu fibroblasts expressing *Apoe* Mt4 (E213K) displayed an evident alteration, namely an anomalous accumulation of lipid droplets in their cytoplasm compared to human control fibroblasts and wild-type (E213E) degu fibroblasts (Figures 7A–C).

To examine the cellular functions of Mt4 in fibroblasts we investigated whether the accumulation of lipid droplets observed in degu fibroblasts was also preserved in human fibroblast cells carrying the degu Mt4 mutation. We generated the orthologous degu *Apoe* Mt4 mutation (213E/213K) in human embryonic stem cells using CRISPR/Cas9 genome editing. A heterozygous mutant cell line was confirmed by Sanger sequencing (Figure 7D). A significant increase in the number of lipid droplets was found in the degu *Apoe* Mt4 neurons compared to non-mutant wild-type neurons derived from isogenic controls (Figures 7E,F *P* = 0.0095, Student's *t*-test, *n* = 124 degu *Apoe* Mt4 neurons and *n* = 113 wild-type neurons derived from two independent cultures). These data show that the degu *Apoe* Mt4 mutation increases lipid droplet accumulation in both human and degu derived neurons and fibroblasts.

## Discussion

The aim of this work was to characterize and validate the unconventional natural degu model of aging and Alzheimer's disease. We use whole-genome sequencing techniques to create a new degu genome annotation that allowed the identification of mutations associated with aging and AD-like pathology in this long-lived animal model. Our research achieved the goal of providing new investigative tools to identify the gaps in current knowledge related to molecular mechanisms of aging and late-onset AD using a long-lived animal model that may assist in the identification of potential therapeutic targets.

We found variants in 14,992 genes (Supplementary Table 2) among the 11 degu genomes evaluated and we report novel variants in 19 genes (Supplementary Table 3) associated with processes involved in aging and AD such as neuroinflammation, protein misfolding, diabetes and DNA-repair genes. From these we selected 4 genes to report that are involved in DNA repair, glucose metabolism, protein misfolding and lipid homeostasis.

Xbp1 is a very complex protein regulating many physiological functions, including immune system, inflammatory responses, lipid metabolism, neurodegeneration, diabetes and is implicated in AD (Selkoe, 1991; Leuner and Shors, 2004; Piperi et al., 2016; Remondelli and Renna, 2017). We identified an almost identical homology between the degu and humans *Xbp1* (Uniprot code: P17861), conserving the bZIP domain (residues 70 to 133) which suggests that the degu *Xbp1* has the same functions as the human isoform 1. The structure of this protein is unknown for any species. The INDEL we have identified in 80% of the analyzed degu shows that some degu only have the unspliced form of this protein and others the spliced form of this protein which affects the bZIP domain.

The *Octodon degus* naturally develops type-2 diabetes mellitus (T2DM), a known risk factor for AD (Zemva and Schubert, 2011). Based on previous work that insulin and Igf-1 influence Aβ toxicity we examined degu Igf-1(Westwood et al., 2014). For *Igf-1* we identified the homology between degu and human (XP_016874752.1) IGF1 to be 97.9 %, with only one amino acid substitution relative to human. Although more studies are needed to support these observations, our results indicate that it may be the case that some AD-like degu recapitulate some *Igf1* gene regulatory features seen in aging, insulin resistance and AD pathogenesis (Talbot et al., 2012).

Because in humans *APOE* alleles have a major impact on aging-associated diseases, particularly cardiovascular disease, type 2 diabetes and late-onset AD we focused on the effect of the *Apoe* variants we identified in the degu. The degu *Apoe* gene (Mt4) was significantly associated with an increase of Aβ deposition throughout cortical brain regions and correlated with cognitive impairment. Moreover, cell biology analysis suggested that the degu *Apoe* Mt4 was responsible, at least in part, for the excessive accumulation of lipid droplets, in degu fibroblasts and human-derived neurons as reported in *APOE4* human carriers (Liu et al., 2015; Wong et al., 2017). While altered lipid metabolism is reported here, the degu *Apoe* Mt4 variants may also contribute to AD-like phenotypes *via* other functions of *APOE* known to be relevant to AD pathogenesis, including brain glucose metabolism and the trafficking of Aβ, considering that this animal model has been reported as a natural model of atherosclerosis, diabetes and the neuropathogenesis of AD (Homan et al., 2010; Hurley et al., 2018).

Degu were divided according to their performance in activities of daily living (ADL) into two groups, AD-like and normal healthy (control) animals. ADL are defined as the things we normally do such as feeding ourselves, bathing and dressing. Deterioration in the ability to perform ADL is an early sign of cognitive decline. At present, learning and memory tests are widely used in the preclinical search for treatments for AD. However, in most cases these tests do not probe “episodic memory” which is the type most affected in AD. Healthy controls for this task are animals that complete the burrowing test in the expected time. AD-like are the animals that failed to complete the task in time. Burrowing performance also leave a number of intermediate burrowers, animals that in the time of the test, come out as not good or bad burrowers, but with an intermediate outcome. Interestingly, we have observed that with time, as they age, some intermediate individuals tend to become AD-like, although not all. Thus, in Figure 4C we compared the allele association with AD pathology and, an intermediate group of degu to analyse the impact of the *Apoe* variants on burrowing speed and variability in AD-like and intermediate degu. Interestingly, the amount of bedding displaced was significantly lower in the E213K group suggesting that this variant could have an impact on burrowing speed and variability.

The human E4 variant contains a single amino acid substitution that strongly influences the compactness and rigidity of APOE and consequent lipid binding capability, which is thought to involve the coordination of N- and C-terminal domains (Frieden et al., 2017). We predicted that the novel degu *Apoe* Mt4 mutation would affect interactions with lipids and/or other relevant macromolecules. The Mt4 forms of the degu *Apoe* change a crucial part of the hinge region immediately before the C-terminal domain, thereby making the hinge region more flexible, which allows conformational changes such as those occurring in *APOE4R* (with R at position 112) (Mizuguchi et al., 2014). Overall, our analysis of the dynamic properties of wild-type and the different the degu *Apoe* variants revealed how the E213Q and E213K polymorphisms strongly affect proper degu *Apoe* function. Similarities between the E213K and Q mutations and human ApoE4 are shown in Supplementary Figure 2.

Apoe3 only differs from Apoe4 by a single amino acid substitution, the human APOE gene has 90.0 % homology with the degu Apoe gene. Apoe4 > Apoe3 > Apoe2, lead to increased aggregation and deposition and decreased clearance of Aβ peptides. In humans APOE ε2 may provide some protection against Alzheimer's disease, but people with this allele can still develop the disease. APOE ε3, the most common allele plays a neutral role in the disease, people with this isoform can either have or not have the disease. While people with the APOE ε4 are most at risk of developing AD. It should be noted that the 213 polymorphism is a contributing factor to AD-like risk in the degu and is not exclusive or directly causative of AD-like pathology in all cases.

Lipid droplets are influenced by many variables. Nevertheless, the reasons for exploring lipid droplets in association with the *Apoe* mutation was based on recent publications. Astrocytes expressing E4 accumulate significantly more and smaller lipid droplets compared to E3 astrocytes (Farmer et al., 2019). Moreover, by different mechanisms the over accumulation of lipid droplets has been linked to atherosclerosis, diabetes (Krahmer et al., 2013) and AD (Alzheimer et al., 1995; Hamilton et al., 2015). Furthermore, recent findings suggest that glial cells within the brain form lipid droplets in the fly brain and this process is *Apoe* dependent (Liu et al., 2017; Claes et al., 2021; Yin, 2022). Pani et al. (2009) compared controls and AD patient skin fibroblasts and report elevated lipid droplets in AD fibroblasts.

The work we report here is observational, we do not attempt to identify a mechanism to link the presence of lipid droplets and *Apoe* in *Octodon degus*. We observed an increase of lipid droplets in degu cells carrying the *Apoe* variants. And when we inserted degu *Apoe* mutation by CRISPR into human fibroblasts, we observed a significant increase in lipid droplets. However, numerous genes in addition to *Apoe* are associated with lipid metabolism, transport, and lipid droplet formation and further study is necessary to determine whether there is a direct correlation between lipid droplet formation and the *Apoe* genotype in the degu.

The strengths of our study include the use of an outbred degu population that exhibits the hallmark features of the disease to create a genetic characterization of this model of late-onset AD. Importantly, we found a novel mutation that confers APOE4-like properties to the degu *Apoe* which was correlated with AD-like phenotypes, thereby identifying a genetic AD susceptibility in the degu. Nevertheless, there were limitations to this study. For instance, we did not address the relationship between the degu *Apoe* Mt4 to tau pathology or microglial activation, which are both major mediators of human AD pathogenesis, nor did we test for causal influences of microbiome changes associated with the degu *Apoe* Mt4and human APOE4. Future studies should be conducted to achieve these observations in degu. Moreover, a population study is necessary to understand the interplay between the degu *Apoe* Mt4 and environmental pressures in the degu population.

*In silico* modeling revealed that this variant imparted structural and functional changes in the APOE protein that resemble those seen in human *APOE4* carriers. *In vitro*, this variant caused an increase in lipid droplet formation. Remarkably, the degu *Apoe* Mt4variant was highly correlated with the accumulation of β-amyloid in the brain, alterations in neuronal lipid metabolism and a decline in behaviors analogous to activities of daily living in humans.

The whole-genome results suggest that, as in the human population, the *Apoe* gene is a risk factor for AD-like phenotype in a subpopulation of wild type lab-outbred (but not lab inbred) degu that spontaneously develop AD-like neuropathology. Some studies failed to report AD-like pathology in inbred populations of degu (Steffen et al., 2016). We can only speculate as to whether the inbred colonies that fail to show AD-like pathology also lack the K/Q 213 polymorphisms. However, in order to understand the degu as a model of AD correctly, we maintain our colony as a wild-outbred population because we have found some of these animals to spontaneously develop AD-like behaviors and pathology. The degu colonies in Europe and the USA were imported from Chile in the 1970's primarily to study circadian rhythm. Thus, these colonies have been maintained as inbred since then and have consequently lost genetic diversity. Moreover, when we performed a whole genome study of an inbred Chilean degu colony we identified 2.400.000 variants vs. 11.500.000 variants in our wild outbred colony. Clearly indicating the impact of inbreeding and providing the possible reason for the differences seen between animals from our outbred colony in Chile and inbred European colonies.

In summary, we have produced a genome resource of an unconventional animal model of AD, which is a particularly valuable tool for within family comparison of transgenic mouse AD pathways in terms of which AD gene regulatory pathways are common to spontaneous AD-like features in degu vs. different transgenic mouse models.

## Data Availability Statement

The datasets presented in this study can be found in online repositories. The names of the repository/repositories and accession number(s) can be found in the article/Supplementary Material.

## Ethics Statement

The animal study was reviewed and approved by Institute of Ecology and Biodiversity Ethics Committee, University of Chile, Santiago, Chile.

## Author Contributions

PC designed the study and the whole-genome sequencing and data analyses. PC, XX, and MC conceived the strategies. MH, CU, and FA analyzed the genome data. MH and PC wrote and edited the manuscript. XX designed the degu brain amyloid analysis. BG was involved in the amyloid immunohistochemistry experiments on degu brain sections. GC, AB, and CM-B contributed to the protein structure analysis of the degu APOE Mt4. ME and FE contributed to the lipid accumulation experiments on degu and human fibroblasts. MC contributed to the design of experiments involving human fibroblasts and CRISPR, as well as neuron-derived lipid droplet analysis and data interpretation. AK and BW performed the experimental work. PA and RD performed and designed the behavioral studies. EP, RV, TH, AA, PW-V, GZ, and CA worked on the manuscript and data analysis. All authors contributed to writing the final manuscript version and extensively to the work presented in this paper, have approved the submitted version and agreed to be personally accountable for their contributions, as well as to ensure that questions related to the accuracy and integrity of any part of the work are appropriately investigated, and resolved and that the resolution is documented in the literature.

## Funding

XX and PC were funded by US National Institutes of Health Grant R24AG073198. PC was funded by ANID-FONDECYT 1200928. RV and EP were funded by AFB-170008-CONICYT-Chile-IEB. BW and MC were funded by grant 3R01MH115005-02S1. AA was funded by ANID EX-CONICYT PAI77180086. GZ and CA are grateful for support from Research England's THYME project. TH is funded by R35 GM127102.

## Conflict of Interest

The authors declare that the research was conducted in the absence of any commercial or financial relationships that could be construed as a potential conflict of interest.

## Publisher's Note

All claims expressed in this article are solely those of the authors and do not necessarily represent those of their affiliated organizations, or those of the publisher, the editors and the reviewers. Any product that may be evaluated in this article, or claim that may be made by its manufacturer, is not guaranteed or endorsed by the publisher.

## Supplementary Material

Supplementary Figure 1 Electrostatic potential. **(A)** Wild-type odAPOE ribbon representation with transparent surface and 90-degree rotation to show the positions of the structures in B; E213 is represented as a red sphere. **(B)** Coulombic surfaces of wild type odAPOE, K odAPOE and Q odAPOE showing the reduction in electronegative surface potential in the two mutated models. Red indicates more electronegative, and blue indicates more electropositive.

Supplementary Figure 2 Domain structure of APOE3, APOE 4 and degu odAPOE Mt4. APOE3 and 4 proteins contain two independent domains that are connected by a hinge region. It has been proposed that Arg112 in APOE4 (instead of Cys 112 in APOE3 variant) exposes Arg61, which forms a salt bridge with Glu255 in the C-terminal domain and leads to close contact between the C- and N-terminal domains. The N-terminal domain contains the receptor-binding domain (indicated in green), and the C-terminal domain contains the lipid-binding region (indicated in orange). The odAPOE Mt4 at the E213 residue in the degu sequence is located at the end of the hinge region immediately before the start of the C-terminal domain at the end of the tube before the box representing the C-terminal domain in APOE4 (indicated in blue). The computational analysis suggested that the conformation induced by the mutation is more similar to that reported for APOE4 due to the formation of a salt bridge between Glu133 and Arg148, approaching the C-terminal and N-terminal domains. Similar to APOE4, this unique domain interaction in the degu odAPOE protein may result in lipidation and folding.

Supplementary Table 1 Variant calling metrics. Variants found in each *O. degus* genome compared to the *O. degus* reference genome (GCA_000260255.1). VQ filter represents the variant quality filter (Q > 100). DP filter represents the variant depth filter (DP > 20). InDel correspond to Insertions/Deletions.

Supplementary Table 2 *O. degus* genes containing missense variants. List of genes of *O. degus* that contain missense variants compared to the *O. degus* reference genome (GCA_000260255.1).

Supplementary Table 3 AD, DNA repair and diabetes related genes with missense variants. Extraction of variant information from vcf file of AD, DNA repair and diabetes related genes that contain missense variants in the evaluated animals compared to the *O. degus* reference genome (GCA_000260255.1).
