# Supplementary material for: Genome Sequencing Variations in the Octodon degus, an Unconventional Natural Model of Aging and Alzheimer's Disease
Source: Front Aging Neurosci. 2022 Jun 30;14:894994. doi: 10.3389/fnagi.2022.894994 (PMC9291219; doi:10.3389/fnagi.2022.894994)
Supplement: Supplementary Figure 1 — Electrostatic potential. (A) Wild-type odAPOE ribbon representation with transparent surface and 90-degree rotation to show the positions of the structures in B; E213 is represented as a red sphere. (B) Coulombic surfaces of wild type odAPOE, K odAPOE and Q odAPOE showing the reduction in electronegative surface potential in the two mutated models. Red indicates more electronegative, and blue indicates more electropositive. [file Table_1.DOCX]

**Genome sequencing variations in the *Octodon degus*, an unconventional natural model of aging and Alzheimer’s disease**

Michael J. Hurley^1,2^, Claudio Urra^2^, B. Maximiliano Garduno^3^, Agostino Bruno^4^, Allison Kimbell^5^, Brent Wilkinson^5^, Cristina Marino-Buslje^6^, Marcelo Ezquer^7^, Fernando Ezquer^7^, Pedro F. Aburto^2^, Elie Poulin^2^, Rodrigo A. Vasquez^2^, Robert Deacon^2^, Ariel Avila^8^, Francisco Altimiras^9^, Peter Whitney-Vanderklish^10^, Guido Zampieri^11^, Claudio Angione^11^, Gabriele Constantino^4†^, Todd C. Holmes^3†^, Marcelo P. Coba^5,12†^, Xiangmin Xu^3†^, Patricia Cogram^2,3†*^

**List of supplementary materials**


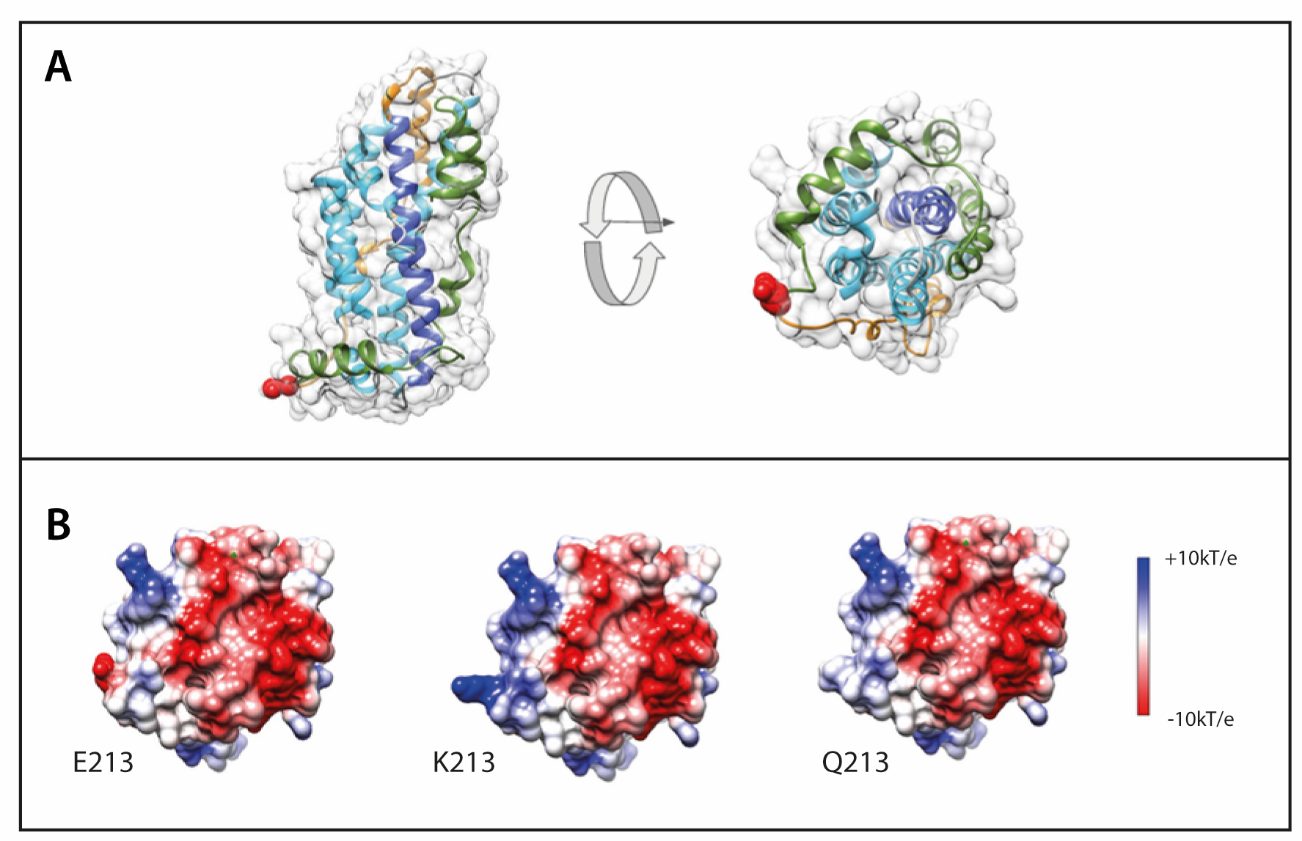


**Supplementary Figure S1. Electrostatic potential. a**) *WT-* odAPOE ribbon representation with transparent surface and 90 degree rotation to show E213 position in the structure(represented as a red sphere. **b**) Coulombic surfaces of *WT* odAPOE, K odAPOE and Q odAPOE showing the reduction in electronegative surface potential in the two mutated models. Red indicates more electronegative, and blue indicates more electropositive.

**Supplementary Figure S2.**


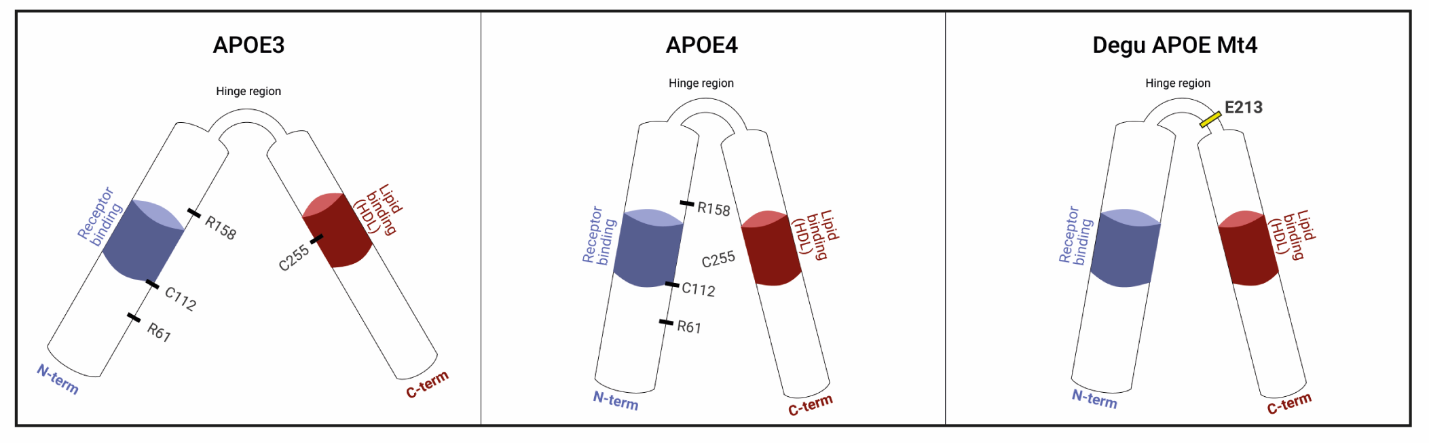


**Supplementary Figure S3. Domain structure of APOE3, APOE 4 and degu odAPOE Mt4 .** APOE3 and 4 proteins contain two independent domains that are connected by a hinge region. It has been proposed that Arg112 in APOE4 (instead of Cys 112 in APOE3 variant) exposes Arg61, which forms a salt bridge with Glu255 in the C-terminal domain and leads to close contact between the C- and N-terminal domains. The N-terminal domain contains the receptor-binding domain (indicated in green), and the C-terminal domain contains the lipid-binding region (indicated in orange). The odAPOE Mt4 at the E213 residue in the degu sequence is located at the end of the hinge region immediately before the start of the C-terminal domain at the end of the tube before the box representing the C-terminal domain in APOE4 (indicated in blue). The computational analysis suggested that the conformation induced by the mutation is more similar to that reported for APOE4 due to the formation of a salt bridge between Glu133 and Arg148, approaching the C-terminal and N-terminal domains. Similar to APOE4, this unique domain interaction in the degu odAPOE protein may result in lipidation and folding.
