# Supplementary material for: Genome Sequencing Variations in the Octodon degus, an Unconventional Natural Model of Aging and Alzheimer's Disease
Source: Front Aging Neurosci. 2022 Jun 30;14:894994. doi: 10.3389/fnagi.2022.894994 (PMC9291219; doi:10.3389/fnagi.2022.894994)
Supplement: Supplementary Table 1 — Variant calling metrics. Variants found in each O. degus genome compared to the O. degus reference genome (GCA_000260255.1). VQ filter represents the variant quality filter (Q > 100). DP filter represents the variant depth filter (DP > 20). InDel correspond to Insertions/Deletions. [file Data_Sheet_1.PDF]

Gene
ENSODEG00000000702
ENSODEG00000001428
ENSODEG00000001852
ENSODEG00000001936
ENSODEG00000002500
ENSODEG00000003063
ENSODEG00000004484
ENSODEG00000005206
ENSODEG00000006393
ENSODEG00000007177
ENSODEG00000007272
ENSODEG00000008474
ENSODEG00000009040
ENSODEG00000009478
ENSODEG00000010251
ENSODEG00000010616
ENSODEG00000010978
ENSODEG00000011346
ENSODEG00000011555
ENSODEG00000011558
ENSODEG00000011659
ENSODEG00000012032
ENSODEG00000012232
ENSODEG00000012354
ENSODEG00000013747
ENSODEG00000013885
ENSODEG00000014167
ENSODEG00000014372
ENSODEG00000014788
ENSODEG00000015386
ENSODEG00000018053
ENSODEG00000018308
ENSODEG00000000003
ENSODEG00000000004
ENSODEG00000000005
ENSODEG00000000006
ENSODEG00000000008
ENSODEG00000000009
ENSODEG00000000010
ENSODEG00000000011
ENSODEG00000000012
ENSODEG00000000013
ENSODEG00000000014
ENSODEG00000000015
ENSODEG00000000016
ENSODEG00000000017
ENSODEG00000000018
ENSODEG00000000020
ENSODEG00000000022
ENSODEG00000000023
ENSODEG00000000024
ENSODEG00000000025
ENSODEG00000000026
ENSODEG00000000028
#N/A
ENSODEG00000000029
ENSODEG00000000030
ENSODEG00000000032

ENSODEG00000000033
ENSODEG00000000034
ENSODEG00000000035
ENSODEG00000000036
ENSODEG00000000037
ENSODEG00000000038
ENSODEG00000000039
ENSODEG00000000040
ENSODEG00000000041
ENSODEG00000000042
ENSODEG00000000043
ENSODEG00000000044
ENSODEG00000000045
ENSODEG00000000046
ENSODEG00000000047
ENSODEG00000000048
ENSODEG00000000049
ENSODEG00000000050
ENSODEG00000000051
ENSODEG00000000052
ENSODEG00000000053
ENSODEG00000000055
ENSODEG00000000056
ENSODEG00000000058
ENSODEG00000000059
ENSODEG00000000062
ENSODEG00000000064
ENSODEG00000000065
ENSODEG00000000066
ENSODEG00000000067
ENSODEG00000000069
ENSODEG00000000071
ENSODEG00000000072
ENSODEG00000000073
ENSODEG00000000074
ENSODEG00000000075
ENSODEG00000000076
ENSODEG00000000077
ENSODEG00000000078
ENSODEG00000000079
ENSODEG00000000080
ENSODEG00000000081
ENSODEG00000000082
ENSODEG00000000085
ENSODEG00000000086
ENSODEG00000000087
ENSODEG00000000088
ENSODEG00000000091
ENSODEG00000000095
ENSODEG00000000096
ENSODEG00000000097
ENSODEG00000000099
ENSODEG00000000101
ENSODEG00000000102
ENSODEG00000000105
ENSODEG00000000108
ENSODEG00000000109
ENSODEG00000000110
ENSODEG00000000111

ENSODEG00000000112
ENSODEG00000000113
ENSODEG00000000115
ENSODEG00000000116
ENSODEG00000000117
ENSODEG00000000118
ENSODEG00000000119
ENSODEG00000000120
ENSODEG00000000121
ENSODEG00000000123
ENSODEG00000000124
ENSODEG00000000126
ENSODEG00000000130
ENSODEG00000000132
ENSODEG00000000133
ENSODEG00000000134
ENSODEG00000000136
ENSODEG00000000137
ENSODEG00000000138
ENSODEG00000000139
ENSODEG00000000140
ENSODEG00000000141
ENSODEG00000000143
ENSODEG00000000144
ENSODEG00000000145
ENSODEG00000000146
ENSODEG00000000147
ENSODEG00000000148
ENSODEG00000000149
ENSODEG00000000150
ENSODEG00000000151
ENSODEG00000000152
ENSODEG00000000154
ENSODEG00000000155
ENSODEG00000000156
ENSODEG00000000157
ENSODEG00000000159
ENSODEG00000000161
ENSODEG00000000164
ENSODEG00000000165
ENSODEG00000000166
ENSODEG00000000167
ENSODEG00000000168
ENSODEG00000000170
ENSODEG00000000171
ENSODEG00000000172
ENSODEG00000000173
ENSODEG00000000174
ENSODEG00000000175
ENSODEG00000000176
ENSODEG00000000177
ENSODEG00000000178
ENSODEG00000000180
ENSODEG00000000181
ENSODEG00000000184
ENSODEG00000000185
ENSODEG00000000186
ENSODEG00000000187
ENSODEG00000000189

ENSODEG00000000191
ENSODEG00000000192
ENSODEG00000000194
ENSODEG00000000195
ENSODEG00000000196
ENSODEG00000000200
ENSODEG00000000201
ENSODEG00000000202
ENSODEG00000000203
ENSODEG00000000204
ENSODEG00000000205
ENSODEG00000000206
ENSODEG00000000207
ENSODEG00000000208
ENSODEG00000000209
ENSODEG00000000212
ENSODEG00000000213
ENSODEG00000000215
ENSODEG00000000218
ENSODEG00000000219
ENSODEG00000000220
ENSODEG00000000221
ENSODEG00000000222
ENSODEG00000000223
ENSODEG00000000224
ENSODEG00000000225
ENSODEG00000000229
ENSODEG00000000232
ENSODEG00000000233
ENSODEG00000000235
ENSODEG00000000236
ENSODEG00000000237
ENSODEG00000000239
ENSODEG00000000241
ENSODEG00000000242
ENSODEG00000000244
ENSODEG00000000245
ENSODEG00000000246
ENSODEG00000000247
ENSODEG00000000248
ENSODEG00000000250
ENSODEG00000000251
ENSODEG00000000252
ENSODEG00000000253
ENSODEG00000000254
ENSODEG00000000255
ENSODEG00000000256
ENSODEG00000000257
ENSODEG00000000258
ENSODEG00000000259
ENSODEG00000000260
ENSODEG00000000261
ENSODEG00000000262
ENSODEG00000000263
ENSODEG00000000265
ENSODEG00000000267
ENSODEG00000000268
ENSODEG00000000271
ENSODEG00000000272

ENSODEG00000000273
ENSODEG00000000274
ENSODEG00000000275
ENSODEG00000000276
ENSODEG00000000277
ENSODEG00000000278
ENSODEG00000000279
ENSODEG00000000280
ENSODEG00000000281
ENSODEG00000000282
ENSODEG00000000283
ENSODEG00000000284
ENSODEG00000000285
ENSODEG00000000288
ENSODEG00000000289
ENSODEG00000000292
ENSODEG00000000293
ENSODEG00000000294
ENSODEG00000000295
ENSODEG00000000297
ENSODEG00000000298
ENSODEG00000000299
ENSODEG00000000301
ENSODEG00000000302
ENSODEG00000000303
ENSODEG00000000304
ENSODEG00000000306
ENSODEG00000000309
ENSODEG00000000310
ENSODEG00000000312
ENSODEG00000000313
ENSODEG00000000314
ENSODEG00000000315
ENSODEG00000000316
ENSODEG00000000317
ENSODEG00000000318
ENSODEG00000000320
ENSODEG00000000321
ENSODEG00000000322
ENSODEG00000000324
ENSODEG00000000327
ENSODEG00000000328
ENSODEG00000000331
ENSODEG00000000332
ENSODEG00000000333
ENSODEG00000000334
ENSODEG00000000336
ENSODEG00000000338
ENSODEG00000000339
ENSODEG00000000340
ENSODEG00000000341
ENSODEG00000000342
ENSODEG00000000343
ENSODEG00000000345
ENSODEG00000000348
ENSODEG00000000350
ENSODEG00000000351
ENSODEG00000000352
ENSODEG00000000353

ENSODEG00000000354
ENSODEG00000000355
ENSODEG00000000356
ENSODEG00000000357
ENSODEG00000000358
ENSODEG00000000359
ENSODEG00000000361
ENSODEG00000000362
ENSODEG00000000363
ENSODEG00000000364
ENSODEG00000000365
ENSODEG00000000366
ENSODEG00000000368
ENSODEG00000000369
ENSODEG00000000371
ENSODEG00000000372
ENSODEG00000000373
ENSODEG00000000374
ENSODEG00000000375
ENSODEG00000000376
ENSODEG00000000377
ENSODEG00000000378
ENSODEG00000000380
ENSODEG00000000381
ENSODEG00000000382
ENSODEG00000000383
ENSODEG00000000384
ENSODEG00000000385
ENSODEG00000000386
ENSODEG00000000387
ENSODEG00000000388
ENSODEG00000000390
ENSODEG00000000391
ENSODEG00000000393
ENSODEG00000000396
ENSODEG00000000397
ENSODEG00000000398
ENSODEG00000000399
ENSODEG00000000400
ENSODEG00000000401
ENSODEG00000000403
ENSODEG00000000404
ENSODEG00000000406
ENSODEG00000000411
ENSODEG00000000412
ENSODEG00000000413
ENSODEG00000000415
ENSODEG00000000416
ENSODEG00000000418
ENSODEG00000000419
ENSODEG00000000420
ENSODEG00000000421
ENSODEG00000000422
ENSODEG00000000423
ENSODEG00000000424
ENSODEG00000000425
ENSODEG00000000427
ENSODEG00000000428
ENSODEG00000000429

ENSODEG00000000430
ENSODEG00000000431
ENSODEG00000000432
ENSODEG00000000433
ENSODEG00000000434
ENSODEG00000000435
ENSODEG00000000436
ENSODEG00000000437
ENSODEG00000000438
ENSODEG00000000439
ENSODEG00000000440
ENSODEG00000000441
ENSODEG00000000442
ENSODEG00000000443
ENSODEG00000000444
ENSODEG00000000445
ENSODEG00000000446
ENSODEG00000000447
ENSODEG00000000448
ENSODEG00000000449
ENSODEG00000000450
ENSODEG00000000451
ENSODEG00000000452
ENSODEG00000000455
ENSODEG00000000456
ENSODEG00000000457
ENSODEG00000000458
ENSODEG00000000459
ENSODEG00000000460
ENSODEG00000000461
ENSODEG00000000462
ENSODEG00000000463
ENSODEG00000000464
ENSODEG00000000465
ENSODEG00000000466
ENSODEG00000000467
ENSODEG00000000468
ENSODEG00000000470
ENSODEG00000000471
ENSODEG00000000472
ENSODEG00000000473
ENSODEG00000000476
ENSODEG00000000477
ENSODEG00000000479
ENSODEG00000000481
ENSODEG00000000482
ENSODEG00000000485
ENSODEG00000000487
ENSODEG00000000488
ENSODEG00000000490
ENSODEG00000000491
ENSODEG00000000492
ENSODEG00000000493
ENSODEG00000000494
ENSODEG00000000495
ENSODEG00000000496
ENSODEG00000000499
ENSODEG00000000500
ENSODEG00000000501

ENSODEG00000000502
ENSODEG00000000503
ENSODEG00000000504
ENSODEG00000000505
ENSODEG00000000506
ENSODEG00000000507
ENSODEG00000000508
ENSODEG00000000510
ENSODEG00000000511
ENSODEG00000000513
ENSODEG00000000516
ENSODEG00000000517
ENSODEG00000000518
ENSODEG00000000519
ENSODEG00000000522
ENSODEG00000000523
ENSODEG00000000524
ENSODEG00000000526
ENSODEG00000000527
ENSODEG00000000528
ENSODEG00000000529
ENSODEG00000000530
ENSODEG00000000531
ENSODEG00000000533
ENSODEG00000000535
ENSODEG00000000536
ENSODEG00000000537
ENSODEG00000000538
ENSODEG00000000539
ENSODEG00000000540
ENSODEG00000000541
ENSODEG00000000543
ENSODEG00000000544
ENSODEG00000000545
ENSODEG00000000546
ENSODEG00000000548
ENSODEG00000000549
ENSODEG00000000550
ENSODEG00000000551
ENSODEG00000000552
ENSODEG00000000553
ENSODEG00000000554
ENSODEG00000000556
ENSODEG00000000557
ENSODEG00000000559
ENSODEG00000000561
ENSODEG00000000562
ENSODEG00000000564
ENSODEG00000000565
ENSODEG00000000566
ENSODEG00000000567
ENSODEG00000000568
ENSODEG00000000569
ENSODEG00000000572
ENSODEG00000000573
ENSODEG00000000574
ENSODEG00000000575
ENSODEG00000000576
ENSODEG00000000577

ENSODEG00000000578
ENSODEG00000000579
ENSODEG00000000580
ENSODEG00000000582
ENSODEG00000000583
ENSODEG00000000584
ENSODEG00000000587
ENSODEG00000000588
ENSODEG00000000590
ENSODEG00000000592
ENSODEG00000000593
ENSODEG00000000594
ENSODEG00000000596
ENSODEG00000000597
ENSODEG00000000598
ENSODEG00000000599
ENSODEG00000000600
ENSODEG00000000601
ENSODEG00000000602
ENSODEG00000000603
ENSODEG00000000607
ENSODEG00000000608
ENSODEG00000000609
ENSODEG00000000611
ENSODEG00000000612
ENSODEG00000000613
ENSODEG00000000614
ENSODEG00000000615
ENSODEG00000000616
ENSODEG00000000617
ENSODEG00000000618
ENSODEG00000000621
ENSODEG00000000625
ENSODEG00000000626
ENSODEG00000000627
ENSODEG00000000628
ENSODEG00000000629
ENSODEG00000000630
ENSODEG00000000631
ENSODEG00000000632
ENSODEG00000000633
ENSODEG00000000634
ENSODEG00000000635
ENSODEG00000000636
ENSODEG00000000637
ENSODEG00000000638
ENSODEG00000000639
ENSODEG00000000640
ENSODEG00000000641
ENSODEG00000000642
ENSODEG00000000643
ENSODEG00000000644
ENSODEG00000000645
ENSODEG00000000646
ENSODEG00000000647
ENSODEG00000000648
ENSODEG00000000649
ENSODEG00000000650
ENSODEG00000000651

ENSODEG00000000652
ENSODEG00000000653
ENSODEG00000000654
ENSODEG00000000655
ENSODEG00000000656
ENSODEG00000000657
ENSODEG00000000660
ENSODEG00000000661
ENSODEG00000000662
ENSODEG00000000663
ENSODEG00000000664
ENSODEG00000000665
ENSODEG00000000667
ENSODEG00000000668
ENSODEG00000000669
ENSODEG00000000671
ENSODEG00000000673
ENSODEG00000000674
ENSODEG00000000675
ENSODEG00000000676
ENSODEG00000000677
ENSODEG00000000679
ENSODEG00000000680
ENSODEG00000000681
ENSODEG00000000682
ENSODEG00000000684
ENSODEG00000000688
ENSODEG00000000689
ENSODEG00000000690
ENSODEG00000000691
ENSODEG00000000692
ENSODEG00000000693
ENSODEG00000000694
ENSODEG00000000695
ENSODEG00000000696
ENSODEG00000000697
ENSODEG00000000699
ENSODEG00000000700
ENSODEG00000000701
ENSODEG00000000703
ENSODEG00000000704
ENSODEG00000000708
ENSODEG00000000709
ENSODEG00000000710
ENSODEG00000000712
ENSODEG00000000713
ENSODEG00000000714
ENSODEG00000000715
ENSODEG00000000717
ENSODEG00000000718
ENSODEG00000000719
ENSODEG00000000720
ENSODEG00000000721
ENSODEG00000000722
ENSODEG00000000723
ENSODEG00000000724
ENSODEG00000000726
ENSODEG00000000727
ENSODEG00000000728

ENSODEG00000000729
ENSODEG00000000730
ENSODEG00000000731
ENSODEG00000000733
ENSODEG00000000734
ENSODEG00000000735
ENSODEG00000000737
ENSODEG00000000738
ENSODEG00000000739
ENSODEG00000000740
ENSODEG00000000742
ENSODEG00000000743
ENSODEG00000000744
ENSODEG00000000745
ENSODEG00000000746
ENSODEG00000000747
ENSODEG00000000748
ENSODEG00000000750
ENSODEG00000000751
ENSODEG00000000752
ENSODEG00000000753
ENSODEG00000000754
ENSODEG00000000756
ENSODEG00000000757
ENSODEG00000000758
ENSODEG00000000760
ENSODEG00000000761
ENSODEG00000000762
ENSODEG00000000763
ENSODEG00000000764
ENSODEG00000000765
ENSODEG00000000766
ENSODEG00000000768
ENSODEG00000000769
ENSODEG00000000770
ENSODEG00000000773
ENSODEG00000000776
ENSODEG00000000777
ENSODEG00000000778
ENSODEG00000000779
ENSODEG00000000780
ENSODEG00000000781
ENSODEG00000000782
ENSODEG00000000783
ENSODEG00000000785
ENSODEG00000000786
ENSODEG00000000790
ENSODEG00000000791
ENSODEG00000000792
ENSODEG00000000793
ENSODEG00000000794
ENSODEG00000000796
ENSODEG00000000797
ENSODEG00000000799
ENSODEG00000000801
ENSODEG00000000802
ENSODEG00000000803
ENSODEG00000000804
ENSODEG00000000807

ENSODEG00000000808
ENSODEG00000000809
ENSODEG00000000810
ENSODEG00000000811
ENSODEG00000000812
ENSODEG00000000814
ENSODEG00000000816
ENSODEG00000000817
ENSODEG00000000818
ENSODEG00000000820
ENSODEG00000000821
ENSODEG00000000823
ENSODEG00000000824
ENSODEG00000000825
ENSODEG00000000826
ENSODEG00000000827
ENSODEG00000000828
ENSODEG00000000830
ENSODEG00000000833
ENSODEG00000000834
ENSODEG00000000836
ENSODEG00000000837
ENSODEG00000000838
ENSODEG00000000840
ENSODEG00000000841
ENSODEG00000000842
ENSODEG00000000843
ENSODEG00000000844
ENSODEG00000000845
ENSODEG00000000846
ENSODEG00000000847
ENSODEG00000000848
ENSODEG00000000849
ENSODEG00000000850
ENSODEG00000000852
ENSODEG00000000853
ENSODEG00000000854
ENSODEG00000000856
ENSODEG00000000857
ENSODEG00000000859
ENSODEG00000000860
ENSODEG00000000862
ENSODEG00000000865
ENSODEG00000000866
ENSODEG00000000867
ENSODEG00000000868
ENSODEG00000000870
ENSODEG00000000871
ENSODEG00000000872
ENSODEG00000000873
ENSODEG00000000874
ENSODEG00000000875
ENSODEG00000000876
ENSODEG00000000877
ENSODEG00000000878
ENSODEG00000000879
ENSODEG00000000880
ENSODEG00000000881
ENSODEG00000000882

ENSODEG00000000885
ENSODEG00000000886
ENSODEG00000000889
ENSODEG00000000891
ENSODEG00000000892
ENSODEG00000000893
ENSODEG00000000894
ENSODEG00000000895
ENSODEG00000000899
ENSODEG00000000901
ENSODEG00000000902
ENSODEG00000000904
ENSODEG00000000905
ENSODEG00000000906
ENSODEG00000000907
ENSODEG00000000908
ENSODEG00000000909
ENSODEG00000000914
ENSODEG00000000915
ENSODEG00000000916
ENSODEG00000000917
ENSODEG00000000918
ENSODEG00000000919
ENSODEG00000000921
ENSODEG00000000923
ENSODEG00000000924
ENSODEG00000000925
ENSODEG00000000926
ENSODEG00000000927
ENSODEG00000000928
ENSODEG00000000930
ENSODEG00000000932
ENSODEG00000000933
ENSODEG00000000934
ENSODEG00000000935
ENSODEG00000000936
ENSODEG00000000937
ENSODEG00000000938
ENSODEG00000000940
ENSODEG00000000942
ENSODEG00000000943
ENSODEG00000000944
ENSODEG00000000945
ENSODEG00000000946
ENSODEG00000000947
ENSODEG00000000948
ENSODEG00000000949
ENSODEG00000000950
ENSODEG00000000951
ENSODEG00000000956
ENSODEG00000000957
ENSODEG00000000958
ENSODEG00000000959
ENSODEG00000000960
ENSODEG00000000962
ENSODEG00000000965
ENSODEG00000000966
ENSODEG00000000967
ENSODEG00000000968

ENSODEG00000000969
ENSODEG00000000970
ENSODEG00000000971
ENSODEG00000000972
ENSODEG00000000973
ENSODEG00000000977
ENSODEG00000000979
ENSODEG00000000980
ENSODEG00000000981
ENSODEG00000000982
ENSODEG00000000983
ENSODEG00000000985
ENSODEG00000000987
ENSODEG00000000988
ENSODEG00000000990
ENSODEG00000000991
ENSODEG00000000992
ENSODEG00000000993
ENSODEG00000000994
ENSODEG00000000995
ENSODEG00000000996
ENSODEG00000000997
ENSODEG00000000998
ENSODEG00000001000
ENSODEG00000001001
ENSODEG00000001002
ENSODEG00000001003
ENSODEG00000001004
ENSODEG00000001005
ENSODEG00000001006
ENSODEG00000001007
ENSODEG00000001008
ENSODEG00000001009
ENSODEG00000001010
ENSODEG00000001011
ENSODEG00000001013
ENSODEG00000001014
ENSODEG00000001015
ENSODEG00000001017
ENSODEG00000001019
ENSODEG00000001020
ENSODEG00000001022
ENSODEG00000001024
ENSODEG00000001025
ENSODEG00000001026
ENSODEG00000001027
ENSODEG00000001028
ENSODEG00000001029
ENSODEG00000001030
ENSODEG00000001031
ENSODEG00000001032
ENSODEG00000001033
ENSODEG00000001034
ENSODEG00000001035
ENSODEG00000001037
ENSODEG00000001038
ENSODEG00000001039
ENSODEG00000001041
ENSODEG00000001042

ENSODEG00000001046
ENSODEG00000001047
ENSODEG00000001049
ENSODEG00000001050
ENSODEG00000001051
ENSODEG00000001052
ENSODEG00000001054
ENSODEG00000001055
ENSODEG00000001056
ENSODEG00000001057
ENSODEG00000001060
ENSODEG00000001061
ENSODEG00000001062
ENSODEG00000001064
ENSODEG00000001066
ENSODEG00000001067
ENSODEG00000001068
ENSODEG00000001071
ENSODEG00000001073
ENSODEG00000001074
ENSODEG00000001075
ENSODEG00000001076
ENSODEG00000001077
ENSODEG00000001078
ENSODEG00000001079
ENSODEG00000001081
ENSODEG00000001082
ENSODEG00000001083
ENSODEG00000001084
ENSODEG00000001086
ENSODEG00000001087
ENSODEG00000001090
ENSODEG00000001093
ENSODEG00000001094
ENSODEG00000001095
ENSODEG00000001097
ENSODEG00000001098
ENSODEG00000001099
ENSODEG00000001100
ENSODEG00000001103
ENSODEG00000001104
ENSODEG00000001106
ENSODEG00000001107
ENSODEG00000001110
ENSODEG00000001111
ENSODEG00000001112
ENSODEG00000001113
ENSODEG00000001114
ENSODEG00000001115
ENSODEG00000001116
ENSODEG00000001117
ENSODEG00000001118
ENSODEG00000001122
ENSODEG00000001123
ENSODEG00000001125
ENSODEG00000001126
ENSODEG00000001127
ENSODEG00000001128
ENSODEG00000001129

ENSODEG00000001130
ENSODEG00000001132
ENSODEG00000001133
ENSODEG00000001137
ENSODEG00000001141
ENSODEG00000001143
ENSODEG00000001144
ENSODEG00000001145
ENSODEG00000001146
ENSODEG00000001150
ENSODEG00000001151
ENSODEG00000001152
ENSODEG00000001153
ENSODEG00000001154
ENSODEG00000001155
ENSODEG00000001156
ENSODEG00000001157
ENSODEG00000001159
ENSODEG00000001160
ENSODEG00000001161
ENSODEG00000001164
ENSODEG00000001165
ENSODEG00000001166
ENSODEG00000001167
ENSODEG00000001168
ENSODEG00000001169
ENSODEG00000001170
ENSODEG00000001172
ENSODEG00000001173
ENSODEG00000001174
ENSODEG00000001176
ENSODEG00000001177
ENSODEG00000001178
ENSODEG00000001179
ENSODEG00000001180
ENSODEG00000001181
ENSODEG00000001182
ENSODEG00000001183
ENSODEG00000001184
ENSODEG00000001186
ENSODEG00000001187
ENSODEG00000001188
ENSODEG00000001189
ENSODEG00000001190
ENSODEG00000001191
ENSODEG00000001192
ENSODEG00000001195
ENSODEG00000001196
ENSODEG00000001199
ENSODEG00000001200
ENSODEG00000001201
ENSODEG00000001203
ENSODEG00000001205
ENSODEG00000001206
ENSODEG00000001207
ENSODEG00000001208
ENSODEG00000001209
ENSODEG00000001212
ENSODEG00000001213

ENSODEG00000001216
ENSODEG00000001217
ENSODEG00000001218
ENSODEG00000001219
ENSODEG00000001220
ENSODEG00000001221
ENSODEG00000001222
ENSODEG00000001223
ENSODEG00000001224
ENSODEG00000001227
ENSODEG00000001228
ENSODEG00000001230
ENSODEG00000001231
ENSODEG00000001232
ENSODEG00000001234
ENSODEG00000001236
ENSODEG00000001239
ENSODEG00000001240
ENSODEG00000001243
ENSODEG00000001244
ENSODEG00000001245
ENSODEG00000001246
ENSODEG00000001247
ENSODEG00000001248
ENSODEG00000001254
ENSODEG00000001255
ENSODEG00000001256
ENSODEG00000001257
ENSODEG00000001258
ENSODEG00000001260
ENSODEG00000001261
ENSODEG00000001263
ENSODEG00000001264
ENSODEG00000001265
ENSODEG00000001266
ENSODEG00000001267
ENSODEG00000001268
ENSODEG00000001269
ENSODEG00000001270
ENSODEG00000001271
ENSODEG00000001273
ENSODEG00000001276
ENSODEG00000001278
ENSODEG00000001279
ENSODEG00000001280
ENSODEG00000001281
ENSODEG00000001283
ENSODEG00000001284
ENSODEG00000001285
ENSODEG00000001286
ENSODEG00000001288
ENSODEG00000001290
ENSODEG00000001291
ENSODEG00000001293
ENSODEG00000001294
ENSODEG00000001297
ENSODEG00000001298
ENSODEG00000001299
ENSODEG00000001300

ENSODEG00000001301
ENSODEG00000001302
ENSODEG00000001303
ENSODEG00000001304
ENSODEG00000001305
ENSODEG00000001306
ENSODEG00000001307
ENSODEG00000001308
ENSODEG00000001309
ENSODEG00000001310
ENSODEG00000001311
ENSODEG00000001312
ENSODEG00000001313
ENSODEG00000001314
ENSODEG00000001316
ENSODEG00000001317
ENSODEG00000001318
ENSODEG00000001319
ENSODEG00000001320
ENSODEG00000001321
ENSODEG00000001322
ENSODEG00000001323
ENSODEG00000001324
ENSODEG00000001325
ENSODEG00000001326
ENSODEG00000001328
ENSODEG00000001329
ENSODEG00000001330
ENSODEG00000001331
ENSODEG00000001332
ENSODEG00000001334
ENSODEG00000001335
ENSODEG00000001337
ENSODEG00000001338
ENSODEG00000001339
ENSODEG00000001340
ENSODEG00000001341
ENSODEG00000001342
ENSODEG00000001343
ENSODEG00000001344
ENSODEG00000001345
ENSODEG00000001346
ENSODEG00000001349
ENSODEG00000001350
ENSODEG00000001351
ENSODEG00000001352
ENSODEG00000001354
ENSODEG00000001355
ENSODEG00000001357
ENSODEG00000001359
ENSODEG00000001360
ENSODEG00000001363
ENSODEG00000001365
ENSODEG00000001366
ENSODEG00000001367
ENSODEG00000001368
ENSODEG00000001369
ENSODEG00000001370
ENSODEG00000001371

ENSODEG00000001372
ENSODEG00000001373
ENSODEG00000001375
ENSODEG00000001377
ENSODEG00000001379
ENSODEG00000001380
ENSODEG00000001381
ENSODEG00000001382
ENSODEG00000001383
ENSODEG00000001386
ENSODEG00000001387
ENSODEG00000001388
ENSODEG00000001391
ENSODEG00000001392
ENSODEG00000001394
ENSODEG00000001397
ENSODEG00000001398
ENSODEG00000001399
ENSODEG00000001400
ENSODEG00000001402
ENSODEG00000001403
ENSODEG00000001404
ENSODEG00000001407
ENSODEG00000001408
ENSODEG00000001412
ENSODEG00000001413
ENSODEG00000001414
ENSODEG00000001415
ENSODEG00000001417
ENSODEG00000001419
ENSODEG00000001420
ENSODEG00000001421
ENSODEG00000001425
ENSODEG00000001426
ENSODEG00000001430
ENSODEG00000001431
ENSODEG00000001432
ENSODEG00000001433
ENSODEG00000001434
ENSODEG00000001435
ENSODEG00000001437
ENSODEG00000001439
ENSODEG00000001440
ENSODEG00000001441
ENSODEG00000001442
ENSODEG00000001444
ENSODEG00000001445
ENSODEG00000001446
ENSODEG00000001447
ENSODEG00000001448
ENSODEG00000001449
ENSODEG00000001451
ENSODEG00000001452
ENSODEG00000001453
ENSODEG00000001454
ENSODEG00000001456
ENSODEG00000001457
ENSODEG00000001459
ENSODEG00000001464

ENSODEG00000001465
ENSODEG00000001468
ENSODEG00000001469
ENSODEG00000001472
ENSODEG00000001473
ENSODEG00000001474
ENSODEG00000001475
ENSODEG00000001477
ENSODEG00000001480
ENSODEG00000001481
ENSODEG00000001482
ENSODEG00000001483
ENSODEG00000001484
ENSODEG00000001485
ENSODEG00000001486
ENSODEG00000001487
ENSODEG00000001488
ENSODEG00000001489
ENSODEG00000001490
ENSODEG00000001491
ENSODEG00000001492
ENSODEG00000001499
ENSODEG00000001500
ENSODEG00000001502
ENSODEG00000001503
ENSODEG00000001504
ENSODEG00000001505
ENSODEG00000001506
ENSODEG00000001509
ENSODEG00000001510
ENSODEG00000001511
ENSODEG00000001512
ENSODEG00000001514
ENSODEG00000001515
ENSODEG00000001516
ENSODEG00000001517
ENSODEG00000001518
ENSODEG00000001519
ENSODEG00000001521
ENSODEG00000001522
ENSODEG00000001525
ENSODEG00000001526
ENSODEG00000001527
ENSODEG00000001530
ENSODEG00000001531
ENSODEG00000001532
ENSODEG00000001533
ENSODEG00000001534
ENSODEG00000001535
ENSODEG00000001536
ENSODEG00000001538
ENSODEG00000001539
ENSODEG00000001540
ENSODEG00000001541
ENSODEG00000001542
ENSODEG00000001543
ENSODEG00000001546
ENSODEG00000001549
ENSODEG00000001550

ENSODEG00000001551
ENSODEG00000001553
ENSODEG00000001554
ENSODEG00000001555
ENSODEG00000001556
ENSODEG00000001557
ENSODEG00000001558
ENSODEG00000001560
ENSODEG00000001561
ENSODEG00000001563
ENSODEG00000001564
ENSODEG00000001565
ENSODEG00000001567
ENSODEG00000001568
ENSODEG00000001569
ENSODEG00000001570
ENSODEG00000001572
ENSODEG00000001574
ENSODEG00000001575
ENSODEG00000001576
ENSODEG00000001577
ENSODEG00000001578
ENSODEG00000001579
ENSODEG00000001581
ENSODEG00000001582
ENSODEG00000001584
ENSODEG00000001585
ENSODEG00000001586
ENSODEG00000001588
ENSODEG00000001589
ENSODEG00000001591
ENSODEG00000001592
ENSODEG00000001593
ENSODEG00000001595
ENSODEG00000001597
ENSODEG00000001598
ENSODEG00000001599
ENSODEG00000001601
ENSODEG00000001602
ENSODEG00000001603
ENSODEG00000001604
ENSODEG00000001607
ENSODEG00000001609
ENSODEG00000001610
ENSODEG00000001612
ENSODEG00000001613
ENSODEG00000001615
ENSODEG00000001617
ENSODEG00000001618
ENSODEG00000001619
ENSODEG00000001620
ENSODEG00000001622
ENSODEG00000001623
ENSODEG00000001624
ENSODEG00000001625
ENSODEG00000001626
ENSODEG00000001628
ENSODEG00000001629
ENSODEG00000001630

ENSODEG00000001631
ENSODEG00000001632
ENSODEG00000001633
ENSODEG00000001635
ENSODEG00000001636
ENSODEG00000001637
ENSODEG00000001638
ENSODEG00000001639
ENSODEG00000001640
ENSODEG00000001642
ENSODEG00000001643
ENSODEG00000001644
ENSODEG00000001645
ENSODEG00000001646
ENSODEG00000001647
ENSODEG00000001649
ENSODEG00000001650
ENSODEG00000001651
ENSODEG00000001655
ENSODEG00000001656
ENSODEG00000001657
ENSODEG00000001658
ENSODEG00000001660
ENSODEG00000001661
ENSODEG00000001662
ENSODEG00000001663
ENSODEG00000001664
ENSODEG00000001665
ENSODEG00000001668
ENSODEG00000001670
ENSODEG00000001671
ENSODEG00000001672
ENSODEG00000001675
ENSODEG00000001676
ENSODEG00000001677
ENSODEG00000001678
ENSODEG00000001679
ENSODEG00000001680
ENSODEG00000001683
ENSODEG00000001685
ENSODEG00000001686
ENSODEG00000001687
ENSODEG00000001688
ENSODEG00000001689
ENSODEG00000001690
ENSODEG00000001693
ENSODEG00000001694
ENSODEG00000001695
ENSODEG00000001696
ENSODEG00000001697
ENSODEG00000001698
ENSODEG00000001699
ENSODEG00000001700
ENSODEG00000001701
ENSODEG00000001702
ENSODEG00000001703
ENSODEG00000001704
ENSODEG00000001707
ENSODEG00000001710

ENSODEG00000001711
ENSODEG00000001716
ENSODEG00000001717
ENSODEG00000001719
ENSODEG00000001720
ENSODEG00000001721
ENSODEG00000001723
ENSODEG00000001724
ENSODEG00000001725
ENSODEG00000001726
ENSODEG00000001727
ENSODEG00000001728
ENSODEG00000001730
ENSODEG00000001732
ENSODEG00000001733
ENSODEG00000001735
ENSODEG00000001736
ENSODEG00000001741
ENSODEG00000001742
ENSODEG00000001743
ENSODEG00000001745
ENSODEG00000001746
ENSODEG00000001747
ENSODEG00000001748
ENSODEG00000001749
ENSODEG00000001750
ENSODEG00000001751
ENSODEG00000001752
ENSODEG00000001753
ENSODEG00000001754
ENSODEG00000001755
ENSODEG00000001757
ENSODEG00000001759
ENSODEG00000001760
ENSODEG00000001761
ENSODEG00000001762
ENSODEG00000001763
ENSODEG00000001765
ENSODEG00000001766
ENSODEG00000001767
ENSODEG00000001768
ENSODEG00000001769
ENSODEG00000001770
ENSODEG00000001771
ENSODEG00000001772
ENSODEG00000001773
ENSODEG00000001774
ENSODEG00000001775
ENSODEG00000001776
ENSODEG00000001777
ENSODEG00000001779
ENSODEG00000001780
ENSODEG00000001781
ENSODEG00000001782
ENSODEG00000001783
ENSODEG00000001784
ENSODEG00000001785
ENSODEG00000001786
ENSODEG00000001787

ENSODEG00000001788
ENSODEG00000001789
ENSODEG00000001790
ENSODEG00000001791
ENSODEG00000001792
ENSODEG00000001794
ENSODEG00000001795
ENSODEG00000001796
ENSODEG00000001797
ENSODEG00000001798
ENSODEG00000001799
ENSODEG00000001800
ENSODEG00000001802
ENSODEG00000001803
ENSODEG00000001804
ENSODEG00000001805
ENSODEG00000001806
ENSODEG00000001807
ENSODEG00000001810
ENSODEG00000001811
ENSODEG00000001812
ENSODEG00000001813
ENSODEG00000001816
ENSODEG00000001819
ENSODEG00000001821
ENSODEG00000001822
ENSODEG00000001823
ENSODEG00000001824
ENSODEG00000001825
ENSODEG00000001826
ENSODEG00000001832
ENSODEG00000001833
ENSODEG00000001835
ENSODEG00000001837
ENSODEG00000001838
ENSODEG00000001839
ENSODEG00000001840
ENSODEG00000001841
ENSODEG00000001842
ENSODEG00000001843
ENSODEG00000001844
ENSODEG00000001845
ENSODEG00000001846
ENSODEG00000001847
ENSODEG00000001848
ENSODEG00000001849
ENSODEG00000001850
ENSODEG00000001853
ENSODEG00000001854
ENSODEG00000001855
ENSODEG00000001856
ENSODEG00000001857
ENSODEG00000001858
ENSODEG00000001859
ENSODEG00000001861
ENSODEG00000001864
ENSODEG00000001865
ENSODEG00000001866
ENSODEG00000001868

ENSODEG00000001869
ENSODEG00000001871
ENSODEG00000001873
ENSODEG00000001874
ENSODEG00000001876
ENSODEG00000001877
ENSODEG00000001878
ENSODEG00000001879
ENSODEG00000001881
ENSODEG00000001882
ENSODEG00000001884
ENSODEG00000001885
ENSODEG00000001886
ENSODEG00000001887
ENSODEG00000001888
ENSODEG00000001889
ENSODEG00000001890
ENSODEG00000001894
ENSODEG00000001895
ENSODEG00000001897
ENSODEG00000001898
ENSODEG00000001900
ENSODEG00000001902
ENSODEG00000001903
ENSODEG00000001904
ENSODEG00000001906
ENSODEG00000001907
ENSODEG00000001909
ENSODEG00000001911
ENSODEG00000001912
ENSODEG00000001913
ENSODEG00000001914
ENSODEG00000001915
ENSODEG00000001916
ENSODEG00000001917
ENSODEG00000001918
ENSODEG00000001919
ENSODEG00000001920
ENSODEG00000001921
ENSODEG00000001922
ENSODEG00000001923
ENSODEG00000001926
ENSODEG00000001927
ENSODEG00000001928
ENSODEG00000001930
ENSODEG00000001931
ENSODEG00000001932
ENSODEG00000001933
ENSODEG00000001935
ENSODEG00000001938
ENSODEG00000001940
ENSODEG00000001941
ENSODEG00000001942
ENSODEG00000001943
ENSODEG00000001944
ENSODEG00000001945
ENSODEG00000001947
ENSODEG00000001951
ENSODEG00000001952

ENSODEG00000001953
ENSODEG00000001954
ENSODEG00000001955
ENSODEG00000001959
ENSODEG00000001962
ENSODEG00000001966
ENSODEG00000001967
ENSODEG00000001968
ENSODEG00000001969
ENSODEG00000001970
ENSODEG00000001971
ENSODEG00000001972
ENSODEG00000001973
ENSODEG00000001976
ENSODEG00000001977
ENSODEG00000001978
ENSODEG00000001980
ENSODEG00000001981
ENSODEG00000001982
ENSODEG00000001984
ENSODEG00000001985
ENSODEG00000001986
ENSODEG00000001987
ENSODEG00000001988
ENSODEG00000001990
ENSODEG00000001991
ENSODEG00000001994
ENSODEG00000001995
ENSODEG00000001996
ENSODEG00000001999
ENSODEG00000002000
ENSODEG00000002001
ENSODEG00000002002
ENSODEG00000002003
ENSODEG00000002006
ENSODEG00000002008
ENSODEG00000002010
ENSODEG00000002012
ENSODEG00000002015
ENSODEG00000002018
ENSODEG00000002019
ENSODEG00000002021
ENSODEG00000002022
ENSODEG00000002023
ENSODEG00000002024
ENSODEG00000002025
ENSODEG00000002026
ENSODEG00000002027
ENSODEG00000002028
ENSODEG00000002029
ENSODEG00000002030
ENSODEG00000002031
ENSODEG00000002032
ENSODEG00000002033
ENSODEG00000002035
ENSODEG00000002037
ENSODEG00000002038
ENSODEG00000002039
ENSODEG00000002040

ENSODEG00000002041
ENSODEG00000002042
ENSODEG00000002043
ENSODEG00000002046
ENSODEG00000002047
ENSODEG00000002048
ENSODEG00000002050
ENSODEG00000002051
ENSODEG00000002052
ENSODEG00000002053
ENSODEG00000002054
ENSODEG00000002056
ENSODEG00000002058
ENSODEG00000002059
ENSODEG00000002060
ENSODEG00000002061
ENSODEG00000002062
ENSODEG00000002064
ENSODEG00000002066
ENSODEG00000002068
ENSODEG00000002070
ENSODEG00000002071
ENSODEG00000002073
ENSODEG00000002075
ENSODEG00000002076
ENSODEG00000002077
ENSODEG00000002078
ENSODEG00000002080
ENSODEG00000002081
ENSODEG00000002082
ENSODEG00000002084
ENSODEG00000002085
ENSODEG00000002086
ENSODEG00000002088
ENSODEG00000002089
ENSODEG00000002091
ENSODEG00000002093
ENSODEG00000002094
ENSODEG00000002095
ENSODEG00000002096
ENSODEG00000002097
ENSODEG00000002098
ENSODEG00000002100
ENSODEG00000002101
ENSODEG00000002104
ENSODEG00000002105
ENSODEG00000002106
ENSODEG00000002107
ENSODEG00000002108
ENSODEG00000002109
ENSODEG00000002110
ENSODEG00000002111
ENSODEG00000002113
ENSODEG00000002115
ENSODEG00000002116
ENSODEG00000002117
ENSODEG00000002118
ENSODEG00000002121
ENSODEG00000002124

ENSODEG00000002125
ENSODEG00000002126
ENSODEG00000002128
ENSODEG00000002129
ENSODEG00000002130
ENSODEG00000002131
ENSODEG00000002132
ENSODEG00000002133
ENSODEG00000002134
ENSODEG00000002135
ENSODEG00000002137
ENSODEG00000002138
ENSODEG00000002139
ENSODEG00000002142
ENSODEG00000002143
ENSODEG00000002144
ENSODEG00000002145
ENSODEG00000002147
ENSODEG00000002148
ENSODEG00000002149
ENSODEG00000002150
ENSODEG00000002151
ENSODEG00000002152
ENSODEG00000002153
ENSODEG00000002154
ENSODEG00000002155
ENSODEG00000002156
ENSODEG00000002157
ENSODEG00000002158
ENSODEG00000002160
ENSODEG00000002161
ENSODEG00000002162
ENSODEG00000002163
ENSODEG00000002164
ENSODEG00000002166
ENSODEG00000002167
ENSODEG00000002168
ENSODEG00000002169
ENSODEG00000002170
ENSODEG00000002173
ENSODEG00000002175
ENSODEG00000002176
ENSODEG00000002177
ENSODEG00000002178
ENSODEG00000002179
ENSODEG00000002180
ENSODEG00000002182
ENSODEG00000002184
ENSODEG00000002185
ENSODEG00000002186
ENSODEG00000002187
ENSODEG00000002189
ENSODEG00000002190
ENSODEG00000002191
ENSODEG00000002192
ENSODEG00000002193
ENSODEG00000002195
ENSODEG00000002196
ENSODEG00000002197

ENSODEG00000002198
ENSODEG00000002200
ENSODEG00000002201
ENSODEG00000002202
ENSODEG00000002203
ENSODEG00000002205
ENSODEG00000002206
ENSODEG00000002208
ENSODEG00000002209
ENSODEG00000002210
ENSODEG00000002211
ENSODEG00000002212
ENSODEG00000002213
ENSODEG00000002214
ENSODEG00000002215
ENSODEG00000002216
ENSODEG00000002217
ENSODEG00000002218
ENSODEG00000002219
ENSODEG00000002220
ENSODEG00000002221
ENSODEG00000002222
ENSODEG00000002223
ENSODEG00000002224
ENSODEG00000002225
ENSODEG00000002226
ENSODEG00000002227
ENSODEG00000002231
ENSODEG00000002232
ENSODEG00000002235
ENSODEG00000002236
ENSODEG00000002237
ENSODEG00000002238
ENSODEG00000002239
ENSODEG00000002240
ENSODEG00000002241
ENSODEG00000002242
ENSODEG00000002244
ENSODEG00000002246
ENSODEG00000002249
ENSODEG00000002250
ENSODEG00000002251
ENSODEG00000002255
ENSODEG00000002256
ENSODEG00000002260
ENSODEG00000002263
ENSODEG00000002264
ENSODEG00000002265
ENSODEG00000002266
ENSODEG00000002268
ENSODEG00000002269
ENSODEG00000002270
ENSODEG00000002272
ENSODEG00000002273
ENSODEG00000002275
ENSODEG00000002276
ENSODEG00000002277
ENSODEG00000002278
ENSODEG00000002279

ENSODEG00000002281
ENSODEG00000002282
ENSODEG00000002283
ENSODEG00000002284
ENSODEG00000002286
ENSODEG00000002289
ENSODEG00000002290
ENSODEG00000002291
ENSODEG00000002293
ENSODEG00000002297
ENSODEG00000002298
ENSODEG00000002299
ENSODEG00000002301
ENSODEG00000002303
ENSODEG00000002304
ENSODEG00000002306
ENSODEG00000002307
ENSODEG00000002309
ENSODEG00000002310
ENSODEG00000002311
ENSODEG00000002312
ENSODEG00000002313
ENSODEG00000002314
ENSODEG00000002317
ENSODEG00000002320
ENSODEG00000002321
ENSODEG00000002323
ENSODEG00000002327
ENSODEG00000002328
ENSODEG00000002330
ENSODEG00000002331
ENSODEG00000002332
ENSODEG00000002333
ENSODEG00000002334
ENSODEG00000002335
ENSODEG00000002336
ENSODEG00000002338
ENSODEG00000002341
ENSODEG00000002342
ENSODEG00000002343
ENSODEG00000002344
ENSODEG00000002346
ENSODEG00000002347
ENSODEG00000002348
ENSODEG00000002349
ENSODEG00000002350
ENSODEG00000002351
ENSODEG00000002352
ENSODEG00000002354
ENSODEG00000002355
ENSODEG00000002356
ENSODEG00000002357
ENSODEG00000002358
ENSODEG00000002359
ENSODEG00000002360
ENSODEG00000002361
ENSODEG00000002362
ENSODEG00000002363
ENSODEG00000002365

ENSODEG00000002366
ENSODEG00000002367
ENSODEG00000002368
ENSODEG00000002369
ENSODEG00000002371
ENSODEG00000002373
ENSODEG00000002374
ENSODEG00000002375
ENSODEG00000002376
ENSODEG00000002377
ENSODEG00000002378
ENSODEG00000002379
ENSODEG00000002382
ENSODEG00000002384
ENSODEG00000002386
ENSODEG00000002389
ENSODEG00000002392
ENSODEG00000002393
ENSODEG00000002394
ENSODEG00000002395
ENSODEG00000002398
ENSODEG00000002400
ENSODEG00000002402
ENSODEG00000002404
ENSODEG00000002405
ENSODEG00000002406
ENSODEG00000002407
ENSODEG00000002408
ENSODEG00000002409
ENSODEG00000002410
ENSODEG00000002411
ENSODEG00000002412
ENSODEG00000002413
ENSODEG00000002416
ENSODEG00000002417
ENSODEG00000002421
ENSODEG00000002422
ENSODEG00000002423
ENSODEG00000002425
ENSODEG00000002426
ENSODEG00000002427
ENSODEG00000002429
ENSODEG00000002430
ENSODEG00000002431
ENSODEG00000002432
ENSODEG00000002433
ENSODEG00000002434
ENSODEG00000002435
ENSODEG00000002436
ENSODEG00000002437
ENSODEG00000002438
ENSODEG00000002439
ENSODEG00000002441
ENSODEG00000002442
ENSODEG00000002444
ENSODEG00000002445
ENSODEG00000002446
ENSODEG00000002447
ENSODEG00000002448

ENSODEG00000002450
ENSODEG00000002451
ENSODEG00000002452
ENSODEG00000002454
ENSODEG00000002456
ENSODEG00000002457
ENSODEG00000002458
ENSODEG00000002460
ENSODEG00000002461
ENSODEG00000002462
ENSODEG00000002463
ENSODEG00000002466
ENSODEG00000002468
ENSODEG00000002469
ENSODEG00000002470
ENSODEG00000002471
ENSODEG00000002472
ENSODEG00000002473
ENSODEG00000002474
ENSODEG00000002475
ENSODEG00000002477
ENSODEG00000002478
ENSODEG00000002479
ENSODEG00000002480
ENSODEG00000002482
ENSODEG00000002483
ENSODEG00000002484
ENSODEG00000002485
ENSODEG00000002486
ENSODEG00000002487
ENSODEG00000002488
ENSODEG00000002489
ENSODEG00000002492
ENSODEG00000002493
ENSODEG00000002494
ENSODEG00000002495
ENSODEG00000002497
ENSODEG00000002498
ENSODEG00000002499
ENSODEG00000002503
ENSODEG00000002506
ENSODEG00000002508
ENSODEG00000002509
ENSODEG00000002511
ENSODEG00000002512
ENSODEG00000002513
ENSODEG00000002514
ENSODEG00000002515
ENSODEG00000002516
ENSODEG00000002517
ENSODEG00000002518
ENSODEG00000002520
ENSODEG00000002521
ENSODEG00000002522
ENSODEG00000002524
ENSODEG00000002525
ENSODEG00000002527
ENSODEG00000002529
ENSODEG00000002530

ENSODEG00000002532
ENSODEG00000002534
ENSODEG00000002536
ENSODEG00000002537
ENSODEG00000002538
ENSODEG00000002539
ENSODEG00000002541
ENSODEG00000002543
ENSODEG00000002544
ENSODEG00000002545
ENSODEG00000002546
ENSODEG00000002547
ENSODEG00000002549
ENSODEG00000002550
ENSODEG00000002551
ENSODEG00000002552
ENSODEG00000002555
ENSODEG00000002557
ENSODEG00000002561
ENSODEG00000002562
ENSODEG00000002564
ENSODEG00000002565
ENSODEG00000002566
ENSODEG00000002567
ENSODEG00000002568
ENSODEG00000002569
ENSODEG00000002572
ENSODEG00000002574
ENSODEG00000002576
ENSODEG00000002577
ENSODEG00000002579
ENSODEG00000002580
ENSODEG00000002582
ENSODEG00000002583
ENSODEG00000002584
ENSODEG00000002586
ENSODEG00000002587
ENSODEG00000002588
ENSODEG00000002590
ENSODEG00000002594
ENSODEG00000002596
ENSODEG00000002598
ENSODEG00000002599
ENSODEG00000002601
ENSODEG00000002602
ENSODEG00000002603
ENSODEG00000002604
ENSODEG00000002605
ENSODEG00000002606
ENSODEG00000002607
ENSODEG00000002608
ENSODEG00000002609
ENSODEG00000002610
ENSODEG00000002611
ENSODEG00000002613
ENSODEG00000002614
ENSODEG00000002615
ENSODEG00000002617
ENSODEG00000002618

ENSODEG00000002619
ENSODEG00000002621
ENSODEG00000002622
ENSODEG00000002623
ENSODEG00000002625
ENSODEG00000002626
ENSODEG00000002627
ENSODEG00000002629
ENSODEG00000002630
ENSODEG00000002632
ENSODEG00000002633
ENSODEG00000002634
ENSODEG00000002636
ENSODEG00000002637
ENSODEG00000002638
ENSODEG00000002639
ENSODEG00000002641
ENSODEG00000002643
ENSODEG00000002646
ENSODEG00000002647
ENSODEG00000002648
ENSODEG00000002649
ENSODEG00000002650
ENSODEG00000002652
ENSODEG00000002653
ENSODEG00000002654
ENSODEG00000002655
ENSODEG00000002657
ENSODEG00000002658
ENSODEG00000002662
ENSODEG00000002664
ENSODEG00000002665
ENSODEG00000002666
ENSODEG00000002667
ENSODEG00000002668
ENSODEG00000002669
ENSODEG00000002671
ENSODEG00000002673
ENSODEG00000002674
ENSODEG00000002675
ENSODEG00000002676
ENSODEG00000002677
ENSODEG00000002678
ENSODEG00000002680
ENSODEG00000002681
ENSODEG00000002684
ENSODEG00000002685
ENSODEG00000002686
ENSODEG00000002687
ENSODEG00000002688
ENSODEG00000002689
ENSODEG00000002690
ENSODEG00000002692
ENSODEG00000002694
ENSODEG00000002695
ENSODEG00000002696
ENSODEG00000002698
ENSODEG00000002700
ENSODEG00000002701

ENSODEG00000002702
ENSODEG00000002704
ENSODEG00000002705
ENSODEG00000002706
ENSODEG00000002709
ENSODEG00000002710
ENSODEG00000002712
ENSODEG00000002713
ENSODEG00000002714
ENSODEG00000002716
ENSODEG00000002717
ENSODEG00000002718
ENSODEG00000002720
ENSODEG00000002721
ENSODEG00000002722
ENSODEG00000002723
ENSODEG00000002724
ENSODEG00000002727
ENSODEG00000002728
ENSODEG00000002729
ENSODEG00000002730
ENSODEG00000002731
ENSODEG00000002733
ENSODEG00000002734
ENSODEG00000002735
ENSODEG00000002738
ENSODEG00000002739
ENSODEG00000002740
ENSODEG00000002743
ENSODEG00000002744
ENSODEG00000002745
ENSODEG00000002747
ENSODEG00000002748
ENSODEG00000002749
ENSODEG00000002750
ENSODEG00000002751
ENSODEG00000002752
ENSODEG00000002753
ENSODEG00000002754
ENSODEG00000002755
ENSODEG00000002756
ENSODEG00000002757
ENSODEG00000002758
ENSODEG00000002759
ENSODEG00000002760
ENSODEG00000002761
ENSODEG00000002762
ENSODEG00000002763
ENSODEG00000002764
ENSODEG00000002765
ENSODEG00000002766
ENSODEG00000002767
ENSODEG00000002768
ENSODEG00000002772
ENSODEG00000002773
ENSODEG00000002777
ENSODEG00000002779
ENSODEG00000002781
ENSODEG00000002782

ENSODEG00000002784
ENSODEG00000002785
ENSODEG00000002787
ENSODEG00000002789
ENSODEG00000002790
ENSODEG00000002791
ENSODEG00000002792
ENSODEG00000002793
ENSODEG00000002794
ENSODEG00000002796
ENSODEG00000002798
ENSODEG00000002799
ENSODEG00000002801
ENSODEG00000002802
ENSODEG00000002803
ENSODEG00000002804
ENSODEG00000002805
ENSODEG00000002809
ENSODEG00000002810
ENSODEG00000002811
ENSODEG00000002812
ENSODEG00000002814
ENSODEG00000002815
ENSODEG00000002816
ENSODEG00000002817
ENSODEG00000002818
ENSODEG00000002819
ENSODEG00000002820
ENSODEG00000002821
ENSODEG00000002824
ENSODEG00000002825
ENSODEG00000002826
ENSODEG00000002827
ENSODEG00000002828
ENSODEG00000002831
ENSODEG00000002832
ENSODEG00000002833
ENSODEG00000002835
ENSODEG00000002836
ENSODEG00000002837
ENSODEG00000002838
ENSODEG00000002839
ENSODEG00000002841
ENSODEG00000002842
ENSODEG00000002843
ENSODEG00000002844
ENSODEG00000002845
ENSODEG00000002846
ENSODEG00000002847
ENSODEG00000002849
ENSODEG00000002850
ENSODEG00000002851
ENSODEG00000002852
ENSODEG00000002853
ENSODEG00000002854
ENSODEG00000002855
ENSODEG00000002856
ENSODEG00000002857
ENSODEG00000002858

ENSODEG00000002860
ENSODEG00000002861
ENSODEG00000002862
ENSODEG00000002864
ENSODEG00000002866
ENSODEG00000002867
ENSODEG00000002868
ENSODEG00000002870
ENSODEG00000002871
ENSODEG00000002872
ENSODEG00000002873
ENSODEG00000002874
ENSODEG00000002875
ENSODEG00000002876
ENSODEG00000002877
ENSODEG00000002878
ENSODEG00000002879
ENSODEG00000002880
ENSODEG00000002881
ENSODEG00000002882
ENSODEG00000002883
ENSODEG00000002884
ENSODEG00000002886
ENSODEG00000002887
ENSODEG00000002889
ENSODEG00000002892
ENSODEG00000002893
ENSODEG00000002898
ENSODEG00000002899
ENSODEG00000002900
ENSODEG00000002902
ENSODEG00000002903
ENSODEG00000002904
ENSODEG00000002905
ENSODEG00000002906
ENSODEG00000002907
ENSODEG00000002908
ENSODEG00000002909
ENSODEG00000002912
ENSODEG00000002913
ENSODEG00000002915
ENSODEG00000002917
ENSODEG00000002918
ENSODEG00000002920
ENSODEG00000002921
ENSODEG00000002922
ENSODEG00000002923
ENSODEG00000002924
ENSODEG00000002925
ENSODEG00000002927
ENSODEG00000002928
ENSODEG00000002929
ENSODEG00000002930
ENSODEG00000002931
ENSODEG00000002932
ENSODEG00000002934
ENSODEG00000002935
ENSODEG00000002936
ENSODEG00000002939

ENSODEG00000002941
ENSODEG00000002942
ENSODEG00000002943
ENSODEG00000002944
ENSODEG00000002945
ENSODEG00000002947
ENSODEG00000002948
ENSODEG00000002949
ENSODEG00000002950
ENSODEG00000002952
ENSODEG00000002953
ENSODEG00000002954
ENSODEG00000002955
ENSODEG00000002956
ENSODEG00000002958
ENSODEG00000002960
ENSODEG00000002962
ENSODEG00000002963
ENSODEG00000002964
ENSODEG00000002965
ENSODEG00000002968
ENSODEG00000002969
ENSODEG00000002971
ENSODEG00000002972
ENSODEG00000002973
ENSODEG00000002974
ENSODEG00000002975
ENSODEG00000002978
ENSODEG00000002979
ENSODEG00000002983
ENSODEG00000002984
ENSODEG00000002985
ENSODEG00000002988
ENSODEG00000002989
ENSODEG00000002990
ENSODEG00000002991
ENSODEG00000002992
ENSODEG00000002993
ENSODEG00000002994
ENSODEG00000002995
ENSODEG00000002996
ENSODEG00000003000
ENSODEG00000003002
ENSODEG00000003003
ENSODEG00000003004
ENSODEG00000003005
ENSODEG00000003008
ENSODEG00000003009
ENSODEG00000003010
ENSODEG00000003011
ENSODEG00000003012
ENSODEG00000003013
ENSODEG00000003014
ENSODEG00000003015
ENSODEG00000003016
ENSODEG00000003017
ENSODEG00000003018
ENSODEG00000003019
ENSODEG00000003021

ENSODEG00000003022
ENSODEG00000003023
ENSODEG00000003026
ENSODEG00000003027
ENSODEG00000003028
ENSODEG00000003029
ENSODEG00000003030
ENSODEG00000003032
ENSODEG00000003033
ENSODEG00000003035
ENSODEG00000003038
ENSODEG00000003039
ENSODEG00000003040
ENSODEG00000003041
ENSODEG00000003042
ENSODEG00000003043
ENSODEG00000003044
ENSODEG00000003045
ENSODEG00000003046
ENSODEG00000003048
ENSODEG00000003049
ENSODEG00000003050
ENSODEG00000003051
ENSODEG00000003052
ENSODEG00000003053
ENSODEG00000003054
ENSODEG00000003055
ENSODEG00000003056
ENSODEG00000003057
ENSODEG00000003058
ENSODEG00000003059
ENSODEG00000003060
ENSODEG00000003061
ENSODEG00000003062
ENSODEG00000003064
ENSODEG00000003065
ENSODEG00000003066
ENSODEG00000003067
ENSODEG00000003068
ENSODEG00000003070
ENSODEG00000003071
ENSODEG00000003072
ENSODEG00000003073
ENSODEG00000003074
ENSODEG00000003075
ENSODEG00000003076
ENSODEG00000003077
ENSODEG00000003078
ENSODEG00000003079
ENSODEG00000003080
ENSODEG00000003081
ENSODEG00000003083
ENSODEG00000003084
ENSODEG00000003085
ENSODEG00000003086
ENSODEG00000003087
ENSODEG00000003088
ENSODEG00000003090
ENSODEG00000003092

ENSODEG00000003093
ENSODEG00000003094
ENSODEG00000003096
ENSODEG00000003097
ENSODEG00000003098
ENSODEG00000003099
ENSODEG00000003100
ENSODEG00000003102
ENSODEG00000003104
ENSODEG00000003105
ENSODEG00000003106
ENSODEG00000003108
ENSODEG00000003110
ENSODEG00000003111
ENSODEG00000003112
ENSODEG00000003113
ENSODEG00000003114
ENSODEG00000003117
ENSODEG00000003118
ENSODEG00000003119
ENSODEG00000003121
ENSODEG00000003122
ENSODEG00000003123
ENSODEG00000003124
ENSODEG00000003125
ENSODEG00000003126
ENSODEG00000003130
ENSODEG00000003131
ENSODEG00000003132
ENSODEG00000003133
ENSODEG00000003134
ENSODEG00000003135
ENSODEG00000003136
ENSODEG00000003137
ENSODEG00000003139
ENSODEG00000003140
ENSODEG00000003141
ENSODEG00000003142
ENSODEG00000003143
ENSODEG00000003144
ENSODEG00000003145
ENSODEG00000003146
ENSODEG00000003148
ENSODEG00000003149
ENSODEG00000003150
ENSODEG00000003151
ENSODEG00000003152
ENSODEG00000003153
ENSODEG00000003154
ENSODEG00000003155
ENSODEG00000003156
ENSODEG00000003157
ENSODEG00000003158
ENSODEG00000003159
ENSODEG00000003161
ENSODEG00000003163
ENSODEG00000003164
ENSODEG00000003167
ENSODEG00000003168

ENSODEG00000003169
ENSODEG00000003171
ENSODEG00000003172
ENSODEG00000003175
ENSODEG00000003177
ENSODEG00000003179
ENSODEG00000003180
ENSODEG00000003182
ENSODEG00000003183
ENSODEG00000003185
ENSODEG00000003187
ENSODEG00000003189
ENSODEG00000003192
ENSODEG00000003193
ENSODEG00000003198
ENSODEG00000003200
ENSODEG00000003202
ENSODEG00000003204
ENSODEG00000003208
ENSODEG00000003209
ENSODEG00000003211
ENSODEG00000003212
ENSODEG00000003213
ENSODEG00000003215
ENSODEG00000003216
ENSODEG00000003217
ENSODEG00000003218
ENSODEG00000003219
ENSODEG00000003220
ENSODEG00000003223
ENSODEG00000003225
ENSODEG00000003226
ENSODEG00000003227
ENSODEG00000003228
ENSODEG00000003229
ENSODEG00000003230
ENSODEG00000003231
ENSODEG00000003233
ENSODEG00000003234
ENSODEG00000003235
ENSODEG00000003238
ENSODEG00000003239
ENSODEG00000003240
ENSODEG00000003241
ENSODEG00000003242
ENSODEG00000003243
ENSODEG00000003244
ENSODEG00000003245
ENSODEG00000003246
ENSODEG00000003248
ENSODEG00000003249
ENSODEG00000003253
ENSODEG00000003254
ENSODEG00000003256
ENSODEG00000003257
ENSODEG00000003258
ENSODEG00000003260
ENSODEG00000003261
ENSODEG00000003262

ENSODEG00000003263
ENSODEG00000003264
ENSODEG00000003266
ENSODEG00000003267
ENSODEG00000003269
ENSODEG00000003270
ENSODEG00000003271
ENSODEG00000003272
ENSODEG00000003273
ENSODEG00000003274
ENSODEG00000003278
ENSODEG00000003279
ENSODEG00000003281
ENSODEG00000003282
ENSODEG00000003283
ENSODEG00000003284
ENSODEG00000003285
ENSODEG00000003288
ENSODEG00000003290
ENSODEG00000003292
ENSODEG00000003293
ENSODEG00000003295
ENSODEG00000003299
ENSODEG00000003300
ENSODEG00000003302
ENSODEG00000003303
ENSODEG00000003304
ENSODEG00000003306
ENSODEG00000003307
ENSODEG00000003310
ENSODEG00000003313
ENSODEG00000003314
ENSODEG00000003315
ENSODEG00000003316
ENSODEG00000003317
ENSODEG00000003319
ENSODEG00000003320
ENSODEG00000003321
ENSODEG00000003322
ENSODEG00000003323
ENSODEG00000003324
ENSODEG00000003325
ENSODEG00000003327
ENSODEG00000003328
ENSODEG00000003330
ENSODEG00000003332
ENSODEG00000003335
ENSODEG00000003336
ENSODEG00000003337
ENSODEG00000003341
ENSODEG00000003342
ENSODEG00000003343
ENSODEG00000003344
ENSODEG00000003345
ENSODEG00000003346
ENSODEG00000003347
ENSODEG00000003348
ENSODEG00000003351
ENSODEG00000003352

ENSODEG00000003353
ENSODEG00000003358
ENSODEG00000003359
ENSODEG00000003360
ENSODEG00000003361
ENSODEG00000003362
ENSODEG00000003364
ENSODEG00000003365
ENSODEG00000003367
ENSODEG00000003368
ENSODEG00000003369
ENSODEG00000003370
ENSODEG00000003371
ENSODEG00000003372
ENSODEG00000003374
ENSODEG00000003375
ENSODEG00000003376
ENSODEG00000003377
ENSODEG00000003379
ENSODEG00000003380
ENSODEG00000003381
ENSODEG00000003382
ENSODEG00000003383
ENSODEG00000003385
ENSODEG00000003386
ENSODEG00000003387
ENSODEG00000003389
ENSODEG00000003390
ENSODEG00000003391
ENSODEG00000003392
ENSODEG00000003393
ENSODEG00000003394
ENSODEG00000003397
ENSODEG00000003398
ENSODEG00000003399
ENSODEG00000003400
ENSODEG00000003401
ENSODEG00000003403
ENSODEG00000003404
ENSODEG00000003405
ENSODEG00000003406
ENSODEG00000003407
ENSODEG00000003408
ENSODEG00000003410
ENSODEG00000003411
ENSODEG00000003412
ENSODEG00000003413
ENSODEG00000003415
ENSODEG00000003416
ENSODEG00000003419
ENSODEG00000003420
ENSODEG00000003422
ENSODEG00000003423
ENSODEG00000003427
ENSODEG00000003428
ENSODEG00000003429
ENSODEG00000003430
ENSODEG00000003431
ENSODEG00000003432

ENSODEG00000003433
ENSODEG00000003435
ENSODEG00000003436
ENSODEG00000003439
ENSODEG00000003440
ENSODEG00000003441
ENSODEG00000003442
ENSODEG00000003443
ENSODEG00000003445
ENSODEG00000003446
ENSODEG00000003447
ENSODEG00000003448
ENSODEG00000003450
ENSODEG00000003452
ENSODEG00000003453
ENSODEG00000003454
ENSODEG00000003457
ENSODEG00000003458
ENSODEG00000003459
ENSODEG00000003460
ENSODEG00000003461
ENSODEG00000003462
ENSODEG00000003463
ENSODEG00000003464
ENSODEG00000003465
ENSODEG00000003468
ENSODEG00000003469
ENSODEG00000003470
ENSODEG00000003473
ENSODEG00000003474
ENSODEG00000003475
ENSODEG00000003476
ENSODEG00000003477
ENSODEG00000003478
ENSODEG00000003479
ENSODEG00000003480
ENSODEG00000003481
ENSODEG00000003482
ENSODEG00000003483
ENSODEG00000003484
ENSODEG00000003485
ENSODEG00000003486
ENSODEG00000003487
ENSODEG00000003488
ENSODEG00000003489
ENSODEG00000003491
ENSODEG00000003493
ENSODEG00000003495
ENSODEG00000003496
ENSODEG00000003498
ENSODEG00000003499
ENSODEG00000003500
ENSODEG00000003502
ENSODEG00000003504
ENSODEG00000003505
ENSODEG00000003506
ENSODEG00000003507
ENSODEG00000003508
ENSODEG00000003509

ENSODEG00000003511
ENSODEG00000003514
ENSODEG00000003515
ENSODEG00000003517
ENSODEG00000003518
ENSODEG00000003519
ENSODEG00000003520
ENSODEG00000003521
ENSODEG00000003522
ENSODEG00000003523
ENSODEG00000003526
ENSODEG00000003527
ENSODEG00000003528
ENSODEG00000003529
ENSODEG00000003530
ENSODEG00000003531
ENSODEG00000003532
ENSODEG00000003534
ENSODEG00000003535
ENSODEG00000003536
ENSODEG00000003537
ENSODEG00000003539
ENSODEG00000003540
ENSODEG00000003541
ENSODEG00000003542
ENSODEG00000003544
ENSODEG00000003545
ENSODEG00000003547
ENSODEG00000003548
ENSODEG00000003551
ENSODEG00000003552
ENSODEG00000003553
ENSODEG00000003554
ENSODEG00000003556
ENSODEG00000003558
ENSODEG00000003559
ENSODEG00000003561
ENSODEG00000003562
ENSODEG00000003563
ENSODEG00000003564
ENSODEG00000003567
ENSODEG00000003568
ENSODEG00000003569
ENSODEG00000003574
ENSODEG00000003576
ENSODEG00000003577
ENSODEG00000003578
ENSODEG00000003579
ENSODEG00000003580
ENSODEG00000003581
ENSODEG00000003582
ENSODEG00000003583
ENSODEG00000003584
ENSODEG00000003585
ENSODEG00000003586
ENSODEG00000003587
ENSODEG00000003589
ENSODEG00000003591
ENSODEG00000003594

ENSODEG00000003597
ENSODEG00000003598
ENSODEG00000003599
ENSODEG00000003600
ENSODEG00000003601
ENSODEG00000003602
ENSODEG00000003605
ENSODEG00000003606
ENSODEG00000003607
ENSODEG00000003608
ENSODEG00000003611
ENSODEG00000003613
ENSODEG00000003614
ENSODEG00000003615
ENSODEG00000003616
ENSODEG00000003619
ENSODEG00000003620
ENSODEG00000003621
ENSODEG00000003623
ENSODEG00000003624
ENSODEG00000003625
ENSODEG00000003626
ENSODEG00000003627
ENSODEG00000003632
ENSODEG00000003635
ENSODEG00000003636
ENSODEG00000003637
ENSODEG00000003638
ENSODEG00000003639
ENSODEG00000003640
ENSODEG00000003641
ENSODEG00000003642
ENSODEG00000003643
ENSODEG00000003644
ENSODEG00000003645
ENSODEG00000003646
ENSODEG00000003647
ENSODEG00000003648
ENSODEG00000003649
ENSODEG00000003650
ENSODEG00000003652
ENSODEG00000003653
ENSODEG00000003655
ENSODEG00000003656
ENSODEG00000003657
ENSODEG00000003658
ENSODEG00000003659
ENSODEG00000003660
ENSODEG00000003661
ENSODEG00000003663
ENSODEG00000003664
ENSODEG00000003665
ENSODEG00000003666
ENSODEG00000003667
ENSODEG00000003668
ENSODEG00000003669
ENSODEG00000003670
ENSODEG00000003672
ENSODEG00000003675

ENSODEG00000003676
ENSODEG00000003678
ENSODEG00000003679
ENSODEG00000003680
ENSODEG00000003681
ENSODEG00000003683
ENSODEG00000003685
ENSODEG00000003686
ENSODEG00000003687
ENSODEG00000003689
ENSODEG00000003690
ENSODEG00000003691
ENSODEG00000003692
ENSODEG00000003693
ENSODEG00000003694
ENSODEG00000003695
ENSODEG00000003696
ENSODEG00000003697
ENSODEG00000003699
ENSODEG00000003700
ENSODEG00000003701
ENSODEG00000003702
ENSODEG00000003703
ENSODEG00000003704
ENSODEG00000003705
ENSODEG00000003706
ENSODEG00000003707
ENSODEG00000003708
ENSODEG00000003709
ENSODEG00000003710
ENSODEG00000003712
ENSODEG00000003713
ENSODEG00000003714
ENSODEG00000003717
ENSODEG00000003720
ENSODEG00000003721
ENSODEG00000003723
ENSODEG00000003724
ENSODEG00000003725
ENSODEG00000003726
ENSODEG00000003727
ENSODEG00000003728
ENSODEG00000003729
ENSODEG00000003730
ENSODEG00000003731
ENSODEG00000003732
ENSODEG00000003733
ENSODEG00000003734
ENSODEG00000003736
ENSODEG00000003738
ENSODEG00000003739
ENSODEG00000003740
ENSODEG00000003742
ENSODEG00000003743
ENSODEG00000003744
ENSODEG00000003745
ENSODEG00000003746
ENSODEG00000003747
ENSODEG00000003749

ENSODEG00000003750
ENSODEG00000003751
ENSODEG00000003752
ENSODEG00000003754
ENSODEG00000003755
ENSODEG00000003756
ENSODEG00000003759
ENSODEG00000003762
ENSODEG00000003763
ENSODEG00000003764
ENSODEG00000003765
ENSODEG00000003766
ENSODEG00000003767
ENSODEG00000003769
ENSODEG00000003771
ENSODEG00000003772
ENSODEG00000003773
ENSODEG00000003774
ENSODEG00000003775
ENSODEG00000003776
ENSODEG00000003778
ENSODEG00000003779
ENSODEG00000003780
ENSODEG00000003781
ENSODEG00000003782
ENSODEG00000003786
ENSODEG00000003788
ENSODEG00000003789
ENSODEG00000003790
ENSODEG00000003791
ENSODEG00000003793
ENSODEG00000003794
ENSODEG00000003795
ENSODEG00000003797
ENSODEG00000003798
ENSODEG00000003799
ENSODEG00000003800
ENSODEG00000003801
ENSODEG00000003803
ENSODEG00000003805
ENSODEG00000003807
ENSODEG00000003809
ENSODEG00000003810
ENSODEG00000003811
ENSODEG00000003812
ENSODEG00000003813
ENSODEG00000003814
ENSODEG00000003815
ENSODEG00000003816
ENSODEG00000003817
ENSODEG00000003818
ENSODEG00000003819
ENSODEG00000003821
ENSODEG00000003823
ENSODEG00000003824
ENSODEG00000003825
ENSODEG00000003828
ENSODEG00000003829
ENSODEG00000003832

ENSODEG00000003834
ENSODEG00000003836
ENSODEG00000003837
ENSODEG00000003838
ENSODEG00000003839
ENSODEG00000003841
ENSODEG00000003842
ENSODEG00000003843
ENSODEG00000003844
ENSODEG00000003845
ENSODEG00000003846
ENSODEG00000003847
ENSODEG00000003848
ENSODEG00000003849
ENSODEG00000003850
ENSODEG00000003851
ENSODEG00000003853
ENSODEG00000003854
ENSODEG00000003857
ENSODEG00000003859
ENSODEG00000003861
ENSODEG00000003862
ENSODEG00000003864
ENSODEG00000003865
ENSODEG00000003866
ENSODEG00000003867
ENSODEG00000003868
ENSODEG00000003870
ENSODEG00000003871
ENSODEG00000003875
ENSODEG00000003878
ENSODEG00000003882
ENSODEG00000003883
ENSODEG00000003884
ENSODEG00000003885
ENSODEG00000003893
ENSODEG00000003895
ENSODEG00000003896
ENSODEG00000003897
ENSODEG00000003899
ENSODEG00000003900
ENSODEG00000003901
ENSODEG00000003903
ENSODEG00000003904
ENSODEG00000003905
ENSODEG00000003906
ENSODEG00000003909
ENSODEG00000003910
ENSODEG00000003912
ENSODEG00000003913
ENSODEG00000003914
ENSODEG00000003915
ENSODEG00000003916
ENSODEG00000003917
ENSODEG00000003918
ENSODEG00000003920
ENSODEG00000003921
ENSODEG00000003922
ENSODEG00000003923

ENSODEG00000003924
ENSODEG00000003925
ENSODEG00000003927
ENSODEG00000003928
ENSODEG00000003931
ENSODEG00000003932
ENSODEG00000003933
ENSODEG00000003934
ENSODEG00000003937
ENSODEG00000003938
ENSODEG00000003939
ENSODEG00000003940
ENSODEG00000003941
ENSODEG00000003946
ENSODEG00000003947
ENSODEG00000003948
ENSODEG00000003950
ENSODEG00000003951
ENSODEG00000003952
ENSODEG00000003953
ENSODEG00000003954
ENSODEG00000003957
ENSODEG00000003958
ENSODEG00000003959
ENSODEG00000003964
ENSODEG00000003966
ENSODEG00000003969
ENSODEG00000003970
ENSODEG00000003971
ENSODEG00000003972
ENSODEG00000003973
ENSODEG00000003974
ENSODEG00000003976
ENSODEG00000003977
ENSODEG00000003978
ENSODEG00000003979
ENSODEG00000003980
ENSODEG00000003983
ENSODEG00000003984
ENSODEG00000003985
ENSODEG00000003986
ENSODEG00000003987
ENSODEG00000003989
ENSODEG00000003991
ENSODEG00000003992
ENSODEG00000003993
ENSODEG00000003994
ENSODEG00000003995
ENSODEG00000003996
ENSODEG00000003997
ENSODEG00000003998
ENSODEG00000003999
ENSODEG00000004000
ENSODEG00000004001
ENSODEG00000004003
ENSODEG00000004004
ENSODEG00000004005
ENSODEG00000004008
ENSODEG00000004010

ENSODEG00000004011
ENSODEG00000004012
ENSODEG00000004013
ENSODEG00000004015
ENSODEG00000004016
ENSODEG00000004019
ENSODEG00000004021
ENSODEG00000004022
ENSODEG00000004023
ENSODEG00000004024
ENSODEG00000004025
ENSODEG00000004027
ENSODEG00000004029
ENSODEG00000004032
ENSODEG00000004033
ENSODEG00000004036
ENSODEG00000004037
ENSODEG00000004039
ENSODEG00000004040
ENSODEG00000004041
ENSODEG00000004042
ENSODEG00000004044
ENSODEG00000004045
ENSODEG00000004047
ENSODEG00000004048
ENSODEG00000004049
ENSODEG00000004050
ENSODEG00000004051
ENSODEG00000004052
ENSODEG00000004053
ENSODEG00000004055
ENSODEG00000004056
ENSODEG00000004057
ENSODEG00000004058
ENSODEG00000004061
ENSODEG00000004063
ENSODEG00000004065
ENSODEG00000004066
ENSODEG00000004068
ENSODEG00000004069
ENSODEG00000004070
ENSODEG00000004074
ENSODEG00000004076
ENSODEG00000004077
ENSODEG00000004079
ENSODEG00000004080
ENSODEG00000004081
ENSODEG00000004083
ENSODEG00000004084
ENSODEG00000004085
ENSODEG00000004087
ENSODEG00000004088
ENSODEG00000004089
ENSODEG00000004090
ENSODEG00000004092
ENSODEG00000004093
ENSODEG00000004094
ENSODEG00000004095
ENSODEG00000004096

ENSODEG00000004097
ENSODEG00000004098
ENSODEG00000004099
ENSODEG00000004100
ENSODEG00000004102
ENSODEG00000004103
ENSODEG00000004105
ENSODEG00000004106
ENSODEG00000004107
ENSODEG00000004108
ENSODEG00000004109
ENSODEG00000004110
ENSODEG00000004111
ENSODEG00000004112
ENSODEG00000004113
ENSODEG00000004114
ENSODEG00000004116
ENSODEG00000004117
ENSODEG00000004118
ENSODEG00000004121
ENSODEG00000004123
ENSODEG00000004124
ENSODEG00000004125
ENSODEG00000004127
ENSODEG00000004128
ENSODEG00000004130
ENSODEG00000004131
ENSODEG00000004133
ENSODEG00000004134
ENSODEG00000004138
ENSODEG00000004139
ENSODEG00000004140
ENSODEG00000004141
ENSODEG00000004142
ENSODEG00000004143
ENSODEG00000004146
ENSODEG00000004147
ENSODEG00000004148
ENSODEG00000004152
ENSODEG00000004153
ENSODEG00000004154
ENSODEG00000004155
ENSODEG00000004156
ENSODEG00000004158
ENSODEG00000004163
ENSODEG00000004164
ENSODEG00000004166
ENSODEG00000004167
ENSODEG00000004169
ENSODEG00000004170
ENSODEG00000004171
ENSODEG00000004172
ENSODEG00000004173
ENSODEG00000004177
ENSODEG00000004178
ENSODEG00000004179
ENSODEG00000004180
ENSODEG00000004181
ENSODEG00000004182

ENSODEG00000004183
ENSODEG00000004184
ENSODEG00000004185
ENSODEG00000004186
ENSODEG00000004187
ENSODEG00000004189
ENSODEG00000004190
ENSODEG00000004191
ENSODEG00000004192
ENSODEG00000004193
ENSODEG00000004194
ENSODEG00000004195
ENSODEG00000004196
ENSODEG00000004197
ENSODEG00000004198
ENSODEG00000004199
ENSODEG00000004200
ENSODEG00000004202
ENSODEG00000004203
ENSODEG00000004204
ENSODEG00000004205
ENSODEG00000004206
ENSODEG00000004207
ENSODEG00000004208
ENSODEG00000004209
ENSODEG00000004211
ENSODEG00000004213
ENSODEG00000004214
ENSODEG00000004215
ENSODEG00000004216
ENSODEG00000004217
ENSODEG00000004218
ENSODEG00000004219
ENSODEG00000004220
ENSODEG00000004223
ENSODEG00000004226
ENSODEG00000004227
ENSODEG00000004229
ENSODEG00000004230
ENSODEG00000004232
ENSODEG00000004233
ENSODEG00000004234
ENSODEG00000004235
ENSODEG00000004236
ENSODEG00000004237
ENSODEG00000004238
ENSODEG00000004239
ENSODEG00000004240
ENSODEG00000004241
ENSODEG00000004242
ENSODEG00000004246
ENSODEG00000004248
ENSODEG00000004249
ENSODEG00000004250
ENSODEG00000004251
ENSODEG00000004252
ENSODEG00000004253
ENSODEG00000004254
ENSODEG00000004256

ENSODEG00000004257
ENSODEG00000004258
ENSODEG00000004259
ENSODEG00000004260
ENSODEG00000004261
ENSODEG00000004262
ENSODEG00000004263
ENSODEG00000004264
ENSODEG00000004265
ENSODEG00000004266
ENSODEG00000004268
ENSODEG00000004270
ENSODEG00000004272
ENSODEG00000004273
ENSODEG00000004274
ENSODEG00000004275
ENSODEG00000004276
ENSODEG00000004277
ENSODEG00000004279
ENSODEG00000004280
ENSODEG00000004281
ENSODEG00000004282
ENSODEG00000004283
ENSODEG00000004285
ENSODEG00000004288
ENSODEG00000004290
ENSODEG00000004291
ENSODEG00000004292
ENSODEG00000004293
ENSODEG00000004294
ENSODEG00000004295
ENSODEG00000004296
ENSODEG00000004297
ENSODEG00000004299
ENSODEG00000004300
ENSODEG00000004301
ENSODEG00000004303
ENSODEG00000004304
ENSODEG00000004305
ENSODEG00000004307
ENSODEG00000004309
ENSODEG00000004310
ENSODEG00000004311
ENSODEG00000004312
ENSODEG00000004313
ENSODEG00000004314
ENSODEG00000004316
ENSODEG00000004318
ENSODEG00000004321
ENSODEG00000004323
ENSODEG00000004326
ENSODEG00000004327
ENSODEG00000004328
ENSODEG00000004330
ENSODEG00000004332
ENSODEG00000004333
ENSODEG00000004334
ENSODEG00000004335
ENSODEG00000004336

ENSODEG00000004337
ENSODEG00000004338
ENSODEG00000004339
ENSODEG00000004340
ENSODEG00000004342
ENSODEG00000004344
ENSODEG00000004345
ENSODEG00000004347
ENSODEG00000004348
ENSODEG00000004350
ENSODEG00000004351
ENSODEG00000004353
ENSODEG00000004354
ENSODEG00000004355
ENSODEG00000004356
ENSODEG00000004357
ENSODEG00000004360
ENSODEG00000004361
ENSODEG00000004362
ENSODEG00000004363
ENSODEG00000004364
ENSODEG00000004365
ENSODEG00000004366
ENSODEG00000004367
ENSODEG00000004368
ENSODEG00000004369
ENSODEG00000004370
ENSODEG00000004372
ENSODEG00000004373
ENSODEG00000004374
ENSODEG00000004376
ENSODEG00000004377
ENSODEG00000004378
ENSODEG00000004379
ENSODEG00000004380
ENSODEG00000004382
ENSODEG00000004385
ENSODEG00000004387
ENSODEG00000004388
ENSODEG00000004389
ENSODEG00000004391
ENSODEG00000004393
ENSODEG00000004395
ENSODEG00000004396
ENSODEG00000004398
ENSODEG00000004400
ENSODEG00000004401
ENSODEG00000004402
ENSODEG00000004403
ENSODEG00000004404
ENSODEG00000004405
ENSODEG00000004406
ENSODEG00000004407
ENSODEG00000004408
ENSODEG00000004409
ENSODEG00000004410
ENSODEG00000004411
ENSODEG00000004412
ENSODEG00000004413

ENSODEG00000004414
ENSODEG00000004418
ENSODEG00000004419
ENSODEG00000004420
ENSODEG00000004421
ENSODEG00000004422
ENSODEG00000004423
ENSODEG00000004424
ENSODEG00000004425
ENSODEG00000004426
ENSODEG00000004430
ENSODEG00000004432
ENSODEG00000004433
ENSODEG00000004434
ENSODEG00000004435
ENSODEG00000004436
ENSODEG00000004437
ENSODEG00000004438
ENSODEG00000004439
ENSODEG00000004440
ENSODEG00000004441
ENSODEG00000004442
ENSODEG00000004444
ENSODEG00000004445
ENSODEG00000004446
ENSODEG00000004448
ENSODEG00000004449
ENSODEG00000004450
ENSODEG00000004451
ENSODEG00000004452
ENSODEG00000004454
ENSODEG00000004455
ENSODEG00000004457
ENSODEG00000004458
ENSODEG00000004460
ENSODEG00000004463
ENSODEG00000004464
ENSODEG00000004465
ENSODEG00000004466
ENSODEG00000004467
ENSODEG00000004468
ENSODEG00000004470
ENSODEG00000004471
ENSODEG00000004474
ENSODEG00000004475
ENSODEG00000004476
ENSODEG00000004477
ENSODEG00000004481
ENSODEG00000004483
ENSODEG00000004486
ENSODEG00000004487
ENSODEG00000004488
ENSODEG00000004489
ENSODEG00000004490
ENSODEG00000004491
ENSODEG00000004492
ENSODEG00000004493
ENSODEG00000004494
ENSODEG00000004495

ENSODEG00000004497
ENSODEG00000004499
ENSODEG00000004500
ENSODEG00000004501
ENSODEG00000004502
ENSODEG00000004503
ENSODEG00000004505
ENSODEG00000004506
ENSODEG00000004507
ENSODEG00000004509
ENSODEG00000004510
ENSODEG00000004511
ENSODEG00000004514
ENSODEG00000004515
ENSODEG00000004516
ENSODEG00000004517
ENSODEG00000004518
ENSODEG00000004519
ENSODEG00000004520
ENSODEG00000004521
ENSODEG00000004523
ENSODEG00000004524
ENSODEG00000004525
ENSODEG00000004527
ENSODEG00000004528
ENSODEG00000004529
ENSODEG00000004530
ENSODEG00000004533
ENSODEG00000004534
ENSODEG00000004535
ENSODEG00000004536
ENSODEG00000004538
ENSODEG00000004540
ENSODEG00000004541
ENSODEG00000004542
ENSODEG00000004544
ENSODEG00000004545
ENSODEG00000004546
ENSODEG00000004547
ENSODEG00000004548
ENSODEG00000004549
ENSODEG00000004550
ENSODEG00000004551
ENSODEG00000004552
ENSODEG00000004553
ENSODEG00000004554
ENSODEG00000004557
ENSODEG00000004558
ENSODEG00000004559
ENSODEG00000004560
ENSODEG00000004561
ENSODEG00000004563
ENSODEG00000004564
ENSODEG00000004565
ENSODEG00000004566
ENSODEG00000004568
ENSODEG00000004572
ENSODEG00000004573
ENSODEG00000004574

ENSODEG00000004575
ENSODEG00000004576
ENSODEG00000004577
ENSODEG00000004579
ENSODEG00000004581
ENSODEG00000004582
ENSODEG00000004583
ENSODEG00000004584
ENSODEG00000004587
ENSODEG00000004588
ENSODEG00000004589
ENSODEG00000004590
ENSODEG00000004593
ENSODEG00000004594
ENSODEG00000004595
ENSODEG00000004599
ENSODEG00000004600
ENSODEG00000004601
ENSODEG00000004602
ENSODEG00000004604
ENSODEG00000004607
ENSODEG00000004610
ENSODEG00000004612
ENSODEG00000004613
ENSODEG00000004614
ENSODEG00000004615
ENSODEG00000004616
ENSODEG00000004617
ENSODEG00000004618
ENSODEG00000004619
ENSODEG00000004620
ENSODEG00000004621
ENSODEG00000004622
ENSODEG00000004624
ENSODEG00000004626
ENSODEG00000004628
ENSODEG00000004630
ENSODEG00000004632
ENSODEG00000004633
ENSODEG00000004635
ENSODEG00000004638
ENSODEG00000004639
ENSODEG00000004640
ENSODEG00000004641
ENSODEG00000004645
ENSODEG00000004646
ENSODEG00000004648
ENSODEG00000004649
ENSODEG00000004650
ENSODEG00000004653
ENSODEG00000004654
ENSODEG00000004655
ENSODEG00000004656
ENSODEG00000004659
ENSODEG00000004661
ENSODEG00000004662
ENSODEG00000004663
ENSODEG00000004664
ENSODEG00000004665

ENSODEG00000004667
ENSODEG00000004668
ENSODEG00000004669
ENSODEG00000004670
ENSODEG00000004671
ENSODEG00000004675
ENSODEG00000004678
ENSODEG00000004679
ENSODEG00000004682
ENSODEG00000004683
ENSODEG00000004686
ENSODEG00000004687
ENSODEG00000004688
ENSODEG00000004691
ENSODEG00000004693
ENSODEG00000004694
ENSODEG00000004695
ENSODEG00000004696
ENSODEG00000004697
ENSODEG00000004698
ENSODEG00000004699
ENSODEG00000004701
ENSODEG00000004702
ENSODEG00000004703
ENSODEG00000004705
ENSODEG00000004706
ENSODEG00000004708
ENSODEG00000004710
ENSODEG00000004712
ENSODEG00000004713
ENSODEG00000004714
ENSODEG00000004715
ENSODEG00000004716
ENSODEG00000004717
ENSODEG00000004718
ENSODEG00000004719
ENSODEG00000004720
ENSODEG00000004721
ENSODEG00000004723
ENSODEG00000004724
ENSODEG00000004726
ENSODEG00000004727
ENSODEG00000004728
ENSODEG00000004729
ENSODEG00000004730
ENSODEG00000004732
ENSODEG00000004733
ENSODEG00000004734
ENSODEG00000004735
ENSODEG00000004736
ENSODEG00000004737
ENSODEG00000004738
ENSODEG00000004740
ENSODEG00000004742
ENSODEG00000004744
ENSODEG00000004745
ENSODEG00000004746
ENSODEG00000004747
ENSODEG00000004748

ENSODEG00000004750
ENSODEG00000004751
ENSODEG00000004752
ENSODEG00000004753
ENSODEG00000004754
ENSODEG00000004755
ENSODEG00000004757
ENSODEG00000004758
ENSODEG00000004759
ENSODEG00000004760
ENSODEG00000004762
ENSODEG00000004764
ENSODEG00000004765
ENSODEG00000004766
ENSODEG00000004767
ENSODEG00000004768
ENSODEG00000004769
ENSODEG00000004770
ENSODEG00000004771
ENSODEG00000004772
ENSODEG00000004773
ENSODEG00000004774
ENSODEG00000004775
ENSODEG00000004776
ENSODEG00000004777
ENSODEG00000004779
ENSODEG00000004780
ENSODEG00000004781
ENSODEG00000004782
ENSODEG00000004783
ENSODEG00000004784
ENSODEG00000004788
ENSODEG00000004789
ENSODEG00000004791
ENSODEG00000004792
ENSODEG00000004793
ENSODEG00000004794
ENSODEG00000004795
ENSODEG00000004797
ENSODEG00000004799
ENSODEG00000004801
ENSODEG00000004802
ENSODEG00000004803
ENSODEG00000004804
ENSODEG00000004806
ENSODEG00000004807
ENSODEG00000004808
ENSODEG00000004809
ENSODEG00000004810
ENSODEG00000004811
ENSODEG00000004812
ENSODEG00000004813
ENSODEG00000004814
ENSODEG00000004815
ENSODEG00000004816
ENSODEG00000004817
ENSODEG00000004818
ENSODEG00000004819
ENSODEG00000004820

ENSODEG00000004821
ENSODEG00000004822
ENSODEG00000004823
ENSODEG00000004824
ENSODEG00000004825
ENSODEG00000004826
ENSODEG00000004827
ENSODEG00000004828
ENSODEG00000004829
ENSODEG00000004831
ENSODEG00000004832
ENSODEG00000004833
ENSODEG00000004836
ENSODEG00000004837
ENSODEG00000004838
ENSODEG00000004839
ENSODEG00000004840
ENSODEG00000004844
ENSODEG00000004845
ENSODEG00000004847
ENSODEG00000004848
ENSODEG00000004849
ENSODEG00000004850
ENSODEG00000004851
ENSODEG00000004852
ENSODEG00000004853
ENSODEG00000004854
ENSODEG00000004855
ENSODEG00000004856
ENSODEG00000004858
ENSODEG00000004859
ENSODEG00000004860
ENSODEG00000004861
ENSODEG00000004862
ENSODEG00000004863
ENSODEG00000004867
ENSODEG00000004868
ENSODEG00000004869
ENSODEG00000004870
ENSODEG00000004873
ENSODEG00000004874
ENSODEG00000004875
ENSODEG00000004877
ENSODEG00000004878
ENSODEG00000004879
ENSODEG00000004881
ENSODEG00000004882
ENSODEG00000004883
ENSODEG00000004884
ENSODEG00000004885
ENSODEG00000004886
ENSODEG00000004887
ENSODEG00000004888
ENSODEG00000004889
ENSODEG00000004890
ENSODEG00000004891
ENSODEG00000004892
ENSODEG00000004893
ENSODEG00000004894

ENSODEG00000004897
ENSODEG00000004898
ENSODEG00000004899
ENSODEG00000004900
ENSODEG00000004901
ENSODEG00000004902
ENSODEG00000004903
ENSODEG00000004905
ENSODEG00000004906
ENSODEG00000004907
ENSODEG00000004908
ENSODEG00000004909
ENSODEG00000004910
ENSODEG00000004913
ENSODEG00000004914
ENSODEG00000004915
ENSODEG00000004916
ENSODEG00000004917
ENSODEG00000004920
ENSODEG00000004921
ENSODEG00000004922
ENSODEG00000004924
ENSODEG00000004926
ENSODEG00000004927
ENSODEG00000004928
ENSODEG00000004929
ENSODEG00000004930
ENSODEG00000004931
ENSODEG00000004932
ENSODEG00000004934
ENSODEG00000004936
ENSODEG00000004937
ENSODEG00000004938
ENSODEG00000004939
ENSODEG00000004941
ENSODEG00000004942
ENSODEG00000004943
ENSODEG00000004945
ENSODEG00000004947
ENSODEG00000004949
ENSODEG00000004950
ENSODEG00000004951
ENSODEG00000004952
ENSODEG00000004953
ENSODEG00000004954
ENSODEG00000004955
ENSODEG00000004956
ENSODEG00000004957
ENSODEG00000004958
ENSODEG00000004959
ENSODEG00000004962
ENSODEG00000004963
ENSODEG00000004965
ENSODEG00000004969
ENSODEG00000004970
ENSODEG00000004972
ENSODEG00000004973
ENSODEG00000004974
ENSODEG00000004975

ENSODEG00000004976
ENSODEG00000004977
ENSODEG00000004978
ENSODEG00000004981
ENSODEG00000004982
ENSODEG00000004983
ENSODEG00000004984
ENSODEG00000004986
ENSODEG00000004987
ENSODEG00000004989
ENSODEG00000004990
ENSODEG00000004991
ENSODEG00000004992
ENSODEG00000004993
ENSODEG00000004995
ENSODEG00000004997
ENSODEG00000004998
ENSODEG00000004999
ENSODEG00000005000
ENSODEG00000005001
ENSODEG00000005002
ENSODEG00000005004
ENSODEG00000005006
ENSODEG00000005008
ENSODEG00000005010
ENSODEG00000005011
ENSODEG00000005014
ENSODEG00000005015
ENSODEG00000005016
ENSODEG00000005017
ENSODEG00000005018
ENSODEG00000005020
ENSODEG00000005021
ENSODEG00000005023
ENSODEG00000005025
ENSODEG00000005026
ENSODEG00000005027
ENSODEG00000005028
ENSODEG00000005030
ENSODEG00000005032
ENSODEG00000005033
ENSODEG00000005035
ENSODEG00000005036
ENSODEG00000005037
ENSODEG00000005038
ENSODEG00000005039
ENSODEG00000005040
ENSODEG00000005041
ENSODEG00000005042
ENSODEG00000005043
ENSODEG00000005044
ENSODEG00000005045
ENSODEG00000005046
ENSODEG00000005047
ENSODEG00000005048
ENSODEG00000005049
ENSODEG00000005052
ENSODEG00000005053
ENSODEG00000005055

ENSODEG00000005057
ENSODEG00000005058
ENSODEG00000005059
ENSODEG00000005062
ENSODEG00000005063
ENSODEG00000005064
ENSODEG00000005065
ENSODEG00000005066
ENSODEG00000005067
ENSODEG00000005069
ENSODEG00000005070
ENSODEG00000005071
ENSODEG00000005073
ENSODEG00000005074
ENSODEG00000005075
ENSODEG00000005076
ENSODEG00000005077
ENSODEG00000005078
ENSODEG00000005080
ENSODEG00000005082
ENSODEG00000005083
ENSODEG00000005084
ENSODEG00000005085
ENSODEG00000005086
ENSODEG00000005087
ENSODEG00000005088
ENSODEG00000005089
ENSODEG00000005090
ENSODEG00000005091
ENSODEG00000005092
ENSODEG00000005093
ENSODEG00000005094
ENSODEG00000005095
ENSODEG00000005096
ENSODEG00000005099
ENSODEG00000005101
ENSODEG00000005102
ENSODEG00000005103
ENSODEG00000005104
ENSODEG00000005105
ENSODEG00000005106
ENSODEG00000005107
ENSODEG00000005108
ENSODEG00000005109
ENSODEG00000005110
ENSODEG00000005113
ENSODEG00000005115
ENSODEG00000005116
ENSODEG00000005117
ENSODEG00000005119
ENSODEG00000005121
ENSODEG00000005122
ENSODEG00000005123
ENSODEG00000005124
ENSODEG00000005126
ENSODEG00000005129
ENSODEG00000005130
ENSODEG00000005131
ENSODEG00000005132

ENSODEG00000005134
ENSODEG00000005135
ENSODEG00000005137
ENSODEG00000005138
ENSODEG00000005139
ENSODEG00000005140
ENSODEG00000005141
ENSODEG00000005142
ENSODEG00000005143
ENSODEG00000005144
ENSODEG00000005145
ENSODEG00000005146
ENSODEG00000005147
ENSODEG00000005148
ENSODEG00000005149
ENSODEG00000005151
ENSODEG00000005152
ENSODEG00000005153
ENSODEG00000005155
ENSODEG00000005156
ENSODEG00000005157
ENSODEG00000005158
ENSODEG00000005159
ENSODEG00000005160
ENSODEG00000005163
ENSODEG00000005164
ENSODEG00000005165
ENSODEG00000005166
ENSODEG00000005168
ENSODEG00000005169
ENSODEG00000005171
ENSODEG00000005172
ENSODEG00000005173
ENSODEG00000005174
ENSODEG00000005175
ENSODEG00000005177
ENSODEG00000005178
ENSODEG00000005181
ENSODEG00000005183
ENSODEG00000005184
ENSODEG00000005185
ENSODEG00000005186
ENSODEG00000005187
ENSODEG00000005188
ENSODEG00000005189
ENSODEG00000005190
ENSODEG00000005191
ENSODEG00000005193
ENSODEG00000005194
ENSODEG00000005195
ENSODEG00000005196
ENSODEG00000005197
ENSODEG00000005198
ENSODEG00000005199
ENSODEG00000005200
ENSODEG00000005201
ENSODEG00000005202
ENSODEG00000005203
ENSODEG00000005204

ENSODEG00000005205
ENSODEG00000005207
ENSODEG00000005209
ENSODEG00000005211
ENSODEG00000005212
ENSODEG00000005214
ENSODEG00000005215
ENSODEG00000005216
ENSODEG00000005218
ENSODEG00000005220
ENSODEG00000005221
ENSODEG00000005222
ENSODEG00000005225
ENSODEG00000005227
ENSODEG00000005228
ENSODEG00000005231
ENSODEG00000005232
ENSODEG00000005233
ENSODEG00000005235
ENSODEG00000005236
ENSODEG00000005237
ENSODEG00000005238
ENSODEG00000005239
ENSODEG00000005240
ENSODEG00000005242
ENSODEG00000005243
ENSODEG00000005246
ENSODEG00000005248
ENSODEG00000005249
ENSODEG00000005250
ENSODEG00000005251
ENSODEG00000005252
ENSODEG00000005254
ENSODEG00000005255
ENSODEG00000005256
ENSODEG00000005259
ENSODEG00000005260
ENSODEG00000005261
ENSODEG00000005262
ENSODEG00000005263
ENSODEG00000005264
ENSODEG00000005265
ENSODEG00000005266
ENSODEG00000005267
ENSODEG00000005268
ENSODEG00000005269
ENSODEG00000005270
ENSODEG00000005271
ENSODEG00000005272
ENSODEG00000005273
ENSODEG00000005274
ENSODEG00000005275
ENSODEG00000005276
ENSODEG00000005277
ENSODEG00000005278
ENSODEG00000005280
ENSODEG00000005281
ENSODEG00000005282
ENSODEG00000005284

ENSODEG00000005286
ENSODEG00000005287
ENSODEG00000005290
ENSODEG00000005291
ENSODEG00000005294
ENSODEG00000005295
ENSODEG00000005296
ENSODEG00000005297
ENSODEG00000005298
ENSODEG00000005299
ENSODEG00000005300
ENSODEG00000005302
ENSODEG00000005304
ENSODEG00000005305
ENSODEG00000005306
ENSODEG00000005307
ENSODEG00000005308
ENSODEG00000005309
ENSODEG00000005310
ENSODEG00000005312
ENSODEG00000005314
ENSODEG00000005315
ENSODEG00000005317
ENSODEG00000005318
ENSODEG00000005319
ENSODEG00000005322
ENSODEG00000005323
ENSODEG00000005324
ENSODEG00000005325
ENSODEG00000005326
ENSODEG00000005328
ENSODEG00000005330
ENSODEG00000005332
ENSODEG00000005333
ENSODEG00000005334
ENSODEG00000005335
ENSODEG00000005337
ENSODEG00000005339
ENSODEG00000005340
ENSODEG00000005341
ENSODEG00000005342
ENSODEG00000005343
ENSODEG00000005344
ENSODEG00000005345
ENSODEG00000005346
ENSODEG00000005348
ENSODEG00000005349
ENSODEG00000005350
ENSODEG00000005351
ENSODEG00000005353
ENSODEG00000005354
ENSODEG00000005355
ENSODEG00000005356
ENSODEG00000005357
ENSODEG00000005358
ENSODEG00000005359
ENSODEG00000005361
ENSODEG00000005366
ENSODEG00000005368

ENSODEG00000005370
ENSODEG00000005371
ENSODEG00000005372
ENSODEG00000005373
ENSODEG00000005374
ENSODEG00000005376
ENSODEG00000005377
ENSODEG00000005378
ENSODEG00000005379
ENSODEG00000005380
ENSODEG00000005381
ENSODEG00000005382
ENSODEG00000005383
ENSODEG00000005384
ENSODEG00000005385
ENSODEG00000005386
ENSODEG00000005389
ENSODEG00000005390
ENSODEG00000005391
ENSODEG00000005392
ENSODEG00000005394
ENSODEG00000005395
ENSODEG00000005396
ENSODEG00000005397
ENSODEG00000005399
ENSODEG00000005400
ENSODEG00000005401
ENSODEG00000005402
ENSODEG00000005404
ENSODEG00000005405
ENSODEG00000005407
ENSODEG00000005408
ENSODEG00000005409
ENSODEG00000005410
ENSODEG00000005412
ENSODEG00000005413
ENSODEG00000005414
ENSODEG00000005415
ENSODEG00000005418
ENSODEG00000005419
ENSODEG00000005420
ENSODEG00000005421
ENSODEG00000005423
ENSODEG00000005424
ENSODEG00000005425
ENSODEG00000005426
ENSODEG00000005427
ENSODEG00000005428
ENSODEG00000005429
ENSODEG00000005430
ENSODEG00000005431
ENSODEG00000005432
ENSODEG00000005433
ENSODEG00000005435
ENSODEG00000005437
ENSODEG00000005438
ENSODEG00000005439
ENSODEG00000005443
ENSODEG00000005444

ENSODEG00000005445
ENSODEG00000005446
ENSODEG00000005447
ENSODEG00000005448
ENSODEG00000005449
ENSODEG00000005450
ENSODEG00000005451
ENSODEG00000005452
ENSODEG00000005454
ENSODEG00000005455
ENSODEG00000005456
ENSODEG00000005457
ENSODEG00000005461
ENSODEG00000005462
ENSODEG00000005464
ENSODEG00000005465
ENSODEG00000005466
ENSODEG00000005467
ENSODEG00000005468
ENSODEG00000005469
ENSODEG00000005470
ENSODEG00000005471
ENSODEG00000005472
ENSODEG00000005474
ENSODEG00000005475
ENSODEG00000005476
ENSODEG00000005477
ENSODEG00000005478
ENSODEG00000005479
ENSODEG00000005480
ENSODEG00000005482
ENSODEG00000005483
ENSODEG00000005485
ENSODEG00000005486
ENSODEG00000005487
ENSODEG00000005488
ENSODEG00000005489
ENSODEG00000005490
ENSODEG00000005492
ENSODEG00000005493
ENSODEG00000005494
ENSODEG00000005495
ENSODEG00000005496
ENSODEG00000005497
ENSODEG00000005499
ENSODEG00000005501
ENSODEG00000005502
ENSODEG00000005504
ENSODEG00000005505
ENSODEG00000005506
ENSODEG00000005507
ENSODEG00000005508
ENSODEG00000005509
ENSODEG00000005510
ENSODEG00000005511
ENSODEG00000005512
ENSODEG00000005514
ENSODEG00000005515
ENSODEG00000005516

ENSODEG00000005517
ENSODEG00000005519
ENSODEG00000005520
ENSODEG00000005521
ENSODEG00000005522
ENSODEG00000005523
ENSODEG00000005524
ENSODEG00000005527
ENSODEG00000005528
ENSODEG00000005529
ENSODEG00000005531
ENSODEG00000005533
ENSODEG00000005534
ENSODEG00000005535
ENSODEG00000005537
ENSODEG00000005538
ENSODEG00000005539
ENSODEG00000005543
ENSODEG00000005544
ENSODEG00000005545
ENSODEG00000005549
ENSODEG00000005550
ENSODEG00000005552
ENSODEG00000005553
ENSODEG00000005554
ENSODEG00000005555
ENSODEG00000005557
ENSODEG00000005558
ENSODEG00000005559
ENSODEG00000005562
ENSODEG00000005565
ENSODEG00000005566
ENSODEG00000005568
ENSODEG00000005569
ENSODEG00000005570
ENSODEG00000005573
ENSODEG00000005574
ENSODEG00000005575
ENSODEG00000005576
ENSODEG00000005578
ENSODEG00000005579
ENSODEG00000005581
ENSODEG00000005582
ENSODEG00000005583
ENSODEG00000005584
ENSODEG00000005585
ENSODEG00000005586
ENSODEG00000005587
ENSODEG00000005588
ENSODEG00000005589
ENSODEG00000005590
ENSODEG00000005591
ENSODEG00000005592
ENSODEG00000005593
ENSODEG00000005594
ENSODEG00000005595
ENSODEG00000005596
ENSODEG00000005597
ENSODEG00000005598

ENSODEG00000005599
ENSODEG00000005600
ENSODEG00000005601
ENSODEG00000005602
ENSODEG00000005603
ENSODEG00000005605
ENSODEG00000005606
ENSODEG00000005607
ENSODEG00000005608
ENSODEG00000005609
ENSODEG00000005610
ENSODEG00000005612
ENSODEG00000005613
ENSODEG00000005615
ENSODEG00000005616
ENSODEG00000005617
ENSODEG00000005620
ENSODEG00000005622
ENSODEG00000005623
ENSODEG00000005625
ENSODEG00000005627
ENSODEG00000005628
ENSODEG00000005629
ENSODEG00000005630
ENSODEG00000005631
ENSODEG00000005632
ENSODEG00000005633
ENSODEG00000005634
ENSODEG00000005635
ENSODEG00000005637
ENSODEG00000005638
ENSODEG00000005639
ENSODEG00000005640
ENSODEG00000005642
ENSODEG00000005643
ENSODEG00000005644
ENSODEG00000005645
ENSODEG00000005646
ENSODEG00000005648
ENSODEG00000005649
ENSODEG00000005650
ENSODEG00000005655
ENSODEG00000005656
ENSODEG00000005657
ENSODEG00000005659
ENSODEG00000005660
ENSODEG00000005662
ENSODEG00000005663
ENSODEG00000005664
ENSODEG00000005665
ENSODEG00000005667
ENSODEG00000005668
ENSODEG00000005669
ENSODEG00000005670
ENSODEG00000005671
ENSODEG00000005672
ENSODEG00000005673
ENSODEG00000005675
ENSODEG00000005676

ENSODEG00000005679
ENSODEG00000005680
ENSODEG00000005681
ENSODEG00000005682
ENSODEG00000005683
ENSODEG00000005685
ENSODEG00000005687
ENSODEG00000005688
ENSODEG00000005689
ENSODEG00000005690
ENSODEG00000005691
ENSODEG00000005694
ENSODEG00000005696
ENSODEG00000005697
ENSODEG00000005698
ENSODEG00000005699
ENSODEG00000005701
ENSODEG00000005702
ENSODEG00000005705
ENSODEG00000005706
ENSODEG00000005707
ENSODEG00000005708
ENSODEG00000005709
ENSODEG00000005711
ENSODEG00000005712
ENSODEG00000005713
ENSODEG00000005715
ENSODEG00000005716
ENSODEG00000005717
ENSODEG00000005718
ENSODEG00000005719
ENSODEG00000005720
ENSODEG00000005721
ENSODEG00000005724
ENSODEG00000005725
ENSODEG00000005726
ENSODEG00000005728
ENSODEG00000005730
ENSODEG00000005731
ENSODEG00000005733
ENSODEG00000005735
ENSODEG00000005736
ENSODEG00000005738
ENSODEG00000005739
ENSODEG00000005740
ENSODEG00000005741
ENSODEG00000005742
ENSODEG00000005743
ENSODEG00000005745
ENSODEG00000005746
ENSODEG00000005747
ENSODEG00000005748
ENSODEG00000005749
ENSODEG00000005750
ENSODEG00000005751
ENSODEG00000005752
ENSODEG00000005753
ENSODEG00000005754
ENSODEG00000005755

ENSODEG00000005756
ENSODEG00000005758
ENSODEG00000005759
ENSODEG00000005761
ENSODEG00000005763
ENSODEG00000005766
ENSODEG00000005767
ENSODEG00000005769
ENSODEG00000005770
ENSODEG00000005772
ENSODEG00000005773
ENSODEG00000005775
ENSODEG00000005776
ENSODEG00000005778
ENSODEG00000005782
ENSODEG00000005783
ENSODEG00000005784
ENSODEG00000005786
ENSODEG00000005788
ENSODEG00000005789
ENSODEG00000005791
ENSODEG00000005792
ENSODEG00000005793
ENSODEG00000005794
ENSODEG00000005795
ENSODEG00000005796
ENSODEG00000005797
ENSODEG00000005798
ENSODEG00000005800
ENSODEG00000005803
ENSODEG00000005804
ENSODEG00000005805
ENSODEG00000005806
ENSODEG00000005807
ENSODEG00000005808
ENSODEG00000005809
ENSODEG00000005810
ENSODEG00000005811
ENSODEG00000005812
ENSODEG00000005814
ENSODEG00000005815
ENSODEG00000005816
ENSODEG00000005817
ENSODEG00000005820
ENSODEG00000005821
ENSODEG00000005822
ENSODEG00000005823
ENSODEG00000005824
ENSODEG00000005825
ENSODEG00000005826
ENSODEG00000005827
ENSODEG00000005828
ENSODEG00000005832
ENSODEG00000005833
ENSODEG00000005836
ENSODEG00000005837
ENSODEG00000005838
ENSODEG00000005839
ENSODEG00000005840

ENSODEG00000005841
ENSODEG00000005842
ENSODEG00000005843
ENSODEG00000005845
ENSODEG00000005846
ENSODEG00000005847
ENSODEG00000005848
ENSODEG00000005849
ENSODEG00000005850
ENSODEG00000005851
ENSODEG00000005852
ENSODEG00000005853
ENSODEG00000005854
ENSODEG00000005855
ENSODEG00000005856
ENSODEG00000005858
ENSODEG00000005859
ENSODEG00000005860
ENSODEG00000005861
ENSODEG00000005862
ENSODEG00000005863
ENSODEG00000005864
ENSODEG00000005865
ENSODEG00000005867
ENSODEG00000005868
ENSODEG00000005870
ENSODEG00000005871
ENSODEG00000005872
ENSODEG00000005873
ENSODEG00000005874
ENSODEG00000005875
ENSODEG00000005877
ENSODEG00000005878
ENSODEG00000005879
ENSODEG00000005880
ENSODEG00000005883
ENSODEG00000005884
ENSODEG00000005886
ENSODEG00000005888
ENSODEG00000005890
ENSODEG00000005891
ENSODEG00000005892
ENSODEG00000005894
ENSODEG00000005897
ENSODEG00000005899
ENSODEG00000005901
ENSODEG00000005903
ENSODEG00000005906
ENSODEG00000005907
ENSODEG00000005908
ENSODEG00000005909
ENSODEG00000005911
ENSODEG00000005912
ENSODEG00000005914
ENSODEG00000005915
ENSODEG00000005917
ENSODEG00000005919
ENSODEG00000005920
ENSODEG00000005922

ENSODEG00000005923
ENSODEG00000005924
ENSODEG00000005925
ENSODEG00000005926
ENSODEG00000005927
ENSODEG00000005928
ENSODEG00000005929
ENSODEG00000005930
ENSODEG00000005931
ENSODEG00000005933
ENSODEG00000005934
ENSODEG00000005935
ENSODEG00000005936
ENSODEG00000005937
ENSODEG00000005938
ENSODEG00000005939
ENSODEG00000005941
ENSODEG00000005944
ENSODEG00000005945
ENSODEG00000005947
ENSODEG00000005948
ENSODEG00000005949
ENSODEG00000005950
ENSODEG00000005951
ENSODEG00000005952
ENSODEG00000005953
ENSODEG00000005954
ENSODEG00000005955
ENSODEG00000005956
ENSODEG00000005959
ENSODEG00000005960
ENSODEG00000005961
ENSODEG00000005962
ENSODEG00000005963
ENSODEG00000005964
ENSODEG00000005965
ENSODEG00000005966
ENSODEG00000005968
ENSODEG00000005969
ENSODEG00000005970
ENSODEG00000005972
ENSODEG00000005973
ENSODEG00000005974
ENSODEG00000005975
ENSODEG00000005976
ENSODEG00000005977
ENSODEG00000005978
ENSODEG00000005979
ENSODEG00000005980
ENSODEG00000005981
ENSODEG00000005983
ENSODEG00000005984
ENSODEG00000005985
ENSODEG00000005986
ENSODEG00000005987
ENSODEG00000005989
ENSODEG00000005990
ENSODEG00000005991
ENSODEG00000005992

ENSODEG00000005993
ENSODEG00000005994
ENSODEG00000005995
ENSODEG00000005996
ENSODEG00000005997
ENSODEG00000005999
ENSODEG00000006000
ENSODEG00000006001
ENSODEG00000006002
ENSODEG00000006004
ENSODEG00000006005
ENSODEG00000006006
ENSODEG00000006007
ENSODEG00000006008
ENSODEG00000006009
ENSODEG00000006010
ENSODEG00000006011
ENSODEG00000006013
ENSODEG00000006014
ENSODEG00000006016
ENSODEG00000006017
ENSODEG00000006018
ENSODEG00000006019
ENSODEG00000006020
ENSODEG00000006022
ENSODEG00000006023
ENSODEG00000006025
ENSODEG00000006026
ENSODEG00000006027
ENSODEG00000006032
ENSODEG00000006033
ENSODEG00000006034
ENSODEG00000006035
ENSODEG00000006036
ENSODEG00000006037
ENSODEG00000006038
ENSODEG00000006040
ENSODEG00000006041
ENSODEG00000006042
ENSODEG00000006043
ENSODEG00000006044
ENSODEG00000006045
ENSODEG00000006046
ENSODEG00000006047
ENSODEG00000006049
ENSODEG00000006050
ENSODEG00000006051
ENSODEG00000006052
ENSODEG00000006053
ENSODEG00000006054
ENSODEG00000006057
ENSODEG00000006058
ENSODEG00000006059
ENSODEG00000006060
ENSODEG00000006062
ENSODEG00000006063
ENSODEG00000006064
ENSODEG00000006065
ENSODEG00000006066

ENSODEG00000006068
ENSODEG00000006071
ENSODEG00000006072
ENSODEG00000006073
ENSODEG00000006076
ENSODEG00000006077
ENSODEG00000006078
ENSODEG00000006079
ENSODEG00000006080
ENSODEG00000006081
ENSODEG00000006083
ENSODEG00000006084
ENSODEG00000006085
ENSODEG00000006088
ENSODEG00000006089
ENSODEG00000006090
ENSODEG00000006091
ENSODEG00000006092
ENSODEG00000006093
ENSODEG00000006095
ENSODEG00000006097
ENSODEG00000006098
ENSODEG00000006099
ENSODEG00000006100
ENSODEG00000006101
ENSODEG00000006102
ENSODEG00000006103
ENSODEG00000006107
ENSODEG00000006108
ENSODEG00000006111
ENSODEG00000006113
ENSODEG00000006114
ENSODEG00000006116
ENSODEG00000006117
ENSODEG00000006119
ENSODEG00000006120
ENSODEG00000006121
ENSODEG00000006122
ENSODEG00000006123
ENSODEG00000006124
ENSODEG00000006126
ENSODEG00000006128
ENSODEG00000006129
ENSODEG00000006130
ENSODEG00000006131
ENSODEG00000006132
ENSODEG00000006133
ENSODEG00000006134
ENSODEG00000006135
ENSODEG00000006136
ENSODEG00000006137
ENSODEG00000006138
ENSODEG00000006139
ENSODEG00000006140
ENSODEG00000006141
ENSODEG00000006142
ENSODEG00000006143
ENSODEG00000006145
ENSODEG00000006146

ENSODEG00000006147
ENSODEG00000006148
ENSODEG00000006149
ENSODEG00000006150
ENSODEG00000006151
ENSODEG00000006153
ENSODEG00000006155
ENSODEG00000006156
ENSODEG00000006157
ENSODEG00000006158
ENSODEG00000006160
ENSODEG00000006162
ENSODEG00000006163
ENSODEG00000006170
ENSODEG00000006172
ENSODEG00000006175
ENSODEG00000006176
ENSODEG00000006177
ENSODEG00000006178
ENSODEG00000006182
ENSODEG00000006183
ENSODEG00000006184
ENSODEG00000006186
ENSODEG00000006188
ENSODEG00000006190
ENSODEG00000006191
ENSODEG00000006192
ENSODEG00000006193
ENSODEG00000006194
ENSODEG00000006195
ENSODEG00000006196
ENSODEG00000006197
ENSODEG00000006198
ENSODEG00000006199
ENSODEG00000006201
ENSODEG00000006202
ENSODEG00000006203
ENSODEG00000006205
ENSODEG00000006207
ENSODEG00000006208
ENSODEG00000006209
ENSODEG00000006210
ENSODEG00000006216
ENSODEG00000006219
ENSODEG00000006220
ENSODEG00000006221
ENSODEG00000006222
ENSODEG00000006224
ENSODEG00000006225
ENSODEG00000006228
ENSODEG00000006229
ENSODEG00000006230
ENSODEG00000006231
ENSODEG00000006232
ENSODEG00000006233
ENSODEG00000006234
ENSODEG00000006235
ENSODEG00000006237
ENSODEG00000006238

ENSODEG00000006239
ENSODEG00000006240
ENSODEG00000006241
ENSODEG00000006243
ENSODEG00000006244
ENSODEG00000006245
ENSODEG00000006246
ENSODEG00000006247
ENSODEG00000006248
ENSODEG00000006250
ENSODEG00000006251
ENSODEG00000006253
ENSODEG00000006254
ENSODEG00000006255
ENSODEG00000006256
ENSODEG00000006257
ENSODEG00000006258
ENSODEG00000006259
ENSODEG00000006262
ENSODEG00000006265
ENSODEG00000006267
ENSODEG00000006268
ENSODEG00000006270
ENSODEG00000006271
ENSODEG00000006272
ENSODEG00000006273
ENSODEG00000006274
ENSODEG00000006275
ENSODEG00000006276
ENSODEG00000006278
ENSODEG00000006279
ENSODEG00000006280
ENSODEG00000006281
ENSODEG00000006282
ENSODEG00000006283
ENSODEG00000006285
ENSODEG00000006286
ENSODEG00000006288
ENSODEG00000006289
ENSODEG00000006290
ENSODEG00000006291
ENSODEG00000006292
ENSODEG00000006294
ENSODEG00000006295
ENSODEG00000006296
ENSODEG00000006297
ENSODEG00000006298
ENSODEG00000006299
ENSODEG00000006300
ENSODEG00000006301
ENSODEG00000006302
ENSODEG00000006303
ENSODEG00000006304
ENSODEG00000006305
ENSODEG00000006307
ENSODEG00000006308
ENSODEG00000006309
ENSODEG00000006310
ENSODEG00000006311

ENSODEG00000006312
ENSODEG00000006313
ENSODEG00000006314
ENSODEG00000006315
ENSODEG00000006316
ENSODEG00000006317
ENSODEG00000006318
ENSODEG00000006319
ENSODEG00000006320
ENSODEG00000006322
ENSODEG00000006323
ENSODEG00000006324
ENSODEG00000006325
ENSODEG00000006326
ENSODEG00000006329
ENSODEG00000006331
ENSODEG00000006333
ENSODEG00000006334
ENSODEG00000006335
ENSODEG00000006336
ENSODEG00000006337
ENSODEG00000006338
ENSODEG00000006341
ENSODEG00000006342
ENSODEG00000006343
ENSODEG00000006344
ENSODEG00000006345
ENSODEG00000006347
ENSODEG00000006348
ENSODEG00000006349
ENSODEG00000006350
ENSODEG00000006351
ENSODEG00000006352
ENSODEG00000006353
ENSODEG00000006354
ENSODEG00000006356
ENSODEG00000006359
ENSODEG00000006360
ENSODEG00000006361
ENSODEG00000006362
ENSODEG00000006364
ENSODEG00000006365
ENSODEG00000006366
ENSODEG00000006367
ENSODEG00000006368
ENSODEG00000006370
ENSODEG00000006371
ENSODEG00000006372
ENSODEG00000006373
ENSODEG00000006375
ENSODEG00000006377
ENSODEG00000006379
ENSODEG00000006380
ENSODEG00000006382
ENSODEG00000006385
ENSODEG00000006388
ENSODEG00000006389
ENSODEG00000006390
ENSODEG00000006391

ENSODEG00000006392
ENSODEG00000006394
ENSODEG00000006395
ENSODEG00000006396
ENSODEG00000006398
ENSODEG00000006399
ENSODEG00000006400
ENSODEG00000006401
ENSODEG00000006402
ENSODEG00000006404
ENSODEG00000006406
ENSODEG00000006407
ENSODEG00000006408
ENSODEG00000006412
ENSODEG00000006413
ENSODEG00000006414
ENSODEG00000006415
ENSODEG00000006416
ENSODEG00000006417
ENSODEG00000006419
ENSODEG00000006420
ENSODEG00000006422
ENSODEG00000006425
ENSODEG00000006426
ENSODEG00000006429
ENSODEG00000006430
ENSODEG00000006431
ENSODEG00000006432
ENSODEG00000006433
ENSODEG00000006434
ENSODEG00000006437
ENSODEG00000006438
ENSODEG00000006440
ENSODEG00000006442
ENSODEG00000006443
ENSODEG00000006444
ENSODEG00000006445
ENSODEG00000006446
ENSODEG00000006447
ENSODEG00000006448
ENSODEG00000006449
ENSODEG00000006450
ENSODEG00000006451
ENSODEG00000006452
ENSODEG00000006453
ENSODEG00000006454
ENSODEG00000006455
ENSODEG00000006457
ENSODEG00000006460
ENSODEG00000006461
ENSODEG00000006462
ENSODEG00000006463
ENSODEG00000006464
ENSODEG00000006465
ENSODEG00000006466
ENSODEG00000006467
ENSODEG00000006471
ENSODEG00000006472
ENSODEG00000006473

ENSODEG00000006475
ENSODEG00000006476
ENSODEG00000006477
ENSODEG00000006479
ENSODEG00000006480
ENSODEG00000006481
ENSODEG00000006482
ENSODEG00000006483
ENSODEG00000006485
ENSODEG00000006486
ENSODEG00000006487
ENSODEG00000006488
ENSODEG00000006489
ENSODEG00000006490
ENSODEG00000006492
ENSODEG00000006493
ENSODEG00000006494
ENSODEG00000006497
ENSODEG00000006498
ENSODEG00000006499
ENSODEG00000006500
ENSODEG00000006501
ENSODEG00000006504
ENSODEG00000006506
ENSODEG00000006507
ENSODEG00000006508
ENSODEG00000006511
ENSODEG00000006512
ENSODEG00000006513
ENSODEG00000006514
ENSODEG00000006515
ENSODEG00000006516
ENSODEG00000006517
ENSODEG00000006518
ENSODEG00000006520
ENSODEG00000006521
ENSODEG00000006522
ENSODEG00000006526
ENSODEG00000006527
ENSODEG00000006528
ENSODEG00000006529
ENSODEG00000006530
ENSODEG00000006531
ENSODEG00000006532
ENSODEG00000006533
ENSODEG00000006534
ENSODEG00000006535
ENSODEG00000006537
ENSODEG00000006539
ENSODEG00000006540
ENSODEG00000006541
ENSODEG00000006542
ENSODEG00000006545
ENSODEG00000006546
ENSODEG00000006547
ENSODEG00000006548
ENSODEG00000006549
ENSODEG00000006551
ENSODEG00000006554

ENSODEG00000006556
ENSODEG00000006557
ENSODEG00000006558
ENSODEG00000006559
ENSODEG00000006560
ENSODEG00000006561
ENSODEG00000006562
ENSODEG00000006563
ENSODEG00000006564
ENSODEG00000006565
ENSODEG00000006566
ENSODEG00000006568
ENSODEG00000006569
ENSODEG00000006570
ENSODEG00000006571
ENSODEG00000006572
ENSODEG00000006573
ENSODEG00000006574
ENSODEG00000006575
ENSODEG00000006576
ENSODEG00000006577
ENSODEG00000006578
ENSODEG00000006579
ENSODEG00000006581
ENSODEG00000006583
ENSODEG00000006584
ENSODEG00000006585
ENSODEG00000006586
ENSODEG00000006587
ENSODEG00000006588
ENSODEG00000006589
ENSODEG00000006590
ENSODEG00000006591
ENSODEG00000006592
ENSODEG00000006593
ENSODEG00000006594
ENSODEG00000006595
ENSODEG00000006596
ENSODEG00000006597
ENSODEG00000006598
ENSODEG00000006599
ENSODEG00000006600
ENSODEG00000006602
ENSODEG00000006603
ENSODEG00000006605
ENSODEG00000006606
ENSODEG00000006608
ENSODEG00000006609
ENSODEG00000006611
ENSODEG00000006612
ENSODEG00000006613
ENSODEG00000006614
ENSODEG00000006615
ENSODEG00000006617
ENSODEG00000006618
ENSODEG00000006619
ENSODEG00000006620
ENSODEG00000006621
ENSODEG00000006623

ENSODEG00000006624
ENSODEG00000006625
ENSODEG00000006626
ENSODEG00000006627
ENSODEG00000006630
ENSODEG00000006632
ENSODEG00000006633
ENSODEG00000006635
ENSODEG00000006636
ENSODEG00000006637
ENSODEG00000006638
ENSODEG00000006639
ENSODEG00000006643
ENSODEG00000006644
ENSODEG00000006646
ENSODEG00000006647
ENSODEG00000006648
ENSODEG00000006650
ENSODEG00000006651
ENSODEG00000006652
ENSODEG00000006653
ENSODEG00000006655
ENSODEG00000006656
ENSODEG00000006657
ENSODEG00000006660
ENSODEG00000006663
ENSODEG00000006664
ENSODEG00000006665
ENSODEG00000006666
ENSODEG00000006668
ENSODEG00000006669
ENSODEG00000006670
ENSODEG00000006671
ENSODEG00000006673
ENSODEG00000006674
ENSODEG00000006676
ENSODEG00000006677
ENSODEG00000006678
ENSODEG00000006679
ENSODEG00000006680
ENSODEG00000006681
ENSODEG00000006682
ENSODEG00000006683
ENSODEG00000006685
ENSODEG00000006688
ENSODEG00000006689
ENSODEG00000006691
ENSODEG00000006692
ENSODEG00000006693
ENSODEG00000006694
ENSODEG00000006695
ENSODEG00000006697
ENSODEG00000006699
ENSODEG00000006701
ENSODEG00000006702
ENSODEG00000006704
ENSODEG00000006706
ENSODEG00000006707
ENSODEG00000006708

ENSODEG00000006709
ENSODEG00000006711
ENSODEG00000006713
ENSODEG00000006715
ENSODEG00000006717
ENSODEG00000006718
ENSODEG00000006720
ENSODEG00000006722
ENSODEG00000006724
ENSODEG00000006725
ENSODEG00000006726
ENSODEG00000006727
ENSODEG00000006728
ENSODEG00000006729
ENSODEG00000006732
ENSODEG00000006735
ENSODEG00000006736
ENSODEG00000006737
ENSODEG00000006739
ENSODEG00000006740
ENSODEG00000006741
ENSODEG00000006742
ENSODEG00000006743
ENSODEG00000006744
ENSODEG00000006746
ENSODEG00000006747
ENSODEG00000006748
ENSODEG00000006749
ENSODEG00000006751
ENSODEG00000006752
ENSODEG00000006753
ENSODEG00000006754
ENSODEG00000006755
ENSODEG00000006756
ENSODEG00000006757
ENSODEG00000006758
ENSODEG00000006759
ENSODEG00000006760
ENSODEG00000006762
ENSODEG00000006763
ENSODEG00000006765
ENSODEG00000006766
ENSODEG00000006768
ENSODEG00000006769
ENSODEG00000006771
ENSODEG00000006772
ENSODEG00000006773
ENSODEG00000006774
ENSODEG00000006775
ENSODEG00000006778
ENSODEG00000006783
ENSODEG00000006785
ENSODEG00000006788
ENSODEG00000006789
ENSODEG00000006790
ENSODEG00000006791
ENSODEG00000006792
ENSODEG00000006793
ENSODEG00000006795

ENSODEG00000006796
ENSODEG00000006797
ENSODEG00000006798
ENSODEG00000006799
ENSODEG00000006800
ENSODEG00000006801
ENSODEG00000006802
ENSODEG00000006803
ENSODEG00000006804
ENSODEG00000006806
ENSODEG00000006807
ENSODEG00000006808
ENSODEG00000006809
ENSODEG00000006810
ENSODEG00000006811
ENSODEG00000006812
ENSODEG00000006814
ENSODEG00000006815
ENSODEG00000006816
ENSODEG00000006817
ENSODEG00000006818
ENSODEG00000006819
ENSODEG00000006821
ENSODEG00000006822
ENSODEG00000006823
ENSODEG00000006824
ENSODEG00000006825
ENSODEG00000006826
ENSODEG00000006827
ENSODEG00000006828
ENSODEG00000006829
ENSODEG00000006830
ENSODEG00000006831
ENSODEG00000006833
ENSODEG00000006835
ENSODEG00000006838
ENSODEG00000006840
ENSODEG00000006842
ENSODEG00000006844
ENSODEG00000006846
ENSODEG00000006847
ENSODEG00000006848
ENSODEG00000006850
ENSODEG00000006851
ENSODEG00000006852
ENSODEG00000006853
ENSODEG00000006854
ENSODEG00000006855
ENSODEG00000006858
ENSODEG00000006861
ENSODEG00000006863
ENSODEG00000006864
ENSODEG00000006867
ENSODEG00000006868
ENSODEG00000006870
ENSODEG00000006871
ENSODEG00000006872
ENSODEG00000006874
ENSODEG00000006875

ENSODEG00000006876
ENSODEG00000006877
ENSODEG00000006878
ENSODEG00000006879
ENSODEG00000006880
ENSODEG00000006881
ENSODEG00000006883
ENSODEG00000006884
ENSODEG00000006885
ENSODEG00000006886
ENSODEG00000006888
ENSODEG00000006889
ENSODEG00000006891
ENSODEG00000006892
ENSODEG00000006893
ENSODEG00000006896
ENSODEG00000006897
ENSODEG00000006898
ENSODEG00000006899
ENSODEG00000006900
ENSODEG00000006902
ENSODEG00000006904
ENSODEG00000006905
ENSODEG00000006906
ENSODEG00000006907
ENSODEG00000006908
ENSODEG00000006912
ENSODEG00000006913
ENSODEG00000006914
ENSODEG00000006915
ENSODEG00000006917
ENSODEG00000006918
ENSODEG00000006919
ENSODEG00000006920
ENSODEG00000006922
ENSODEG00000006924
ENSODEG00000006926
ENSODEG00000006927
ENSODEG00000006928
ENSODEG00000006929
ENSODEG00000006930
ENSODEG00000006931
ENSODEG00000006932
ENSODEG00000006933
ENSODEG00000006934
ENSODEG00000006935
ENSODEG00000006936
ENSODEG00000006937
ENSODEG00000006938
ENSODEG00000006939
ENSODEG00000006940
ENSODEG00000006941
ENSODEG00000006942
ENSODEG00000006943
ENSODEG00000006944
ENSODEG00000006946
ENSODEG00000006947
ENSODEG00000006949
ENSODEG00000006950

ENSODEG00000006952
ENSODEG00000006953
ENSODEG00000006954
ENSODEG00000006956
ENSODEG00000006957
ENSODEG00000006958
ENSODEG00000006960
ENSODEG00000006962
ENSODEG00000006963
ENSODEG00000006965
ENSODEG00000006966
ENSODEG00000006967
ENSODEG00000006968
ENSODEG00000006969
ENSODEG00000006970
ENSODEG00000006972
ENSODEG00000006974
ENSODEG00000006978
ENSODEG00000006981
ENSODEG00000006983
ENSODEG00000006984
ENSODEG00000006985
ENSODEG00000006986
ENSODEG00000006988
ENSODEG00000006990
ENSODEG00000006991
ENSODEG00000006993
ENSODEG00000006994
ENSODEG00000006995
ENSODEG00000006997
ENSODEG00000006998
ENSODEG00000006999
ENSODEG00000007000
ENSODEG00000007001
ENSODEG00000007002
ENSODEG00000007003
ENSODEG00000007004
ENSODEG00000007006
ENSODEG00000007008
ENSODEG00000007009
ENSODEG00000007010
ENSODEG00000007013
ENSODEG00000007016
ENSODEG00000007017
ENSODEG00000007018
ENSODEG00000007019
ENSODEG00000007020
ENSODEG00000007022
ENSODEG00000007023
ENSODEG00000007024
ENSODEG00000007025
ENSODEG00000007026
ENSODEG00000007028
ENSODEG00000007029
ENSODEG00000007030
ENSODEG00000007032
ENSODEG00000007033
ENSODEG00000007034
ENSODEG00000007038

ENSODEG00000007039
ENSODEG00000007040
ENSODEG00000007042
ENSODEG00000007043
ENSODEG00000007045
ENSODEG00000007046
ENSODEG00000007050
ENSODEG00000007051
ENSODEG00000007052
ENSODEG00000007053
ENSODEG00000007054
ENSODEG00000007055
ENSODEG00000007056
ENSODEG00000007057
ENSODEG00000007059
ENSODEG00000007060
ENSODEG00000007061
ENSODEG00000007067
ENSODEG00000007068
ENSODEG00000007069
ENSODEG00000007070
ENSODEG00000007071
ENSODEG00000007072
ENSODEG00000007074
ENSODEG00000007075
ENSODEG00000007076
ENSODEG00000007078
ENSODEG00000007079
ENSODEG00000007080
ENSODEG00000007081
ENSODEG00000007082
ENSODEG00000007083
ENSODEG00000007084
ENSODEG00000007086
ENSODEG00000007087
ENSODEG00000007088
ENSODEG00000007090
ENSODEG00000007092
ENSODEG00000007093
ENSODEG00000007094
ENSODEG00000007095
ENSODEG00000007096
ENSODEG00000007097
ENSODEG00000007098
ENSODEG00000007099
ENSODEG00000007100
ENSODEG00000007101
ENSODEG00000007102
ENSODEG00000007103
ENSODEG00000007104
ENSODEG00000007105
ENSODEG00000007106
ENSODEG00000007109
ENSODEG00000007110
ENSODEG00000007112
ENSODEG00000007113
ENSODEG00000007115
ENSODEG00000007116
ENSODEG00000007117

ENSODEG00000007118
ENSODEG00000007119
ENSODEG00000007120
ENSODEG00000007121
ENSODEG00000007122
ENSODEG00000007123
ENSODEG00000007124
ENSODEG00000007125
ENSODEG00000007128
ENSODEG00000007129
ENSODEG00000007131
ENSODEG00000007132
ENSODEG00000007134
ENSODEG00000007137
ENSODEG00000007139
ENSODEG00000007141
ENSODEG00000007142
ENSODEG00000007143
ENSODEG00000007147
ENSODEG00000007148
ENSODEG00000007149
ENSODEG00000007150
ENSODEG00000007151
ENSODEG00000007152
ENSODEG00000007153
ENSODEG00000007155
ENSODEG00000007158
ENSODEG00000007159
ENSODEG00000007160
ENSODEG00000007163
ENSODEG00000007164
ENSODEG00000007165
ENSODEG00000007166
ENSODEG00000007170
ENSODEG00000007173
ENSODEG00000007174
ENSODEG00000007176
ENSODEG00000007179
ENSODEG00000007181
ENSODEG00000007182
ENSODEG00000007183
ENSODEG00000007184
ENSODEG00000007186
ENSODEG00000007187
ENSODEG00000007188
ENSODEG00000007189
ENSODEG00000007190
ENSODEG00000007191
ENSODEG00000007192
ENSODEG00000007193
ENSODEG00000007194
ENSODEG00000007195
ENSODEG00000007196
ENSODEG00000007198
ENSODEG00000007199
ENSODEG00000007200
ENSODEG00000007202
ENSODEG00000007203
ENSODEG00000007204

ENSODEG00000007206
ENSODEG00000007207
ENSODEG00000007208
ENSODEG00000007210
ENSODEG00000007212
ENSODEG00000007213
ENSODEG00000007215
ENSODEG00000007217
ENSODEG00000007220
ENSODEG00000007221
ENSODEG00000007222
ENSODEG00000007223
ENSODEG00000007224
ENSODEG00000007228
ENSODEG00000007229
ENSODEG00000007230
ENSODEG00000007231
ENSODEG00000007232
ENSODEG00000007233
ENSODEG00000007236
ENSODEG00000007237
ENSODEG00000007238
ENSODEG00000007239
ENSODEG00000007240
ENSODEG00000007241
ENSODEG00000007242
ENSODEG00000007243
ENSODEG00000007244
ENSODEG00000007245
ENSODEG00000007246
ENSODEG00000007247
ENSODEG00000007248
ENSODEG00000007249
ENSODEG00000007250
ENSODEG00000007251
ENSODEG00000007252
ENSODEG00000007253
ENSODEG00000007254
ENSODEG00000007255
ENSODEG00000007256
ENSODEG00000007258
ENSODEG00000007259
ENSODEG00000007260
ENSODEG00000007261
ENSODEG00000007262
ENSODEG00000007263
ENSODEG00000007264
ENSODEG00000007265
ENSODEG00000007266
ENSODEG00000007268
ENSODEG00000007269
ENSODEG00000007270
ENSODEG00000007273
ENSODEG00000007275
ENSODEG00000007276
ENSODEG00000007277
ENSODEG00000007278
ENSODEG00000007279
ENSODEG00000007281

ENSODEG00000007282
ENSODEG00000007283
ENSODEG00000007284
ENSODEG00000007286
ENSODEG00000007287
ENSODEG00000007288
ENSODEG00000007289
ENSODEG00000007291
ENSODEG00000007292
ENSODEG00000007293
ENSODEG00000007295
ENSODEG00000007296
ENSODEG00000007297
ENSODEG00000007298
ENSODEG00000007300
ENSODEG00000007301
ENSODEG00000007303
ENSODEG00000007304
ENSODEG00000007305
ENSODEG00000007306
ENSODEG00000007307
ENSODEG00000007308
ENSODEG00000007309
ENSODEG00000007310
ENSODEG00000007311
ENSODEG00000007312
ENSODEG00000007313
ENSODEG00000007315
ENSODEG00000007317
ENSODEG00000007319
ENSODEG00000007320
ENSODEG00000007323
ENSODEG00000007324
ENSODEG00000007325
ENSODEG00000007326
ENSODEG00000007328
ENSODEG00000007329
ENSODEG00000007330
ENSODEG00000007331
ENSODEG00000007332
ENSODEG00000007334
ENSODEG00000007335
ENSODEG00000007337
ENSODEG00000007338
ENSODEG00000007341
ENSODEG00000007342
ENSODEG00000007343
ENSODEG00000007347
ENSODEG00000007348
ENSODEG00000007349
ENSODEG00000007350
ENSODEG00000007351
ENSODEG00000007352
ENSODEG00000007353
ENSODEG00000007354
ENSODEG00000007356
ENSODEG00000007357
ENSODEG00000007358
ENSODEG00000007359

ENSODEG00000007361
ENSODEG00000007362
ENSODEG00000007364
ENSODEG00000007366
ENSODEG00000007367
ENSODEG00000007368
ENSODEG00000007370
ENSODEG00000007374
ENSODEG00000007375
ENSODEG00000007377
ENSODEG00000007378
ENSODEG00000007379
ENSODEG00000007380
ENSODEG00000007383
ENSODEG00000007384
ENSODEG00000007386
ENSODEG00000007387
ENSODEG00000007388
ENSODEG00000007390
ENSODEG00000007391
ENSODEG00000007394
ENSODEG00000007395
ENSODEG00000007396
ENSODEG00000007397
ENSODEG00000007398
ENSODEG00000007400
ENSODEG00000007401
ENSODEG00000007402
ENSODEG00000007403
ENSODEG00000007404
ENSODEG00000007405
ENSODEG00000007406
ENSODEG00000007407
ENSODEG00000007408
ENSODEG00000007410
ENSODEG00000007411
ENSODEG00000007412
ENSODEG00000007413
ENSODEG00000007414
ENSODEG00000007415
ENSODEG00000007416
ENSODEG00000007417
ENSODEG00000007418
ENSODEG00000007419
ENSODEG00000007420
ENSODEG00000007421
ENSODEG00000007422
ENSODEG00000007424
ENSODEG00000007427
ENSODEG00000007429
ENSODEG00000007430
ENSODEG00000007431
ENSODEG00000007432
ENSODEG00000007433
ENSODEG00000007437
ENSODEG00000007438
ENSODEG00000007439
ENSODEG00000007440
ENSODEG00000007441

ENSODEG00000007443
ENSODEG00000007444
ENSODEG00000007446
ENSODEG00000007447
ENSODEG00000007448
ENSODEG00000007449
ENSODEG00000007451
ENSODEG00000007452
ENSODEG00000007453
ENSODEG00000007454
ENSODEG00000007455
ENSODEG00000007456
ENSODEG00000007457
ENSODEG00000007458
ENSODEG00000007459
ENSODEG00000007460
ENSODEG00000007462
ENSODEG00000007463
ENSODEG00000007464
ENSODEG00000007466
ENSODEG00000007469
ENSODEG00000007470
ENSODEG00000007471
ENSODEG00000007472
ENSODEG00000007473
ENSODEG00000007474
ENSODEG00000007475
ENSODEG00000007476
ENSODEG00000007477
ENSODEG00000007478
ENSODEG00000007479
ENSODEG00000007480
ENSODEG00000007481
ENSODEG00000007482
ENSODEG00000007484
ENSODEG00000007485
ENSODEG00000007486
ENSODEG00000007487
ENSODEG00000007490
ENSODEG00000007491
ENSODEG00000007492
ENSODEG00000007493
ENSODEG00000007495
ENSODEG00000007496
ENSODEG00000007500
ENSODEG00000007501
ENSODEG00000007503
ENSODEG00000007504
ENSODEG00000007506
ENSODEG00000007507
ENSODEG00000007508
ENSODEG00000007509
ENSODEG00000007510
ENSODEG00000007512
ENSODEG00000007514
ENSODEG00000007515
ENSODEG00000007516
ENSODEG00000007517
ENSODEG00000007518

ENSODEG00000007519
ENSODEG00000007520
ENSODEG00000007521
ENSODEG00000007522
ENSODEG00000007523
ENSODEG00000007524
ENSODEG00000007525
ENSODEG00000007526
ENSODEG00000007529
ENSODEG00000007532
ENSODEG00000007533
ENSODEG00000007536
ENSODEG00000007538
ENSODEG00000007539
ENSODEG00000007541
ENSODEG00000007542
ENSODEG00000007543
ENSODEG00000007545
ENSODEG00000007546
ENSODEG00000007547
ENSODEG00000007548
ENSODEG00000007549
ENSODEG00000007550
ENSODEG00000007551
ENSODEG00000007552
ENSODEG00000007553
ENSODEG00000007554
ENSODEG00000007555
ENSODEG00000007557
ENSODEG00000007559
ENSODEG00000007561
ENSODEG00000007563
ENSODEG00000007564
ENSODEG00000007565
ENSODEG00000007566
ENSODEG00000007568
ENSODEG00000007569
ENSODEG00000007570
ENSODEG00000007571
ENSODEG00000007572
ENSODEG00000007573
ENSODEG00000007575
ENSODEG00000007577
ENSODEG00000007578
ENSODEG00000007579
ENSODEG00000007582
ENSODEG00000007583
ENSODEG00000007584
ENSODEG00000007586
ENSODEG00000007587
ENSODEG00000007588
ENSODEG00000007589
ENSODEG00000007590
ENSODEG00000007591
ENSODEG00000007593
ENSODEG00000007594
ENSODEG00000007595
ENSODEG00000007596
ENSODEG00000007598

ENSODEG00000007599
ENSODEG00000007600
ENSODEG00000007601
ENSODEG00000007602
ENSODEG00000007603
ENSODEG00000007604
ENSODEG00000007606
ENSODEG00000007609
ENSODEG00000007611
ENSODEG00000007612
ENSODEG00000007613
ENSODEG00000007614
ENSODEG00000007616
ENSODEG00000007619
ENSODEG00000007620
ENSODEG00000007621
ENSODEG00000007623
ENSODEG00000007626
ENSODEG00000007627
ENSODEG00000007628
ENSODEG00000007629
ENSODEG00000007631
ENSODEG00000007632
ENSODEG00000007633
ENSODEG00000007634
ENSODEG00000007636
ENSODEG00000007637
ENSODEG00000007638
ENSODEG00000007639
ENSODEG00000007641
ENSODEG00000007642
ENSODEG00000007643
ENSODEG00000007644
ENSODEG00000007645
ENSODEG00000007647
ENSODEG00000007649
ENSODEG00000007650
ENSODEG00000007654
ENSODEG00000007655
ENSODEG00000007656
ENSODEG00000007660
ENSODEG00000007663
ENSODEG00000007664
ENSODEG00000007665
ENSODEG00000007666
ENSODEG00000007668
ENSODEG00000007669
ENSODEG00000007671
ENSODEG00000007673
ENSODEG00000007674
ENSODEG00000007676
ENSODEG00000007677
ENSODEG00000007678
ENSODEG00000007679
ENSODEG00000007681
ENSODEG00000007682
ENSODEG00000007683
ENSODEG00000007684
ENSODEG00000007687

ENSODEG00000007688
ENSODEG00000007689
ENSODEG00000007690
ENSODEG00000007691
ENSODEG00000007692
ENSODEG00000007694
ENSODEG00000007695
ENSODEG00000007696
ENSODEG00000007697
ENSODEG00000007698
ENSODEG00000007699
ENSODEG00000007700
ENSODEG00000007701
ENSODEG00000007703
ENSODEG00000007704
ENSODEG00000007705
ENSODEG00000007707
ENSODEG00000007708
ENSODEG00000007709
ENSODEG00000007710
ENSODEG00000007712
ENSODEG00000007713
ENSODEG00000007715
ENSODEG00000007717
ENSODEG00000007719
ENSODEG00000007720
ENSODEG00000007721
ENSODEG00000007722
ENSODEG00000007723
ENSODEG00000007724
ENSODEG00000007727
ENSODEG00000007728
ENSODEG00000007730
ENSODEG00000007731
ENSODEG00000007732
ENSODEG00000007733
ENSODEG00000007734
ENSODEG00000007736
ENSODEG00000007737
ENSODEG00000007740
ENSODEG00000007741
ENSODEG00000007743
ENSODEG00000007745
ENSODEG00000007747
ENSODEG00000007750
ENSODEG00000007752
ENSODEG00000007754
ENSODEG00000007755
ENSODEG00000007756
ENSODEG00000007757
ENSODEG00000007758
ENSODEG00000007759
ENSODEG00000007761
ENSODEG00000007762
ENSODEG00000007764
ENSODEG00000007767
ENSODEG00000007768
ENSODEG00000007769
ENSODEG00000007770

ENSODEG00000007771
ENSODEG00000007772
ENSODEG00000007775
ENSODEG00000007776
ENSODEG00000007778
ENSODEG00000007779
ENSODEG00000007780
ENSODEG00000007782
ENSODEG00000007783
ENSODEG00000007784
ENSODEG00000007785
ENSODEG00000007786
ENSODEG00000007787
ENSODEG00000007788
ENSODEG00000007789
ENSODEG00000007792
ENSODEG00000007795
ENSODEG00000007796
ENSODEG00000007798
ENSODEG00000007799
ENSODEG00000007800
ENSODEG00000007801
ENSODEG00000007802
ENSODEG00000007803
ENSODEG00000007804
ENSODEG00000007805
ENSODEG00000007806
ENSODEG00000007807
ENSODEG00000007811
ENSODEG00000007815
ENSODEG00000007816
ENSODEG00000007817
ENSODEG00000007818
ENSODEG00000007819
ENSODEG00000007820
ENSODEG00000007821
ENSODEG00000007822
ENSODEG00000007823
ENSODEG00000007824
ENSODEG00000007825
ENSODEG00000007826
ENSODEG00000007827
ENSODEG00000007828
ENSODEG00000007829
ENSODEG00000007830
ENSODEG00000007831
ENSODEG00000007833
ENSODEG00000007834
ENSODEG00000007836
ENSODEG00000007838
ENSODEG00000007841
ENSODEG00000007842
ENSODEG00000007843
ENSODEG00000007844
ENSODEG00000007846
ENSODEG00000007847
ENSODEG00000007849
ENSODEG00000007850
ENSODEG00000007851

ENSODEG00000007853
ENSODEG00000007856
ENSODEG00000007857
ENSODEG00000007858
ENSODEG00000007859
ENSODEG00000007860
ENSODEG00000007861
ENSODEG00000007862
ENSODEG00000007863
ENSODEG00000007864
ENSODEG00000007865
ENSODEG00000007866
ENSODEG00000007867
ENSODEG00000007868
ENSODEG00000007869
ENSODEG00000007870
ENSODEG00000007871
ENSODEG00000007872
ENSODEG00000007873
ENSODEG00000007874
ENSODEG00000007875
ENSODEG00000007877
ENSODEG00000007878
ENSODEG00000007879
ENSODEG00000007880
ENSODEG00000007882
ENSODEG00000007883
ENSODEG00000007884
ENSODEG00000007885
ENSODEG00000007886
ENSODEG00000007887
ENSODEG00000007890
ENSODEG00000007891
ENSODEG00000007892
ENSODEG00000007894
ENSODEG00000007895
ENSODEG00000007896
ENSODEG00000007897
ENSODEG00000007901
ENSODEG00000007903
ENSODEG00000007905
ENSODEG00000007906
ENSODEG00000007907
ENSODEG00000007908
ENSODEG00000007910
ENSODEG00000007912
ENSODEG00000007913
ENSODEG00000007914
ENSODEG00000007915
ENSODEG00000007918
ENSODEG00000007919
ENSODEG00000007920
ENSODEG00000007921
ENSODEG00000007922
ENSODEG00000007923
ENSODEG00000007924
ENSODEG00000007925
ENSODEG00000007927
ENSODEG00000007929

ENSODEG00000007930
ENSODEG00000007931
ENSODEG00000007933
ENSODEG00000007935
ENSODEG00000007939
ENSODEG00000007942
ENSODEG00000007946
ENSODEG00000007947
ENSODEG00000007949
ENSODEG00000007950
ENSODEG00000007951
ENSODEG00000007952
ENSODEG00000007953
ENSODEG00000007954
ENSODEG00000007955
ENSODEG00000007956
ENSODEG00000007958
ENSODEG00000007959
ENSODEG00000007960
ENSODEG00000007962
ENSODEG00000007963
ENSODEG00000007964
ENSODEG00000007965
ENSODEG00000007966
ENSODEG00000007967
ENSODEG00000007968
ENSODEG00000007969
ENSODEG00000007970
ENSODEG00000007972
ENSODEG00000007973
ENSODEG00000007975
ENSODEG00000007976
ENSODEG00000007977
ENSODEG00000007978
ENSODEG00000007980
ENSODEG00000007981
ENSODEG00000007982
ENSODEG00000007983
ENSODEG00000007985
ENSODEG00000007986
ENSODEG00000007987
ENSODEG00000007988
ENSODEG00000007989
ENSODEG00000007991
ENSODEG00000007992
ENSODEG00000007993
ENSODEG00000007994
ENSODEG00000007995
ENSODEG00000007996
ENSODEG00000007997
ENSODEG00000007998
ENSODEG00000007999
ENSODEG00000008000
ENSODEG00000008001
ENSODEG00000008002
ENSODEG00000008004
ENSODEG00000008006
ENSODEG00000008007
ENSODEG00000008008

ENSODEG00000008009
ENSODEG00000008011
ENSODEG00000008013
ENSODEG00000008015
ENSODEG00000008017
ENSODEG00000008018
ENSODEG00000008020
ENSODEG00000008021
ENSODEG00000008022
ENSODEG00000008024
ENSODEG00000008026
ENSODEG00000008028
ENSODEG00000008029
ENSODEG00000008030
ENSODEG00000008032
ENSODEG00000008033
ENSODEG00000008034
ENSODEG00000008035
ENSODEG00000008036
ENSODEG00000008038
ENSODEG00000008039
ENSODEG00000008040
ENSODEG00000008041
ENSODEG00000008042
ENSODEG00000008043
ENSODEG00000008046
ENSODEG00000008047
ENSODEG00000008050
ENSODEG00000008051
ENSODEG00000008052
ENSODEG00000008054
ENSODEG00000008055
ENSODEG00000008056
ENSODEG00000008057
ENSODEG00000008058
ENSODEG00000008059
ENSODEG00000008060
ENSODEG00000008063
ENSODEG00000008065
ENSODEG00000008066
ENSODEG00000008067
ENSODEG00000008068
ENSODEG00000008069
ENSODEG00000008070
ENSODEG00000008071
ENSODEG00000008072
ENSODEG00000008073
ENSODEG00000008074
ENSODEG00000008076
ENSODEG00000008077
ENSODEG00000008079
ENSODEG00000008080
ENSODEG00000008082
ENSODEG00000008083
ENSODEG00000008084
ENSODEG00000008085
ENSODEG00000008087
ENSODEG00000008088
ENSODEG00000008089

ENSODEG00000008091
ENSODEG00000008092
ENSODEG00000008094
ENSODEG00000008095
ENSODEG00000008097
ENSODEG00000008099
ENSODEG00000008102
ENSODEG00000008103
ENSODEG00000008104
ENSODEG00000008105
ENSODEG00000008106
ENSODEG00000008108
ENSODEG00000008109
ENSODEG00000008110
ENSODEG00000008112
ENSODEG00000008115
ENSODEG00000008116
ENSODEG00000008118
ENSODEG00000008119
ENSODEG00000008120
ENSODEG00000008122
ENSODEG00000008123
ENSODEG00000008124
ENSODEG00000008125
ENSODEG00000008127
ENSODEG00000008128
ENSODEG00000008131
ENSODEG00000008132
ENSODEG00000008133
ENSODEG00000008134
ENSODEG00000008135
ENSODEG00000008136
ENSODEG00000008137
ENSODEG00000008139
ENSODEG00000008140
ENSODEG00000008142
ENSODEG00000008143
ENSODEG00000008144
ENSODEG00000008145
ENSODEG00000008146
ENSODEG00000008148
ENSODEG00000008150
ENSODEG00000008152
ENSODEG00000008153
ENSODEG00000008154
ENSODEG00000008155
ENSODEG00000008158
ENSODEG00000008159
ENSODEG00000008160
ENSODEG00000008161
ENSODEG00000008162
ENSODEG00000008163
ENSODEG00000008164
ENSODEG00000008165
ENSODEG00000008166
ENSODEG00000008167
ENSODEG00000008168
ENSODEG00000008169
ENSODEG00000008170

ENSODEG00000008171
ENSODEG00000008172
ENSODEG00000008174
ENSODEG00000008175
ENSODEG00000008176
ENSODEG00000008177
ENSODEG00000008178
ENSODEG00000008179
ENSODEG00000008180
ENSODEG00000008181
ENSODEG00000008184
ENSODEG00000008186
ENSODEG00000008187
ENSODEG00000008188
ENSODEG00000008189
ENSODEG00000008191
ENSODEG00000008192
ENSODEG00000008193
ENSODEG00000008194
ENSODEG00000008195
ENSODEG00000008196
ENSODEG00000008198
ENSODEG00000008199
ENSODEG00000008200
ENSODEG00000008202
ENSODEG00000008206
ENSODEG00000008207
ENSODEG00000008208
ENSODEG00000008209
ENSODEG00000008210
ENSODEG00000008212
ENSODEG00000008215
ENSODEG00000008216
ENSODEG00000008221
ENSODEG00000008222
ENSODEG00000008224
ENSODEG00000008225
ENSODEG00000008226
ENSODEG00000008228
ENSODEG00000008229
ENSODEG00000008230
ENSODEG00000008232
ENSODEG00000008235
ENSODEG00000008236
ENSODEG00000008237
ENSODEG00000008238
ENSODEG00000008239
ENSODEG00000008240
ENSODEG00000008241
ENSODEG00000008242
ENSODEG00000008243
ENSODEG00000008245
ENSODEG00000008246
ENSODEG00000008247
ENSODEG00000008250
ENSODEG00000008251
ENSODEG00000008252
ENSODEG00000008253
ENSODEG00000008254

ENSODEG00000008255
ENSODEG00000008256
ENSODEG00000008257
ENSODEG00000008259
ENSODEG00000008263
ENSODEG00000008264
ENSODEG00000008266
ENSODEG00000008267
ENSODEG00000008269
ENSODEG00000008270
ENSODEG00000008271
ENSODEG00000008272
ENSODEG00000008274
ENSODEG00000008275
ENSODEG00000008277
ENSODEG00000008278
ENSODEG00000008281
ENSODEG00000008282
ENSODEG00000008283
ENSODEG00000008286
ENSODEG00000008287
ENSODEG00000008288
ENSODEG00000008289
ENSODEG00000008292
ENSODEG00000008294
ENSODEG00000008295
ENSODEG00000008296
ENSODEG00000008297
ENSODEG00000008298
ENSODEG00000008299
ENSODEG00000008300
ENSODEG00000008301
ENSODEG00000008302
ENSODEG00000008303
ENSODEG00000008305
ENSODEG00000008307
ENSODEG00000008308
ENSODEG00000008309
ENSODEG00000008310
ENSODEG00000008311
ENSODEG00000008313
ENSODEG00000008314
ENSODEG00000008316
ENSODEG00000008317
ENSODEG00000008318
ENSODEG00000008319
ENSODEG00000008320
ENSODEG00000008323
ENSODEG00000008324
ENSODEG00000008325
ENSODEG00000008326
ENSODEG00000008327
ENSODEG00000008329
ENSODEG00000008330
ENSODEG00000008332
ENSODEG00000008335
ENSODEG00000008337
ENSODEG00000008338
ENSODEG00000008339

ENSODEG00000008340
ENSODEG00000008341
ENSODEG00000008342
ENSODEG00000008343
ENSODEG00000008344
ENSODEG00000008347
ENSODEG00000008348
ENSODEG00000008349
ENSODEG00000008350
ENSODEG00000008352
ENSODEG00000008353
ENSODEG00000008354
ENSODEG00000008355
ENSODEG00000008356
ENSODEG00000008357
ENSODEG00000008358
ENSODEG00000008359
ENSODEG00000008360
ENSODEG00000008361
ENSODEG00000008362
ENSODEG00000008363
ENSODEG00000008364
ENSODEG00000008365
ENSODEG00000008367
ENSODEG00000008368
ENSODEG00000008369
ENSODEG00000008370
ENSODEG00000008371
ENSODEG00000008372
ENSODEG00000008373
ENSODEG00000008375
ENSODEG00000008376
ENSODEG00000008378
ENSODEG00000008382
ENSODEG00000008384
ENSODEG00000008385
ENSODEG00000008387
ENSODEG00000008390
ENSODEG00000008392
ENSODEG00000008393
ENSODEG00000008396
ENSODEG00000008399
ENSODEG00000008400
ENSODEG00000008401
ENSODEG00000008402
ENSODEG00000008404
ENSODEG00000008408
ENSODEG00000008410
ENSODEG00000008411
ENSODEG00000008417
ENSODEG00000008418
ENSODEG00000008420
ENSODEG00000008422
ENSODEG00000008423
ENSODEG00000008425
ENSODEG00000008426
ENSODEG00000008428
ENSODEG00000008429
ENSODEG00000008430

ENSODEG00000008431
ENSODEG00000008435
ENSODEG00000008437
ENSODEG00000008441
ENSODEG00000008442
ENSODEG00000008443
ENSODEG00000008444
ENSODEG00000008445
ENSODEG00000008447
ENSODEG00000008448
ENSODEG00000008450
ENSODEG00000008451
ENSODEG00000008452
ENSODEG00000008453
ENSODEG00000008454
ENSODEG00000008456
ENSODEG00000008457
ENSODEG00000008458
ENSODEG00000008460
ENSODEG00000008461
ENSODEG00000008462
ENSODEG00000008463
ENSODEG00000008464
ENSODEG00000008466
ENSODEG00000008467
ENSODEG00000008469
ENSODEG00000008470
ENSODEG00000008471
ENSODEG00000008472
ENSODEG00000008473
ENSODEG00000008475
ENSODEG00000008477
ENSODEG00000008478
ENSODEG00000008480
ENSODEG00000008481
ENSODEG00000008482
ENSODEG00000008483
ENSODEG00000008484
ENSODEG00000008487
ENSODEG00000008488
ENSODEG00000008489
ENSODEG00000008490
ENSODEG00000008491
ENSODEG00000008492
ENSODEG00000008493
ENSODEG00000008494
ENSODEG00000008495
ENSODEG00000008496
ENSODEG00000008497
ENSODEG00000008498
ENSODEG00000008499
ENSODEG00000008500
ENSODEG00000008501
ENSODEG00000008502
ENSODEG00000008503
ENSODEG00000008504
ENSODEG00000008505
ENSODEG00000008506
ENSODEG00000008507

ENSODEG00000008508
ENSODEG00000008509
ENSODEG00000008510
ENSODEG00000008511
ENSODEG00000008512
ENSODEG00000008513
ENSODEG00000008514
ENSODEG00000008515
ENSODEG00000008516
ENSODEG00000008517
ENSODEG00000008518
ENSODEG00000008519
ENSODEG00000008521
ENSODEG00000008522
ENSODEG00000008523
ENSODEG00000008524
ENSODEG00000008525
ENSODEG00000008526
ENSODEG00000008528
ENSODEG00000008529
ENSODEG00000008532
ENSODEG00000008533
ENSODEG00000008534
ENSODEG00000008535
ENSODEG00000008536
ENSODEG00000008539
ENSODEG00000008543
ENSODEG00000008544
ENSODEG00000008546
ENSODEG00000008547
ENSODEG00000008548
ENSODEG00000008551
ENSODEG00000008552
ENSODEG00000008553
ENSODEG00000008556
ENSODEG00000008558
ENSODEG00000008559
ENSODEG00000008560
ENSODEG00000008561
ENSODEG00000008562
ENSODEG00000008563
ENSODEG00000008564
ENSODEG00000008565
ENSODEG00000008566
ENSODEG00000008567
ENSODEG00000008568
ENSODEG00000008569
ENSODEG00000008570
ENSODEG00000008572
ENSODEG00000008574
ENSODEG00000008576
ENSODEG00000008577
ENSODEG00000008579
ENSODEG00000008580
ENSODEG00000008582
ENSODEG00000008583
ENSODEG00000008584
ENSODEG00000008586
ENSODEG00000008587

ENSODEG00000008588
ENSODEG00000008591
ENSODEG00000008593
ENSODEG00000008594
ENSODEG00000008596
ENSODEG00000008597
ENSODEG00000008598
ENSODEG00000008599
ENSODEG00000008601
ENSODEG00000008606
ENSODEG00000008607
ENSODEG00000008608
ENSODEG00000008609
ENSODEG00000008610
ENSODEG00000008611
ENSODEG00000008612
ENSODEG00000008614
ENSODEG00000008615
ENSODEG00000008617
ENSODEG00000008618
ENSODEG00000008619
ENSODEG00000008621
ENSODEG00000008623
ENSODEG00000008624
ENSODEG00000008625
ENSODEG00000008628
ENSODEG00000008629
ENSODEG00000008630
ENSODEG00000008631
ENSODEG00000008633
ENSODEG00000008637
ENSODEG00000008642
ENSODEG00000008643
ENSODEG00000008644
ENSODEG00000008645
ENSODEG00000008646
ENSODEG00000008647
ENSODEG00000008648
ENSODEG00000008649
ENSODEG00000008650
ENSODEG00000008651
ENSODEG00000008653
ENSODEG00000008654
ENSODEG00000008655
ENSODEG00000008656
ENSODEG00000008657
ENSODEG00000008658
ENSODEG00000008659
ENSODEG00000008660
ENSODEG00000008661
ENSODEG00000008662
ENSODEG00000008663
ENSODEG00000008664
ENSODEG00000008665
ENSODEG00000008666
ENSODEG00000008667
ENSODEG00000008670
ENSODEG00000008671
ENSODEG00000008673

ENSODEG00000008674
ENSODEG00000008675
ENSODEG00000008676
ENSODEG00000008677
ENSODEG00000008678
ENSODEG00000008680
ENSODEG00000008681
ENSODEG00000008683
ENSODEG00000008684
ENSODEG00000008685
ENSODEG00000008686
ENSODEG00000008688
ENSODEG00000008689
ENSODEG00000008690
ENSODEG00000008691
ENSODEG00000008692
ENSODEG00000008693
ENSODEG00000008694
ENSODEG00000008696
ENSODEG00000008697
ENSODEG00000008698
ENSODEG00000008700
ENSODEG00000008701
ENSODEG00000008703
ENSODEG00000008704
ENSODEG00000008705
ENSODEG00000008708
ENSODEG00000008709
ENSODEG00000008710
ENSODEG00000008711
ENSODEG00000008712
ENSODEG00000008713
ENSODEG00000008715
ENSODEG00000008716
ENSODEG00000008718
ENSODEG00000008719
ENSODEG00000008722
ENSODEG00000008724
ENSODEG00000008725
ENSODEG00000008726
ENSODEG00000008728
ENSODEG00000008729
ENSODEG00000008730
ENSODEG00000008731
ENSODEG00000008732
ENSODEG00000008733
ENSODEG00000008734
ENSODEG00000008735
ENSODEG00000008737
ENSODEG00000008738
ENSODEG00000008740
ENSODEG00000008741
ENSODEG00000008742
ENSODEG00000008743
ENSODEG00000008745
ENSODEG00000008746
ENSODEG00000008748
ENSODEG00000008749
ENSODEG00000008750

ENSODEG00000008751
ENSODEG00000008752
ENSODEG00000008753
ENSODEG00000008754
ENSODEG00000008755
ENSODEG00000008756
ENSODEG00000008757
ENSODEG00000008758
ENSODEG00000008759
ENSODEG00000008760
ENSODEG00000008763
ENSODEG00000008764
ENSODEG00000008765
ENSODEG00000008768
ENSODEG00000008769
ENSODEG00000008770
ENSODEG00000008771
ENSODEG00000008773
ENSODEG00000008774
ENSODEG00000008775
ENSODEG00000008778
ENSODEG00000008780
ENSODEG00000008782
ENSODEG00000008786
ENSODEG00000008787
ENSODEG00000008788
ENSODEG00000008789
ENSODEG00000008790
ENSODEG00000008792
ENSODEG00000008794
ENSODEG00000008795
ENSODEG00000008798
ENSODEG00000008799
ENSODEG00000008800
ENSODEG00000008801
ENSODEG00000008802
ENSODEG00000008803
ENSODEG00000008806
ENSODEG00000008807
ENSODEG00000008808
ENSODEG00000008809
ENSODEG00000008810
ENSODEG00000008812
ENSODEG00000008815
ENSODEG00000008817
ENSODEG00000008818
ENSODEG00000008819
ENSODEG00000008820
ENSODEG00000008823
ENSODEG00000008824
ENSODEG00000008825
ENSODEG00000008826
ENSODEG00000008828
ENSODEG00000008829
ENSODEG00000008832
ENSODEG00000008833
ENSODEG00000008835
ENSODEG00000008837
ENSODEG00000008838

ENSODEG00000008840
ENSODEG00000008841
ENSODEG00000008845
ENSODEG00000008847
ENSODEG00000008848
ENSODEG00000008849
ENSODEG00000008850
ENSODEG00000008851
ENSODEG00000008852
ENSODEG00000008853
ENSODEG00000008854
ENSODEG00000008856
ENSODEG00000008857
ENSODEG00000008858
ENSODEG00000008859
ENSODEG00000008860
ENSODEG00000008861
ENSODEG00000008862
ENSODEG00000008863
ENSODEG00000008864
ENSODEG00000008865
ENSODEG00000008866
ENSODEG00000008867
ENSODEG00000008869
ENSODEG00000008870
ENSODEG00000008872
ENSODEG00000008873
ENSODEG00000008875
ENSODEG00000008877
ENSODEG00000008879
ENSODEG00000008881
ENSODEG00000008882
ENSODEG00000008883
ENSODEG00000008885
ENSODEG00000008886
ENSODEG00000008887
ENSODEG00000008888
ENSODEG00000008890
ENSODEG00000008891
ENSODEG00000008893
ENSODEG00000008894
ENSODEG00000008895
ENSODEG00000008896
ENSODEG00000008897
ENSODEG00000008898
ENSODEG00000008900
ENSODEG00000008901
ENSODEG00000008902
ENSODEG00000008903
ENSODEG00000008907
ENSODEG00000008908
ENSODEG00000008909
ENSODEG00000008910
ENSODEG00000008911
ENSODEG00000008912
ENSODEG00000008913
ENSODEG00000008914
ENSODEG00000008915
ENSODEG00000008916

ENSODEG00000008917
ENSODEG00000008918
ENSODEG00000008919
ENSODEG00000008920
ENSODEG00000008922
ENSODEG00000008923
ENSODEG00000008925
ENSODEG00000008926
ENSODEG00000008927
ENSODEG00000008928
ENSODEG00000008931
ENSODEG00000008932
ENSODEG00000008933
ENSODEG00000008935
ENSODEG00000008936
ENSODEG00000008937
ENSODEG00000008940
ENSODEG00000008941
ENSODEG00000008942
ENSODEG00000008945
ENSODEG00000008946
ENSODEG00000008947
ENSODEG00000008948
ENSODEG00000008949
ENSODEG00000008950
ENSODEG00000008951
ENSODEG00000008952
ENSODEG00000008953
ENSODEG00000008955
ENSODEG00000008956
ENSODEG00000008957
ENSODEG00000008958
ENSODEG00000008959
ENSODEG00000008960
ENSODEG00000008961
ENSODEG00000008963
ENSODEG00000008964
ENSODEG00000008965
ENSODEG00000008966
ENSODEG00000008967
ENSODEG00000008968
ENSODEG00000008969
ENSODEG00000008971
ENSODEG00000008972
ENSODEG00000008973
ENSODEG00000008974
ENSODEG00000008980
ENSODEG00000008981
ENSODEG00000008982
ENSODEG00000008983
ENSODEG00000008984
ENSODEG00000008985
ENSODEG00000008987
ENSODEG00000008988
ENSODEG00000008989
ENSODEG00000008991
ENSODEG00000008992
ENSODEG00000008993
ENSODEG00000008994

ENSODEG00000008995
ENSODEG00000008996
ENSODEG00000008997
ENSODEG00000008998
ENSODEG00000008999
ENSODEG00000009002
ENSODEG00000009003
ENSODEG00000009004
ENSODEG00000009006
ENSODEG00000009007
ENSODEG00000009008
ENSODEG00000009010
ENSODEG00000009013
ENSODEG00000009014
ENSODEG00000009016
ENSODEG00000009018
ENSODEG00000009019
ENSODEG00000009020
ENSODEG00000009021
ENSODEG00000009022
ENSODEG00000009023
ENSODEG00000009024
ENSODEG00000009026
ENSODEG00000009027
ENSODEG00000009028
ENSODEG00000009029
ENSODEG00000009031
ENSODEG00000009033
ENSODEG00000009034
ENSODEG00000009035
ENSODEG00000009036
ENSODEG00000009038
ENSODEG00000009041
ENSODEG00000009043
ENSODEG00000009045
ENSODEG00000009047
ENSODEG00000009048
ENSODEG00000009049
ENSODEG00000009050
ENSODEG00000009051
ENSODEG00000009052
ENSODEG00000009055
ENSODEG00000009056
ENSODEG00000009057
ENSODEG00000009058
ENSODEG00000009059
ENSODEG00000009060
ENSODEG00000009061
ENSODEG00000009062
ENSODEG00000009064
ENSODEG00000009066
ENSODEG00000009067
ENSODEG00000009068
ENSODEG00000009069
ENSODEG00000009073
ENSODEG00000009074
ENSODEG00000009075
ENSODEG00000009078
ENSODEG00000009079

ENSODEG00000009080
ENSODEG00000009082
ENSODEG00000009083
ENSODEG00000009084
ENSODEG00000009085
ENSODEG00000009086
ENSODEG00000009088
ENSODEG00000009091
ENSODEG00000009092
ENSODEG00000009093
ENSODEG00000009094
ENSODEG00000009095
ENSODEG00000009097
ENSODEG00000009098
ENSODEG00000009099
ENSODEG00000009101
ENSODEG00000009102
ENSODEG00000009103
ENSODEG00000009106
ENSODEG00000009107
ENSODEG00000009108
ENSODEG00000009109
ENSODEG00000009110
ENSODEG00000009111
ENSODEG00000009112
ENSODEG00000009113
ENSODEG00000009114
ENSODEG00000009115
ENSODEG00000009116
ENSODEG00000009117
ENSODEG00000009118
ENSODEG00000009121
ENSODEG00000009122
ENSODEG00000009123
ENSODEG00000009124
ENSODEG00000009125
ENSODEG00000009126
ENSODEG00000009127
ENSODEG00000009128
ENSODEG00000009129
ENSODEG00000009130
ENSODEG00000009131
ENSODEG00000009132
ENSODEG00000009133
ENSODEG00000009135
ENSODEG00000009136
ENSODEG00000009137
ENSODEG00000009138
ENSODEG00000009139
ENSODEG00000009140
ENSODEG00000009142
ENSODEG00000009144
ENSODEG00000009146
ENSODEG00000009148
ENSODEG00000009149
ENSODEG00000009152
ENSODEG00000009153
ENSODEG00000009154
ENSODEG00000009157

ENSODEG00000009159
ENSODEG00000009163
ENSODEG00000009165
ENSODEG00000009166
ENSODEG00000009168
ENSODEG00000009170
ENSODEG00000009171
ENSODEG00000009173
ENSODEG00000009174
ENSODEG00000009175
ENSODEG00000009176
ENSODEG00000009177
ENSODEG00000009178
ENSODEG00000009180
ENSODEG00000009181
ENSODEG00000009182
ENSODEG00000009184
ENSODEG00000009185
ENSODEG00000009186
ENSODEG00000009189
ENSODEG00000009191
ENSODEG00000009192
ENSODEG00000009193
ENSODEG00000009194
ENSODEG00000009195
ENSODEG00000009196
ENSODEG00000009197
ENSODEG00000009199
ENSODEG00000009200
ENSODEG00000009201
ENSODEG00000009202
ENSODEG00000009203
ENSODEG00000009204
ENSODEG00000009205
ENSODEG00000009207
ENSODEG00000009208
ENSODEG00000009209
ENSODEG00000009210
ENSODEG00000009211
ENSODEG00000009212
ENSODEG00000009213
ENSODEG00000009214
ENSODEG00000009216
ENSODEG00000009218
ENSODEG00000009221
ENSODEG00000009222
ENSODEG00000009225
ENSODEG00000009226
ENSODEG00000009227
ENSODEG00000009228
ENSODEG00000009229
ENSODEG00000009232
ENSODEG00000009233
ENSODEG00000009235
ENSODEG00000009236
ENSODEG00000009237
ENSODEG00000009241
ENSODEG00000009242
ENSODEG00000009243

ENSODEG00000009245
ENSODEG00000009247
ENSODEG00000009248
ENSODEG00000009250
ENSODEG00000009251
ENSODEG00000009252
ENSODEG00000009253
ENSODEG00000009254
ENSODEG00000009256
ENSODEG00000009257
ENSODEG00000009259
ENSODEG00000009260
ENSODEG00000009261
ENSODEG00000009262
ENSODEG00000009263
ENSODEG00000009264
ENSODEG00000009265
ENSODEG00000009266
ENSODEG00000009267
ENSODEG00000009268
ENSODEG00000009270
ENSODEG00000009271
ENSODEG00000009273
ENSODEG00000009275
ENSODEG00000009276
ENSODEG00000009277
ENSODEG00000009278
ENSODEG00000009279
ENSODEG00000009280
ENSODEG00000009282
ENSODEG00000009283
ENSODEG00000009285
ENSODEG00000009286
ENSODEG00000009289
ENSODEG00000009290
ENSODEG00000009292
ENSODEG00000009294
ENSODEG00000009297
ENSODEG00000009298
ENSODEG00000009299
ENSODEG00000009301
ENSODEG00000009302
ENSODEG00000009303
ENSODEG00000009307
ENSODEG00000009310
ENSODEG00000009311
ENSODEG00000009312
ENSODEG00000009313
ENSODEG00000009314
ENSODEG00000009316
ENSODEG00000009317
ENSODEG00000009318
ENSODEG00000009319
ENSODEG00000009320
ENSODEG00000009322
ENSODEG00000009323
ENSODEG00000009324
ENSODEG00000009325
ENSODEG00000009326

ENSODEG00000009327
ENSODEG00000009328
ENSODEG00000009329
ENSODEG00000009330
ENSODEG00000009331
ENSODEG00000009332
ENSODEG00000009333
ENSODEG00000009334
ENSODEG00000009335
ENSODEG00000009336
ENSODEG00000009337
ENSODEG00000009338
ENSODEG00000009339
ENSODEG00000009340
ENSODEG00000009341
ENSODEG00000009342
ENSODEG00000009343
ENSODEG00000009344
ENSODEG00000009345
ENSODEG00000009351
ENSODEG00000009352
ENSODEG00000009353
ENSODEG00000009354
ENSODEG00000009355
ENSODEG00000009356
ENSODEG00000009357
ENSODEG00000009358
ENSODEG00000009359
ENSODEG00000009360
ENSODEG00000009361
ENSODEG00000009364
ENSODEG00000009366
ENSODEG00000009367
ENSODEG00000009368
ENSODEG00000009370
ENSODEG00000009373
ENSODEG00000009374
ENSODEG00000009375
ENSODEG00000009376
ENSODEG00000009377
ENSODEG00000009378
ENSODEG00000009380
ENSODEG00000009383
ENSODEG00000009384
ENSODEG00000009385
ENSODEG00000009386
ENSODEG00000009387
ENSODEG00000009391
ENSODEG00000009392
ENSODEG00000009394
ENSODEG00000009399
ENSODEG00000009400
ENSODEG00000009401
ENSODEG00000009402
ENSODEG00000009403
ENSODEG00000009404
ENSODEG00000009405
ENSODEG00000009406
ENSODEG00000009407

ENSODEG00000009408
ENSODEG00000009410
ENSODEG00000009411
ENSODEG00000009412
ENSODEG00000009413
ENSODEG00000009416
ENSODEG00000009417
ENSODEG00000009418
ENSODEG00000009419
ENSODEG00000009420
ENSODEG00000009422
ENSODEG00000009423
ENSODEG00000009424
ENSODEG00000009425
ENSODEG00000009427
ENSODEG00000009428
ENSODEG00000009429
ENSODEG00000009430
ENSODEG00000009431
ENSODEG00000009435
ENSODEG00000009437
ENSODEG00000009439
ENSODEG00000009440
ENSODEG00000009442
ENSODEG00000009443
ENSODEG00000009444
ENSODEG00000009445
ENSODEG00000009446
ENSODEG00000009447
ENSODEG00000009448
ENSODEG00000009451
ENSODEG00000009452
ENSODEG00000009453
ENSODEG00000009454
ENSODEG00000009455
ENSODEG00000009456
ENSODEG00000009458
ENSODEG00000009459
ENSODEG00000009460
ENSODEG00000009462
ENSODEG00000009463
ENSODEG00000009464
ENSODEG00000009465
ENSODEG00000009466
ENSODEG00000009468
ENSODEG00000009469
ENSODEG00000009471
ENSODEG00000009472
ENSODEG00000009474
ENSODEG00000009477
ENSODEG00000009479
ENSODEG00000009480
ENSODEG00000009481
ENSODEG00000009483
ENSODEG00000009484
ENSODEG00000009486
ENSODEG00000009487
ENSODEG00000009488
ENSODEG00000009489

ENSODEG00000009492
ENSODEG00000009494
ENSODEG00000009495
ENSODEG00000009496
ENSODEG00000009498
ENSODEG00000009500
ENSODEG00000009501
ENSODEG00000009502
ENSODEG00000009503
ENSODEG00000009504
ENSODEG00000009505
ENSODEG00000009506
ENSODEG00000009507
ENSODEG00000009508
ENSODEG00000009509
ENSODEG00000009511
ENSODEG00000009512
ENSODEG00000009513
ENSODEG00000009515
ENSODEG00000009517
ENSODEG00000009519
ENSODEG00000009520
ENSODEG00000009521
ENSODEG00000009522
ENSODEG00000009523
ENSODEG00000009525
ENSODEG00000009526
ENSODEG00000009527
ENSODEG00000009528
ENSODEG00000009531
ENSODEG00000009532
ENSODEG00000009534
ENSODEG00000009536
ENSODEG00000009537
ENSODEG00000009538
ENSODEG00000009540
ENSODEG00000009541
ENSODEG00000009544
ENSODEG00000009546
ENSODEG00000009547
ENSODEG00000009548
ENSODEG00000009549
ENSODEG00000009551
ENSODEG00000009553
ENSODEG00000009556
ENSODEG00000009557
ENSODEG00000009558
ENSODEG00000009559
ENSODEG00000009560
ENSODEG00000009562
ENSODEG00000009563
ENSODEG00000009564
ENSODEG00000009566
ENSODEG00000009569
ENSODEG00000009571
ENSODEG00000009573
ENSODEG00000009574
ENSODEG00000009576
ENSODEG00000009577

ENSODEG00000009578
ENSODEG00000009582
ENSODEG00000009584
ENSODEG00000009585
ENSODEG00000009586
ENSODEG00000009587
ENSODEG00000009589
ENSODEG00000009591
ENSODEG00000009593
ENSODEG00000009595
ENSODEG00000009598
ENSODEG00000009600
ENSODEG00000009602
ENSODEG00000009603
ENSODEG00000009605
ENSODEG00000009606
ENSODEG00000009607
ENSODEG00000009608
ENSODEG00000009610
ENSODEG00000009611
ENSODEG00000009612
ENSODEG00000009613
ENSODEG00000009615
ENSODEG00000009617
ENSODEG00000009618
ENSODEG00000009619
ENSODEG00000009620
ENSODEG00000009622
ENSODEG00000009627
ENSODEG00000009628
ENSODEG00000009629
ENSODEG00000009630
ENSODEG00000009631
ENSODEG00000009632
ENSODEG00000009633
ENSODEG00000009634
ENSODEG00000009635
ENSODEG00000009637
ENSODEG00000009641
ENSODEG00000009642
ENSODEG00000009644
ENSODEG00000009647
ENSODEG00000009648
ENSODEG00000009649
ENSODEG00000009650
ENSODEG00000009651
ENSODEG00000009652
ENSODEG00000009653
ENSODEG00000009654
ENSODEG00000009656
ENSODEG00000009657
ENSODEG00000009658
ENSODEG00000009659
ENSODEG00000009660
ENSODEG00000009661
ENSODEG00000009662
ENSODEG00000009665
ENSODEG00000009666
ENSODEG00000009667

ENSODEG00000009669
ENSODEG00000009671
ENSODEG00000009672
ENSODEG00000009673
ENSODEG00000009674
ENSODEG00000009677
ENSODEG00000009678
ENSODEG00000009679
ENSODEG00000009680
ENSODEG00000009681
ENSODEG00000009684
ENSODEG00000009685
ENSODEG00000009686
ENSODEG00000009687
ENSODEG00000009689
ENSODEG00000009691
ENSODEG00000009692
ENSODEG00000009693
ENSODEG00000009694
ENSODEG00000009695
ENSODEG00000009697
ENSODEG00000009698
ENSODEG00000009699
ENSODEG00000009702
ENSODEG00000009703
ENSODEG00000009705
ENSODEG00000009706
ENSODEG00000009707
ENSODEG00000009708
ENSODEG00000009709
ENSODEG00000009711
ENSODEG00000009712
ENSODEG00000009713
ENSODEG00000009714
ENSODEG00000009715
ENSODEG00000009717
ENSODEG00000009718
ENSODEG00000009719
ENSODEG00000009720
ENSODEG00000009721
ENSODEG00000009724
ENSODEG00000009725
ENSODEG00000009726
ENSODEG00000009727
ENSODEG00000009730
ENSODEG00000009732
ENSODEG00000009734
ENSODEG00000009737
ENSODEG00000009739
ENSODEG00000009740
ENSODEG00000009741
ENSODEG00000009742
ENSODEG00000009743
ENSODEG00000009744
ENSODEG00000009745
ENSODEG00000009746
ENSODEG00000009747
ENSODEG00000009748
ENSODEG00000009749

ENSODEG00000009750
ENSODEG00000009752
ENSODEG00000009753
ENSODEG00000009754
ENSODEG00000009756
ENSODEG00000009757
ENSODEG00000009760
ENSODEG00000009762
ENSODEG00000009763
ENSODEG00000009766
ENSODEG00000009767
ENSODEG00000009768
ENSODEG00000009769
ENSODEG00000009770
ENSODEG00000009771
ENSODEG00000009772
ENSODEG00000009773
ENSODEG00000009774
ENSODEG00000009775
ENSODEG00000009776
ENSODEG00000009778
ENSODEG00000009779
ENSODEG00000009780
ENSODEG00000009781
ENSODEG00000009782
ENSODEG00000009784
ENSODEG00000009785
ENSODEG00000009786
ENSODEG00000009788
ENSODEG00000009791
ENSODEG00000009792
ENSODEG00000009793
ENSODEG00000009794
ENSODEG00000009795
ENSODEG00000009796
ENSODEG00000009800
ENSODEG00000009801
ENSODEG00000009802
ENSODEG00000009804
ENSODEG00000009805
ENSODEG00000009806
ENSODEG00000009808
ENSODEG00000009809
ENSODEG00000009810
ENSODEG00000009811
ENSODEG00000009812
ENSODEG00000009813
ENSODEG00000009814
ENSODEG00000009816
ENSODEG00000009817
ENSODEG00000009818
ENSODEG00000009819
ENSODEG00000009820
ENSODEG00000009823
ENSODEG00000009824
ENSODEG00000009825
ENSODEG00000009826
ENSODEG00000009827
ENSODEG00000009828

ENSODEG00000009829
ENSODEG00000009831
ENSODEG00000009832
ENSODEG00000009833
ENSODEG00000009836
ENSODEG00000009839
ENSODEG00000009843
ENSODEG00000009844
ENSODEG00000009845
ENSODEG00000009846
ENSODEG00000009847
ENSODEG00000009848
ENSODEG00000009849
ENSODEG00000009850
ENSODEG00000009851
ENSODEG00000009853
ENSODEG00000009854
ENSODEG00000009856
ENSODEG00000009857
ENSODEG00000009859
ENSODEG00000009861
ENSODEG00000009862
ENSODEG00000009863
ENSODEG00000009864
ENSODEG00000009866
ENSODEG00000009868
ENSODEG00000009871
ENSODEG00000009872
ENSODEG00000009873
ENSODEG00000009874
ENSODEG00000009875
ENSODEG00000009876
ENSODEG00000009879
ENSODEG00000009880
ENSODEG00000009881
ENSODEG00000009882
ENSODEG00000009883
ENSODEG00000009884
ENSODEG00000009885
ENSODEG00000009886
ENSODEG00000009887
ENSODEG00000009888
ENSODEG00000009889
ENSODEG00000009890
ENSODEG00000009891
ENSODEG00000009892
ENSODEG00000009893
ENSODEG00000009894
ENSODEG00000009895
ENSODEG00000009896
ENSODEG00000009897
ENSODEG00000009898
ENSODEG00000009899
ENSODEG00000009900
ENSODEG00000009901
ENSODEG00000009902
ENSODEG00000009903
ENSODEG00000009906
ENSODEG00000009907

ENSODEG00000009913
ENSODEG00000009914
ENSODEG00000009915
ENSODEG00000009916
ENSODEG00000009917
ENSODEG00000009919
ENSODEG00000009920
ENSODEG00000009922
ENSODEG00000009924
ENSODEG00000009925
ENSODEG00000009926
ENSODEG00000009927
ENSODEG00000009929
ENSODEG00000009930
ENSODEG00000009932
ENSODEG00000009933
ENSODEG00000009934
ENSODEG00000009935
ENSODEG00000009936
ENSODEG00000009937
ENSODEG00000009939
ENSODEG00000009940
ENSODEG00000009941
ENSODEG00000009947
ENSODEG00000009948
ENSODEG00000009949
ENSODEG00000009950
ENSODEG00000009951
ENSODEG00000009954
ENSODEG00000009955
ENSODEG00000009956
ENSODEG00000009958
ENSODEG00000009961
ENSODEG00000009964
ENSODEG00000009967
ENSODEG00000009968
ENSODEG00000009970
ENSODEG00000009971
ENSODEG00000009972
ENSODEG00000009973
ENSODEG00000009974
ENSODEG00000009975
ENSODEG00000009976
ENSODEG00000009977
ENSODEG00000009978
ENSODEG00000009979
ENSODEG00000009980
ENSODEG00000009983
ENSODEG00000009984
ENSODEG00000009985
ENSODEG00000009986
ENSODEG00000009987
ENSODEG00000009988
ENSODEG00000009991
ENSODEG00000009992
ENSODEG00000009993
ENSODEG00000009995
ENSODEG00000009996
ENSODEG00000009998

ENSODEG00000010001
ENSODEG00000010002
ENSODEG00000010003
ENSODEG00000010004
ENSODEG00000010005
ENSODEG00000010006
ENSODEG00000010008
ENSODEG00000010009
ENSODEG00000010010
ENSODEG00000010011
ENSODEG00000010012
ENSODEG00000010013
ENSODEG00000010017
ENSODEG00000010018
ENSODEG00000010020
ENSODEG00000010021
ENSODEG00000010025
ENSODEG00000010026
ENSODEG00000010027
ENSODEG00000010028
ENSODEG00000010030
ENSODEG00000010033
ENSODEG00000010035
ENSODEG00000010036
ENSODEG00000010037
ENSODEG00000010038
ENSODEG00000010039
ENSODEG00000010040
ENSODEG00000010041
ENSODEG00000010042
ENSODEG00000010043
ENSODEG00000010044
ENSODEG00000010046
ENSODEG00000010047
ENSODEG00000010048
ENSODEG00000010050
ENSODEG00000010051
ENSODEG00000010054
ENSODEG00000010055
ENSODEG00000010057
ENSODEG00000010058
ENSODEG00000010059
ENSODEG00000010061
ENSODEG00000010062
ENSODEG00000010063
ENSODEG00000010064
ENSODEG00000010066
ENSODEG00000010067
ENSODEG00000010068
ENSODEG00000010069
ENSODEG00000010070
ENSODEG00000010071
ENSODEG00000010073
ENSODEG00000010074
ENSODEG00000010075
ENSODEG00000010076
ENSODEG00000010077
ENSODEG00000010078
ENSODEG00000010080

ENSODEG00000010082
ENSODEG00000010083
ENSODEG00000010085
ENSODEG00000010086
ENSODEG00000010087
ENSODEG00000010091
ENSODEG00000010092
ENSODEG00000010093
ENSODEG00000010094
ENSODEG00000010095
ENSODEG00000010096
ENSODEG00000010097
ENSODEG00000010098
ENSODEG00000010101
ENSODEG00000010102
ENSODEG00000010103
ENSODEG00000010105
ENSODEG00000010106
ENSODEG00000010107
ENSODEG00000010108
ENSODEG00000010110
ENSODEG00000010111
ENSODEG00000010112
ENSODEG00000010114
ENSODEG00000010115
ENSODEG00000010116
ENSODEG00000010117
ENSODEG00000010118
ENSODEG00000010119
ENSODEG00000010120
ENSODEG00000010121
ENSODEG00000010122
ENSODEG00000010124
ENSODEG00000010125
ENSODEG00000010126
ENSODEG00000010127
ENSODEG00000010129
ENSODEG00000010130
ENSODEG00000010131
ENSODEG00000010133
ENSODEG00000010135
ENSODEG00000010136
ENSODEG00000010137
ENSODEG00000010138
ENSODEG00000010141
ENSODEG00000010142
ENSODEG00000010143
ENSODEG00000010144
ENSODEG00000010146
ENSODEG00000010149
ENSODEG00000010150
ENSODEG00000010151
ENSODEG00000010154
ENSODEG00000010155
ENSODEG00000010156
ENSODEG00000010157
ENSODEG00000010161
ENSODEG00000010162
ENSODEG00000010165

ENSODEG00000010166
ENSODEG00000010167
ENSODEG00000010168
ENSODEG00000010169
ENSODEG00000010170
ENSODEG00000010172
ENSODEG00000010173
ENSODEG00000010174
ENSODEG00000010177
ENSODEG00000010178
ENSODEG00000010179
ENSODEG00000010183
ENSODEG00000010184
ENSODEG00000010185
ENSODEG00000010186
ENSODEG00000010188
ENSODEG00000010189
ENSODEG00000010192
ENSODEG00000010193
ENSODEG00000010195
ENSODEG00000010201
ENSODEG00000010202
ENSODEG00000010203
ENSODEG00000010205
ENSODEG00000010208
ENSODEG00000010210
ENSODEG00000010211
ENSODEG00000010213
ENSODEG00000010214
ENSODEG00000010215
ENSODEG00000010219
ENSODEG00000010221
ENSODEG00000010222
ENSODEG00000010223
ENSODEG00000010224
ENSODEG00000010225
ENSODEG00000010226
ENSODEG00000010227
ENSODEG00000010229
ENSODEG00000010231
ENSODEG00000010232
ENSODEG00000010235
ENSODEG00000010236
ENSODEG00000010237
ENSODEG00000010239
ENSODEG00000010240
ENSODEG00000010241
ENSODEG00000010242
ENSODEG00000010243
ENSODEG00000010245
ENSODEG00000010246
ENSODEG00000010247
ENSODEG00000010249
ENSODEG00000010252
ENSODEG00000010253
ENSODEG00000010254
ENSODEG00000010255
ENSODEG00000010256
ENSODEG00000010257

ENSODEG00000010260
ENSODEG00000010261
ENSODEG00000010262
ENSODEG00000010263
ENSODEG00000010265
ENSODEG00000010266
ENSODEG00000010268
ENSODEG00000010269
ENSODEG00000010271
ENSODEG00000010273
ENSODEG00000010274
ENSODEG00000010276
ENSODEG00000010277
ENSODEG00000010278
ENSODEG00000010280
ENSODEG00000010281
ENSODEG00000010283
ENSODEG00000010284
ENSODEG00000010285
ENSODEG00000010286
ENSODEG00000010287
ENSODEG00000010288
ENSODEG00000010290
ENSODEG00000010292
ENSODEG00000010294
ENSODEG00000010295
ENSODEG00000010296
ENSODEG00000010298
ENSODEG00000010299
ENSODEG00000010301
ENSODEG00000010302
ENSODEG00000010305
ENSODEG00000010306
ENSODEG00000010307
ENSODEG00000010308
ENSODEG00000010310
ENSODEG00000010311
ENSODEG00000010312
ENSODEG00000010314
ENSODEG00000010315
ENSODEG00000010317
ENSODEG00000010318
ENSODEG00000010319
ENSODEG00000010320
ENSODEG00000010321
ENSODEG00000010323
ENSODEG00000010324
ENSODEG00000010325
ENSODEG00000010326
ENSODEG00000010327
ENSODEG00000010328
ENSODEG00000010329
ENSODEG00000010330
ENSODEG00000010333
ENSODEG00000010334
ENSODEG00000010335
ENSODEG00000010337
ENSODEG00000010338
ENSODEG00000010339

ENSODEG00000010340
ENSODEG00000010343
ENSODEG00000010344
ENSODEG00000010348
ENSODEG00000010349
ENSODEG00000010350
ENSODEG00000010351
ENSODEG00000010352
ENSODEG00000010353
ENSODEG00000010354
ENSODEG00000010355
ENSODEG00000010356
ENSODEG00000010357
ENSODEG00000010358
ENSODEG00000010359
ENSODEG00000010360
ENSODEG00000010361
ENSODEG00000010362
ENSODEG00000010363
ENSODEG00000010365
ENSODEG00000010366
ENSODEG00000010367
ENSODEG00000010368
ENSODEG00000010369
ENSODEG00000010370
ENSODEG00000010371
ENSODEG00000010373
ENSODEG00000010374
ENSODEG00000010376
ENSODEG00000010377
ENSODEG00000010378
ENSODEG00000010379
ENSODEG00000010380
ENSODEG00000010381
ENSODEG00000010383
ENSODEG00000010384
ENSODEG00000010385
ENSODEG00000010386
ENSODEG00000010388
ENSODEG00000010389
ENSODEG00000010393
ENSODEG00000010394
ENSODEG00000010395
ENSODEG00000010397
ENSODEG00000010398
ENSODEG00000010399
ENSODEG00000010401
ENSODEG00000010402
ENSODEG00000010403
ENSODEG00000010404
ENSODEG00000010405
ENSODEG00000010406
ENSODEG00000010407
ENSODEG00000010408
ENSODEG00000010410
ENSODEG00000010411
ENSODEG00000010413
ENSODEG00000010414
ENSODEG00000010415

ENSODEG00000010416
ENSODEG00000010418
ENSODEG00000010419
ENSODEG00000010420
ENSODEG00000010421
ENSODEG00000010422
ENSODEG00000010423
ENSODEG00000010424
ENSODEG00000010425
ENSODEG00000010426
ENSODEG00000010427
ENSODEG00000010432
ENSODEG00000010434
ENSODEG00000010436
ENSODEG00000010437
ENSODEG00000010439
ENSODEG00000010440
ENSODEG00000010441
ENSODEG00000010443
ENSODEG00000010444
ENSODEG00000010445
ENSODEG00000010446
ENSODEG00000010447
ENSODEG00000010448
ENSODEG00000010449
ENSODEG00000010451
ENSODEG00000010452
ENSODEG00000010453
ENSODEG00000010454
ENSODEG00000010458
ENSODEG00000010459
ENSODEG00000010460
ENSODEG00000010461
ENSODEG00000010462
ENSODEG00000010463
ENSODEG00000010465
ENSODEG00000010466
ENSODEG00000010467
ENSODEG00000010469
ENSODEG00000010470
ENSODEG00000010471
ENSODEG00000010473
ENSODEG00000010474
ENSODEG00000010476
ENSODEG00000010482
ENSODEG00000010483
ENSODEG00000010485
ENSODEG00000010488
ENSODEG00000010489
ENSODEG00000010490
ENSODEG00000010491
ENSODEG00000010492
ENSODEG00000010493
ENSODEG00000010494
ENSODEG00000010495
ENSODEG00000010497
ENSODEG00000010498
ENSODEG00000010499
ENSODEG00000010503

ENSODEG00000010504
ENSODEG00000010505
ENSODEG00000010506
ENSODEG00000010508
ENSODEG00000010510
ENSODEG00000010511
ENSODEG00000010512
ENSODEG00000010513
ENSODEG00000010515
ENSODEG00000010516
ENSODEG00000010517
ENSODEG00000010520
ENSODEG00000010521
ENSODEG00000010522
ENSODEG00000010524
ENSODEG00000010525
ENSODEG00000010526
ENSODEG00000010529
ENSODEG00000010530
ENSODEG00000010533
ENSODEG00000010534
ENSODEG00000010535
ENSODEG00000010536
ENSODEG00000010538
ENSODEG00000010539
ENSODEG00000010542
ENSODEG00000010543
ENSODEG00000010544
ENSODEG00000010547
ENSODEG00000010548
ENSODEG00000010549
ENSODEG00000010550
ENSODEG00000010551
ENSODEG00000010552
ENSODEG00000010553
ENSODEG00000010554
ENSODEG00000010555
ENSODEG00000010556
ENSODEG00000010557
ENSODEG00000010558
ENSODEG00000010559
ENSODEG00000010560
ENSODEG00000010561
ENSODEG00000010562
ENSODEG00000010564
ENSODEG00000010565
ENSODEG00000010566
ENSODEG00000010567
ENSODEG00000010568
ENSODEG00000010569
ENSODEG00000010571
ENSODEG00000010572
ENSODEG00000010575
ENSODEG00000010576
ENSODEG00000010578
ENSODEG00000010579
ENSODEG00000010581
ENSODEG00000010583
ENSODEG00000010584

ENSODEG00000010586
ENSODEG00000010587
ENSODEG00000010590
ENSODEG00000010592
ENSODEG00000010593
ENSODEG00000010594
ENSODEG00000010596
ENSODEG00000010597
ENSODEG00000010598
ENSODEG00000010599
ENSODEG00000010601
ENSODEG00000010602
ENSODEG00000010603
ENSODEG00000010604
ENSODEG00000010605
ENSODEG00000010606
ENSODEG00000010607
ENSODEG00000010608
ENSODEG00000010609
ENSODEG00000010610
ENSODEG00000010611
ENSODEG00000010612
ENSODEG00000010614
ENSODEG00000010615
ENSODEG00000010620
ENSODEG00000010621
ENSODEG00000010622
ENSODEG00000010623
ENSODEG00000010624
ENSODEG00000010628
ENSODEG00000010629
ENSODEG00000010631
ENSODEG00000010632
ENSODEG00000010633
ENSODEG00000010635
ENSODEG00000010636
ENSODEG00000010638
ENSODEG00000010639
ENSODEG00000010640
ENSODEG00000010641
ENSODEG00000010643
ENSODEG00000010644
ENSODEG00000010646
ENSODEG00000010648
ENSODEG00000010649
ENSODEG00000010650
ENSODEG00000010651
ENSODEG00000010652
ENSODEG00000010653
ENSODEG00000010656
ENSODEG00000010657
ENSODEG00000010658
ENSODEG00000010664
ENSODEG00000010665
ENSODEG00000010666
ENSODEG00000010667
ENSODEG00000010668
ENSODEG00000010670
ENSODEG00000010671

ENSODEG00000010672
ENSODEG00000010673
ENSODEG00000010675
ENSODEG00000010676
ENSODEG00000010677
ENSODEG00000010678
ENSODEG00000010679
ENSODEG00000010680
ENSODEG00000010681
ENSODEG00000010682
ENSODEG00000010683
ENSODEG00000010685
ENSODEG00000010686
ENSODEG00000010687
ENSODEG00000010689
ENSODEG00000010690
ENSODEG00000010691
ENSODEG00000010692
ENSODEG00000010693
ENSODEG00000010695
ENSODEG00000010696
ENSODEG00000010697
ENSODEG00000010700
ENSODEG00000010702
ENSODEG00000010703
ENSODEG00000010704
ENSODEG00000010706
ENSODEG00000010707
ENSODEG00000010708
ENSODEG00000010709
ENSODEG00000010710
ENSODEG00000010712
ENSODEG00000010713
ENSODEG00000010714
ENSODEG00000010715
ENSODEG00000010716
ENSODEG00000010717
ENSODEG00000010718
ENSODEG00000010719
ENSODEG00000010720
ENSODEG00000010721
ENSODEG00000010722
ENSODEG00000010723
ENSODEG00000010726
ENSODEG00000010727
ENSODEG00000010728
ENSODEG00000010729
ENSODEG00000010732
ENSODEG00000010734
ENSODEG00000010735
ENSODEG00000010736
ENSODEG00000010737
ENSODEG00000010738
ENSODEG00000010739
ENSODEG00000010740
ENSODEG00000010742
ENSODEG00000010744
ENSODEG00000010746
ENSODEG00000010747

ENSODEG00000010750
ENSODEG00000010751
ENSODEG00000010752
ENSODEG00000010755
ENSODEG00000010756
ENSODEG00000010757
ENSODEG00000010759
ENSODEG00000010761
ENSODEG00000010762
ENSODEG00000010764
ENSODEG00000010765
ENSODEG00000010766
ENSODEG00000010768
ENSODEG00000010769
ENSODEG00000010771
ENSODEG00000010772
ENSODEG00000010773
ENSODEG00000010774
ENSODEG00000010775
ENSODEG00000010776
ENSODEG00000010778
ENSODEG00000010779
ENSODEG00000010780
ENSODEG00000010781
ENSODEG00000010784
ENSODEG00000010785
ENSODEG00000010786
ENSODEG00000010787
ENSODEG00000010788
ENSODEG00000010789
ENSODEG00000010792
ENSODEG00000010793
ENSODEG00000010794
ENSODEG00000010795
ENSODEG00000010796
ENSODEG00000010799
ENSODEG00000010800
ENSODEG00000010801
ENSODEG00000010803
ENSODEG00000010804
ENSODEG00000010805
ENSODEG00000010807
ENSODEG00000010808
ENSODEG00000010809
ENSODEG00000010811
ENSODEG00000010812
ENSODEG00000010814
ENSODEG00000010815
ENSODEG00000010816
ENSODEG00000010817
ENSODEG00000010818
ENSODEG00000010820
ENSODEG00000010821
ENSODEG00000010822
ENSODEG00000010823
ENSODEG00000010824
ENSODEG00000010825
ENSODEG00000010826
ENSODEG00000010827

ENSODEG00000010828
ENSODEG00000010829
ENSODEG00000010831
ENSODEG00000010832
ENSODEG00000010833
ENSODEG00000010834
ENSODEG00000010836
ENSODEG00000010837
ENSODEG00000010838
ENSODEG00000010839
ENSODEG00000010840
ENSODEG00000010841
ENSODEG00000010843
ENSODEG00000010845
ENSODEG00000010846
ENSODEG00000010847
ENSODEG00000010848
ENSODEG00000010850
ENSODEG00000010851
ENSODEG00000010852
ENSODEG00000010853
ENSODEG00000010854
ENSODEG00000010855
ENSODEG00000010856
ENSODEG00000010858
ENSODEG00000010859
ENSODEG00000010860
ENSODEG00000010861
ENSODEG00000010862
ENSODEG00000010863
ENSODEG00000010864
ENSODEG00000010865
ENSODEG00000010867
ENSODEG00000010868
ENSODEG00000010869
ENSODEG00000010871
ENSODEG00000010872
ENSODEG00000010874
ENSODEG00000010875
ENSODEG00000010876
ENSODEG00000010880
ENSODEG00000010881
ENSODEG00000010882
ENSODEG00000010884
ENSODEG00000010885
ENSODEG00000010888
ENSODEG00000010889
ENSODEG00000010890
ENSODEG00000010891
ENSODEG00000010895
ENSODEG00000010896
ENSODEG00000010898
ENSODEG00000010900
ENSODEG00000010902
ENSODEG00000010904
ENSODEG00000010905
ENSODEG00000010906
ENSODEG00000010907
ENSODEG00000010908

ENSODEG00000010909
ENSODEG00000010910
ENSODEG00000010911
ENSODEG00000010913
ENSODEG00000010915
ENSODEG00000010916
ENSODEG00000010917
ENSODEG00000010918
ENSODEG00000010921
ENSODEG00000010922
ENSODEG00000010923
ENSODEG00000010924
ENSODEG00000010929
ENSODEG00000010932
ENSODEG00000010933
ENSODEG00000010936
ENSODEG00000010937
ENSODEG00000010938
ENSODEG00000010940
ENSODEG00000010941
ENSODEG00000010942
ENSODEG00000010943
ENSODEG00000010944
ENSODEG00000010945
ENSODEG00000010946
ENSODEG00000010947
ENSODEG00000010948
ENSODEG00000010949
ENSODEG00000010951
ENSODEG00000010954
ENSODEG00000010955
ENSODEG00000010956
ENSODEG00000010957
ENSODEG00000010958
ENSODEG00000010959
ENSODEG00000010961
ENSODEG00000010962
ENSODEG00000010963
ENSODEG00000010965
ENSODEG00000010966
ENSODEG00000010968
ENSODEG00000010969
ENSODEG00000010972
ENSODEG00000010974
ENSODEG00000010975
ENSODEG00000010976
ENSODEG00000010977
ENSODEG00000010980
ENSODEG00000010981
ENSODEG00000010982
ENSODEG00000010984
ENSODEG00000010986
ENSODEG00000010987
ENSODEG00000010988
ENSODEG00000010989
ENSODEG00000010990
ENSODEG00000010991
ENSODEG00000010992
ENSODEG00000010993

ENSODEG00000010994
ENSODEG00000010995
ENSODEG00000010996
ENSODEG00000010997
ENSODEG00000010998
ENSODEG00000010999
ENSODEG00000011001
ENSODEG00000011002
ENSODEG00000011003
ENSODEG00000011004
ENSODEG00000011006
ENSODEG00000011007
ENSODEG00000011008
ENSODEG00000011010
ENSODEG00000011012
ENSODEG00000011013
ENSODEG00000011014
ENSODEG00000011015
ENSODEG00000011016
ENSODEG00000011018
ENSODEG00000011019
ENSODEG00000011020
ENSODEG00000011022
ENSODEG00000011023
ENSODEG00000011026
ENSODEG00000011027
ENSODEG00000011028
ENSODEG00000011030
ENSODEG00000011032
ENSODEG00000011033
ENSODEG00000011034
ENSODEG00000011035
ENSODEG00000011036
ENSODEG00000011037
ENSODEG00000011040
ENSODEG00000011041
ENSODEG00000011042
ENSODEG00000011043
ENSODEG00000011044
ENSODEG00000011045
ENSODEG00000011046
ENSODEG00000011047
ENSODEG00000011048
ENSODEG00000011049
ENSODEG00000011050
ENSODEG00000011052
ENSODEG00000011054
ENSODEG00000011055
ENSODEG00000011056
ENSODEG00000011057
ENSODEG00000011062
ENSODEG00000011064
ENSODEG00000011066
ENSODEG00000011067
ENSODEG00000011068
ENSODEG00000011069
ENSODEG00000011072
ENSODEG00000011073
ENSODEG00000011074

ENSODEG00000011075
ENSODEG00000011076
ENSODEG00000011077
ENSODEG00000011078
ENSODEG00000011079
ENSODEG00000011082
ENSODEG00000011083
ENSODEG00000011085
ENSODEG00000011086
ENSODEG00000011087
ENSODEG00000011089
ENSODEG00000011090
ENSODEG00000011091
ENSODEG00000011092
ENSODEG00000011096
ENSODEG00000011097
ENSODEG00000011100
ENSODEG00000011103
ENSODEG00000011104
ENSODEG00000011105
ENSODEG00000011106
ENSODEG00000011109
ENSODEG00000011110
ENSODEG00000011111
ENSODEG00000011112
ENSODEG00000011113
ENSODEG00000011115
ENSODEG00000011116
ENSODEG00000011120
ENSODEG00000011121
ENSODEG00000011123
ENSODEG00000011124
ENSODEG00000011125
ENSODEG00000011128
ENSODEG00000011129
ENSODEG00000011132
ENSODEG00000011133
ENSODEG00000011136
ENSODEG00000011137
ENSODEG00000011138
ENSODEG00000011139
ENSODEG00000011140
ENSODEG00000011141
ENSODEG00000011143
ENSODEG00000011144
ENSODEG00000011145
ENSODEG00000011146
ENSODEG00000011147
ENSODEG00000011148
ENSODEG00000011149
ENSODEG00000011150
ENSODEG00000011151
ENSODEG00000011152
ENSODEG00000011154
ENSODEG00000011155
ENSODEG00000011156
ENSODEG00000011157
ENSODEG00000011158
ENSODEG00000011160

ENSODEG00000011161
ENSODEG00000011163
ENSODEG00000011164
ENSODEG00000011165
ENSODEG00000011166
ENSODEG00000011167
ENSODEG00000011168
ENSODEG00000011169
ENSODEG00000011171
ENSODEG00000011172
ENSODEG00000011174
ENSODEG00000011175
ENSODEG00000011176
ENSODEG00000011180
ENSODEG00000011182
ENSODEG00000011184
ENSODEG00000011186
ENSODEG00000011187
ENSODEG00000011188
ENSODEG00000011190
ENSODEG00000011191
ENSODEG00000011192
ENSODEG00000011193
ENSODEG00000011194
ENSODEG00000011195
ENSODEG00000011197
ENSODEG00000011198
ENSODEG00000011199
ENSODEG00000011203
ENSODEG00000011204
ENSODEG00000011205
ENSODEG00000011206
ENSODEG00000011208
ENSODEG00000011209
ENSODEG00000011210
ENSODEG00000011211
ENSODEG00000011212
ENSODEG00000011213
ENSODEG00000011214
ENSODEG00000011216
ENSODEG00000011217
ENSODEG00000011218
ENSODEG00000011219
ENSODEG00000011220
ENSODEG00000011221
ENSODEG00000011223
ENSODEG00000011224
ENSODEG00000011228
ENSODEG00000011231
ENSODEG00000011232
ENSODEG00000011233
ENSODEG00000011235
ENSODEG00000011236
ENSODEG00000011238
ENSODEG00000011241
ENSODEG00000011243
ENSODEG00000011245
ENSODEG00000011246
ENSODEG00000011248

ENSODEG00000011249
ENSODEG00000011250
ENSODEG00000011251
ENSODEG00000011252
ENSODEG00000011253
ENSODEG00000011254
ENSODEG00000011255
ENSODEG00000011256
ENSODEG00000011257
ENSODEG00000011258
ENSODEG00000011259
ENSODEG00000011260
ENSODEG00000011261
ENSODEG00000011262
ENSODEG00000011264
ENSODEG00000011265
ENSODEG00000011266
ENSODEG00000011267
ENSODEG00000011268
ENSODEG00000011269
ENSODEG00000011270
ENSODEG00000011271
ENSODEG00000011273
ENSODEG00000011274
ENSODEG00000011275
ENSODEG00000011276
ENSODEG00000011277
ENSODEG00000011279
ENSODEG00000011280
ENSODEG00000011281
ENSODEG00000011282
ENSODEG00000011283
ENSODEG00000011284
ENSODEG00000011285
ENSODEG00000011286
ENSODEG00000011289
ENSODEG00000011290
ENSODEG00000011291
ENSODEG00000011293
ENSODEG00000011294
ENSODEG00000011295
ENSODEG00000011296
ENSODEG00000011297
ENSODEG00000011298
ENSODEG00000011299
ENSODEG00000011300
ENSODEG00000011301
ENSODEG00000011304
ENSODEG00000011305
ENSODEG00000011306
ENSODEG00000011309
ENSODEG00000011310
ENSODEG00000011312
ENSODEG00000011314
ENSODEG00000011316
ENSODEG00000011318
ENSODEG00000011319
ENSODEG00000011320
ENSODEG00000011322

ENSODEG00000011323
ENSODEG00000011326
ENSODEG00000011327
ENSODEG00000011328
ENSODEG00000011329
ENSODEG00000011330
ENSODEG00000011333
ENSODEG00000011334
ENSODEG00000011335
ENSODEG00000011336
ENSODEG00000011337
ENSODEG00000011338
ENSODEG00000011339
ENSODEG00000011340
ENSODEG00000011341
ENSODEG00000011342
ENSODEG00000011343
ENSODEG00000011344
ENSODEG00000011345
ENSODEG00000011347
ENSODEG00000011348
ENSODEG00000011349
ENSODEG00000011350
ENSODEG00000011351
ENSODEG00000011352
ENSODEG00000011353
ENSODEG00000011354
ENSODEG00000011357
ENSODEG00000011358
ENSODEG00000011359
ENSODEG00000011360
ENSODEG00000011362
ENSODEG00000011364
ENSODEG00000011365
ENSODEG00000011367
ENSODEG00000011368
ENSODEG00000011370
ENSODEG00000011372
ENSODEG00000011375
ENSODEG00000011376
ENSODEG00000011378
ENSODEG00000011379
ENSODEG00000011380
ENSODEG00000011382
ENSODEG00000011383
ENSODEG00000011384
ENSODEG00000011385
ENSODEG00000011386
ENSODEG00000011387
ENSODEG00000011388
ENSODEG00000011389
ENSODEG00000011390
ENSODEG00000011391
ENSODEG00000011392
ENSODEG00000011393
ENSODEG00000011394
ENSODEG00000011398
ENSODEG00000011399
ENSODEG00000011400

ENSODEG00000011401
ENSODEG00000011402
ENSODEG00000011405
ENSODEG00000011406
ENSODEG00000011407
ENSODEG00000011408
ENSODEG00000011409
ENSODEG00000011411
ENSODEG00000011412
ENSODEG00000011414
ENSODEG00000011415
ENSODEG00000011416
ENSODEG00000011420
ENSODEG00000011421
ENSODEG00000011422
ENSODEG00000011423
ENSODEG00000011424
ENSODEG00000011425
ENSODEG00000011427
ENSODEG00000011429
ENSODEG00000011430
ENSODEG00000011431
ENSODEG00000011433
ENSODEG00000011434
ENSODEG00000011437
ENSODEG00000011438
ENSODEG00000011439
ENSODEG00000011440
ENSODEG00000011441
ENSODEG00000011442
ENSODEG00000011443
ENSODEG00000011444
ENSODEG00000011445
ENSODEG00000011447
ENSODEG00000011449
ENSODEG00000011451
ENSODEG00000011452
ENSODEG00000011453
ENSODEG00000011455
ENSODEG00000011456
ENSODEG00000011458
ENSODEG00000011459
ENSODEG00000011460
ENSODEG00000011461
ENSODEG00000011462
ENSODEG00000011463
ENSODEG00000011464
ENSODEG00000011465
ENSODEG00000011466
ENSODEG00000011468
ENSODEG00000011470
ENSODEG00000011471
ENSODEG00000011472
ENSODEG00000011474
ENSODEG00000011477
ENSODEG00000011479
ENSODEG00000011481
ENSODEG00000011483
ENSODEG00000011484

ENSODEG00000011487
ENSODEG00000011490
ENSODEG00000011491
ENSODEG00000011492
ENSODEG00000011494
ENSODEG00000011496
ENSODEG00000011497
ENSODEG00000011498
ENSODEG00000011499
ENSODEG00000011500
ENSODEG00000011501
ENSODEG00000011503
ENSODEG00000011505
ENSODEG00000011507
ENSODEG00000011508
ENSODEG00000011509
ENSODEG00000011510
ENSODEG00000011511
ENSODEG00000011512
ENSODEG00000011514
ENSODEG00000011517
ENSODEG00000011519
ENSODEG00000011520
ENSODEG00000011521
ENSODEG00000011522
ENSODEG00000011524
ENSODEG00000011526
ENSODEG00000011527
ENSODEG00000011528
ENSODEG00000011530
ENSODEG00000011531
ENSODEG00000011532
ENSODEG00000011533
ENSODEG00000011534
ENSODEG00000011535
ENSODEG00000011537
ENSODEG00000011538
ENSODEG00000011539
ENSODEG00000011540
ENSODEG00000011541
ENSODEG00000011542
ENSODEG00000011544
ENSODEG00000011545
ENSODEG00000011546
ENSODEG00000011548
ENSODEG00000011549
ENSODEG00000011550
ENSODEG00000011552
ENSODEG00000011553
ENSODEG00000011554
ENSODEG00000011556
ENSODEG00000011557
ENSODEG00000011560
ENSODEG00000011562
ENSODEG00000011563
ENSODEG00000011565
ENSODEG00000011566
ENSODEG00000011567
ENSODEG00000011569

ENSODEG00000011570
ENSODEG00000011571
ENSODEG00000011572
ENSODEG00000011573
ENSODEG00000011574
ENSODEG00000011575
ENSODEG00000011577
ENSODEG00000011578
ENSODEG00000011579
ENSODEG00000011581
ENSODEG00000011584
ENSODEG00000011585
ENSODEG00000011586
ENSODEG00000011588
ENSODEG00000011589
ENSODEG00000011590
ENSODEG00000011592
ENSODEG00000011593
ENSODEG00000011594
ENSODEG00000011595
ENSODEG00000011596
ENSODEG00000011597
ENSODEG00000011598
ENSODEG00000011599
ENSODEG00000011601
ENSODEG00000011602
ENSODEG00000011603
ENSODEG00000011604
ENSODEG00000011605
ENSODEG00000011606
ENSODEG00000011607
ENSODEG00000011608
ENSODEG00000011609
ENSODEG00000011612
ENSODEG00000011614
ENSODEG00000011616
ENSODEG00000011617
ENSODEG00000011618
ENSODEG00000011619
ENSODEG00000011621
ENSODEG00000011622
ENSODEG00000011623
ENSODEG00000011625
ENSODEG00000011626
ENSODEG00000011627
ENSODEG00000011628
ENSODEG00000011629
ENSODEG00000011630
ENSODEG00000011633
ENSODEG00000011634
ENSODEG00000011636
ENSODEG00000011637
ENSODEG00000011639
ENSODEG00000011640
ENSODEG00000011641
ENSODEG00000011642
ENSODEG00000011644
ENSODEG00000011645
ENSODEG00000011647

ENSODEG00000011648
ENSODEG00000011649
ENSODEG00000011650
ENSODEG00000011651
ENSODEG00000011652
ENSODEG00000011654
ENSODEG00000011655
ENSODEG00000011656
ENSODEG00000011657
ENSODEG00000011660
ENSODEG00000011661
ENSODEG00000011662
ENSODEG00000011663
ENSODEG00000011664
ENSODEG00000011667
ENSODEG00000011669
ENSODEG00000011670
ENSODEG00000011671
ENSODEG00000011673
ENSODEG00000011675
ENSODEG00000011677
ENSODEG00000011678
ENSODEG00000011680
ENSODEG00000011682
ENSODEG00000011685
ENSODEG00000011686
ENSODEG00000011687
ENSODEG00000011688
ENSODEG00000011690
ENSODEG00000011691
ENSODEG00000011692
ENSODEG00000011693
ENSODEG00000011694
ENSODEG00000011695
ENSODEG00000011696
ENSODEG00000011697
ENSODEG00000011698
ENSODEG00000011699
ENSODEG00000011700
ENSODEG00000011701
ENSODEG00000011702
ENSODEG00000011703
ENSODEG00000011706
ENSODEG00000011709
ENSODEG00000011710
ENSODEG00000011711
ENSODEG00000011716
ENSODEG00000011718
ENSODEG00000011719
ENSODEG00000011720
ENSODEG00000011721
ENSODEG00000011722
ENSODEG00000011723
ENSODEG00000011724
ENSODEG00000011725
ENSODEG00000011728
ENSODEG00000011729
ENSODEG00000011730
ENSODEG00000011731

ENSODEG00000011733
ENSODEG00000011734
ENSODEG00000011736
ENSODEG00000011737
ENSODEG00000011740
ENSODEG00000011742
ENSODEG00000011743
ENSODEG00000011744
ENSODEG00000011746
ENSODEG00000011747
ENSODEG00000011749
ENSODEG00000011750
ENSODEG00000011751
ENSODEG00000011752
ENSODEG00000011753
ENSODEG00000011754
ENSODEG00000011755
ENSODEG00000011756
ENSODEG00000011757
ENSODEG00000011758
ENSODEG00000011759
ENSODEG00000011761
ENSODEG00000011762
ENSODEG00000011763
ENSODEG00000011765
ENSODEG00000011766
ENSODEG00000011767
ENSODEG00000011768
ENSODEG00000011769
ENSODEG00000011770
ENSODEG00000011771
ENSODEG00000011772
ENSODEG00000011773
ENSODEG00000011775
ENSODEG00000011776
ENSODEG00000011780
ENSODEG00000011782
ENSODEG00000011783
ENSODEG00000011785
ENSODEG00000011786
ENSODEG00000011789
ENSODEG00000011790
ENSODEG00000011792
ENSODEG00000011793
ENSODEG00000011795
ENSODEG00000011797
ENSODEG00000011798
ENSODEG00000011802
ENSODEG00000011804
ENSODEG00000011806
ENSODEG00000011807
ENSODEG00000011809
ENSODEG00000011810
ENSODEG00000011811
ENSODEG00000011814
ENSODEG00000011818
ENSODEG00000011819
ENSODEG00000011820
ENSODEG00000011821

ENSODEG00000011822
ENSODEG00000011823
ENSODEG00000011824
ENSODEG00000011826
ENSODEG00000011827
ENSODEG00000011828
ENSODEG00000011829
ENSODEG00000011830
ENSODEG00000011831
ENSODEG00000011832
ENSODEG00000011835
ENSODEG00000011837
ENSODEG00000011838
ENSODEG00000011839
ENSODEG00000011840
ENSODEG00000011842
ENSODEG00000011843
ENSODEG00000011844
ENSODEG00000011845
ENSODEG00000011847
ENSODEG00000011848
ENSODEG00000011849
ENSODEG00000011850
ENSODEG00000011851
ENSODEG00000011853
ENSODEG00000011854
ENSODEG00000011856
ENSODEG00000011857
ENSODEG00000011858
ENSODEG00000011859
ENSODEG00000011860
ENSODEG00000011861
ENSODEG00000011862
ENSODEG00000011864
ENSODEG00000011866
ENSODEG00000011867
ENSODEG00000011868
ENSODEG00000011869
ENSODEG00000011870
ENSODEG00000011871
ENSODEG00000011876
ENSODEG00000011878
ENSODEG00000011879
ENSODEG00000011880
ENSODEG00000011881
ENSODEG00000011882
ENSODEG00000011885
ENSODEG00000011887
ENSODEG00000011888
ENSODEG00000011889
ENSODEG00000011891
ENSODEG00000011892
ENSODEG00000011893
ENSODEG00000011894
ENSODEG00000011895
ENSODEG00000011898
ENSODEG00000011899
ENSODEG00000011901
ENSODEG00000011902

ENSODEG00000011903
ENSODEG00000011904
ENSODEG00000011905
ENSODEG00000011906
ENSODEG00000011907
ENSODEG00000011908
ENSODEG00000011909
ENSODEG00000011911
ENSODEG00000011912
ENSODEG00000011913
ENSODEG00000011914
ENSODEG00000011915
ENSODEG00000011916
ENSODEG00000011917
ENSODEG00000011918
ENSODEG00000011920
ENSODEG00000011921
ENSODEG00000011923
ENSODEG00000011924
ENSODEG00000011926
ENSODEG00000011927
ENSODEG00000011929
ENSODEG00000011931
ENSODEG00000011932
ENSODEG00000011933
ENSODEG00000011934
ENSODEG00000011935
ENSODEG00000011936
ENSODEG00000011937
ENSODEG00000011938
ENSODEG00000011940
ENSODEG00000011941
ENSODEG00000011942
ENSODEG00000011943
ENSODEG00000011944
ENSODEG00000011945
ENSODEG00000011948
ENSODEG00000011949
ENSODEG00000011950
ENSODEG00000011952
ENSODEG00000011954
ENSODEG00000011957
ENSODEG00000011960
ENSODEG00000011962
ENSODEG00000011963
ENSODEG00000011965
ENSODEG00000011966
ENSODEG00000011967
ENSODEG00000011968
ENSODEG00000011970
ENSODEG00000011971
ENSODEG00000011973
ENSODEG00000011975
ENSODEG00000011978
ENSODEG00000011980
ENSODEG00000011981
ENSODEG00000011982
ENSODEG00000011983
ENSODEG00000011984

ENSODEG00000011985
ENSODEG00000011986
ENSODEG00000011987
ENSODEG00000011989
ENSODEG00000011990
ENSODEG00000011991
ENSODEG00000011992
ENSODEG00000011993
ENSODEG00000011995
ENSODEG00000011996
ENSODEG00000011998
ENSODEG00000012000
ENSODEG00000012001
ENSODEG00000012003
ENSODEG00000012004
ENSODEG00000012005
ENSODEG00000012006
ENSODEG00000012008
ENSODEG00000012009
ENSODEG00000012011
ENSODEG00000012012
ENSODEG00000012013
ENSODEG00000012014
ENSODEG00000012015
ENSODEG00000012016
ENSODEG00000012017
ENSODEG00000012018
ENSODEG00000012020
ENSODEG00000012021
ENSODEG00000012022
ENSODEG00000012023
ENSODEG00000012024
ENSODEG00000012025
ENSODEG00000012026
ENSODEG00000012029
ENSODEG00000012030
ENSODEG00000012031
ENSODEG00000012033
ENSODEG00000012034
ENSODEG00000012035
ENSODEG00000012036
ENSODEG00000012038
ENSODEG00000012039
ENSODEG00000012041
ENSODEG00000012042
ENSODEG00000012043
ENSODEG00000012044
ENSODEG00000012045
ENSODEG00000012047
ENSODEG00000012048
ENSODEG00000012050
ENSODEG00000012051
ENSODEG00000012054
ENSODEG00000012055
ENSODEG00000012056
ENSODEG00000012057
ENSODEG00000012058
ENSODEG00000012059
ENSODEG00000012061

ENSODEG00000012062
ENSODEG00000012063
ENSODEG00000012064
ENSODEG00000012065
ENSODEG00000012066
ENSODEG00000012067
ENSODEG00000012068
ENSODEG00000012069
ENSODEG00000012071
ENSODEG00000012072
ENSODEG00000012073
ENSODEG00000012074
ENSODEG00000012075
ENSODEG00000012076
ENSODEG00000012077
ENSODEG00000012078
ENSODEG00000012079
ENSODEG00000012080
ENSODEG00000012081
ENSODEG00000012082
ENSODEG00000012083
ENSODEG00000012085
ENSODEG00000012086
ENSODEG00000012087
ENSODEG00000012091
ENSODEG00000012092
ENSODEG00000012093
ENSODEG00000012095
ENSODEG00000012096
ENSODEG00000012097
ENSODEG00000012099
ENSODEG00000012100
ENSODEG00000012101
ENSODEG00000012103
ENSODEG00000012104
ENSODEG00000012105
ENSODEG00000012107
ENSODEG00000012108
ENSODEG00000012110
ENSODEG00000012111
ENSODEG00000012113
ENSODEG00000012114
ENSODEG00000012115
ENSODEG00000012116
ENSODEG00000012117
ENSODEG00000012118
ENSODEG00000012120
ENSODEG00000012121
ENSODEG00000012122
ENSODEG00000012124
ENSODEG00000012126
ENSODEG00000012127
ENSODEG00000012128
ENSODEG00000012129
ENSODEG00000012131
ENSODEG00000012132
ENSODEG00000012134
ENSODEG00000012135
ENSODEG00000012136

ENSODEG00000012137
ENSODEG00000012139
ENSODEG00000012141
ENSODEG00000012142
ENSODEG00000012145
ENSODEG00000012148
ENSODEG00000012149
ENSODEG00000012150
ENSODEG00000012151
ENSODEG00000012153
ENSODEG00000012154
ENSODEG00000012155
ENSODEG00000012156
ENSODEG00000012157
ENSODEG00000012159
ENSODEG00000012160
ENSODEG00000012162
ENSODEG00000012163
ENSODEG00000012164
ENSODEG00000012165
ENSODEG00000012166
ENSODEG00000012167
ENSODEG00000012168
ENSODEG00000012170
ENSODEG00000012171
ENSODEG00000012173
ENSODEG00000012174
ENSODEG00000012178
ENSODEG00000012180
ENSODEG00000012181
ENSODEG00000012182
ENSODEG00000012183
ENSODEG00000012185
ENSODEG00000012186
ENSODEG00000012187
ENSODEG00000012188
ENSODEG00000012190
ENSODEG00000012191
ENSODEG00000012194
ENSODEG00000012195
ENSODEG00000012196
ENSODEG00000012199
ENSODEG00000012201
ENSODEG00000012202
ENSODEG00000012203
ENSODEG00000012204
ENSODEG00000012206
ENSODEG00000012207
ENSODEG00000012208
ENSODEG00000012209
ENSODEG00000012210
ENSODEG00000012211
ENSODEG00000012212
ENSODEG00000012213
ENSODEG00000012214
ENSODEG00000012216
ENSODEG00000012217
ENSODEG00000012218
ENSODEG00000012219

ENSODEG00000012220
ENSODEG00000012222
ENSODEG00000012223
ENSODEG00000012224
ENSODEG00000012225
ENSODEG00000012226
ENSODEG00000012227
ENSODEG00000012230
ENSODEG00000012231
ENSODEG00000012233
ENSODEG00000012234
ENSODEG00000012235
ENSODEG00000012236
ENSODEG00000012237
ENSODEG00000012238
ENSODEG00000012239
ENSODEG00000012240
ENSODEG00000012241
ENSODEG00000012242
ENSODEG00000012244
ENSODEG00000012245
ENSODEG00000012246
ENSODEG00000012247
ENSODEG00000012248
ENSODEG00000012249
ENSODEG00000012252
ENSODEG00000012253
ENSODEG00000012254
ENSODEG00000012255
ENSODEG00000012257
ENSODEG00000012258
ENSODEG00000012259
ENSODEG00000012261
ENSODEG00000012263
ENSODEG00000012265
ENSODEG00000012266
ENSODEG00000012267
ENSODEG00000012268
ENSODEG00000012271
ENSODEG00000012274
ENSODEG00000012275
ENSODEG00000012280
ENSODEG00000012281
ENSODEG00000012282
ENSODEG00000012284
ENSODEG00000012285
ENSODEG00000012286
ENSODEG00000012287
ENSODEG00000012289
ENSODEG00000012290
ENSODEG00000012291
ENSODEG00000012292
ENSODEG00000012293
ENSODEG00000012294
ENSODEG00000012295
ENSODEG00000012296
ENSODEG00000012297
ENSODEG00000012298
ENSODEG00000012299

ENSODEG00000012300
ENSODEG00000012301
ENSODEG00000012302
ENSODEG00000012304
ENSODEG00000012306
ENSODEG00000012307
ENSODEG00000012308
ENSODEG00000012309
ENSODEG00000012310
ENSODEG00000012312
ENSODEG00000012315
ENSODEG00000012316
ENSODEG00000012317
ENSODEG00000012318
ENSODEG00000012319
ENSODEG00000012320
ENSODEG00000012321
ENSODEG00000012322
ENSODEG00000012323
ENSODEG00000012324
ENSODEG00000012325
ENSODEG00000012326
ENSODEG00000012327
ENSODEG00000012328
ENSODEG00000012329
ENSODEG00000012330
ENSODEG00000012334
ENSODEG00000012335
ENSODEG00000012339
ENSODEG00000012340
ENSODEG00000012341
ENSODEG00000012342
ENSODEG00000012343
ENSODEG00000012345
ENSODEG00000012346
ENSODEG00000012347
ENSODEG00000012348
ENSODEG00000012350
ENSODEG00000012351
ENSODEG00000012352
ENSODEG00000012353
ENSODEG00000012355
ENSODEG00000012356
ENSODEG00000012357
ENSODEG00000012358
ENSODEG00000012360
ENSODEG00000012361
ENSODEG00000012362
ENSODEG00000012363
ENSODEG00000012364
ENSODEG00000012365
ENSODEG00000012367
ENSODEG00000012368
ENSODEG00000012369
ENSODEG00000012370
ENSODEG00000012371
ENSODEG00000012372
ENSODEG00000012373
ENSODEG00000012374

ENSODEG00000012375
ENSODEG00000012376
ENSODEG00000012377
ENSODEG00000012379
ENSODEG00000012380
ENSODEG00000012381
ENSODEG00000012382
ENSODEG00000012383
ENSODEG00000012384
ENSODEG00000012385
ENSODEG00000012386
ENSODEG00000012389
ENSODEG00000012390
ENSODEG00000012392
ENSODEG00000012395
ENSODEG00000012396
ENSODEG00000012397
ENSODEG00000012398
ENSODEG00000012399
ENSODEG00000012400
ENSODEG00000012401
ENSODEG00000012402
ENSODEG00000012403
ENSODEG00000012404
ENSODEG00000012405
ENSODEG00000012408
ENSODEG00000012409
ENSODEG00000012411
ENSODEG00000012412
ENSODEG00000012413
ENSODEG00000012414
ENSODEG00000012415
ENSODEG00000012416
ENSODEG00000012418
ENSODEG00000012419
ENSODEG00000012420
ENSODEG00000012422
ENSODEG00000012423
ENSODEG00000012424
ENSODEG00000012425
ENSODEG00000012427
ENSODEG00000012428
ENSODEG00000012430
ENSODEG00000012432
ENSODEG00000012433
ENSODEG00000012434
ENSODEG00000012436
ENSODEG00000012438
ENSODEG00000012441
ENSODEG00000012442
ENSODEG00000012444
ENSODEG00000012447
ENSODEG00000012448
ENSODEG00000012449
ENSODEG00000012451
ENSODEG00000012452
ENSODEG00000012453
ENSODEG00000012454
ENSODEG00000012457

ENSODEG00000012460
ENSODEG00000012461
ENSODEG00000012462
ENSODEG00000012463
ENSODEG00000012464
ENSODEG00000012465
ENSODEG00000012466
ENSODEG00000012467
ENSODEG00000012469
ENSODEG00000012471
ENSODEG00000012472
ENSODEG00000012473
ENSODEG00000012474
ENSODEG00000012475
ENSODEG00000012476
ENSODEG00000012477
ENSODEG00000012479
ENSODEG00000012480
ENSODEG00000012481
ENSODEG00000012482
ENSODEG00000012483
ENSODEG00000012485
ENSODEG00000012486
ENSODEG00000012488
ENSODEG00000012489
ENSODEG00000012490
ENSODEG00000012491
ENSODEG00000012492
ENSODEG00000012493
ENSODEG00000012494
ENSODEG00000012496
ENSODEG00000012498
ENSODEG00000012499
ENSODEG00000012502
ENSODEG00000012503
ENSODEG00000012504
ENSODEG00000012505
ENSODEG00000012507
ENSODEG00000012508
ENSODEG00000012509
ENSODEG00000012510
ENSODEG00000012511
ENSODEG00000012512
ENSODEG00000012513
ENSODEG00000012514
ENSODEG00000012515
ENSODEG00000012516
ENSODEG00000012518
ENSODEG00000012519
ENSODEG00000012520
ENSODEG00000012521
ENSODEG00000012523
ENSODEG00000012524
ENSODEG00000012525
ENSODEG00000012529
ENSODEG00000012531
ENSODEG00000012532
ENSODEG00000012533
ENSODEG00000012534

ENSODEG00000012536
ENSODEG00000012538
ENSODEG00000012539
ENSODEG00000012541
ENSODEG00000012542
ENSODEG00000012543
ENSODEG00000012545
ENSODEG00000012547
ENSODEG00000012548
ENSODEG00000012549
ENSODEG00000012550
ENSODEG00000012553
ENSODEG00000012554
ENSODEG00000012555
ENSODEG00000012556
ENSODEG00000012557
ENSODEG00000012560
ENSODEG00000012561
ENSODEG00000012562
ENSODEG00000012563
ENSODEG00000012564
ENSODEG00000012566
ENSODEG00000012568
ENSODEG00000012569
ENSODEG00000012570
ENSODEG00000012571
ENSODEG00000012575
ENSODEG00000012578
ENSODEG00000012579
ENSODEG00000012581
ENSODEG00000012583
ENSODEG00000012585
ENSODEG00000012586
ENSODEG00000012587
ENSODEG00000012588
ENSODEG00000012589
ENSODEG00000012590
ENSODEG00000012591
ENSODEG00000012593
ENSODEG00000012594
ENSODEG00000012596
ENSODEG00000012597
ENSODEG00000012598
ENSODEG00000012599
ENSODEG00000012600
ENSODEG00000012601
ENSODEG00000012602
ENSODEG00000012603
ENSODEG00000012604
ENSODEG00000012605
ENSODEG00000012606
ENSODEG00000012609
ENSODEG00000012611
ENSODEG00000012612
ENSODEG00000012613
ENSODEG00000012614
ENSODEG00000012615
ENSODEG00000012616
ENSODEG00000012617

ENSODEG00000012618
ENSODEG00000012619
ENSODEG00000012621
ENSODEG00000012623
ENSODEG00000012624
ENSODEG00000012625
ENSODEG00000012626
ENSODEG00000012627
ENSODEG00000012628
ENSODEG00000012629
ENSODEG00000012631
ENSODEG00000012632
ENSODEG00000012633
ENSODEG00000012635
ENSODEG00000012636
ENSODEG00000012637
ENSODEG00000012640
ENSODEG00000012641
ENSODEG00000012642
ENSODEG00000012643
ENSODEG00000012644
ENSODEG00000012645
ENSODEG00000012646
ENSODEG00000012647
ENSODEG00000012652
ENSODEG00000012653
ENSODEG00000012655
ENSODEG00000012657
ENSODEG00000012658
ENSODEG00000012659
ENSODEG00000012664
ENSODEG00000012665
ENSODEG00000012667
ENSODEG00000012670
ENSODEG00000012672
ENSODEG00000012673
ENSODEG00000012674
ENSODEG00000012675
ENSODEG00000012676
ENSODEG00000012677
ENSODEG00000012678
ENSODEG00000012679
ENSODEG00000012680
ENSODEG00000012681
ENSODEG00000012682
ENSODEG00000012687
ENSODEG00000012688
ENSODEG00000012689
ENSODEG00000012690
ENSODEG00000012691
ENSODEG00000012693
ENSODEG00000012694
ENSODEG00000012695
ENSODEG00000012696
ENSODEG00000012697
ENSODEG00000012699
ENSODEG00000012701
ENSODEG00000012702
ENSODEG00000012704

ENSODEG00000012705
ENSODEG00000012707
ENSODEG00000012708
ENSODEG00000012710
ENSODEG00000012712
ENSODEG00000012713
ENSODEG00000012714
ENSODEG00000012717
ENSODEG00000012718
ENSODEG00000012720
ENSODEG00000012721
ENSODEG00000012723
ENSODEG00000012724
ENSODEG00000012725
ENSODEG00000012728
ENSODEG00000012729
ENSODEG00000012730
ENSODEG00000012731
ENSODEG00000012732
ENSODEG00000012733
ENSODEG00000012734
ENSODEG00000012735
ENSODEG00000012736
ENSODEG00000012737
ENSODEG00000012738
ENSODEG00000012739
ENSODEG00000012740
ENSODEG00000012741
ENSODEG00000012743
ENSODEG00000012746
ENSODEG00000012748
ENSODEG00000012750
ENSODEG00000012752
ENSODEG00000012753
ENSODEG00000012755
ENSODEG00000012756
ENSODEG00000012759
ENSODEG00000012760
ENSODEG00000012761
ENSODEG00000012763
ENSODEG00000012766
ENSODEG00000012767
ENSODEG00000012768
ENSODEG00000012769
ENSODEG00000012770
ENSODEG00000012771
ENSODEG00000012774
ENSODEG00000012775
ENSODEG00000012777
ENSODEG00000012778
ENSODEG00000012780
ENSODEG00000012782
ENSODEG00000012783
ENSODEG00000012786
ENSODEG00000012789
ENSODEG00000012790
ENSODEG00000012792
ENSODEG00000012794
ENSODEG00000012795

ENSODEG00000012796
ENSODEG00000012798
ENSODEG00000012799
ENSODEG00000012801
ENSODEG00000012802
ENSODEG00000012803
ENSODEG00000012804
ENSODEG00000012806
ENSODEG00000012808
ENSODEG00000012809
ENSODEG00000012810
ENSODEG00000012811
ENSODEG00000012814
ENSODEG00000012816
ENSODEG00000012817
ENSODEG00000012818
ENSODEG00000012821
ENSODEG00000012822
ENSODEG00000012823
ENSODEG00000012824
ENSODEG00000012827
ENSODEG00000012828
ENSODEG00000012829
ENSODEG00000012830
ENSODEG00000012834
ENSODEG00000012835
ENSODEG00000012837
ENSODEG00000012838
ENSODEG00000012839
ENSODEG00000012840
ENSODEG00000012841
ENSODEG00000012842
ENSODEG00000012843
ENSODEG00000012844
ENSODEG00000012845
ENSODEG00000012847
ENSODEG00000012848
ENSODEG00000012849
ENSODEG00000012850
ENSODEG00000012852
ENSODEG00000012853
ENSODEG00000012854
ENSODEG00000012856
ENSODEG00000012858
ENSODEG00000012859
ENSODEG00000012860
ENSODEG00000012861
ENSODEG00000012862
ENSODEG00000012863
ENSODEG00000012864
ENSODEG00000012865
ENSODEG00000012866
ENSODEG00000012867
ENSODEG00000012868
ENSODEG00000012869
ENSODEG00000012870
ENSODEG00000012872
ENSODEG00000012874
ENSODEG00000012875

ENSODEG00000012876
ENSODEG00000012877
ENSODEG00000012878
ENSODEG00000012879
ENSODEG00000012880
ENSODEG00000012881
ENSODEG00000012883
ENSODEG00000012884
ENSODEG00000012885
ENSODEG00000012886
ENSODEG00000012888
ENSODEG00000012889
ENSODEG00000012890
ENSODEG00000012891
ENSODEG00000012892
ENSODEG00000012893
ENSODEG00000012894
ENSODEG00000012897
ENSODEG00000012898
ENSODEG00000012900
ENSODEG00000012901
ENSODEG00000012903
ENSODEG00000012904
ENSODEG00000012905
ENSODEG00000012906
ENSODEG00000012907
ENSODEG00000012908
ENSODEG00000012910
ENSODEG00000012911
ENSODEG00000012912
ENSODEG00000012914
ENSODEG00000012915
ENSODEG00000012916
ENSODEG00000012917
ENSODEG00000012918
ENSODEG00000012919
ENSODEG00000012921
ENSODEG00000012923
ENSODEG00000012924
ENSODEG00000012926
ENSODEG00000012927
ENSODEG00000012929
ENSODEG00000012930
ENSODEG00000012931
ENSODEG00000012934
ENSODEG00000012935
ENSODEG00000012936
ENSODEG00000012937
ENSODEG00000012939
ENSODEG00000012940
ENSODEG00000012941
ENSODEG00000012943
ENSODEG00000012945
ENSODEG00000012946
ENSODEG00000012947
ENSODEG00000012948
ENSODEG00000012949
ENSODEG00000012951
ENSODEG00000012952

ENSODEG00000012953
ENSODEG00000012954
ENSODEG00000012956
ENSODEG00000012957
ENSODEG00000012958
ENSODEG00000012959
ENSODEG00000012960
ENSODEG00000012961
ENSODEG00000012962
ENSODEG00000012963
ENSODEG00000012964
ENSODEG00000012967
ENSODEG00000012968
ENSODEG00000012969
ENSODEG00000012973
ENSODEG00000012974
ENSODEG00000012976
ENSODEG00000012977
ENSODEG00000012978
ENSODEG00000012980
ENSODEG00000012982
ENSODEG00000012983
ENSODEG00000012984
ENSODEG00000012986
ENSODEG00000012988
ENSODEG00000012990
ENSODEG00000012991
ENSODEG00000012992
ENSODEG00000012993
ENSODEG00000012994
ENSODEG00000012996
ENSODEG00000012997
ENSODEG00000013000
ENSODEG00000013002
ENSODEG00000013006
ENSODEG00000013009
ENSODEG00000013012
ENSODEG00000013013
ENSODEG00000013015
ENSODEG00000013016
ENSODEG00000013017
ENSODEG00000013018
ENSODEG00000013019
ENSODEG00000013020
ENSODEG00000013021
ENSODEG00000013024
ENSODEG00000013027
ENSODEG00000013028
ENSODEG00000013029
ENSODEG00000013030
ENSODEG00000013031
ENSODEG00000013032
ENSODEG00000013033
ENSODEG00000013034
ENSODEG00000013035
ENSODEG00000013036
ENSODEG00000013037
ENSODEG00000013038
ENSODEG00000013041

ENSODEG00000013042
ENSODEG00000013043
ENSODEG00000013045
ENSODEG00000013047
ENSODEG00000013048
ENSODEG00000013049
ENSODEG00000013050
ENSODEG00000013051
ENSODEG00000013052
ENSODEG00000013053
ENSODEG00000013054
ENSODEG00000013056
ENSODEG00000013057
ENSODEG00000013058
ENSODEG00000013060
ENSODEG00000013061
ENSODEG00000013062
ENSODEG00000013063
ENSODEG00000013064
ENSODEG00000013065
ENSODEG00000013066
ENSODEG00000013067
ENSODEG00000013068
ENSODEG00000013069
ENSODEG00000013070
ENSODEG00000013071
ENSODEG00000013072
ENSODEG00000013073
ENSODEG00000013074
ENSODEG00000013075
ENSODEG00000013076
ENSODEG00000013078
ENSODEG00000013079
ENSODEG00000013081
ENSODEG00000013082
ENSODEG00000013083
ENSODEG00000013084
ENSODEG00000013085
ENSODEG00000013086
ENSODEG00000013087
ENSODEG00000013089
ENSODEG00000013090
ENSODEG00000013091
ENSODEG00000013092
ENSODEG00000013094
ENSODEG00000013095
ENSODEG00000013096
ENSODEG00000013098
ENSODEG00000013099
ENSODEG00000013101
ENSODEG00000013102
ENSODEG00000013104
ENSODEG00000013105
ENSODEG00000013107
ENSODEG00000013108
ENSODEG00000013110
ENSODEG00000013111
ENSODEG00000013112
ENSODEG00000013113

ENSODEG00000013116
ENSODEG00000013117
ENSODEG00000013119
ENSODEG00000013120
ENSODEG00000013123
ENSODEG00000013125
ENSODEG00000013126
ENSODEG00000013127
ENSODEG00000013128
ENSODEG00000013130
ENSODEG00000013131
ENSODEG00000013132
ENSODEG00000013134
ENSODEG00000013135
ENSODEG00000013137
ENSODEG00000013138
ENSODEG00000013141
ENSODEG00000013142
ENSODEG00000013143
ENSODEG00000013144
ENSODEG00000013145
ENSODEG00000013146
ENSODEG00000013147
ENSODEG00000013148
ENSODEG00000013150
ENSODEG00000013151
ENSODEG00000013152
ENSODEG00000013154
ENSODEG00000013155
ENSODEG00000013159
ENSODEG00000013160
ENSODEG00000013163
ENSODEG00000013164
ENSODEG00000013165
ENSODEG00000013167
ENSODEG00000013168
ENSODEG00000013169
ENSODEG00000013172
ENSODEG00000013173
ENSODEG00000013174
ENSODEG00000013176
ENSODEG00000013177
ENSODEG00000013178
ENSODEG00000013179
ENSODEG00000013180
ENSODEG00000013181
ENSODEG00000013182
ENSODEG00000013184
ENSODEG00000013186
ENSODEG00000013187
ENSODEG00000013190
ENSODEG00000013192
ENSODEG00000013193
ENSODEG00000013194
ENSODEG00000013195
ENSODEG00000013199
ENSODEG00000013200
ENSODEG00000013201
ENSODEG00000013203

ENSODEG00000013205
ENSODEG00000013206
ENSODEG00000013209
ENSODEG00000013210
ENSODEG00000013211
ENSODEG00000013212
ENSODEG00000013213
ENSODEG00000013214
ENSODEG00000013216
ENSODEG00000013217
ENSODEG00000013218
ENSODEG00000013219
ENSODEG00000013221
ENSODEG00000013222
ENSODEG00000013223
ENSODEG00000013224
ENSODEG00000013225
ENSODEG00000013226
ENSODEG00000013227
ENSODEG00000013231
ENSODEG00000013232
ENSODEG00000013233
ENSODEG00000013235
ENSODEG00000013237
ENSODEG00000013238
ENSODEG00000013240
ENSODEG00000013241
ENSODEG00000013244
ENSODEG00000013245
ENSODEG00000013246
ENSODEG00000013247
ENSODEG00000013249
ENSODEG00000013250
ENSODEG00000013253
ENSODEG00000013254
ENSODEG00000013258
ENSODEG00000013259
ENSODEG00000013261
ENSODEG00000013262
ENSODEG00000013263
ENSODEG00000013264
ENSODEG00000013265
ENSODEG00000013266
ENSODEG00000013267
ENSODEG00000013268
ENSODEG00000013269
ENSODEG00000013270
ENSODEG00000013271
ENSODEG00000013274
ENSODEG00000013275
ENSODEG00000013276
ENSODEG00000013277
ENSODEG00000013278
ENSODEG00000013279
ENSODEG00000013281
ENSODEG00000013282
ENSODEG00000013283
ENSODEG00000013284
ENSODEG00000013285

ENSODEG00000013286
ENSODEG00000013287
ENSODEG00000013290
ENSODEG00000013291
ENSODEG00000013292
ENSODEG00000013293
ENSODEG00000013295
ENSODEG00000013296
ENSODEG00000013297
ENSODEG00000013298
ENSODEG00000013299
ENSODEG00000013300
ENSODEG00000013301
ENSODEG00000013302
ENSODEG00000013303
ENSODEG00000013306
ENSODEG00000013308
ENSODEG00000013309
ENSODEG00000013310
ENSODEG00000013312
ENSODEG00000013313
ENSODEG00000013314
ENSODEG00000013315
ENSODEG00000013316
ENSODEG00000013318
ENSODEG00000013319
ENSODEG00000013320
ENSODEG00000013322
ENSODEG00000013323
ENSODEG00000013324
ENSODEG00000013325
ENSODEG00000013327
ENSODEG00000013328
ENSODEG00000013329
ENSODEG00000013330
ENSODEG00000013331
ENSODEG00000013332
ENSODEG00000013333
ENSODEG00000013334
ENSODEG00000013337
ENSODEG00000013340
ENSODEG00000013341
ENSODEG00000013342
ENSODEG00000013343
ENSODEG00000013345
ENSODEG00000013346
ENSODEG00000013347
ENSODEG00000013348
ENSODEG00000013350
ENSODEG00000013352
ENSODEG00000013353
ENSODEG00000013354
ENSODEG00000013355
ENSODEG00000013357
ENSODEG00000013359
ENSODEG00000013361
ENSODEG00000013362
ENSODEG00000013364
ENSODEG00000013365

ENSODEG00000013366
ENSODEG00000013368
ENSODEG00000013372
ENSODEG00000013374
ENSODEG00000013377
ENSODEG00000013378
ENSODEG00000013379
ENSODEG00000013381
ENSODEG00000013382
ENSODEG00000013383
ENSODEG00000013385
ENSODEG00000013386
ENSODEG00000013387
ENSODEG00000013388
ENSODEG00000013389
ENSODEG00000013391
ENSODEG00000013392
ENSODEG00000013394
ENSODEG00000013395
ENSODEG00000013396
ENSODEG00000013397
ENSODEG00000013398
ENSODEG00000013399
ENSODEG00000013400
ENSODEG00000013401
ENSODEG00000013402
ENSODEG00000013403
ENSODEG00000013404
ENSODEG00000013406
ENSODEG00000013407
ENSODEG00000013408
ENSODEG00000013410
ENSODEG00000013411
ENSODEG00000013414
ENSODEG00000013415
ENSODEG00000013419
ENSODEG00000013420
ENSODEG00000013421
ENSODEG00000013422
ENSODEG00000013424
ENSODEG00000013425
ENSODEG00000013426
ENSODEG00000013428
ENSODEG00000013429
ENSODEG00000013430
ENSODEG00000013432
ENSODEG00000013433
ENSODEG00000013434
ENSODEG00000013435
ENSODEG00000013437
ENSODEG00000013438
ENSODEG00000013439
ENSODEG00000013440
ENSODEG00000013441
ENSODEG00000013443
ENSODEG00000013444
ENSODEG00000013445
ENSODEG00000013446
ENSODEG00000013448

ENSODEG00000013450
ENSODEG00000013453
ENSODEG00000013454
ENSODEG00000013455
ENSODEG00000013456
ENSODEG00000013457
ENSODEG00000013458
ENSODEG00000013459
ENSODEG00000013461
ENSODEG00000013462
ENSODEG00000013463
ENSODEG00000013464
ENSODEG00000013465
ENSODEG00000013466
ENSODEG00000013467
ENSODEG00000013468
ENSODEG00000013470
ENSODEG00000013472
ENSODEG00000013473
ENSODEG00000013477
ENSODEG00000013480
ENSODEG00000013481
ENSODEG00000013483
ENSODEG00000013484
ENSODEG00000013485
ENSODEG00000013486
ENSODEG00000013487
ENSODEG00000013488
ENSODEG00000013491
ENSODEG00000013492
ENSODEG00000013495
ENSODEG00000013497
ENSODEG00000013498
ENSODEG00000013500
ENSODEG00000013502
ENSODEG00000013503
ENSODEG00000013505
ENSODEG00000013506
ENSODEG00000013507
ENSODEG00000013511
ENSODEG00000013512
ENSODEG00000013514
ENSODEG00000013515
ENSODEG00000013516
ENSODEG00000013517
ENSODEG00000013518
ENSODEG00000013519
ENSODEG00000013521
ENSODEG00000013523
ENSODEG00000013524
ENSODEG00000013526
ENSODEG00000013528
ENSODEG00000013529
ENSODEG00000013530
ENSODEG00000013531
ENSODEG00000013533
ENSODEG00000013534
ENSODEG00000013535
ENSODEG00000013538

ENSODEG00000013539
ENSODEG00000013540
ENSODEG00000013542
ENSODEG00000013543
ENSODEG00000013545
ENSODEG00000013546
ENSODEG00000013547
ENSODEG00000013548
ENSODEG00000013549
ENSODEG00000013550
ENSODEG00000013551
ENSODEG00000013552
ENSODEG00000013554
ENSODEG00000013555
ENSODEG00000013557
ENSODEG00000013558
ENSODEG00000013559
ENSODEG00000013562
ENSODEG00000013563
ENSODEG00000013564
ENSODEG00000013565
ENSODEG00000013566
ENSODEG00000013568
ENSODEG00000013569
ENSODEG00000013570
ENSODEG00000013571
ENSODEG00000013572
ENSODEG00000013574
ENSODEG00000013575
ENSODEG00000013576
ENSODEG00000013577
ENSODEG00000013578
ENSODEG00000013579
ENSODEG00000013580
ENSODEG00000013581
ENSODEG00000013584
ENSODEG00000013585
ENSODEG00000013586
ENSODEG00000013587
ENSODEG00000013588
ENSODEG00000013589
ENSODEG00000013591
ENSODEG00000013592
ENSODEG00000013593
ENSODEG00000013594
ENSODEG00000013595
ENSODEG00000013597
ENSODEG00000013600
ENSODEG00000013602
ENSODEG00000013603
ENSODEG00000013604
ENSODEG00000013605
ENSODEG00000013606
ENSODEG00000013608
ENSODEG00000013609
ENSODEG00000013610
ENSODEG00000013611
ENSODEG00000013613
ENSODEG00000013614

ENSODEG00000013616
ENSODEG00000013617
ENSODEG00000013618
ENSODEG00000013619
ENSODEG00000013620
ENSODEG00000013621
ENSODEG00000013622
ENSODEG00000013623
ENSODEG00000013624
ENSODEG00000013627
ENSODEG00000013628
ENSODEG00000013629
ENSODEG00000013632
ENSODEG00000013633
ENSODEG00000013634
ENSODEG00000013637
ENSODEG00000013638
ENSODEG00000013641
ENSODEG00000013643
ENSODEG00000013644
ENSODEG00000013646
ENSODEG00000013647
ENSODEG00000013649
ENSODEG00000013650
ENSODEG00000013651
ENSODEG00000013652
ENSODEG00000013654
ENSODEG00000013655
ENSODEG00000013656
ENSODEG00000013657
ENSODEG00000013658
ENSODEG00000013661
ENSODEG00000013662
ENSODEG00000013663
ENSODEG00000013664
ENSODEG00000013665
ENSODEG00000013666
ENSODEG00000013669
ENSODEG00000013670
ENSODEG00000013671
ENSODEG00000013672
ENSODEG00000013674
ENSODEG00000013675
ENSODEG00000013676
ENSODEG00000013677
ENSODEG00000013678
ENSODEG00000013679
ENSODEG00000013680
ENSODEG00000013683
ENSODEG00000013684
ENSODEG00000013685
ENSODEG00000013686
ENSODEG00000013687
ENSODEG00000013688
ENSODEG00000013689
ENSODEG00000013690
ENSODEG00000013691
ENSODEG00000013693
ENSODEG00000013694

ENSODEG00000013695
ENSODEG00000013696
ENSODEG00000013698
ENSODEG00000013699
ENSODEG00000013700
ENSODEG00000013703
ENSODEG00000013704
ENSODEG00000013706
ENSODEG00000013708
ENSODEG00000013709
ENSODEG00000013710
ENSODEG00000013712
ENSODEG00000013713
ENSODEG00000013714
ENSODEG00000013715
ENSODEG00000013716
ENSODEG00000013717
ENSODEG00000013718
ENSODEG00000013719
ENSODEG00000013720
ENSODEG00000013721
ENSODEG00000013722
ENSODEG00000013723
ENSODEG00000013726
ENSODEG00000013727
ENSODEG00000013728
ENSODEG00000013729
ENSODEG00000013730
ENSODEG00000013733
ENSODEG00000013735
ENSODEG00000013736
ENSODEG00000013738
ENSODEG00000013740
ENSODEG00000013741
ENSODEG00000013742
ENSODEG00000013743
ENSODEG00000013744
ENSODEG00000013745
ENSODEG00000013746
ENSODEG00000013749
ENSODEG00000013750
ENSODEG00000013751
ENSODEG00000013752
ENSODEG00000013755
ENSODEG00000013756
ENSODEG00000013758
ENSODEG00000013759
ENSODEG00000013760
ENSODEG00000013761
ENSODEG00000013762
ENSODEG00000013763
ENSODEG00000013764
ENSODEG00000013766
ENSODEG00000013767
ENSODEG00000013768
ENSODEG00000013770
ENSODEG00000013771
ENSODEG00000013772
ENSODEG00000013773

ENSODEG00000013775
ENSODEG00000013776
ENSODEG00000013779
ENSODEG00000013780
ENSODEG00000013781
ENSODEG00000013783
ENSODEG00000013784
ENSODEG00000013785
ENSODEG00000013786
ENSODEG00000013787
ENSODEG00000013788
ENSODEG00000013791
ENSODEG00000013792
ENSODEG00000013793
ENSODEG00000013795
ENSODEG00000013796
ENSODEG00000013799
ENSODEG00000013800
ENSODEG00000013801
ENSODEG00000013802
ENSODEG00000013803
ENSODEG00000013804
ENSODEG00000013805
ENSODEG00000013807
ENSODEG00000013808
ENSODEG00000013809
ENSODEG00000013810
ENSODEG00000013812
ENSODEG00000013813
ENSODEG00000013814
ENSODEG00000013816
ENSODEG00000013818
ENSODEG00000013819
ENSODEG00000013820
ENSODEG00000013821
ENSODEG00000013822
ENSODEG00000013823
ENSODEG00000013824
ENSODEG00000013825
ENSODEG00000013827
ENSODEG00000013828
ENSODEG00000013830
ENSODEG00000013832
ENSODEG00000013833
ENSODEG00000013834
ENSODEG00000013835
ENSODEG00000013838
ENSODEG00000013839
ENSODEG00000013840
ENSODEG00000013843
ENSODEG00000013844
ENSODEG00000013846
ENSODEG00000013848
ENSODEG00000013849
ENSODEG00000013851
ENSODEG00000013852
ENSODEG00000013854
ENSODEG00000013855
ENSODEG00000013856

ENSODEG00000013857
ENSODEG00000013858
ENSODEG00000013859
ENSODEG00000013861
ENSODEG00000013862
ENSODEG00000013863
ENSODEG00000013864
ENSODEG00000013865
ENSODEG00000013868
ENSODEG00000013870
ENSODEG00000013871
ENSODEG00000013872
ENSODEG00000013875
ENSODEG00000013876
ENSODEG00000013877
ENSODEG00000013878
ENSODEG00000013879
ENSODEG00000013880
ENSODEG00000013881
ENSODEG00000013883
ENSODEG00000013884
ENSODEG00000013886
ENSODEG00000013887
ENSODEG00000013888
ENSODEG00000013889
ENSODEG00000013890
ENSODEG00000013891
ENSODEG00000013892
ENSODEG00000013893
ENSODEG00000013894
ENSODEG00000013895
ENSODEG00000013898
ENSODEG00000013899
ENSODEG00000013900
ENSODEG00000013901
ENSODEG00000013904
ENSODEG00000013905
ENSODEG00000013909
ENSODEG00000013910
ENSODEG00000013911
ENSODEG00000013912
ENSODEG00000013913
ENSODEG00000013914
ENSODEG00000013915
ENSODEG00000013916
ENSODEG00000013917
ENSODEG00000013918
ENSODEG00000013919
ENSODEG00000013920
ENSODEG00000013921
ENSODEG00000013922
ENSODEG00000013926
ENSODEG00000013928
ENSODEG00000013929
ENSODEG00000013930
ENSODEG00000013931
ENSODEG00000013932
ENSODEG00000013933
ENSODEG00000013934

ENSODEG00000013935
ENSODEG00000013936
ENSODEG00000013938
ENSODEG00000013939
ENSODEG00000013940
ENSODEG00000013941
ENSODEG00000013942
ENSODEG00000013944
ENSODEG00000013945
ENSODEG00000013946
ENSODEG00000013949
ENSODEG00000013950
ENSODEG00000013952
ENSODEG00000013953
ENSODEG00000013954
ENSODEG00000013956
ENSODEG00000013957
ENSODEG00000013958
ENSODEG00000013960
ENSODEG00000013963
ENSODEG00000013964
ENSODEG00000013966
ENSODEG00000013967
ENSODEG00000013968
ENSODEG00000013969
ENSODEG00000013970
ENSODEG00000013971
ENSODEG00000013973
ENSODEG00000013975
ENSODEG00000013976
ENSODEG00000013977
ENSODEG00000013978
ENSODEG00000013979
ENSODEG00000013980
ENSODEG00000013981
ENSODEG00000013982
ENSODEG00000013984
ENSODEG00000013986
ENSODEG00000013987
ENSODEG00000013988
ENSODEG00000013989
ENSODEG00000013991
ENSODEG00000013992
ENSODEG00000013994
ENSODEG00000013995
ENSODEG00000014000
ENSODEG00000014001
ENSODEG00000014002
ENSODEG00000014003
ENSODEG00000014004
ENSODEG00000014005
ENSODEG00000014006
ENSODEG00000014008
ENSODEG00000014010
ENSODEG00000014011
ENSODEG00000014013
ENSODEG00000014016
ENSODEG00000014017
ENSODEG00000014018

ENSODEG00000014020
ENSODEG00000014021
ENSODEG00000014022
ENSODEG00000014023
ENSODEG00000014024
ENSODEG00000014026
ENSODEG00000014027
ENSODEG00000014029
ENSODEG00000014031
ENSODEG00000014032
ENSODEG00000014034
ENSODEG00000014036
ENSODEG00000014039
ENSODEG00000014040
ENSODEG00000014042
ENSODEG00000014044
ENSODEG00000014045
ENSODEG00000014046
ENSODEG00000014047
ENSODEG00000014048
ENSODEG00000014050
ENSODEG00000014051
ENSODEG00000014053
ENSODEG00000014054
ENSODEG00000014055
ENSODEG00000014057
ENSODEG00000014058
ENSODEG00000014060
ENSODEG00000014062
ENSODEG00000014063
ENSODEG00000014064
ENSODEG00000014065
ENSODEG00000014066
ENSODEG00000014068
ENSODEG00000014070
ENSODEG00000014071
ENSODEG00000014072
ENSODEG00000014074
ENSODEG00000014075
ENSODEG00000014076
ENSODEG00000014078
ENSODEG00000014082
ENSODEG00000014086
ENSODEG00000014087
ENSODEG00000014088
ENSODEG00000014089
ENSODEG00000014091
ENSODEG00000014094
ENSODEG00000014096
ENSODEG00000014097
ENSODEG00000014098
ENSODEG00000014099
ENSODEG00000014100
ENSODEG00000014102
ENSODEG00000014103
ENSODEG00000014105
ENSODEG00000014106
ENSODEG00000014107
ENSODEG00000014111

ENSODEG00000014112
ENSODEG00000014113
ENSODEG00000014114
ENSODEG00000014115
ENSODEG00000014117
ENSODEG00000014120
ENSODEG00000014122
ENSODEG00000014124
ENSODEG00000014125
ENSODEG00000014127
ENSODEG00000014128
ENSODEG00000014129
ENSODEG00000014131
ENSODEG00000014132
ENSODEG00000014133
ENSODEG00000014134
ENSODEG00000014135
ENSODEG00000014138
ENSODEG00000014139
ENSODEG00000014140
ENSODEG00000014142
ENSODEG00000014143
ENSODEG00000014144
ENSODEG00000014146
ENSODEG00000014147
ENSODEG00000014149
ENSODEG00000014150
ENSODEG00000014151
ENSODEG00000014153
ENSODEG00000014156
ENSODEG00000014157
ENSODEG00000014158
ENSODEG00000014159
ENSODEG00000014160
ENSODEG00000014161
ENSODEG00000014162
ENSODEG00000014164
ENSODEG00000014169
ENSODEG00000014170
ENSODEG00000014171
ENSODEG00000014172
ENSODEG00000014174
ENSODEG00000014175
ENSODEG00000014176
ENSODEG00000014177
ENSODEG00000014178
ENSODEG00000014179
ENSODEG00000014180
ENSODEG00000014181
ENSODEG00000014182
ENSODEG00000014184
ENSODEG00000014185
ENSODEG00000014187
ENSODEG00000014190
ENSODEG00000014191
ENSODEG00000014192
ENSODEG00000014194
ENSODEG00000014195
ENSODEG00000014196

ENSODEG00000014197
ENSODEG00000014200
ENSODEG00000014203
ENSODEG00000014204
ENSODEG00000014205
ENSODEG00000014206
ENSODEG00000014207
ENSODEG00000014208
ENSODEG00000014209
ENSODEG00000014210
ENSODEG00000014211
ENSODEG00000014212
ENSODEG00000014214
ENSODEG00000014216
ENSODEG00000014217
ENSODEG00000014221
ENSODEG00000014224
ENSODEG00000014225
ENSODEG00000014226
ENSODEG00000014228
ENSODEG00000014229
ENSODEG00000014231
ENSODEG00000014232
ENSODEG00000014234
ENSODEG00000014235
ENSODEG00000014236
ENSODEG00000014237
ENSODEG00000014238
ENSODEG00000014239
ENSODEG00000014240
ENSODEG00000014242
ENSODEG00000014245
ENSODEG00000014246
ENSODEG00000014247
ENSODEG00000014248
ENSODEG00000014249
ENSODEG00000014250
ENSODEG00000014251
ENSODEG00000014252
ENSODEG00000014253
ENSODEG00000014254
ENSODEG00000014257
ENSODEG00000014258
ENSODEG00000014259
ENSODEG00000014260
ENSODEG00000014262
ENSODEG00000014263
ENSODEG00000014265
ENSODEG00000014267
ENSODEG00000014268
ENSODEG00000014271
ENSODEG00000014272
ENSODEG00000014274
ENSODEG00000014275
ENSODEG00000014276
ENSODEG00000014277
ENSODEG00000014278
ENSODEG00000014279
ENSODEG00000014281

ENSODEG00000014283
ENSODEG00000014284
ENSODEG00000014285
ENSODEG00000014286
ENSODEG00000014287
ENSODEG00000014288
ENSODEG00000014289
ENSODEG00000014292
ENSODEG00000014293
ENSODEG00000014294
ENSODEG00000014295
ENSODEG00000014296
ENSODEG00000014298
ENSODEG00000014299
ENSODEG00000014301
ENSODEG00000014302
ENSODEG00000014303
ENSODEG00000014305
ENSODEG00000014307
ENSODEG00000014308
ENSODEG00000014309
ENSODEG00000014310
ENSODEG00000014311
ENSODEG00000014313
ENSODEG00000014315
ENSODEG00000014317
ENSODEG00000014319
ENSODEG00000014320
ENSODEG00000014322
ENSODEG00000014323
ENSODEG00000014324
ENSODEG00000014325
ENSODEG00000014326
ENSODEG00000014327
ENSODEG00000014328
ENSODEG00000014329
ENSODEG00000014330
ENSODEG00000014331
ENSODEG00000014332
ENSODEG00000014333
ENSODEG00000014334
ENSODEG00000014335
ENSODEG00000014336
ENSODEG00000014337
ENSODEG00000014338
ENSODEG00000014339
ENSODEG00000014340
ENSODEG00000014341
ENSODEG00000014343
ENSODEG00000014344
ENSODEG00000014345
ENSODEG00000014346
ENSODEG00000014347
ENSODEG00000014348
ENSODEG00000014349
ENSODEG00000014351
ENSODEG00000014352
ENSODEG00000014354
ENSODEG00000014355

ENSODEG00000014357
ENSODEG00000014358
ENSODEG00000014359
ENSODEG00000014360
ENSODEG00000014361
ENSODEG00000014362
ENSODEG00000014364
ENSODEG00000014365
ENSODEG00000014366
ENSODEG00000014367
ENSODEG00000014368
ENSODEG00000014369
ENSODEG00000014370
ENSODEG00000014371
ENSODEG00000014373
ENSODEG00000014374
ENSODEG00000014376
ENSODEG00000014377
ENSODEG00000014380
ENSODEG00000014381
ENSODEG00000014382
ENSODEG00000014383
ENSODEG00000014384
ENSODEG00000014385
ENSODEG00000014386
ENSODEG00000014387
ENSODEG00000014388
ENSODEG00000014390
ENSODEG00000014392
ENSODEG00000014393
ENSODEG00000014394
ENSODEG00000014395
ENSODEG00000014397
ENSODEG00000014398
ENSODEG00000014399
ENSODEG00000014400
ENSODEG00000014401
ENSODEG00000014402
ENSODEG00000014403
ENSODEG00000014404
ENSODEG00000014405
ENSODEG00000014406
ENSODEG00000014407
ENSODEG00000014408
ENSODEG00000014409
ENSODEG00000014410
ENSODEG00000014411
ENSODEG00000014412
ENSODEG00000014413
ENSODEG00000014414
ENSODEG00000014416
ENSODEG00000014418
ENSODEG00000014420
ENSODEG00000014421
ENSODEG00000014423
ENSODEG00000014424
ENSODEG00000014425
ENSODEG00000014426
ENSODEG00000014427

ENSODEG00000014428
ENSODEG00000014429
ENSODEG00000014431
ENSODEG00000014432
ENSODEG00000014434
ENSODEG00000014435
ENSODEG00000014438
ENSODEG00000014439
ENSODEG00000014440
ENSODEG00000014441
ENSODEG00000014443
ENSODEG00000014444
ENSODEG00000014445
ENSODEG00000014446
ENSODEG00000014449
ENSODEG00000014450
ENSODEG00000014452
ENSODEG00000014453
ENSODEG00000014454
ENSODEG00000014455
ENSODEG00000014456
ENSODEG00000014458
ENSODEG00000014459
ENSODEG00000014461
ENSODEG00000014462
ENSODEG00000014463
ENSODEG00000014464
ENSODEG00000014465
ENSODEG00000014466
ENSODEG00000014467
ENSODEG00000014468
ENSODEG00000014469
ENSODEG00000014471
ENSODEG00000014473
ENSODEG00000014474
ENSODEG00000014475
ENSODEG00000014477
ENSODEG00000014478
ENSODEG00000014479
ENSODEG00000014480
ENSODEG00000014481
ENSODEG00000014483
ENSODEG00000014484
ENSODEG00000014485
ENSODEG00000014486
ENSODEG00000014488
ENSODEG00000014489
ENSODEG00000014490
ENSODEG00000014491
ENSODEG00000014492
ENSODEG00000014494
ENSODEG00000014495
ENSODEG00000014496
ENSODEG00000014497
ENSODEG00000014498
ENSODEG00000014503
ENSODEG00000014505
ENSODEG00000014506
ENSODEG00000014507

ENSODEG00000014508
ENSODEG00000014509
ENSODEG00000014510
ENSODEG00000014511
ENSODEG00000014512
ENSODEG00000014513
ENSODEG00000014514
ENSODEG00000014516
ENSODEG00000014518
ENSODEG00000014519
ENSODEG00000014520
ENSODEG00000014521
ENSODEG00000014523
ENSODEG00000014526
ENSODEG00000014531
ENSODEG00000014532
ENSODEG00000014533
ENSODEG00000014534
ENSODEG00000014535
ENSODEG00000014537
ENSODEG00000014538
ENSODEG00000014539
ENSODEG00000014540
ENSODEG00000014541
ENSODEG00000014542
ENSODEG00000014543
ENSODEG00000014544
ENSODEG00000014545
ENSODEG00000014546
ENSODEG00000014548
ENSODEG00000014549
ENSODEG00000014551
ENSODEG00000014552
ENSODEG00000014553
ENSODEG00000014555
ENSODEG00000014557
ENSODEG00000014558
ENSODEG00000014559
ENSODEG00000014560
ENSODEG00000014561
ENSODEG00000014562
ENSODEG00000014563
ENSODEG00000014564
ENSODEG00000014565
ENSODEG00000014566
ENSODEG00000014568
ENSODEG00000014569
ENSODEG00000014571
ENSODEG00000014572
ENSODEG00000014573
ENSODEG00000014574
ENSODEG00000014575
ENSODEG00000014576
ENSODEG00000014577
ENSODEG00000014578
ENSODEG00000014580
ENSODEG00000014582
ENSODEG00000014583
ENSODEG00000014584

ENSODEG00000014585
ENSODEG00000014586
ENSODEG00000014587
ENSODEG00000014588
ENSODEG00000014589
ENSODEG00000014591
ENSODEG00000014593
ENSODEG00000014594
ENSODEG00000014595
ENSODEG00000014597
ENSODEG00000014599
ENSODEG00000014600
ENSODEG00000014604
ENSODEG00000014605
ENSODEG00000014606
ENSODEG00000014608
ENSODEG00000014609
ENSODEG00000014613
ENSODEG00000014615
ENSODEG00000014616
ENSODEG00000014618
ENSODEG00000014619
ENSODEG00000014620
ENSODEG00000014621
ENSODEG00000014622
ENSODEG00000014623
ENSODEG00000014625
ENSODEG00000014626
ENSODEG00000014627
ENSODEG00000014628
ENSODEG00000014629
ENSODEG00000014630
ENSODEG00000014631
ENSODEG00000014632
ENSODEG00000014633
ENSODEG00000014634
ENSODEG00000014636
ENSODEG00000014637
ENSODEG00000014641
ENSODEG00000014642
ENSODEG00000014643
ENSODEG00000014644
ENSODEG00000014645
ENSODEG00000014647
ENSODEG00000014650
ENSODEG00000014651
ENSODEG00000014652
ENSODEG00000014653
ENSODEG00000014654
ENSODEG00000014655
ENSODEG00000014656
ENSODEG00000014657
ENSODEG00000014658
ENSODEG00000014661
ENSODEG00000014662
ENSODEG00000014664
ENSODEG00000014665
ENSODEG00000014666
ENSODEG00000014667

ENSODEG00000014668
ENSODEG00000014669
ENSODEG00000014671
ENSODEG00000014673
ENSODEG00000014674
ENSODEG00000014675
ENSODEG00000014676
ENSODEG00000014677
ENSODEG00000014678
ENSODEG00000014679
ENSODEG00000014683
ENSODEG00000014684
ENSODEG00000014685
ENSODEG00000014687
ENSODEG00000014688
ENSODEG00000014689
ENSODEG00000014690
ENSODEG00000014691
ENSODEG00000014692
ENSODEG00000014693
ENSODEG00000014694
ENSODEG00000014695
ENSODEG00000014696
ENSODEG00000014697
ENSODEG00000014698
ENSODEG00000014699
ENSODEG00000014700
ENSODEG00000014702
ENSODEG00000014703
ENSODEG00000014704
ENSODEG00000014705
ENSODEG00000014706
ENSODEG00000014709
ENSODEG00000014710
ENSODEG00000014711
ENSODEG00000014712
ENSODEG00000014713
ENSODEG00000014714
ENSODEG00000014715
ENSODEG00000014717
ENSODEG00000014718
ENSODEG00000014719
ENSODEG00000014721
ENSODEG00000014722
ENSODEG00000014723
ENSODEG00000014725
ENSODEG00000014726
ENSODEG00000014727
ENSODEG00000014728
ENSODEG00000014729
ENSODEG00000014730
ENSODEG00000014732
ENSODEG00000014733
ENSODEG00000014734
ENSODEG00000014736
ENSODEG00000014738
ENSODEG00000014739
ENSODEG00000014740
ENSODEG00000014741

ENSODEG00000014742
ENSODEG00000014745
ENSODEG00000014746
ENSODEG00000014748
ENSODEG00000014749
ENSODEG00000014750
ENSODEG00000014751
ENSODEG00000014752
ENSODEG00000014754
ENSODEG00000014755
ENSODEG00000014756
ENSODEG00000014758
ENSODEG00000014760
ENSODEG00000014761
ENSODEG00000014763
ENSODEG00000014764
ENSODEG00000014765
ENSODEG00000014766
ENSODEG00000014767
ENSODEG00000014768
ENSODEG00000014769
ENSODEG00000014771
ENSODEG00000014772
ENSODEG00000014774
ENSODEG00000014775
ENSODEG00000014776
ENSODEG00000014779
ENSODEG00000014780
ENSODEG00000014781
ENSODEG00000014782
ENSODEG00000014783
ENSODEG00000014786
ENSODEG00000014787
ENSODEG00000014789
ENSODEG00000014790
ENSODEG00000014792
ENSODEG00000014796
ENSODEG00000014797
ENSODEG00000014799
ENSODEG00000014800
ENSODEG00000014801
ENSODEG00000014802
ENSODEG00000014803
ENSODEG00000014805
ENSODEG00000014808
ENSODEG00000014809
ENSODEG00000014810
ENSODEG00000014812
ENSODEG00000014813
ENSODEG00000014815
ENSODEG00000014816
ENSODEG00000014818
ENSODEG00000014819
ENSODEG00000014821
ENSODEG00000014822
ENSODEG00000014823
ENSODEG00000014824
ENSODEG00000014825
ENSODEG00000014826

ENSODEG00000014827
ENSODEG00000014828
ENSODEG00000014829
ENSODEG00000014830
ENSODEG00000014831
ENSODEG00000014832
ENSODEG00000014833
ENSODEG00000014834
ENSODEG00000014835
ENSODEG00000014836
ENSODEG00000014838
ENSODEG00000014840
ENSODEG00000014843
ENSODEG00000014844
ENSODEG00000014845
ENSODEG00000014846
ENSODEG00000014848
ENSODEG00000014849
ENSODEG00000014851
ENSODEG00000014852
ENSODEG00000014856
ENSODEG00000014857
ENSODEG00000014858
ENSODEG00000014859
ENSODEG00000014860
ENSODEG00000014862
ENSODEG00000014863
ENSODEG00000014864
ENSODEG00000014865
ENSODEG00000014866
ENSODEG00000014867
ENSODEG00000014869
ENSODEG00000014870
ENSODEG00000014871
ENSODEG00000014872
ENSODEG00000014874
ENSODEG00000014875
ENSODEG00000014876
ENSODEG00000014877
ENSODEG00000014878
ENSODEG00000014880
ENSODEG00000014882
ENSODEG00000014885
ENSODEG00000014887
ENSODEG00000014888
ENSODEG00000014889
ENSODEG00000014890
ENSODEG00000014891
ENSODEG00000014893
ENSODEG00000014894
ENSODEG00000014896
ENSODEG00000014897
ENSODEG00000014898
ENSODEG00000014899
ENSODEG00000014900
ENSODEG00000014901
ENSODEG00000014902
ENSODEG00000014903
ENSODEG00000014904

ENSODEG00000014905
ENSODEG00000014906
ENSODEG00000014908
ENSODEG00000014909
ENSODEG00000014910
ENSODEG00000014912
ENSODEG00000014913
ENSODEG00000014914
ENSODEG00000014915
ENSODEG00000014916
ENSODEG00000014917
ENSODEG00000014918
ENSODEG00000014919
ENSODEG00000014920
ENSODEG00000014921
ENSODEG00000014922
ENSODEG00000014924
ENSODEG00000014926
ENSODEG00000014927
ENSODEG00000014928
ENSODEG00000014929
ENSODEG00000014931
ENSODEG00000014932
ENSODEG00000014933
ENSODEG00000014934
ENSODEG00000014935
ENSODEG00000014936
ENSODEG00000014937
ENSODEG00000014938
ENSODEG00000014939
ENSODEG00000014940
ENSODEG00000014941
ENSODEG00000014942
ENSODEG00000014943
ENSODEG00000014944
ENSODEG00000014945
ENSODEG00000014946
ENSODEG00000014947
ENSODEG00000014948
ENSODEG00000014949
ENSODEG00000014950
ENSODEG00000014952
ENSODEG00000014953
ENSODEG00000014954
ENSODEG00000014956
ENSODEG00000014957
ENSODEG00000014958
ENSODEG00000014959
ENSODEG00000014960
ENSODEG00000014961
ENSODEG00000014962
ENSODEG00000014964
ENSODEG00000014965
ENSODEG00000014966
ENSODEG00000014967
ENSODEG00000014968
ENSODEG00000014969
ENSODEG00000014970
ENSODEG00000014971

ENSODEG00000014972
ENSODEG00000014973
ENSODEG00000014974
ENSODEG00000014975
ENSODEG00000014979
ENSODEG00000014980
ENSODEG00000014982
ENSODEG00000014983
ENSODEG00000014984
ENSODEG00000014985
ENSODEG00000014986
ENSODEG00000014987
ENSODEG00000014989
ENSODEG00000014990
ENSODEG00000014991
ENSODEG00000014992
ENSODEG00000014993
ENSODEG00000014994
ENSODEG00000014996
ENSODEG00000014999
ENSODEG00000015000
ENSODEG00000015001
ENSODEG00000015002
ENSODEG00000015003
ENSODEG00000015004
ENSODEG00000015005
ENSODEG00000015006
ENSODEG00000015007
ENSODEG00000015008
ENSODEG00000015009
ENSODEG00000015010
ENSODEG00000015011
ENSODEG00000015013
ENSODEG00000015014
ENSODEG00000015015
ENSODEG00000015016
ENSODEG00000015019
ENSODEG00000015020
ENSODEG00000015021
ENSODEG00000015024
ENSODEG00000015025
ENSODEG00000015026
ENSODEG00000015027
ENSODEG00000015028
ENSODEG00000015029
ENSODEG00000015030
ENSODEG00000015034
ENSODEG00000015035
ENSODEG00000015036
ENSODEG00000015037
ENSODEG00000015039
ENSODEG00000015041
ENSODEG00000015045
ENSODEG00000015047
ENSODEG00000015048
ENSODEG00000015049
ENSODEG00000015050
ENSODEG00000015051
ENSODEG00000015052

ENSODEG00000015054
ENSODEG00000015055
ENSODEG00000015058
ENSODEG00000015059
ENSODEG00000015060
ENSODEG00000015061
ENSODEG00000015062
ENSODEG00000015063
ENSODEG00000015064
ENSODEG00000015065
ENSODEG00000015067
ENSODEG00000015068
ENSODEG00000015069
ENSODEG00000015070
ENSODEG00000015071
ENSODEG00000015072
ENSODEG00000015073
ENSODEG00000015074
ENSODEG00000015075
ENSODEG00000015076
ENSODEG00000015077
ENSODEG00000015079
ENSODEG00000015081
ENSODEG00000015082
ENSODEG00000015083
ENSODEG00000015085
ENSODEG00000015086
ENSODEG00000015087
ENSODEG00000015088
ENSODEG00000015090
ENSODEG00000015091
ENSODEG00000015092
ENSODEG00000015094
ENSODEG00000015095
ENSODEG00000015096
ENSODEG00000015097
ENSODEG00000015098
ENSODEG00000015099
ENSODEG00000015100
ENSODEG00000015101
ENSODEG00000015102
ENSODEG00000015104
ENSODEG00000015106
ENSODEG00000015107
ENSODEG00000015108
ENSODEG00000015109
ENSODEG00000015110
ENSODEG00000015112
ENSODEG00000015114
ENSODEG00000015115
ENSODEG00000015116
ENSODEG00000015117
ENSODEG00000015120
ENSODEG00000015121
ENSODEG00000015122
ENSODEG00000015123
ENSODEG00000015124
ENSODEG00000015125
ENSODEG00000015126

ENSODEG00000015128
ENSODEG00000015129
ENSODEG00000015130
ENSODEG00000015131
ENSODEG00000015132
ENSODEG00000015133
ENSODEG00000015134
ENSODEG00000015137
ENSODEG00000015138
ENSODEG00000015139
ENSODEG00000015140
ENSODEG00000015141
ENSODEG00000015142
ENSODEG00000015143
ENSODEG00000015146
ENSODEG00000015147
ENSODEG00000015148
ENSODEG00000015149
ENSODEG00000015152
ENSODEG00000015153
ENSODEG00000015154
ENSODEG00000015155
ENSODEG00000015156
ENSODEG00000015157
ENSODEG00000015158
ENSODEG00000015159
ENSODEG00000015160
ENSODEG00000015161
ENSODEG00000015162
ENSODEG00000015167
ENSODEG00000015168
ENSODEG00000015169
ENSODEG00000015171
ENSODEG00000015172
ENSODEG00000015174
ENSODEG00000015177
ENSODEG00000015178
ENSODEG00000015180
ENSODEG00000015181
ENSODEG00000015182
ENSODEG00000015184
ENSODEG00000015186
ENSODEG00000015189
ENSODEG00000015190
ENSODEG00000015191
ENSODEG00000015192
ENSODEG00000015193
ENSODEG00000015194
ENSODEG00000015195
ENSODEG00000015197
ENSODEG00000015199
ENSODEG00000015200
ENSODEG00000015201
ENSODEG00000015202
ENSODEG00000015206
ENSODEG00000015207
ENSODEG00000015208
ENSODEG00000015209
ENSODEG00000015210

ENSODEG00000015213
ENSODEG00000015214
ENSODEG00000015215
ENSODEG00000015216
ENSODEG00000015218
ENSODEG00000015219
ENSODEG00000015220
ENSODEG00000015221
ENSODEG00000015222
ENSODEG00000015223
ENSODEG00000015224
ENSODEG00000015226
ENSODEG00000015227
ENSODEG00000015228
ENSODEG00000015229
ENSODEG00000015237
ENSODEG00000015239
ENSODEG00000015241
ENSODEG00000015242
ENSODEG00000015244
ENSODEG00000015245
ENSODEG00000015247
ENSODEG00000015248
ENSODEG00000015250
ENSODEG00000015251
ENSODEG00000015252
ENSODEG00000015254
ENSODEG00000015255
ENSODEG00000015256
ENSODEG00000015257
ENSODEG00000015258
ENSODEG00000015260
ENSODEG00000015261
ENSODEG00000015262
ENSODEG00000015263
ENSODEG00000015264
ENSODEG00000015265
ENSODEG00000015266
ENSODEG00000015268
ENSODEG00000015269
ENSODEG00000015270
ENSODEG00000015271
ENSODEG00000015272
ENSODEG00000015273
ENSODEG00000015274
ENSODEG00000015275
ENSODEG00000015276
ENSODEG00000015278
ENSODEG00000015279
ENSODEG00000015282
ENSODEG00000015283
ENSODEG00000015285
ENSODEG00000015286
ENSODEG00000015287
ENSODEG00000015288
ENSODEG00000015289
ENSODEG00000015293
ENSODEG00000015296
ENSODEG00000015298

ENSODEG00000015300
ENSODEG00000015301
ENSODEG00000015302
ENSODEG00000015303
ENSODEG00000015306
ENSODEG00000015307
ENSODEG00000015309
ENSODEG00000015310
ENSODEG00000015311
ENSODEG00000015312
ENSODEG00000015313
ENSODEG00000015314
ENSODEG00000015317
ENSODEG00000015318
ENSODEG00000015319
ENSODEG00000015320
ENSODEG00000015322
ENSODEG00000015323
ENSODEG00000015324
ENSODEG00000015325
ENSODEG00000015326
ENSODEG00000015327
ENSODEG00000015328
ENSODEG00000015329
ENSODEG00000015330
ENSODEG00000015333
ENSODEG00000015335
ENSODEG00000015336
ENSODEG00000015337
ENSODEG00000015338
ENSODEG00000015339
ENSODEG00000015340
ENSODEG00000015341
ENSODEG00000015342
ENSODEG00000015345
ENSODEG00000015346
ENSODEG00000015347
ENSODEG00000015350
ENSODEG00000015352
ENSODEG00000015353
ENSODEG00000015355
ENSODEG00000015356
ENSODEG00000015357
ENSODEG00000015358
ENSODEG00000015361
ENSODEG00000015362
ENSODEG00000015363
ENSODEG00000015364
ENSODEG00000015365
ENSODEG00000015367
ENSODEG00000015370
ENSODEG00000015371
ENSODEG00000015372
ENSODEG00000015373
ENSODEG00000015374
ENSODEG00000015375
ENSODEG00000015376
ENSODEG00000015377
ENSODEG00000015378

ENSODEG00000015379
ENSODEG00000015380
ENSODEG00000015381
ENSODEG00000015383
ENSODEG00000015385
ENSODEG00000015387
ENSODEG00000015388
ENSODEG00000015389
ENSODEG00000015390
ENSODEG00000015392
ENSODEG00000015393
ENSODEG00000015394
ENSODEG00000015395
ENSODEG00000015396
ENSODEG00000015397
ENSODEG00000015398
ENSODEG00000015400
ENSODEG00000015401
ENSODEG00000015402
ENSODEG00000015403
ENSODEG00000015404
ENSODEG00000015405
ENSODEG00000015406
ENSODEG00000015408
ENSODEG00000015410
ENSODEG00000015411
ENSODEG00000015412
ENSODEG00000015413
ENSODEG00000015414
ENSODEG00000015415
ENSODEG00000015416
ENSODEG00000015418
ENSODEG00000015420
ENSODEG00000015422
ENSODEG00000015423
ENSODEG00000015427
ENSODEG00000015428
ENSODEG00000015429
ENSODEG00000015430
ENSODEG00000015432
ENSODEG00000015433
ENSODEG00000015434
ENSODEG00000015435
ENSODEG00000015437
ENSODEG00000015438
ENSODEG00000015439
ENSODEG00000015440
ENSODEG00000015441
ENSODEG00000015442
ENSODEG00000015444
ENSODEG00000015445
ENSODEG00000015446
ENSODEG00000015448
ENSODEG00000015449
ENSODEG00000015450
ENSODEG00000015451
ENSODEG00000015455
ENSODEG00000015456
ENSODEG00000015457

ENSODEG00000015459
ENSODEG00000015460
ENSODEG00000015461
ENSODEG00000015462
ENSODEG00000015464
ENSODEG00000015467
ENSODEG00000015468
ENSODEG00000015469
ENSODEG00000015475
ENSODEG00000015476
ENSODEG00000015478
ENSODEG00000015479
ENSODEG00000015480
ENSODEG00000015481
ENSODEG00000015482
ENSODEG00000015483
ENSODEG00000015484
ENSODEG00000015486
ENSODEG00000015487
ENSODEG00000015488
ENSODEG00000015489
ENSODEG00000015491
ENSODEG00000015492
ENSODEG00000015493
ENSODEG00000015496
ENSODEG00000015497
ENSODEG00000015499
ENSODEG00000015501
ENSODEG00000015502
ENSODEG00000015503
ENSODEG00000015506
ENSODEG00000015507
ENSODEG00000015510
ENSODEG00000015511
ENSODEG00000015512
ENSODEG00000015513
ENSODEG00000015514
ENSODEG00000015515
ENSODEG00000015517
ENSODEG00000015519
ENSODEG00000015522
ENSODEG00000015524
ENSODEG00000015525
ENSODEG00000015527
ENSODEG00000015529
ENSODEG00000015530
ENSODEG00000015531
ENSODEG00000015533
ENSODEG00000015534
ENSODEG00000015537
ENSODEG00000015538
ENSODEG00000015539
ENSODEG00000015540
ENSODEG00000015542
ENSODEG00000015543
ENSODEG00000015544
ENSODEG00000015545
ENSODEG00000015548
ENSODEG00000015549

ENSODEG00000015550
ENSODEG00000015551
ENSODEG00000015553
ENSODEG00000015554
ENSODEG00000015555
ENSODEG00000015556
ENSODEG00000015557
ENSODEG00000015559
ENSODEG00000015560
ENSODEG00000015561
ENSODEG00000015563
ENSODEG00000015564
ENSODEG00000015565
ENSODEG00000015567
ENSODEG00000015568
ENSODEG00000015570
ENSODEG00000015571
ENSODEG00000015572
ENSODEG00000015573
ENSODEG00000015575
ENSODEG00000015576
ENSODEG00000015577
ENSODEG00000015579
ENSODEG00000015580
ENSODEG00000015581
ENSODEG00000015582
ENSODEG00000015583
ENSODEG00000015587
ENSODEG00000015589
ENSODEG00000015590
ENSODEG00000015591
ENSODEG00000015592
ENSODEG00000015593
ENSODEG00000015594
ENSODEG00000015596
ENSODEG00000015598
ENSODEG00000015601
ENSODEG00000015602
ENSODEG00000015603
ENSODEG00000015604
ENSODEG00000015606
ENSODEG00000015610
ENSODEG00000015611
ENSODEG00000015613
ENSODEG00000015614
ENSODEG00000015615
ENSODEG00000015617
ENSODEG00000015618
ENSODEG00000015619
ENSODEG00000015621
ENSODEG00000015622
ENSODEG00000015623
ENSODEG00000015624
ENSODEG00000015626
ENSODEG00000015628
ENSODEG00000015629
ENSODEG00000015630
ENSODEG00000015631
ENSODEG00000015635

ENSODEG00000015636
ENSODEG00000015637
ENSODEG00000015638
ENSODEG00000015639
ENSODEG00000015640
ENSODEG00000015642
ENSODEG00000015643
ENSODEG00000015644
ENSODEG00000015645
ENSODEG00000015646
ENSODEG00000015647
ENSODEG00000015648
ENSODEG00000015651
ENSODEG00000015652
ENSODEG00000015653
ENSODEG00000015654
ENSODEG00000015655
ENSODEG00000015656
ENSODEG00000015657
ENSODEG00000015658
ENSODEG00000015659
ENSODEG00000015660
ENSODEG00000015661
ENSODEG00000015662
ENSODEG00000015664
ENSODEG00000015666
ENSODEG00000015667
ENSODEG00000015670
ENSODEG00000015671
ENSODEG00000015672
ENSODEG00000015673
ENSODEG00000015674
ENSODEG00000015676
ENSODEG00000015677
ENSODEG00000015678
ENSODEG00000015681
ENSODEG00000015682
ENSODEG00000015684
ENSODEG00000015685
ENSODEG00000015686
ENSODEG00000015687
ENSODEG00000015689
ENSODEG00000015690
ENSODEG00000015692
ENSODEG00000015693
ENSODEG00000015694
ENSODEG00000015695
ENSODEG00000015696
ENSODEG00000015697
ENSODEG00000015700
ENSODEG00000015701
ENSODEG00000015702
ENSODEG00000015704
ENSODEG00000015705
ENSODEG00000015707
ENSODEG00000015709
ENSODEG00000015710
ENSODEG00000015711
ENSODEG00000015712

ENSODEG00000015713
ENSODEG00000015714
ENSODEG00000015715
ENSODEG00000015716
ENSODEG00000015717
ENSODEG00000015718
ENSODEG00000015719
ENSODEG00000015720
ENSODEG00000015721
ENSODEG00000015722
ENSODEG00000015724
ENSODEG00000015725
ENSODEG00000015726
ENSODEG00000015727
ENSODEG00000015728
ENSODEG00000015729
ENSODEG00000015730
ENSODEG00000015731
ENSODEG00000015732
ENSODEG00000015734
ENSODEG00000015736
ENSODEG00000015737
ENSODEG00000015738
ENSODEG00000015742
ENSODEG00000015744
ENSODEG00000015745
ENSODEG00000015746
ENSODEG00000015747
ENSODEG00000015748
ENSODEG00000015749
ENSODEG00000015750
ENSODEG00000015751
ENSODEG00000015753
ENSODEG00000015754
ENSODEG00000015756
ENSODEG00000015757
ENSODEG00000015758
ENSODEG00000015759
ENSODEG00000015761
ENSODEG00000015762
ENSODEG00000015763
ENSODEG00000015766
ENSODEG00000015767
ENSODEG00000015768
ENSODEG00000015769
ENSODEG00000015770
ENSODEG00000015771
ENSODEG00000015775
ENSODEG00000015777
ENSODEG00000015778
ENSODEG00000015779
ENSODEG00000015781
ENSODEG00000015782
ENSODEG00000015784
ENSODEG00000015787
ENSODEG00000015788
ENSODEG00000015789
ENSODEG00000015791
ENSODEG00000015794

ENSODEG00000015796
ENSODEG00000015798
ENSODEG00000015800
ENSODEG00000015804
ENSODEG00000015805
ENSODEG00000015807
ENSODEG00000015808
ENSODEG00000015810
ENSODEG00000015811
ENSODEG00000015812
ENSODEG00000015814
ENSODEG00000015815
ENSODEG00000015817
ENSODEG00000015819
ENSODEG00000015820
ENSODEG00000015821
ENSODEG00000015822
ENSODEG00000015823
ENSODEG00000015825
ENSODEG00000015827
ENSODEG00000015828
ENSODEG00000015829
ENSODEG00000015830
ENSODEG00000015831
ENSODEG00000015832
ENSODEG00000015833
ENSODEG00000015834
ENSODEG00000015836
ENSODEG00000015837
ENSODEG00000015838
ENSODEG00000015839
ENSODEG00000015840
ENSODEG00000015841
ENSODEG00000015842
ENSODEG00000015844
ENSODEG00000015845
ENSODEG00000015847
ENSODEG00000015848
ENSODEG00000015851
ENSODEG00000015852
ENSODEG00000015853
ENSODEG00000015855
ENSODEG00000015856
ENSODEG00000015858
ENSODEG00000015860
ENSODEG00000015862
ENSODEG00000015865
ENSODEG00000015866
ENSODEG00000015867
ENSODEG00000015868
ENSODEG00000015870
ENSODEG00000015871
ENSODEG00000015872
ENSODEG00000015873
ENSODEG00000015874
ENSODEG00000015875
ENSODEG00000015876
ENSODEG00000015877
ENSODEG00000015878

ENSODEG00000015880
ENSODEG00000015881
ENSODEG00000015884
ENSODEG00000015885
ENSODEG00000015886
ENSODEG00000015887
ENSODEG00000015889
ENSODEG00000015890
ENSODEG00000015891
ENSODEG00000015893
ENSODEG00000015895
ENSODEG00000015896
ENSODEG00000015897
ENSODEG00000015898
ENSODEG00000015899
ENSODEG00000015900
ENSODEG00000015902
ENSODEG00000015903
ENSODEG00000015904
ENSODEG00000015905
ENSODEG00000015907
ENSODEG00000015908
ENSODEG00000015909
ENSODEG00000015910
ENSODEG00000015912
ENSODEG00000015914
ENSODEG00000015915
ENSODEG00000015917
ENSODEG00000015918
ENSODEG00000015919
ENSODEG00000015920
ENSODEG00000015922
ENSODEG00000015923
ENSODEG00000015924
ENSODEG00000015925
ENSODEG00000015927
ENSODEG00000015928
ENSODEG00000015929
ENSODEG00000015932
ENSODEG00000015933
ENSODEG00000015934
ENSODEG00000015935
ENSODEG00000015937
ENSODEG00000015939
ENSODEG00000015940
ENSODEG00000015942
ENSODEG00000015943
ENSODEG00000015944
ENSODEG00000015945
ENSODEG00000015946
ENSODEG00000015947
ENSODEG00000015948
ENSODEG00000015949
ENSODEG00000015951
ENSODEG00000015953
ENSODEG00000015954
ENSODEG00000015956
ENSODEG00000015957
ENSODEG00000015958

ENSODEG00000015960
ENSODEG00000015961
ENSODEG00000015962
ENSODEG00000015963
ENSODEG00000015964
ENSODEG00000015966
ENSODEG00000015967
ENSODEG00000015968
ENSODEG00000015970
ENSODEG00000015971
ENSODEG00000015972
ENSODEG00000015974
ENSODEG00000015976
ENSODEG00000015977
ENSODEG00000015981
ENSODEG00000015982
ENSODEG00000015983
ENSODEG00000015985
ENSODEG00000015986
ENSODEG00000015987
ENSODEG00000015988
ENSODEG00000015989
ENSODEG00000015990
ENSODEG00000015991
ENSODEG00000015992
ENSODEG00000015993
ENSODEG00000015994
ENSODEG00000015995
ENSODEG00000015996
ENSODEG00000015997
ENSODEG00000015999
ENSODEG00000016000
ENSODEG00000016002
ENSODEG00000016004
ENSODEG00000016005
ENSODEG00000016006
ENSODEG00000016007
ENSODEG00000016008
ENSODEG00000016009
ENSODEG00000016010
ENSODEG00000016011
ENSODEG00000016012
ENSODEG00000016013
ENSODEG00000016014
ENSODEG00000016015
ENSODEG00000016016
ENSODEG00000016017
ENSODEG00000016018
ENSODEG00000016019
ENSODEG00000016020
ENSODEG00000016021
ENSODEG00000016022
ENSODEG00000016023
ENSODEG00000016024
ENSODEG00000016025
ENSODEG00000016026
ENSODEG00000016028
ENSODEG00000016030
ENSODEG00000016031

ENSODEG00000016032
ENSODEG00000016033
ENSODEG00000016034
ENSODEG00000016036
ENSODEG00000016037
ENSODEG00000016038
ENSODEG00000016041
ENSODEG00000016042
ENSODEG00000016043
ENSODEG00000016044
ENSODEG00000016045
ENSODEG00000016046
ENSODEG00000016047
ENSODEG00000016050
ENSODEG00000016051
ENSODEG00000016052
ENSODEG00000016053
ENSODEG00000016054
ENSODEG00000016055
ENSODEG00000016056
ENSODEG00000016057
ENSODEG00000016059
ENSODEG00000016060
ENSODEG00000016061
ENSODEG00000016062
ENSODEG00000016063
ENSODEG00000016064
ENSODEG00000016065
ENSODEG00000016067
ENSODEG00000016068
ENSODEG00000016069
ENSODEG00000016070
ENSODEG00000016071
ENSODEG00000016073
ENSODEG00000016074
ENSODEG00000016076
ENSODEG00000016078
ENSODEG00000016079
ENSODEG00000016082
ENSODEG00000016083
ENSODEG00000016084
ENSODEG00000016085
ENSODEG00000016086
ENSODEG00000016087
ENSODEG00000016090
ENSODEG00000016091
ENSODEG00000016092
ENSODEG00000016094
ENSODEG00000016095
ENSODEG00000016096
ENSODEG00000016097
ENSODEG00000016098
ENSODEG00000016099
ENSODEG00000016100
ENSODEG00000016101
ENSODEG00000016102
ENSODEG00000016103
ENSODEG00000016104
ENSODEG00000016105

ENSODEG00000016107
ENSODEG00000016108
ENSODEG00000016109
ENSODEG00000016110
ENSODEG00000016111
ENSODEG00000016112
ENSODEG00000016113
ENSODEG00000016114
ENSODEG00000016115
ENSODEG00000016116
ENSODEG00000016117
ENSODEG00000016118
ENSODEG00000016119
ENSODEG00000016120
ENSODEG00000016121
ENSODEG00000016122
ENSODEG00000016123
ENSODEG00000016124
ENSODEG00000016125
ENSODEG00000016126
ENSODEG00000016127
ENSODEG00000016129
ENSODEG00000016130
ENSODEG00000016131
ENSODEG00000016132
ENSODEG00000016133
ENSODEG00000016134
ENSODEG00000016135
ENSODEG00000016137
ENSODEG00000016138
ENSODEG00000016139
ENSODEG00000016141
ENSODEG00000016144
ENSODEG00000016145
ENSODEG00000016147
ENSODEG00000016148
ENSODEG00000016152
ENSODEG00000016153
ENSODEG00000016154
ENSODEG00000016157
ENSODEG00000016159
ENSODEG00000016161
ENSODEG00000016165
ENSODEG00000016166
ENSODEG00000016167
ENSODEG00000016168
ENSODEG00000016169
ENSODEG00000016171
ENSODEG00000016172
ENSODEG00000016177
ENSODEG00000016178
ENSODEG00000016179
ENSODEG00000016180
ENSODEG00000016181
ENSODEG00000016182
ENSODEG00000016184
ENSODEG00000016185
ENSODEG00000016187
ENSODEG00000016188

ENSODEG00000016189
ENSODEG00000016191
ENSODEG00000016192
ENSODEG00000016193
ENSODEG00000016194
ENSODEG00000016195
ENSODEG00000016196
ENSODEG00000016197
ENSODEG00000016201
ENSODEG00000016202
ENSODEG00000016203
ENSODEG00000016204
ENSODEG00000016206
ENSODEG00000016207
ENSODEG00000016208
ENSODEG00000016209
ENSODEG00000016210
ENSODEG00000016211
ENSODEG00000016212
ENSODEG00000016213
ENSODEG00000016214
ENSODEG00000016215
ENSODEG00000016219
ENSODEG00000016220
ENSODEG00000016221
ENSODEG00000016222
ENSODEG00000016223
ENSODEG00000016224
ENSODEG00000016225
ENSODEG00000016226
ENSODEG00000016228
ENSODEG00000016229
ENSODEG00000016230
ENSODEG00000016231
ENSODEG00000016232
ENSODEG00000016233
ENSODEG00000016237
ENSODEG00000016238
ENSODEG00000016239
ENSODEG00000016240
ENSODEG00000016241
ENSODEG00000016243
ENSODEG00000016244
ENSODEG00000016246
ENSODEG00000016247
ENSODEG00000016248
ENSODEG00000016249
ENSODEG00000016251
ENSODEG00000016252
ENSODEG00000016253
ENSODEG00000016254
ENSODEG00000016255
ENSODEG00000016256
ENSODEG00000016257
ENSODEG00000016258
ENSODEG00000016259
ENSODEG00000016260
ENSODEG00000016261
ENSODEG00000016262

ENSODEG00000016263
ENSODEG00000016265
ENSODEG00000016266
ENSODEG00000016267
ENSODEG00000016268
ENSODEG00000016271
ENSODEG00000016273
ENSODEG00000016274
ENSODEG00000016275
ENSODEG00000016276
ENSODEG00000016277
ENSODEG00000016278
ENSODEG00000016279
ENSODEG00000016281
ENSODEG00000016282
ENSODEG00000016283
ENSODEG00000016284
ENSODEG00000016285
ENSODEG00000016286
ENSODEG00000016287
ENSODEG00000016288
ENSODEG00000016290
ENSODEG00000016291
ENSODEG00000016293
ENSODEG00000016295
ENSODEG00000016298
ENSODEG00000016300
ENSODEG00000016301
ENSODEG00000016302
ENSODEG00000016303
ENSODEG00000016304
ENSODEG00000016305
ENSODEG00000016307
ENSODEG00000016308
ENSODEG00000016310
ENSODEG00000016312
ENSODEG00000016313
ENSODEG00000016314
ENSODEG00000016315
ENSODEG00000016316
ENSODEG00000016318
ENSODEG00000016320
ENSODEG00000016321
ENSODEG00000016322
ENSODEG00000016323
ENSODEG00000016324
ENSODEG00000016326
ENSODEG00000016327
ENSODEG00000016329
ENSODEG00000016330
ENSODEG00000016331
ENSODEG00000016332
ENSODEG00000016333
ENSODEG00000016335
ENSODEG00000016336
ENSODEG00000016337
ENSODEG00000016340
ENSODEG00000016341
ENSODEG00000016342

ENSODEG00000016343
ENSODEG00000016344
ENSODEG00000016345
ENSODEG00000016346
ENSODEG00000016347
ENSODEG00000016348
ENSODEG00000016349
ENSODEG00000016352
ENSODEG00000016353
ENSODEG00000016354
ENSODEG00000016357
ENSODEG00000016358
ENSODEG00000016359
ENSODEG00000016360
ENSODEG00000016362
ENSODEG00000016363
ENSODEG00000016364
ENSODEG00000016365
ENSODEG00000016367
ENSODEG00000016369
ENSODEG00000016370
ENSODEG00000016371
ENSODEG00000016372
ENSODEG00000016373
ENSODEG00000016374
ENSODEG00000016375
ENSODEG00000016376
ENSODEG00000016377
ENSODEG00000016380
ENSODEG00000016381
ENSODEG00000016382
ENSODEG00000016383
ENSODEG00000016384
ENSODEG00000016386
ENSODEG00000016387
ENSODEG00000016388
ENSODEG00000016389
ENSODEG00000016392
ENSODEG00000016393
ENSODEG00000016394
ENSODEG00000016397
ENSODEG00000016398
ENSODEG00000016399
ENSODEG00000016400
ENSODEG00000016401
ENSODEG00000016403
ENSODEG00000016404
ENSODEG00000016405
ENSODEG00000016406
ENSODEG00000016407
ENSODEG00000016408
ENSODEG00000016409
ENSODEG00000016410
ENSODEG00000016411
ENSODEG00000016412
ENSODEG00000016413
ENSODEG00000016414
ENSODEG00000016415
ENSODEG00000016417

ENSODEG00000016418
ENSODEG00000016419
ENSODEG00000016422
ENSODEG00000016423
ENSODEG00000016424
ENSODEG00000016425
ENSODEG00000016426
ENSODEG00000016429
ENSODEG00000016433
ENSODEG00000016434
ENSODEG00000016435
ENSODEG00000016436
ENSODEG00000016437
ENSODEG00000016438
ENSODEG00000016439
ENSODEG00000016441
ENSODEG00000016442
ENSODEG00000016443
ENSODEG00000016447
ENSODEG00000016448
ENSODEG00000016449
ENSODEG00000016450
ENSODEG00000016451
ENSODEG00000016452
ENSODEG00000016453
ENSODEG00000016454
ENSODEG00000016455
ENSODEG00000016456
ENSODEG00000016457
ENSODEG00000016458
ENSODEG00000016460
ENSODEG00000016461
ENSODEG00000016463
ENSODEG00000016464
ENSODEG00000016465
ENSODEG00000016466
ENSODEG00000016467
ENSODEG00000016468
ENSODEG00000016469
ENSODEG00000016471
ENSODEG00000016472
ENSODEG00000016473
ENSODEG00000016474
ENSODEG00000016476
ENSODEG00000016477
ENSODEG00000016479
ENSODEG00000016480
ENSODEG00000016481
ENSODEG00000016482
ENSODEG00000016483
ENSODEG00000016485
ENSODEG00000016488
ENSODEG00000016489
ENSODEG00000016490
ENSODEG00000016491
ENSODEG00000016492
ENSODEG00000016494
ENSODEG00000016495
ENSODEG00000016496

ENSODEG00000016497
ENSODEG00000016499
ENSODEG00000016500
ENSODEG00000016503
ENSODEG00000016504
ENSODEG00000016505
ENSODEG00000016506
ENSODEG00000016507
ENSODEG00000016508
ENSODEG00000016510
ENSODEG00000016512
ENSODEG00000016514
ENSODEG00000016516
ENSODEG00000016517
ENSODEG00000016521
ENSODEG00000016524
ENSODEG00000016525
ENSODEG00000016526
ENSODEG00000016528
ENSODEG00000016529
ENSODEG00000016530
ENSODEG00000016531
ENSODEG00000016532
ENSODEG00000016533
ENSODEG00000016534
ENSODEG00000016536
ENSODEG00000016537
ENSODEG00000016538
ENSODEG00000016539
ENSODEG00000016540
ENSODEG00000016541
ENSODEG00000016542
ENSODEG00000016544
ENSODEG00000016545
ENSODEG00000016546
ENSODEG00000016548
ENSODEG00000016550
ENSODEG00000016551
ENSODEG00000016552
ENSODEG00000016554
ENSODEG00000016555
ENSODEG00000016556
ENSODEG00000016557
ENSODEG00000016558
ENSODEG00000016559
ENSODEG00000016560
ENSODEG00000016561
ENSODEG00000016562
ENSODEG00000016565
ENSODEG00000016567
ENSODEG00000016568
ENSODEG00000016570
ENSODEG00000016573
ENSODEG00000016574
ENSODEG00000016576
ENSODEG00000016577
ENSODEG00000016578
ENSODEG00000016579
ENSODEG00000016581

ENSODEG00000016582
ENSODEG00000016583
ENSODEG00000016584
ENSODEG00000016587
ENSODEG00000016588
ENSODEG00000016589
ENSODEG00000016591
ENSODEG00000016592
ENSODEG00000016593
ENSODEG00000016594
ENSODEG00000016597
ENSODEG00000016598
ENSODEG00000016599
ENSODEG00000016601
ENSODEG00000016603
ENSODEG00000016605
ENSODEG00000016606
ENSODEG00000016607
ENSODEG00000016608
ENSODEG00000016610
ENSODEG00000016611
ENSODEG00000016612
ENSODEG00000016615
ENSODEG00000016618
ENSODEG00000016620
ENSODEG00000016621
ENSODEG00000016622
ENSODEG00000016624
ENSODEG00000016625
ENSODEG00000016626
ENSODEG00000016627
ENSODEG00000016628
ENSODEG00000016629
ENSODEG00000016631
ENSODEG00000016632
ENSODEG00000016633
ENSODEG00000016634
ENSODEG00000016635
ENSODEG00000016636
ENSODEG00000016637
ENSODEG00000016638
ENSODEG00000016639
ENSODEG00000016640
ENSODEG00000016641
ENSODEG00000016642
ENSODEG00000016644
ENSODEG00000016645
ENSODEG00000016646
ENSODEG00000016648
ENSODEG00000016649
ENSODEG00000016650
ENSODEG00000016651
ENSODEG00000016652
ENSODEG00000016654
ENSODEG00000016655
ENSODEG00000016657
ENSODEG00000016660
ENSODEG00000016663
ENSODEG00000016666

ENSODEG00000016667
ENSODEG00000016668
ENSODEG00000016669
ENSODEG00000016670
ENSODEG00000016671
ENSODEG00000016675
ENSODEG00000016676
ENSODEG00000016677
ENSODEG00000016679
ENSODEG00000016681
ENSODEG00000016682
ENSODEG00000016683
ENSODEG00000016684
ENSODEG00000016685
ENSODEG00000016686
ENSODEG00000016689
ENSODEG00000016690
ENSODEG00000016691
ENSODEG00000016692
ENSODEG00000016695
ENSODEG00000016696
ENSODEG00000016697
ENSODEG00000016698
ENSODEG00000016699
ENSODEG00000016700
ENSODEG00000016701
ENSODEG00000016702
ENSODEG00000016703
ENSODEG00000016704
ENSODEG00000016707
ENSODEG00000016708
ENSODEG00000016710
ENSODEG00000016711
ENSODEG00000016712
ENSODEG00000016713
ENSODEG00000016715
ENSODEG00000016716
ENSODEG00000016717
ENSODEG00000016718
ENSODEG00000016719
ENSODEG00000016720
ENSODEG00000016724
ENSODEG00000016725
ENSODEG00000016726
ENSODEG00000016727
ENSODEG00000016728
ENSODEG00000016729
ENSODEG00000016730
ENSODEG00000016731
ENSODEG00000016732
ENSODEG00000016734
ENSODEG00000016735
ENSODEG00000016736
ENSODEG00000016737
ENSODEG00000016738
ENSODEG00000016740
ENSODEG00000016741
ENSODEG00000016743
ENSODEG00000016744

ENSODEG00000016748
ENSODEG00000016749
ENSODEG00000016751
ENSODEG00000016753
ENSODEG00000016756
ENSODEG00000016757
ENSODEG00000016758
ENSODEG00000016759
ENSODEG00000016761
ENSODEG00000016762
ENSODEG00000016764
ENSODEG00000016765
ENSODEG00000016766
ENSODEG00000016767
ENSODEG00000016768
ENSODEG00000016769
ENSODEG00000016770
ENSODEG00000016773
ENSODEG00000016774
ENSODEG00000016775
ENSODEG00000016776
ENSODEG00000016778
ENSODEG00000016779
ENSODEG00000016780
ENSODEG00000016781
ENSODEG00000016783
ENSODEG00000016785
ENSODEG00000016786
ENSODEG00000016787
ENSODEG00000016788
ENSODEG00000016790
ENSODEG00000016792
ENSODEG00000016793
ENSODEG00000016794
ENSODEG00000016795
ENSODEG00000016797
ENSODEG00000016798
ENSODEG00000016799
ENSODEG00000016800
ENSODEG00000016803
ENSODEG00000016804
ENSODEG00000016805
ENSODEG00000016806
ENSODEG00000016807
ENSODEG00000016808
ENSODEG00000016809
ENSODEG00000016810
ENSODEG00000016811
ENSODEG00000016812
ENSODEG00000016813
ENSODEG00000016814
ENSODEG00000016815
ENSODEG00000016816
ENSODEG00000016818
ENSODEG00000016819
ENSODEG00000016820
ENSODEG00000016823
ENSODEG00000016824
ENSODEG00000016825

ENSODEG00000016826
ENSODEG00000016827
ENSODEG00000016828
ENSODEG00000016829
ENSODEG00000016830
ENSODEG00000016832
ENSODEG00000016833
ENSODEG00000016838
ENSODEG00000016841
ENSODEG00000016842
ENSODEG00000016844
ENSODEG00000016845
ENSODEG00000016846
ENSODEG00000016847
ENSODEG00000016848
ENSODEG00000016849
ENSODEG00000016850
ENSODEG00000016851
ENSODEG00000016852
ENSODEG00000016853
ENSODEG00000016854
ENSODEG00000016857
ENSODEG00000016858
ENSODEG00000016859
ENSODEG00000016860
ENSODEG00000016862
ENSODEG00000016863
ENSODEG00000016864
ENSODEG00000016867
ENSODEG00000016868
ENSODEG00000016869
ENSODEG00000016870
ENSODEG00000016871
ENSODEG00000016873
ENSODEG00000016874
ENSODEG00000016875
ENSODEG00000016876
ENSODEG00000016877
ENSODEG00000016878
ENSODEG00000016879
ENSODEG00000016880
ENSODEG00000016882
ENSODEG00000016883
ENSODEG00000016884
ENSODEG00000016885
ENSODEG00000016889
ENSODEG00000016890
ENSODEG00000016891
ENSODEG00000016894
ENSODEG00000016895
ENSODEG00000016896
ENSODEG00000016897
ENSODEG00000016899
ENSODEG00000016902
ENSODEG00000016903
ENSODEG00000016907
ENSODEG00000016909
ENSODEG00000016911
ENSODEG00000016912

ENSODEG00000016913
ENSODEG00000016915
ENSODEG00000016916
ENSODEG00000016918
ENSODEG00000016919
ENSODEG00000016920
ENSODEG00000016923
ENSODEG00000016924
ENSODEG00000016926
ENSODEG00000016927
ENSODEG00000016930
ENSODEG00000016931
ENSODEG00000016933
ENSODEG00000016935
ENSODEG00000016937
ENSODEG00000016938
ENSODEG00000016939
ENSODEG00000016940
ENSODEG00000016941
ENSODEG00000016942
ENSODEG00000016944
ENSODEG00000016946
ENSODEG00000016948
ENSODEG00000016949
ENSODEG00000016950
ENSODEG00000016951
ENSODEG00000016952
ENSODEG00000016955
ENSODEG00000016958
ENSODEG00000016959
ENSODEG00000016960
ENSODEG00000016962
ENSODEG00000016963
ENSODEG00000016965
ENSODEG00000016966
ENSODEG00000016967
ENSODEG00000016970
ENSODEG00000016972
ENSODEG00000016973
ENSODEG00000016974
ENSODEG00000016975
ENSODEG00000016977
ENSODEG00000016979
ENSODEG00000016980
ENSODEG00000016981
ENSODEG00000016984
ENSODEG00000016985
ENSODEG00000016986
ENSODEG00000016987
ENSODEG00000016989
ENSODEG00000016990
ENSODEG00000016992
ENSODEG00000016993
ENSODEG00000016994
ENSODEG00000016995
ENSODEG00000016996
ENSODEG00000016998
ENSODEG00000016999
ENSODEG00000017000

ENSODEG00000017001
ENSODEG00000017003
ENSODEG00000017004
ENSODEG00000017006
ENSODEG00000017007
ENSODEG00000017008
ENSODEG00000017010
ENSODEG00000017011
ENSODEG00000017012
ENSODEG00000017014
ENSODEG00000017015
ENSODEG00000017016
ENSODEG00000017017
ENSODEG00000017018
ENSODEG00000017019
ENSODEG00000017020
ENSODEG00000017021
ENSODEG00000017024
ENSODEG00000017025
ENSODEG00000017026
ENSODEG00000017027
ENSODEG00000017028
ENSODEG00000017029
ENSODEG00000017030
ENSODEG00000017031
ENSODEG00000017033
ENSODEG00000017034
ENSODEG00000017035
ENSODEG00000017037
ENSODEG00000017038
ENSODEG00000017039
ENSODEG00000017040
ENSODEG00000017041
ENSODEG00000017042
ENSODEG00000017043
ENSODEG00000017044
ENSODEG00000017045
ENSODEG00000017046
ENSODEG00000017048
ENSODEG00000017049
ENSODEG00000017050
ENSODEG00000017052
ENSODEG00000017053
ENSODEG00000017054
ENSODEG00000017055
ENSODEG00000017058
ENSODEG00000017059
ENSODEG00000017060
ENSODEG00000017061
ENSODEG00000017062
ENSODEG00000017063
ENSODEG00000017066
ENSODEG00000017067
ENSODEG00000017068
ENSODEG00000017069
ENSODEG00000017070
ENSODEG00000017071
ENSODEG00000017072
ENSODEG00000017073

ENSODEG00000017074
ENSODEG00000017075
ENSODEG00000017076
ENSODEG00000017078
ENSODEG00000017079
ENSODEG00000017081
ENSODEG00000017082
ENSODEG00000017083
ENSODEG00000017084
ENSODEG00000017085
ENSODEG00000017086
ENSODEG00000017087
ENSODEG00000017088
ENSODEG00000017089
ENSODEG00000017090
ENSODEG00000017091
ENSODEG00000017093
ENSODEG00000017094
ENSODEG00000017096
ENSODEG00000017097
ENSODEG00000017098
ENSODEG00000017099
ENSODEG00000017100
ENSODEG00000017101
ENSODEG00000017102
ENSODEG00000017103
ENSODEG00000017104
ENSODEG00000017105
ENSODEG00000017106
ENSODEG00000017107
ENSODEG00000017109
ENSODEG00000017111
ENSODEG00000017112
ENSODEG00000017113
ENSODEG00000017114
ENSODEG00000017117
ENSODEG00000017118
ENSODEG00000017120
ENSODEG00000017122
ENSODEG00000017123
ENSODEG00000017124
ENSODEG00000017125
ENSODEG00000017126
ENSODEG00000017127
ENSODEG00000017128
ENSODEG00000017129
ENSODEG00000017130
ENSODEG00000017131
ENSODEG00000017133
ENSODEG00000017134
ENSODEG00000017135
ENSODEG00000017137
ENSODEG00000017138
ENSODEG00000017140
ENSODEG00000017142
ENSODEG00000017144
ENSODEG00000017145
ENSODEG00000017147
ENSODEG00000017148

ENSODEG00000017149
ENSODEG00000017150
ENSODEG00000017151
ENSODEG00000017152
ENSODEG00000017155
ENSODEG00000017156
ENSODEG00000017157
ENSODEG00000017160
ENSODEG00000017161
ENSODEG00000017163
ENSODEG00000017164
ENSODEG00000017165
ENSODEG00000017166
ENSODEG00000017167
ENSODEG00000017168
ENSODEG00000017169
ENSODEG00000017170
ENSODEG00000017171
ENSODEG00000017172
ENSODEG00000017174
ENSODEG00000017175
ENSODEG00000017176
ENSODEG00000017179
ENSODEG00000017180
ENSODEG00000017181
ENSODEG00000017183
ENSODEG00000017184
ENSODEG00000017185
ENSODEG00000017186
ENSODEG00000017187
ENSODEG00000017188
ENSODEG00000017189
ENSODEG00000017190
ENSODEG00000017191
ENSODEG00000017192
ENSODEG00000017193
ENSODEG00000017194
ENSODEG00000017195
ENSODEG00000017198
ENSODEG00000017199
ENSODEG00000017200
ENSODEG00000017202
ENSODEG00000017204
ENSODEG00000017206
ENSODEG00000017207
ENSODEG00000017208
ENSODEG00000017209
ENSODEG00000017210
ENSODEG00000017211
ENSODEG00000017212
ENSODEG00000017213
ENSODEG00000017214
ENSODEG00000017215
ENSODEG00000017216
ENSODEG00000017217
ENSODEG00000017218
ENSODEG00000017219
ENSODEG00000017220
ENSODEG00000017221

ENSODEG00000017222
ENSODEG00000017224
ENSODEG00000017225
ENSODEG00000017226
ENSODEG00000017227
ENSODEG00000017229
ENSODEG00000017230
ENSODEG00000017232
ENSODEG00000017234
ENSODEG00000017236
ENSODEG00000017237
ENSODEG00000017238
ENSODEG00000017239
ENSODEG00000017240
ENSODEG00000017241
ENSODEG00000017242
ENSODEG00000017243
ENSODEG00000017244
ENSODEG00000017245
ENSODEG00000017246
ENSODEG00000017248
ENSODEG00000017249
ENSODEG00000017250
ENSODEG00000017253
ENSODEG00000017254
ENSODEG00000017255
ENSODEG00000017256
ENSODEG00000017257
ENSODEG00000017258
ENSODEG00000017259
ENSODEG00000017261
ENSODEG00000017262
ENSODEG00000017264
ENSODEG00000017266
ENSODEG00000017267
ENSODEG00000017269
ENSODEG00000017270
ENSODEG00000017271
ENSODEG00000017272
ENSODEG00000017273
ENSODEG00000017274
ENSODEG00000017275
ENSODEG00000017278
ENSODEG00000017279
ENSODEG00000017280
ENSODEG00000017281
ENSODEG00000017283
ENSODEG00000017284
ENSODEG00000017287
ENSODEG00000017288
ENSODEG00000017289
ENSODEG00000017290
ENSODEG00000017291
ENSODEG00000017292
ENSODEG00000017294
ENSODEG00000017295
ENSODEG00000017296
ENSODEG00000017298
ENSODEG00000017299

ENSODEG00000017300
ENSODEG00000017301
ENSODEG00000017302
ENSODEG00000017303
ENSODEG00000017304
ENSODEG00000017305
ENSODEG00000017307
ENSODEG00000017308
ENSODEG00000017310
ENSODEG00000017311
ENSODEG00000017313
ENSODEG00000017315
ENSODEG00000017316
ENSODEG00000017318
ENSODEG00000017320
ENSODEG00000017322
ENSODEG00000017323
ENSODEG00000017324
ENSODEG00000017325
ENSODEG00000017326
ENSODEG00000017327
ENSODEG00000017328
ENSODEG00000017329
ENSODEG00000017330
ENSODEG00000017331
ENSODEG00000017332
ENSODEG00000017334
ENSODEG00000017336
ENSODEG00000017337
ENSODEG00000017338
ENSODEG00000017339
ENSODEG00000017340
ENSODEG00000017342
ENSODEG00000017343
ENSODEG00000017345
ENSODEG00000017346
ENSODEG00000017347
ENSODEG00000017348
ENSODEG00000017349
ENSODEG00000017351
ENSODEG00000017353
ENSODEG00000017354
ENSODEG00000017355
ENSODEG00000017356
ENSODEG00000017357
ENSODEG00000017358
ENSODEG00000017359
ENSODEG00000017360
ENSODEG00000017361
ENSODEG00000017362
ENSODEG00000017363
ENSODEG00000017364
ENSODEG00000017367
ENSODEG00000017368
ENSODEG00000017369
ENSODEG00000017370
ENSODEG00000017371
ENSODEG00000017373
ENSODEG00000017374

ENSODEG00000017375
ENSODEG00000017376
ENSODEG00000017377
ENSODEG00000017379
ENSODEG00000017380
ENSODEG00000017381
ENSODEG00000017382
ENSODEG00000017383
ENSODEG00000017384
ENSODEG00000017385
ENSODEG00000017387
ENSODEG00000017391
ENSODEG00000017392
ENSODEG00000017394
ENSODEG00000017395
ENSODEG00000017396
ENSODEG00000017397
ENSODEG00000017398
ENSODEG00000017399
ENSODEG00000017400
ENSODEG00000017401
ENSODEG00000017402
ENSODEG00000017403
ENSODEG00000017404
ENSODEG00000017405
ENSODEG00000017406
ENSODEG00000017407
ENSODEG00000017408
ENSODEG00000017409
ENSODEG00000017410
ENSODEG00000017412
ENSODEG00000017417
ENSODEG00000017418
ENSODEG00000017420
ENSODEG00000017421
ENSODEG00000017422
ENSODEG00000017424
ENSODEG00000017425
ENSODEG00000017426
ENSODEG00000017429
ENSODEG00000017430
ENSODEG00000017431
ENSODEG00000017433
ENSODEG00000017434
ENSODEG00000017435
ENSODEG00000017436
ENSODEG00000017437
ENSODEG00000017438
ENSODEG00000017439
ENSODEG00000017440
ENSODEG00000017444
ENSODEG00000017445
ENSODEG00000017446
ENSODEG00000017447
ENSODEG00000017450
ENSODEG00000017451
ENSODEG00000017452
ENSODEG00000017453
ENSODEG00000017454

ENSODEG00000017456
ENSODEG00000017458
ENSODEG00000017459
ENSODEG00000017460
ENSODEG00000017461
ENSODEG00000017462
ENSODEG00000017463
ENSODEG00000017464
ENSODEG00000017465
ENSODEG00000017466
ENSODEG00000017467
ENSODEG00000017468
ENSODEG00000017469
ENSODEG00000017470
ENSODEG00000017471
ENSODEG00000017472
ENSODEG00000017473
ENSODEG00000017474
ENSODEG00000017475
ENSODEG00000017477
ENSODEG00000017478
ENSODEG00000017480
ENSODEG00000017481
ENSODEG00000017484
ENSODEG00000017486
ENSODEG00000017487
ENSODEG00000017493
ENSODEG00000017494
ENSODEG00000017495
ENSODEG00000017496
ENSODEG00000017498
ENSODEG00000017499
ENSODEG00000017500
ENSODEG00000017501
ENSODEG00000017502
ENSODEG00000017503
ENSODEG00000017505
ENSODEG00000017506
ENSODEG00000017507
ENSODEG00000017508
ENSODEG00000017510
ENSODEG00000017511
ENSODEG00000017512
ENSODEG00000017513
ENSODEG00000017514
ENSODEG00000017516
ENSODEG00000017519
ENSODEG00000017520
ENSODEG00000017521
ENSODEG00000017522
ENSODEG00000017523
ENSODEG00000017524
ENSODEG00000017525
ENSODEG00000017527
ENSODEG00000017528
ENSODEG00000017530
ENSODEG00000017531
ENSODEG00000017532
ENSODEG00000017533

ENSODEG00000017534
ENSODEG00000017536
ENSODEG00000017537
ENSODEG00000017538
ENSODEG00000017539
ENSODEG00000017540
ENSODEG00000017544
ENSODEG00000017545
ENSODEG00000017546
ENSODEG00000017547
ENSODEG00000017548
ENSODEG00000017549
ENSODEG00000017550
ENSODEG00000017551
ENSODEG00000017553
ENSODEG00000017554
ENSODEG00000017555
ENSODEG00000017557
ENSODEG00000017559
ENSODEG00000017560
ENSODEG00000017561
ENSODEG00000017562
ENSODEG00000017563
ENSODEG00000017564
ENSODEG00000017565
ENSODEG00000017567
ENSODEG00000017569
ENSODEG00000017571
ENSODEG00000017572
ENSODEG00000017573
ENSODEG00000017575
ENSODEG00000017576
ENSODEG00000017577
ENSODEG00000017579
ENSODEG00000017580
ENSODEG00000017581
ENSODEG00000017584
ENSODEG00000017585
ENSODEG00000017586
ENSODEG00000017587
ENSODEG00000017588
ENSODEG00000017589
ENSODEG00000017590
ENSODEG00000017592
ENSODEG00000017594
ENSODEG00000017596
ENSODEG00000017598
ENSODEG00000017599
ENSODEG00000017600
ENSODEG00000017601
ENSODEG00000017603
ENSODEG00000017604
ENSODEG00000017605
ENSODEG00000017606
ENSODEG00000017607
ENSODEG00000017610
ENSODEG00000017612
ENSODEG00000017613
ENSODEG00000017615

ENSODEG00000017619
ENSODEG00000017620
ENSODEG00000017621
ENSODEG00000017622
ENSODEG00000017624
ENSODEG00000017626
ENSODEG00000017627
ENSODEG00000017628
ENSODEG00000017629
ENSODEG00000017631
ENSODEG00000017632
ENSODEG00000017635
ENSODEG00000017636
ENSODEG00000017637
ENSODEG00000017640
ENSODEG00000017641
ENSODEG00000017642
ENSODEG00000017643
ENSODEG00000017644
ENSODEG00000017645
ENSODEG00000017646
ENSODEG00000017647
ENSODEG00000017648
ENSODEG00000017650
ENSODEG00000017652
ENSODEG00000017653
ENSODEG00000017655
ENSODEG00000017656
ENSODEG00000017660
ENSODEG00000017661
ENSODEG00000017663
ENSODEG00000017664
ENSODEG00000017665
ENSODEG00000017669
ENSODEG00000017670
ENSODEG00000017674
ENSODEG00000017675
ENSODEG00000017676
ENSODEG00000017677
ENSODEG00000017678
ENSODEG00000017679
ENSODEG00000017681
ENSODEG00000017682
ENSODEG00000017684
ENSODEG00000017685
ENSODEG00000017686
ENSODEG00000017688
ENSODEG00000017689
ENSODEG00000017695
ENSODEG00000017696
ENSODEG00000017697
ENSODEG00000017698
ENSODEG00000017700
ENSODEG00000017701
ENSODEG00000017703
ENSODEG00000017705
ENSODEG00000017706
ENSODEG00000017708
ENSODEG00000017713

ENSODEG00000017714
ENSODEG00000017715
ENSODEG00000017716
ENSODEG00000017717
ENSODEG00000017719
ENSODEG00000017720
ENSODEG00000017721
ENSODEG00000017722
ENSODEG00000017723
ENSODEG00000017724
ENSODEG00000017725
ENSODEG00000017726
ENSODEG00000017728
ENSODEG00000017729
ENSODEG00000017730
ENSODEG00000017731
ENSODEG00000017734
ENSODEG00000017736
ENSODEG00000017739
ENSODEG00000017740
ENSODEG00000017745
ENSODEG00000017748
ENSODEG00000017751
ENSODEG00000017753
ENSODEG00000017754
ENSODEG00000017755
ENSODEG00000017756
ENSODEG00000017757
ENSODEG00000017758
ENSODEG00000017760
ENSODEG00000017762
ENSODEG00000017764
ENSODEG00000017765
ENSODEG00000017766
ENSODEG00000017768
ENSODEG00000017769
ENSODEG00000017770
ENSODEG00000017771
ENSODEG00000017773
ENSODEG00000017774
ENSODEG00000017775
ENSODEG00000017776
ENSODEG00000017777
ENSODEG00000017780
ENSODEG00000017781
ENSODEG00000017784
ENSODEG00000017785
ENSODEG00000017788
ENSODEG00000017789
ENSODEG00000017790
ENSODEG00000017791
ENSODEG00000017792
ENSODEG00000017793
ENSODEG00000017794
ENSODEG00000017795
ENSODEG00000017796
ENSODEG00000017797
ENSODEG00000017798
ENSODEG00000017799

ENSODEG00000017801
ENSODEG00000017803
ENSODEG00000017804
ENSODEG00000017805
ENSODEG00000017806
ENSODEG00000017807
ENSODEG00000017810
ENSODEG00000017811
ENSODEG00000017812
ENSODEG00000017813
ENSODEG00000017814
ENSODEG00000017815
ENSODEG00000017817
ENSODEG00000017818
ENSODEG00000017821
ENSODEG00000017822
ENSODEG00000017824
ENSODEG00000017825
ENSODEG00000017827
ENSODEG00000017828
ENSODEG00000017830
ENSODEG00000017831
ENSODEG00000017832
ENSODEG00000017833
ENSODEG00000017834
ENSODEG00000017836
ENSODEG00000017839
ENSODEG00000017841
ENSODEG00000017842
ENSODEG00000017843
ENSODEG00000017844
ENSODEG00000017845
ENSODEG00000017846
ENSODEG00000017849
ENSODEG00000017850
ENSODEG00000017852
ENSODEG00000017853
ENSODEG00000017856
ENSODEG00000017857
ENSODEG00000017858
ENSODEG00000017860
ENSODEG00000017862
ENSODEG00000017863
ENSODEG00000017864
ENSODEG00000017866
ENSODEG00000017867
ENSODEG00000017868
ENSODEG00000017869
ENSODEG00000017870
ENSODEG00000017871
ENSODEG00000017872
ENSODEG00000017873
ENSODEG00000017874
ENSODEG00000017875
ENSODEG00000017876
ENSODEG00000017878
ENSODEG00000017879
ENSODEG00000017880
ENSODEG00000017881

ENSODEG00000017883
ENSODEG00000017884
ENSODEG00000017886
ENSODEG00000017887
ENSODEG00000017888
ENSODEG00000017889
ENSODEG00000017890
ENSODEG00000017892
ENSODEG00000017893
ENSODEG00000017894
ENSODEG00000017895
ENSODEG00000017896
ENSODEG00000017897
ENSODEG00000017898
ENSODEG00000017900
ENSODEG00000017901
ENSODEG00000017902
ENSODEG00000017904
ENSODEG00000017905
ENSODEG00000017906
ENSODEG00000017907
ENSODEG00000017908
ENSODEG00000017909
ENSODEG00000017910
ENSODEG00000017911
ENSODEG00000017912
ENSODEG00000017914
ENSODEG00000017915
ENSODEG00000017916
ENSODEG00000017917
ENSODEG00000017919
ENSODEG00000017921
ENSODEG00000017922
ENSODEG00000017925
ENSODEG00000017926
ENSODEG00000017927
ENSODEG00000017928
ENSODEG00000017929
ENSODEG00000017930
ENSODEG00000017932
ENSODEG00000017933
ENSODEG00000017934
ENSODEG00000017935
ENSODEG00000017937
ENSODEG00000017938
ENSODEG00000017939
ENSODEG00000017942
ENSODEG00000017943
ENSODEG00000017944
ENSODEG00000017945
ENSODEG00000017947
ENSODEG00000017948
ENSODEG00000017949
ENSODEG00000017950
ENSODEG00000017952
ENSODEG00000017953
ENSODEG00000017954
ENSODEG00000017958
ENSODEG00000017959

ENSODEG00000017961
ENSODEG00000017965
ENSODEG00000017966
ENSODEG00000017967
ENSODEG00000017969
ENSODEG00000017970
ENSODEG00000017971
ENSODEG00000017972
ENSODEG00000017974
ENSODEG00000017975
ENSODEG00000017976
ENSODEG00000017977
ENSODEG00000017978
ENSODEG00000017979
ENSODEG00000017980
ENSODEG00000017981
ENSODEG00000017982
ENSODEG00000017984
ENSODEG00000017986
ENSODEG00000017987
ENSODEG00000017988
ENSODEG00000017990
ENSODEG00000017991
ENSODEG00000017993
ENSODEG00000017995
ENSODEG00000017996
ENSODEG00000017998
ENSODEG00000017999
ENSODEG00000018000
ENSODEG00000018001
ENSODEG00000018002
ENSODEG00000018004
ENSODEG00000018005
ENSODEG00000018007
ENSODEG00000018009
ENSODEG00000018014
ENSODEG00000018015
ENSODEG00000018018
ENSODEG00000018019
ENSODEG00000018021
ENSODEG00000018023
ENSODEG00000018026
ENSODEG00000018027
ENSODEG00000018029
ENSODEG00000018030
ENSODEG00000018031
ENSODEG00000018032
ENSODEG00000018033
ENSODEG00000018034
ENSODEG00000018035
ENSODEG00000018037
ENSODEG00000018038
ENSODEG00000018039
ENSODEG00000018040
ENSODEG00000018041
ENSODEG00000018042
ENSODEG00000018043
ENSODEG00000018044
ENSODEG00000018045

ENSODEG00000018046
ENSODEG00000018047
ENSODEG00000018048
ENSODEG00000018049
ENSODEG00000018050
ENSODEG00000018051
ENSODEG00000018054
ENSODEG00000018056
ENSODEG00000018057
ENSODEG00000018058
ENSODEG00000018059
ENSODEG00000018061
ENSODEG00000018062
ENSODEG00000018063
ENSODEG00000018065
ENSODEG00000018066
ENSODEG00000018068
ENSODEG00000018069
ENSODEG00000018070
ENSODEG00000018071
ENSODEG00000018072
ENSODEG00000018073
ENSODEG00000018075
ENSODEG00000018076
ENSODEG00000018077
ENSODEG00000018078
ENSODEG00000018079
ENSODEG00000018080
ENSODEG00000018081
ENSODEG00000018082
ENSODEG00000018085
ENSODEG00000018086
ENSODEG00000018087
ENSODEG00000018088
ENSODEG00000018090
ENSODEG00000018091
ENSODEG00000018092
ENSODEG00000018095
ENSODEG00000018096
ENSODEG00000018098
ENSODEG00000018099
ENSODEG00000018102
ENSODEG00000018103
ENSODEG00000018104
ENSODEG00000018105
ENSODEG00000018106
ENSODEG00000018107
ENSODEG00000018108
ENSODEG00000018111
ENSODEG00000018112
ENSODEG00000018113
ENSODEG00000018114
ENSODEG00000018115
ENSODEG00000018116
ENSODEG00000018117
ENSODEG00000018118
ENSODEG00000018119
ENSODEG00000018120
ENSODEG00000018121

ENSODEG00000018122
ENSODEG00000018123
ENSODEG00000018124
ENSODEG00000018125
ENSODEG00000018126
ENSODEG00000018128
ENSODEG00000018129
ENSODEG00000018131
ENSODEG00000018132
ENSODEG00000018133
ENSODEG00000018134
ENSODEG00000018135
ENSODEG00000018136
ENSODEG00000018137
ENSODEG00000018141
ENSODEG00000018142
ENSODEG00000018143
ENSODEG00000018144
ENSODEG00000018145
ENSODEG00000018149
ENSODEG00000018150
ENSODEG00000018152
ENSODEG00000018153
ENSODEG00000018154
ENSODEG00000018155
ENSODEG00000018157
ENSODEG00000018158
ENSODEG00000018160
ENSODEG00000018161
ENSODEG00000018162
ENSODEG00000018165
ENSODEG00000018166
ENSODEG00000018167
ENSODEG00000018168
ENSODEG00000018170
ENSODEG00000018171
ENSODEG00000018175
ENSODEG00000018176
ENSODEG00000018179
ENSODEG00000018180
ENSODEG00000018181
ENSODEG00000018182
ENSODEG00000018186
ENSODEG00000018189
ENSODEG00000018190
ENSODEG00000018191
ENSODEG00000018193
ENSODEG00000018194
ENSODEG00000018195
ENSODEG00000018196
ENSODEG00000018198
ENSODEG00000018199
ENSODEG00000018201
ENSODEG00000018203
ENSODEG00000018204
ENSODEG00000018205
ENSODEG00000018206
ENSODEG00000018207
ENSODEG00000018208

ENSODEG00000018209
ENSODEG00000018210
ENSODEG00000018212
ENSODEG00000018213
ENSODEG00000018215
ENSODEG00000018217
ENSODEG00000018218
ENSODEG00000018219
ENSODEG00000018220
ENSODEG00000018225
ENSODEG00000018226
ENSODEG00000018227
ENSODEG00000018228
ENSODEG00000018230
ENSODEG00000018232
ENSODEG00000018233
ENSODEG00000018234
ENSODEG00000018235
ENSODEG00000018236
ENSODEG00000018237
ENSODEG00000018238
ENSODEG00000018239
ENSODEG00000018240
ENSODEG00000018242
ENSODEG00000018243
ENSODEG00000018244
ENSODEG00000018246
ENSODEG00000018247
ENSODEG00000018249
ENSODEG00000018251
ENSODEG00000018252
ENSODEG00000018253
ENSODEG00000018254
ENSODEG00000018255
ENSODEG00000018256
ENSODEG00000018257
ENSODEG00000018258
ENSODEG00000018259
ENSODEG00000018260
ENSODEG00000018261
ENSODEG00000018264
ENSODEG00000018265
ENSODEG00000018266
ENSODEG00000018267
ENSODEG00000018268
ENSODEG00000018270
ENSODEG00000018271
ENSODEG00000018272
ENSODEG00000018273
ENSODEG00000018274
ENSODEG00000018275
ENSODEG00000018276
ENSODEG00000018277
ENSODEG00000018278
ENSODEG00000018279
ENSODEG00000018280
ENSODEG00000018282
ENSODEG00000018283
ENSODEG00000018286

ENSODEG00000018289
ENSODEG00000018290
ENSODEG00000018291
ENSODEG00000018292
ENSODEG00000018293
ENSODEG00000018294
ENSODEG00000018295
ENSODEG00000018296
ENSODEG00000018298
ENSODEG00000018299
ENSODEG00000018300
ENSODEG00000018301
ENSODEG00000018302
ENSODEG00000018305
ENSODEG00000018307
ENSODEG00000018309
ENSODEG00000018310
ENSODEG00000018311
ENSODEG00000018313
ENSODEG00000018314
ENSODEG00000018315
ENSODEG00000018316
ENSODEG00000018317
ENSODEG00000018318
ENSODEG00000018319
ENSODEG00000018320
ENSODEG00000018323
ENSODEG00000018325
ENSODEG00000018326
ENSODEG00000018327
ENSODEG00000018329
ENSODEG00000018330
ENSODEG00000018333
ENSODEG00000018334
ENSODEG00000018335
ENSODEG00000018336
ENSODEG00000018337
ENSODEG00000018338
ENSODEG00000018339
ENSODEG00000018340
ENSODEG00000018341
ENSODEG00000018343
ENSODEG00000018344
ENSODEG00000018345
ENSODEG00000018347
ENSODEG00000018348
ENSODEG00000018349
ENSODEG00000018350
ENSODEG00000018351
ENSODEG00000018353
ENSODEG00000018354
ENSODEG00000018355
ENSODEG00000018356
ENSODEG00000018357
ENSODEG00000018360
ENSODEG00000018361
ENSODEG00000018362
ENSODEG00000018363
ENSODEG00000018365

ENSODEG00000018366
ENSODEG00000018367
ENSODEG00000018368
ENSODEG00000018369
ENSODEG00000018370
ENSODEG00000018372
ENSODEG00000018374
ENSODEG00000018376
ENSODEG00000018377
ENSODEG00000018378
ENSODEG00000018379
ENSODEG00000018380
ENSODEG00000018382
ENSODEG00000018384
ENSODEG00000018385
ENSODEG00000018387
ENSODEG00000018389
ENSODEG00000018390
ENSODEG00000018391
ENSODEG00000018392
ENSODEG00000018393
ENSODEG00000018394
ENSODEG00000018395
ENSODEG00000018396
ENSODEG00000018397
ENSODEG00000018399
ENSODEG00000018400
ENSODEG00000018401
ENSODEG00000018402
ENSODEG00000018403
ENSODEG00000018404
ENSODEG00000018405
ENSODEG00000018407
ENSODEG00000018408
ENSODEG00000018409
ENSODEG00000018410
ENSODEG00000018411
ENSODEG00000018413
ENSODEG00000018414
ENSODEG00000018415
ENSODEG00000018416
ENSODEG00000018417
ENSODEG00000018418
ENSODEG00000018419
ENSODEG00000018420
ENSODEG00000018421
ENSODEG00000018423
ENSODEG00000018424
ENSODEG00000018425
ENSODEG00000018427
ENSODEG00000018429
ENSODEG00000018430
ENSODEG00000018431
ENSODEG00000018432
ENSODEG00000018433
ENSODEG00000018434
ENSODEG00000018436
ENSODEG00000018437
ENSODEG00000018438

ENSODEG00000018440
ENSODEG00000018441
ENSODEG00000018442
ENSODEG00000018443
ENSODEG00000018444
ENSODEG00000018445
ENSODEG00000018446
ENSODEG00000018447
ENSODEG00000018449
ENSODEG00000018450
ENSODEG00000018452
ENSODEG00000018453
ENSODEG00000018454
ENSODEG00000018455
ENSODEG00000018456
ENSODEG00000018457
ENSODEG00000018459
ENSODEG00000018461
ENSODEG00000018462
ENSODEG00000018463
ENSODEG00000018467
ENSODEG00000018468
ENSODEG00000018469
ENSODEG00000018470
ENSODEG00000018471
ENSODEG00000018473
ENSODEG00000018474
ENSODEG00000018475
ENSODEG00000018476
ENSODEG00000018477
ENSODEG00000018478
ENSODEG00000018480
ENSODEG00000018481
ENSODEG00000018482
ENSODEG00000018483
ENSODEG00000018484
ENSODEG00000018485
ENSODEG00000018486
ENSODEG00000018487
ENSODEG00000018488
ENSODEG00000018489
ENSODEG00000018491
ENSODEG00000018492
ENSODEG00000018493
ENSODEG00000018495
ENSODEG00000018496
ENSODEG00000018497
ENSODEG00000018498
ENSODEG00000018499
ENSODEG00000018500
ENSODEG00000018503
ENSODEG00000018505
ENSODEG00000018506
ENSODEG00000018507
ENSODEG00000018508
ENSODEG00000018509
ENSODEG00000018510
ENSODEG00000018511
ENSODEG00000018514

ENSODEG00000018515
ENSODEG00000018516
ENSODEG00000018517
ENSODEG00000018518
ENSODEG00000018519
ENSODEG00000018520
ENSODEG00000018521
ENSODEG00000018522
ENSODEG00000018525
ENSODEG00000018526
ENSODEG00000018527
ENSODEG00000018528
ENSODEG00000018529
ENSODEG00000018530
ENSODEG00000018531
ENSODEG00000018533
ENSODEG00000018534
ENSODEG00000018536
ENSODEG00000018537
ENSODEG00000018538
ENSODEG00000018539
ENSODEG00000018541
ENSODEG00000018543
ENSODEG00000018545
ENSODEG00000018546
ENSODEG00000018547
ENSODEG00000018548
ENSODEG00000018549
ENSODEG00000018550
ENSODEG00000018551
ENSODEG00000018552
ENSODEG00000018556
ENSODEG00000018557
ENSODEG00000018560
ENSODEG00000018561
ENSODEG00000018563
ENSODEG00000018568
ENSODEG00000018569
ENSODEG00000018570
ENSODEG00000018571
ENSODEG00000018572
ENSODEG00000018574
ENSODEG00000018575
ENSODEG00000018576
ENSODEG00000018579
ENSODEG00000018580
ENSODEG00000018581
ENSODEG00000018583
ENSODEG00000018584
ENSODEG00000018585
ENSODEG00000018586
ENSODEG00000018588
ENSODEG00000018591
ENSODEG00000018593
ENSODEG00000018595
ENSODEG00000018599
ENSODEG00000018600
ENSODEG00000018603
ENSODEG00000018604

ENSODEG00000018605
ENSODEG00000018607
ENSODEG00000018608
ENSODEG00000018609
ENSODEG00000018610
ENSODEG00000018611
ENSODEG00000018612
ENSODEG00000018613
ENSODEG00000018614
ENSODEG00000018615
ENSODEG00000018616
ENSODEG00000018617
ENSODEG00000018618
ENSODEG00000018621
ENSODEG00000018622
ENSODEG00000018623
ENSODEG00000018624
ENSODEG00000018625
ENSODEG00000018627
ENSODEG00000018628
ENSODEG00000018629
ENSODEG00000018631
ENSODEG00000018632
ENSODEG00000018633
ENSODEG00000018634
ENSODEG00000018637
ENSODEG00000018638
ENSODEG00000018639
ENSODEG00000018640
ENSODEG00000018642
ENSODEG00000018644
ENSODEG00000018645
ENSODEG00000018646
ENSODEG00000018647
ENSODEG00000018648
ENSODEG00000018649
ENSODEG00000018653
ENSODEG00000018654
ENSODEG00000018655
ENSODEG00000018656
ENSODEG00000018657
ENSODEG00000018659
ENSODEG00000018660
ENSODEG00000018662
ENSODEG00000018663
ENSODEG00000018664
ENSODEG00000018665
ENSODEG00000018667
ENSODEG00000018668
ENSODEG00000018669
ENSODEG00000018670
ENSODEG00000018671
ENSODEG00000018672
ENSODEG00000018675
ENSODEG00000018676
ENSODEG00000018677
ENSODEG00000018678
ENSODEG00000018679
ENSODEG00000018680

ENSODEG00000018681
ENSODEG00000018683
ENSODEG00000018684
ENSODEG00000018686
ENSODEG00000018687
ENSODEG00000018688
ENSODEG00000018689
ENSODEG00000018690
ENSODEG00000018691
ENSODEG00000018692
ENSODEG00000018693
ENSODEG00000018695
ENSODEG00000018696
ENSODEG00000018697
ENSODEG00000018700
ENSODEG00000018701
ENSODEG00000018702
ENSODEG00000018704
ENSODEG00000018706
ENSODEG00000018707
ENSODEG00000018708
ENSODEG00000018709
ENSODEG00000018710
ENSODEG00000018712
ENSODEG00000018715
ENSODEG00000018717
ENSODEG00000018718
ENSODEG00000018720
ENSODEG00000018723
ENSODEG00000018724
ENSODEG00000018726
ENSODEG00000018727
ENSODEG00000018729
ENSODEG00000018730
ENSODEG00000018731
ENSODEG00000018732
ENSODEG00000018733
ENSODEG00000018735
ENSODEG00000018736
ENSODEG00000018737
ENSODEG00000018738
ENSODEG00000018739
ENSODEG00000018740
ENSODEG00000018742
ENSODEG00000018743
ENSODEG00000018744
ENSODEG00000018747
ENSODEG00000018748
ENSODEG00000018749
ENSODEG00000018750
ENSODEG00000018751
ENSODEG00000018753
ENSODEG00000018754
ENSODEG00000018756
ENSODEG00000018758
ENSODEG00000018759
ENSODEG00000018760
ENSODEG00000018761
ENSODEG00000018763

ENSODEG00000018764
ENSODEG00000018765
ENSODEG00000018766
ENSODEG00000018768
ENSODEG00000018769
ENSODEG00000018770
ENSODEG00000018773
ENSODEG00000018774
ENSODEG00000018776
ENSODEG00000018777
ENSODEG00000018778
ENSODEG00000018779
ENSODEG00000018781
ENSODEG00000018783
ENSODEG00000018784
ENSODEG00000018786
ENSODEG00000018788
ENSODEG00000018790
ENSODEG00000018791
ENSODEG00000018793
ENSODEG00000018794
ENSODEG00000018795
ENSODEG00000018796
ENSODEG00000018797
ENSODEG00000018798
ENSODEG00000018799
ENSODEG00000018800
ENSODEG00000018801
ENSODEG00000018803
ENSODEG00000018804
ENSODEG00000018806
ENSODEG00000018808
ENSODEG00000018810
ENSODEG00000018811
ENSODEG00000018812
ENSODEG00000018813
ENSODEG00000018814
ENSODEG00000018815
ENSODEG00000018816
ENSODEG00000018818
ENSODEG00000018819
ENSODEG00000018821
ENSODEG00000018822
ENSODEG00000018823
ENSODEG00000018825
ENSODEG00000018826
ENSODEG00000018827
ENSODEG00000018828
ENSODEG00000018829
ENSODEG00000018831
ENSODEG00000018832
ENSODEG00000018834
ENSODEG00000018835
ENSODEG00000018836
ENSODEG00000018837
ENSODEG00000018838
ENSODEG00000018839
ENSODEG00000018840
ENSODEG00000018841

ENSODEG00000018842
ENSODEG00000018843
ENSODEG00000018844
ENSODEG00000018845
ENSODEG00000018846
ENSODEG00000018847
ENSODEG00000018851
ENSODEG00000018853
ENSODEG00000018854
ENSODEG00000018855
ENSODEG00000018856
ENSODEG00000018857
ENSODEG00000018858
ENSODEG00000018859
ENSODEG00000018860
ENSODEG00000018861
ENSODEG00000018864
ENSODEG00000018865
ENSODEG00000018866
ENSODEG00000018867
ENSODEG00000018868
ENSODEG00000018870
ENSODEG00000018871
ENSODEG00000018872
ENSODEG00000018873
ENSODEG00000018874
ENSODEG00000018876
ENSODEG00000018877
ENSODEG00000018878
ENSODEG00000018879
ENSODEG00000018880
ENSODEG00000018881
ENSODEG00000018882
ENSODEG00000018883
ENSODEG00000018884
ENSODEG00000018885
ENSODEG00000018886
ENSODEG00000018888
ENSODEG00000018889
ENSODEG00000018890
ENSODEG00000018891
ENSODEG00000018892
ENSODEG00000018893
ENSODEG00000018894
ENSODEG00000018896
ENSODEG00000018897
ENSODEG00000018898
ENSODEG00000018900
ENSODEG00000018901
ENSODEG00000018902
ENSODEG00000018905
ENSODEG00000018908
ENSODEG00000018909
ENSODEG00000018910
ENSODEG00000018911
ENSODEG00000018912
ENSODEG00000018913
ENSODEG00000018914
ENSODEG00000018915

ENSODEG00000018916
ENSODEG00000018917
ENSODEG00000018919
ENSODEG00000018920
ENSODEG00000018922
ENSODEG00000018923
ENSODEG00000018924
ENSODEG00000018925
ENSODEG00000018927
ENSODEG00000018928
ENSODEG00000018929
ENSODEG00000018931
ENSODEG00000018932
ENSODEG00000018933
ENSODEG00000018934
ENSODEG00000018935
ENSODEG00000018936
ENSODEG00000018937
ENSODEG00000018938
ENSODEG00000018939
ENSODEG00000018940
ENSODEG00000018941
ENSODEG00000018943
ENSODEG00000018944
ENSODEG00000018945
ENSODEG00000018947
ENSODEG00000018949
ENSODEG00000018952
ENSODEG00000018953
ENSODEG00000018954
ENSODEG00000018956
ENSODEG00000018957
ENSODEG00000018958
ENSODEG00000018960
ENSODEG00000018961
ENSODEG00000018963
ENSODEG00000018964
ENSODEG00000018965
ENSODEG00000018967
ENSODEG00000018969
ENSODEG00000018970
ENSODEG00000018971
ENSODEG00000018972
ENSODEG00000018973
ENSODEG00000018975
ENSODEG00000018977
ENSODEG00000018978
ENSODEG00000018979
ENSODEG00000018980
ENSODEG00000018981
ENSODEG00000018982
ENSODEG00000018983
ENSODEG00000018987
ENSODEG00000018988
ENSODEG00000018990
ENSODEG00000018991
ENSODEG00000018993
ENSODEG00000018995
ENSODEG00000018996

ENSODEG00000018997
ENSODEG00000018998
ENSODEG00000018999
ENSODEG00000019000
ENSODEG00000019004
ENSODEG00000019005
ENSODEG00000019007
ENSODEG00000019009
ENSODEG00000019010
ENSODEG00000019011
ENSODEG00000019012
ENSODEG00000019014
ENSODEG00000019016
ENSODEG00000019017
ENSODEG00000019018
ENSODEG00000019019
ENSODEG00000019020
ENSODEG00000019021
ENSODEG00000019022
ENSODEG00000019025
ENSODEG00000019026
ENSODEG00000019028
ENSODEG00000019030
ENSODEG00000019032
ENSODEG00000019033
ENSODEG00000019034
ENSODEG00000019035
ENSODEG00000019036
ENSODEG00000019037
ENSODEG00000019038
ENSODEG00000019040
ENSODEG00000019041
ENSODEG00000019042
ENSODEG00000019043
ENSODEG00000019044
ENSODEG00000019046
ENSODEG00000019048
ENSODEG00000019049
ENSODEG00000019050
ENSODEG00000019051
ENSODEG00000019052
ENSODEG00000019053
ENSODEG00000019057
ENSODEG00000019058
ENSODEG00000019059
ENSODEG00000019060
ENSODEG00000019062
ENSODEG00000019064
ENSODEG00000019065
ENSODEG00000019068
ENSODEG00000019069
ENSODEG00000019070
ENSODEG00000019071
ENSODEG00000019072
ENSODEG00000019074
ENSODEG00000019075
ENSODEG00000019076
ENSODEG00000019078
ENSODEG00000019079

ENSODEG00000019081
ENSODEG00000019084
ENSODEG00000019085
ENSODEG00000019086
ENSODEG00000019090
ENSODEG00000019092
ENSODEG00000019093
ENSODEG00000019094
ENSODEG00000019095
ENSODEG00000019096
ENSODEG00000019097
ENSODEG00000019098
ENSODEG00000019100
ENSODEG00000019101
ENSODEG00000019102
ENSODEG00000019103
ENSODEG00000019104
ENSODEG00000019105
ENSODEG00000019106
ENSODEG00000019107
ENSODEG00000019108
ENSODEG00000019110
ENSODEG00000019111
ENSODEG00000019112
ENSODEG00000019113
ENSODEG00000019114
ENSODEG00000019115
ENSODEG00000019116
ENSODEG00000019117
ENSODEG00000019119
ENSODEG00000019121
ENSODEG00000019122
ENSODEG00000019124
ENSODEG00000019127
ENSODEG00000019128
ENSODEG00000019129
ENSODEG00000019130
ENSODEG00000019131
ENSODEG00000019134
ENSODEG00000019135
ENSODEG00000019136
ENSODEG00000019137
ENSODEG00000019138
ENSODEG00000019140
ENSODEG00000019141
ENSODEG00000019143
ENSODEG00000019144
ENSODEG00000019145
ENSODEG00000019146
ENSODEG00000019147
ENSODEG00000019149
ENSODEG00000019150
ENSODEG00000019151
ENSODEG00000019152
ENSODEG00000019153
ENSODEG00000019154
ENSODEG00000019155
ENSODEG00000019156
ENSODEG00000019157

ENSODEG00000019158
ENSODEG00000019159
ENSODEG00000019162
ENSODEG00000019163
ENSODEG00000019165
ENSODEG00000019169
ENSODEG00000019170
ENSODEG00000019171
ENSODEG00000019172
ENSODEG00000019174
ENSODEG00000019176
ENSODEG00000019177
ENSODEG00000019180
ENSODEG00000019181
ENSODEG00000019183
ENSODEG00000019186
ENSODEG00000019187
ENSODEG00000019189
ENSODEG00000019190
ENSODEG00000019191
ENSODEG00000019192
ENSODEG00000019193
ENSODEG00000019195
ENSODEG00000019196
ENSODEG00000019197
ENSODEG00000019198
ENSODEG00000019199
ENSODEG00000019200
ENSODEG00000019201
ENSODEG00000019202
ENSODEG00000019204
ENSODEG00000019205
ENSODEG00000019206
ENSODEG00000019207
ENSODEG00000019211
ENSODEG00000019214
ENSODEG00000019215
ENSODEG00000019216
ENSODEG00000019217
ENSODEG00000019218
ENSODEG00000019219
ENSODEG00000019220
ENSODEG00000019221
ENSODEG00000019222
ENSODEG00000019223
ENSODEG00000019225
ENSODEG00000019226
ENSODEG00000019227
ENSODEG00000019230
ENSODEG00000019231
ENSODEG00000019232
ENSODEG00000019233
ENSODEG00000019234
ENSODEG00000019235
ENSODEG00000019237
ENSODEG00000019238
ENSODEG00000019239
ENSODEG00000019240
ENSODEG00000019241

ENSODEG00000019244
ENSODEG00000019246
ENSODEG00000019247
ENSODEG00000019248
ENSODEG00000019249
ENSODEG00000019251
ENSODEG00000019253
ENSODEG00000019254
ENSODEG00000019255
ENSODEG00000019257
ENSODEG00000019258
ENSODEG00000019259
ENSODEG00000019261
ENSODEG00000019262
ENSODEG00000019263
ENSODEG00000019264
ENSODEG00000019265
ENSODEG00000019266
ENSODEG00000019267
ENSODEG00000019268
ENSODEG00000019269
ENSODEG00000019270
ENSODEG00000019271
ENSODEG00000019272
ENSODEG00000019273
ENSODEG00000019275
ENSODEG00000019276
ENSODEG00000019277
ENSODEG00000019279
ENSODEG00000019281
ENSODEG00000019282
ENSODEG00000019283
ENSODEG00000019286
ENSODEG00000019287
ENSODEG00000019288
ENSODEG00000019289
ENSODEG00000019290
ENSODEG00000019291
ENSODEG00000019292
ENSODEG00000019295
ENSODEG00000019296
ENSODEG00000019297
ENSODEG00000019298
ENSODEG00000019300
ENSODEG00000019301
ENSODEG00000019303
ENSODEG00000019304
ENSODEG00000019306
ENSODEG00000019307
ENSODEG00000019308
ENSODEG00000019309
ENSODEG00000019310
ENSODEG00000019311
ENSODEG00000019312
ENSODEG00000019313
ENSODEG00000019314
ENSODEG00000019317
ENSODEG00000019318
ENSODEG00000019319

ENSODEG00000019324
ENSODEG00000019326
ENSODEG00000019330
ENSODEG00000019332
ENSODEG00000019333
ENSODEG00000019334
ENSODEG00000019335
ENSODEG00000019336
ENSODEG00000019341
ENSODEG00000019344
ENSODEG00000019345
ENSODEG00000019346
ENSODEG00000019347
ENSODEG00000019348
ENSODEG00000019351
ENSODEG00000019353
ENSODEG00000019354
ENSODEG00000019355
ENSODEG00000019356
ENSODEG00000019358
ENSODEG00000019359
ENSODEG00000019360
ENSODEG00000019363
ENSODEG00000019364
ENSODEG00000019365
ENSODEG00000019366
ENSODEG00000019367
ENSODEG00000019368
ENSODEG00000019372
ENSODEG00000019373
ENSODEG00000019375
ENSODEG00000019376
ENSODEG00000019377
ENSODEG00000019378
ENSODEG00000019379
ENSODEG00000019380
ENSODEG00000019381
ENSODEG00000019382
ENSODEG00000019383
ENSODEG00000019384
ENSODEG00000019385
ENSODEG00000019386
ENSODEG00000019387
ENSODEG00000019388
ENSODEG00000019389
ENSODEG00000019390
ENSODEG00000019391
ENSODEG00000019394
ENSODEG00000019395
ENSODEG00000019397
ENSODEG00000019398
ENSODEG00000019401
ENSODEG00000019403
ENSODEG00000019404
ENSODEG00000019405
ENSODEG00000019406
ENSODEG00000019407
ENSODEG00000019411
ENSODEG00000019412

ENSODEG00000019413
ENSODEG00000019414
ENSODEG00000019415
ENSODEG00000019416
ENSODEG00000019417
ENSODEG00000019419
ENSODEG00000019420
ENSODEG00000019421
ENSODEG00000019422
ENSODEG00000019423
ENSODEG00000019424
ENSODEG00000019425
ENSODEG00000019426
ENSODEG00000019427
ENSODEG00000019428
ENSODEG00000019429
ENSODEG00000019430
ENSODEG00000019431
ENSODEG00000019432
ENSODEG00000019435
ENSODEG00000019436
ENSODEG00000019437
ENSODEG00000019438
ENSODEG00000019439
ENSODEG00000019440
ENSODEG00000019442
ENSODEG00000019443
ENSODEG00000019444
ENSODEG00000019445
ENSODEG00000019446
ENSODEG00000019447
ENSODEG00000019450
ENSODEG00000019451
ENSODEG00000019452
ENSODEG00000019454
ENSODEG00000019455
ENSODEG00000019456
ENSODEG00000019457
ENSODEG00000019458
ENSODEG00000019459
ENSODEG00000019462
ENSODEG00000019464
ENSODEG00000019465
ENSODEG00000019467
ENSODEG00000019468
ENSODEG00000019470
ENSODEG00000019472
ENSODEG00000019473
ENSODEG00000019474
ENSODEG00000019476
ENSODEG00000019477
ENSODEG00000019479
ENSODEG00000019480
ENSODEG00000019481
ENSODEG00000019482
ENSODEG00000019483
ENSODEG00000019484
ENSODEG00000019485
ENSODEG00000019487

ENSODEG00000019488
ENSODEG00000019489
ENSODEG00000019491
ENSODEG00000019492
ENSODEG00000019494
ENSODEG00000019496
ENSODEG00000019498
ENSODEG00000019500
ENSODEG00000019502
ENSODEG00000019503
ENSODEG00000019505
ENSODEG00000019506
ENSODEG00000019508
ENSODEG00000019510
ENSODEG00000019511
ENSODEG00000019512
ENSODEG00000019513
ENSODEG00000019517
ENSODEG00000019519
ENSODEG00000019521
ENSODEG00000019522
ENSODEG00000019523
ENSODEG00000019524
ENSODEG00000019525
ENSODEG00000019526
ENSODEG00000019527
ENSODEG00000019528
ENSODEG00000019529
ENSODEG00000019530
ENSODEG00000019531
ENSODEG00000019532
ENSODEG00000019533
ENSODEG00000019535
ENSODEG00000019537
ENSODEG00000019538
ENSODEG00000019539
ENSODEG00000019541
ENSODEG00000019542
ENSODEG00000019543
ENSODEG00000019544
ENSODEG00000019546
ENSODEG00000019547
ENSODEG00000019548
ENSODEG00000019549
ENSODEG00000019551
ENSODEG00000019554
ENSODEG00000019555
ENSODEG00000019558
ENSODEG00000019560
ENSODEG00000019562
ENSODEG00000019564
ENSODEG00000019565
ENSODEG00000019567
ENSODEG00000019569
ENSODEG00000019572
ENSODEG00000019574
ENSODEG00000019575
ENSODEG00000019577
ENSODEG00000019579

ENSODEG00000019580
ENSODEG00000019581
ENSODEG00000019582
ENSODEG00000019583
ENSODEG00000019585
ENSODEG00000019586
ENSODEG00000019587
ENSODEG00000019589
ENSODEG00000019590
ENSODEG00000019592
ENSODEG00000019594
ENSODEG00000019595
ENSODEG00000019596
ENSODEG00000019597
ENSODEG00000019598
ENSODEG00000019599
ENSODEG00000019600
ENSODEG00000019601
ENSODEG00000019603
ENSODEG00000019604
ENSODEG00000019605
ENSODEG00000019606
ENSODEG00000019608
ENSODEG00000019609
ENSODEG00000019610
ENSODEG00000019611
ENSODEG00000019612
ENSODEG00000019613
ENSODEG00000019615
ENSODEG00000019616
ENSODEG00000019617
ENSODEG00000019619
ENSODEG00000019621
ENSODEG00000019624
ENSODEG00000019625
ENSODEG00000019626
ENSODEG00000019627
ENSODEG00000019628
ENSODEG00000019630
ENSODEG00000019631
ENSODEG00000019633
ENSODEG00000019634
ENSODEG00000019635
ENSODEG00000019636
ENSODEG00000019637
ENSODEG00000019638
ENSODEG00000019639
ENSODEG00000019640
ENSODEG00000019641
ENSODEG00000019644
ENSODEG00000019645
ENSODEG00000019646
ENSODEG00000019647
ENSODEG00000019648
ENSODEG00000019650
ENSODEG00000019652
ENSODEG00000019653
ENSODEG00000019654
ENSODEG00000019655

ENSODEG00000019656
ENSODEG00000019657
ENSODEG00000019659
ENSODEG00000019660
ENSODEG00000019662
ENSODEG00000019664
ENSODEG00000019669
ENSODEG00000019670
ENSODEG00000019671
ENSODEG00000019672
ENSODEG00000019673
ENSODEG00000019675
ENSODEG00000019677
ENSODEG00000019678
ENSODEG00000019679
ENSODEG00000019680
ENSODEG00000019681
ENSODEG00000019683
ENSODEG00000019684
ENSODEG00000019685
ENSODEG00000019686
ENSODEG00000019687
ENSODEG00000019688
ENSODEG00000019689
ENSODEG00000019690
ENSODEG00000019691
ENSODEG00000019692
ENSODEG00000019693
ENSODEG00000019694
ENSODEG00000019695
ENSODEG00000019696
ENSODEG00000019698
ENSODEG00000019699
ENSODEG00000019701
ENSODEG00000019702
ENSODEG00000019703
ENSODEG00000019704
ENSODEG00000019705
ENSODEG00000019706
ENSODEG00000019708
ENSODEG00000019710
ENSODEG00000019711
ENSODEG00000019714
ENSODEG00000019715
ENSODEG00000019716
ENSODEG00000019717
ENSODEG00000019718
ENSODEG00000019719
ENSODEG00000019721
ENSODEG00000019722
ENSODEG00000019723
ENSODEG00000019729
ENSODEG00000019731
ENSODEG00000019735
ENSODEG00000019737
ENSODEG00000019738
ENSODEG00000019739
ENSODEG00000019740
ENSODEG00000019742

ENSODEG00000019744
ENSODEG00000019745
ENSODEG00000019746
ENSODEG00000019747
ENSODEG00000019749
ENSODEG00000019750
ENSODEG00000019751
ENSODEG00000019752
ENSODEG00000019754
ENSODEG00000019755
ENSODEG00000019756
ENSODEG00000019757
ENSODEG00000019760
ENSODEG00000019762
ENSODEG00000019763
ENSODEG00000019764
ENSODEG00000019765
ENSODEG00000019766
ENSODEG00000019768
ENSODEG00000019770
ENSODEG00000019772
ENSODEG00000019773
ENSODEG00000019775
ENSODEG00000019776
ENSODEG00000019777
ENSODEG00000019778
ENSODEG00000019779
ENSODEG00000019780
ENSODEG00000019781
ENSODEG00000019782
ENSODEG00000019783
ENSODEG00000019784
ENSODEG00000019786
ENSODEG00000019787
ENSODEG00000019788
ENSODEG00000019789
ENSODEG00000019791
ENSODEG00000019792
ENSODEG00000019794
ENSODEG00000019795
ENSODEG00000019797
ENSODEG00000019798
ENSODEG00000019799
ENSODEG00000019800
ENSODEG00000019801
ENSODEG00000019802
ENSODEG00000019803
ENSODEG00000019804
ENSODEG00000019805
ENSODEG00000019806
ENSODEG00000019807
ENSODEG00000019808
ENSODEG00000019811
ENSODEG00000019812
ENSODEG00000019813
ENSODEG00000019815
ENSODEG00000019819
ENSODEG00000019820
ENSODEG00000019821

ENSODEG00000019823
ENSODEG00000019824
ENSODEG00000019827
ENSODEG00000019828
ENSODEG00000019829
ENSODEG00000019830
ENSODEG00000019832
ENSODEG00000019833
ENSODEG00000019834
ENSODEG00000019835
ENSODEG00000019836
ENSODEG00000019838
ENSODEG00000019839
ENSODEG00000019840
ENSODEG00000019842
ENSODEG00000019843
ENSODEG00000019844
ENSODEG00000019845
ENSODEG00000019846
ENSODEG00000019847
ENSODEG00000019848
ENSODEG00000019850
ENSODEG00000019851
ENSODEG00000019852
ENSODEG00000019855
ENSODEG00000019856
ENSODEG00000019857
ENSODEG00000019858
ENSODEG00000019859
ENSODEG00000019860
ENSODEG00000019862
ENSODEG00000019863
ENSODEG00000019864
ENSODEG00000019865
ENSODEG00000019866
ENSODEG00000019868
ENSODEG00000019871
ENSODEG00000019872
ENSODEG00000019874
ENSODEG00000019875
ENSODEG00000019876
ENSODEG00000019877
ENSODEG00000019878
ENSODEG00000019879
ENSODEG00000019880
ENSODEG00000019882
ENSODEG00000019883
ENSODEG00000019884
ENSODEG00000019885
ENSODEG00000019886
ENSODEG00000019887
ENSODEG00000019888
ENSODEG00000019889
ENSODEG00000019890
ENSODEG00000019892
ENSODEG00000019894
ENSODEG00000019895
ENSODEG00000019896
ENSODEG00000019897

ENSODEG00000019898
ENSODEG00000019899
ENSODEG00000019901
ENSODEG00000019902
ENSODEG00000019903
ENSODEG00000019904
ENSODEG00000019907
ENSODEG00000019909
ENSODEG00000019910
ENSODEG00000019911
ENSODEG00000019912
ENSODEG00000019913
ENSODEG00000019914
ENSODEG00000019915
ENSODEG00000019916
ENSODEG00000019917
ENSODEG00000019919
ENSODEG00000019920
ENSODEG00000019921
ENSODEG00000019922
ENSODEG00000019923
ENSODEG00000019924
ENSODEG00000019925
ENSODEG00000019926
ENSODEG00000019927
ENSODEG00000019931
ENSODEG00000019932
ENSODEG00000019934
ENSODEG00000019935
ENSODEG00000019938
ENSODEG00000019939
ENSODEG00000019940
ENSODEG00000019941
ENSODEG00000019942
ENSODEG00000019943
ENSODEG00000019945
ENSODEG00000019947
ENSODEG00000019948
ENSODEG00000019949
ENSODEG00000019950
ENSODEG00000019951
ENSODEG00000019952
ENSODEG00000019953
ENSODEG00000019954
ENSODEG00000019956
ENSODEG00000019959
ENSODEG00000019960
ENSODEG00000019962
ENSODEG00000019964
ENSODEG00000019965
ENSODEG00000019966
ENSODEG00000019967
ENSODEG00000019968
ENSODEG00000019969
ENSODEG00000019971
ENSODEG00000019972
ENSODEG00000019974
ENSODEG00000019976
ENSODEG00000019977

ENSODEG00000019978
ENSODEG00000019979
ENSODEG00000019980
ENSODEG00000019981
ENSODEG00000019982
ENSODEG00000019985
ENSODEG00000019986
ENSODEG00000019987
ENSODEG00000019988
ENSODEG00000019989
ENSODEG00000019990
ENSODEG00000019991
ENSODEG00000019992
ENSODEG00000019993
ENSODEG00000019996
ENSODEG00000019997
ENSODEG00000019998
ENSODEG00000019999
ENSODEG00000020000
ENSODEG00000020001
ENSODEG00000020005
ENSODEG00000020006
ENSODEG00000020007
ENSODEG00000020008
ENSODEG00000020009
ENSODEG00000020011
ENSODEG00000020013
ENSODEG00000020016
ENSODEG00000020017
ENSODEG00000020018
ENSODEG00000020019
ENSODEG00000020020
ENSODEG00000020021
ENSODEG00000020022
ENSODEG00000020024
ENSODEG00000020026
ENSODEG00000020028
ENSODEG00000020029
ENSODEG00000020030
ENSODEG00000020031
ENSODEG00000020032
ENSODEG00000020033
ENSODEG00000020034
ENSODEG00000020035
ENSODEG00000020036
ENSODEG00000020037
ENSODEG00000020038
ENSODEG00000020039
ENSODEG00000020041
ENSODEG00000020042
ENSODEG00000020043
ENSODEG00000020044
ENSODEG00000020046
ENSODEG00000020047
ENSODEG00000020048
ENSODEG00000020050
ENSODEG00000020051
ENSODEG00000020053
ENSODEG00000020054

ENSODEG00000020056
ENSODEG00000020058
ENSODEG00000020059
ENSODEG00000020061
ENSODEG00000020062
ENSODEG00000020063
ENSODEG00000020064
ENSODEG00000020065
ENSODEG00000020066
ENSODEG00000020067
ENSODEG00000020068
ENSODEG00000020071
ENSODEG00000020075
ENSODEG00000020076
ENSODEG00000020078
ENSODEG00000020080
ENSODEG00000020081
ENSODEG00000020083
ENSODEG00000020084
ENSODEG00000020085
ENSODEG00000020086
ENSODEG00000020089
ENSODEG00000020090
ENSODEG00000020091
ENSODEG00000020092
ENSODEG00000020093
ENSODEG00000020094
ENSODEG00000020096
ENSODEG00000020097
ENSODEG00000020099
ENSODEG00000020101
ENSODEG00000020102
ENSODEG00000020103
ENSODEG00000020104
ENSODEG00000020105
ENSODEG00000020107
ENSODEG00000020110
ENSODEG00000020111
ENSODEG00000020112
ENSODEG00000020113
ENSODEG00000020115
ENSODEG00000020116
ENSODEG00000020117
ENSODEG00000020118
ENSODEG00000020120
ENSODEG00000020121
ENSODEG00000020123
ENSODEG00000020125
ENSODEG00000020126
ENSODEG00000020127
ENSODEG00000020129
ENSODEG00000020132
ENSODEG00000020133
ENSODEG00000020134
ENSODEG00000020135
ENSODEG00000020136
ENSODEG00000020137
ENSODEG00000020138
ENSODEG00000020139

ENSODEG00000020140
ENSODEG00000020141
ENSODEG00000020144
ENSODEG00000020145
ENSODEG00000020146
ENSODEG00000020147
ENSODEG00000020148
ENSODEG00000020149
ENSODEG00000020150
ENSODEG00000020151
ENSODEG00000020152
ENSODEG00000020154
ENSODEG00000020155
ENSODEG00000020156
ENSODEG00000020157
ENSODEG00000020158
ENSODEG00000020160
ENSODEG00000020161
ENSODEG00000020162
ENSODEG00000020163
ENSODEG00000020164
ENSODEG00000020165
ENSODEG00000020166
ENSODEG00000020167
ENSODEG00000020168
ENSODEG00000020169
ENSODEG00000020171
ENSODEG00000020172
ENSODEG00000020175
ENSODEG00000020176
ENSODEG00000020177
ENSODEG00000020178
ENSODEG00000020179
ENSODEG00000020180
ENSODEG00000020181
ENSODEG00000020182
ENSODEG00000020183
ENSODEG00000020185
ENSODEG00000020187
ENSODEG00000020188
ENSODEG00000020189
ENSODEG00000020190
ENSODEG00000020191
ENSODEG00000020192
ENSODEG00000020193
ENSODEG00000020196
ENSODEG00000020198
ENSODEG00000020201
ENSODEG00000020203
ENSODEG00000020204
ENSODEG00000020205
ENSODEG00000020206
ENSODEG00000020207
ENSODEG00000020209
ENSODEG00000020210
ENSODEG00000020211
ENSODEG00000020212
ENSODEG00000020213
ENSODEG00000020216

ENSODEG00000020217
ENSODEG00000020218
ENSODEG00000020219
ENSODEG00000020221
ENSODEG00000020223
ENSODEG00000020224
ENSODEG00000020225
ENSODEG00000020228
ENSODEG00000020229
ENSODEG00000020231
ENSODEG00000020233
ENSODEG00000020235
ENSODEG00000020236
ENSODEG00000020237
ENSODEG00000020238
ENSODEG00000020239
ENSODEG00000020242
ENSODEG00000020245
ENSODEG00000020246
ENSODEG00000020247
ENSODEG00000020248
ENSODEG00000020249
ENSODEG00000020252
ENSODEG00000020253
ENSODEG00000020254
ENSODEG00000020256
ENSODEG00000020257
ENSODEG00000020259
ENSODEG00000020261
ENSODEG00000020263
ENSODEG00000020264
ENSODEG00000020265
ENSODEG00000020268
ENSODEG00000020269
ENSODEG00000020273
ENSODEG00000020274
ENSODEG00000020275
ENSODEG00000020277
ENSODEG00000020278
ENSODEG00000020279
ENSODEG00000020280
ENSODEG00000020283
ENSODEG00000020284
ENSODEG00000020285
ENSODEG00000020286
ENSODEG00000020287
ENSODEG00000020288
ENSODEG00000020289
ENSODEG00000020290
ENSODEG00000020291
ENSODEG00000020292
ENSODEG00000020293
ENSODEG00000020294
ENSODEG00000020295
ENSODEG00000020296
ENSODEG00000020297
ENSODEG00000020298
ENSODEG00000020300
ENSODEG00000020301

ENSODEG00000020302
ENSODEG00000020303
ENSODEG00000020304
ENSODEG00000020305
ENSODEG00000020306
ENSODEG00000020309
ENSODEG00000020310
ENSODEG00000020313
ENSODEG00000020314
ENSODEG00000020315
ENSODEG00000020316
ENSODEG00000020317
ENSODEG00000020320
ENSODEG00000020322
ENSODEG00000020323
ENSODEG00000020324
ENSODEG00000020325
ENSODEG00000020326
ENSODEG00000020327
ENSODEG00000020328
ENSODEG00000020329
ENSODEG00000020330
ENSODEG00000020331
ENSODEG00000020332
ENSODEG00000020333
ENSODEG00000020336
ENSODEG00000020337
ENSODEG00000020338
ENSODEG00000020339
ENSODEG00000020340
ENSODEG00000020343
ENSODEG00000020344
ENSODEG00000020345
ENSODEG00000020346
ENSODEG00000020348
ENSODEG00000020349
ENSODEG00000020350
ENSODEG00000020351
ENSODEG00000020355
ENSODEG00000020358
ENSODEG00000020359
ENSODEG00000020360
ENSODEG00000020362
ENSODEG00000020365
ENSODEG00000020366
ENSODEG00000020367
ENSODEG00000020368
ENSODEG00000020369
ENSODEG00000020370
ENSODEG00000020371
ENSODEG00000020372
ENSODEG00000020373
ENSODEG00000020374
ENSODEG00000020375
ENSODEG00000020377
ENSODEG00000020379
ENSODEG00000020381
ENSODEG00000020382
ENSODEG00000020383

ENSODEG00000020384
ENSODEG00000020386
ENSODEG00000020388
ENSODEG00000020389
ENSODEG00000020390
ENSODEG00000020392
ENSODEG00000020393
ENSODEG00000020394
ENSODEG00000020396
ENSODEG00000020397
ENSODEG00000020398
ENSODEG00000020399
ENSODEG00000020400
ENSODEG00000020401
ENSODEG00000020402
ENSODEG00000020403
ENSODEG00000020404
ENSODEG00000020406
ENSODEG00000020407
ENSODEG00000020408
ENSODEG00000020409
ENSODEG00000020411
ENSODEG00000020416
ENSODEG00000020417
ENSODEG00000020419
ENSODEG00000020420
ENSODEG00000020421
ENSODEG00000020422
ENSODEG00000020425
ENSODEG00000020426
ENSODEG00000020428
ENSODEG00000020429
ENSODEG00000020430
ENSODEG00000020431
ENSODEG00000020432
ENSODEG00000020433
ENSODEG00000020434
ENSODEG00000020436
ENSODEG00000020437
ENSODEG00000020438
ENSODEG00000020440
ENSODEG00000020442
ENSODEG00000020443
ENSODEG00000020445
ENSODEG00000020446
ENSODEG00000020447
ENSODEG00000020448
ENSODEG00000020450
ENSODEG00000020451
ENSODEG00000020452
ENSODEG00000020453
ENSODEG00000020455
ENSODEG00000020456
ENSODEG00000020457
ENSODEG00000020458
ENSODEG00000020459
ENSODEG00000020460
ENSODEG00000020462
ENSODEG00000020463

ENSODEG00000020466
ENSODEG00000020468
ENSODEG00000020469
ENSODEG00000020470
ENSODEG00000020472
ENSODEG00000020474
ENSODEG00000020475
ENSODEG00000020476
ENSODEG00000020477
ENSODEG00000020478
ENSODEG00000020479
ENSODEG00000020480
ENSODEG00000020483
ENSODEG00000020484
ENSODEG00000020485
ENSODEG00000020488
ENSODEG00000020489
ENSODEG00000020490
ENSODEG00000020491
ENSODEG00000020493
ENSODEG00000020494
ENSODEG00000020496
ENSODEG00000020498
ENSODEG00000020499
ENSODEG00000020500
ENSODEG00000020501
ENSODEG00000020502
ENSODEG00000020503
ENSODEG00000020509
ENSODEG00000020512
ENSODEG00000020513
ENSODEG00000020514
ENSODEG00000020515
ENSODEG00000020516
ENSODEG00000020517
ENSODEG00000020518
ENSODEG00000020520
ENSODEG00000020521
ENSODEG00000020522
ENSODEG00000020523
ENSODEG00000020524
ENSODEG00000020526
ENSODEG00000020527
ENSODEG00000020528
ENSODEG00000020529
ENSODEG00000020531
ENSODEG00000020532
ENSODEG00000020534
ENSODEG00000020535
ENSODEG00000020536
ENSODEG00000020538
ENSODEG00000020542
ENSODEG00000020543
ENSODEG00000020544
ENSODEG00000020545
ENSODEG00000020546
ENSODEG00000020547
ENSODEG00000020548
ENSODEG00000020549

ENSODEG00000020550
ENSODEG00000020554
ENSODEG00000020556
ENSODEG00000020557
ENSODEG00000020558
ENSODEG00000020563
ENSODEG00000020564
